# Synthesis, Molecular Docking and Biological Characterization of Pyrazine Linked 2-Aminobenzamides as New Class I Selective Histone Deacetylase (HDAC) Inhibitors with Anti-Leukemic Activity

**DOI:** 10.3390/ijms23010369

**Published:** 2021-12-29

**Authors:** Hany S. Ibrahim, Mohamed Abdelsalam, Yanira Zeyn, Matthes Zessin, Al-Hassan M. Mustafa, Marten A. Fischer, Patrik Zeyen, Ping Sun, Emre F. Bülbül, Anita Vecchio, Frank Erdmann, Matthias Schmidt, Dina Robaa, Cyril Barinka, Christophe Romier, Mike Schutkowski, Oliver H. Krämer, Wolfgang Sippl

**Affiliations:** 1 Department of Medicinal Chemistry, Institute of Pharmacy, Martin-Luther-University of Halle-Wittenberg, 06120 Halle (Saale), Germany; hany.ibrahim@pharmazie.uni-halle.de (H.S.I.); mohamed.abdelsalam@pharmazie.uni-halle.de (M.A.); matthes.zessin@googlemail.com (M.Z.); patrik.zeyen@gmail.com (P.Z.); ping.sun@pharmazie.uni-halle.de (P.S.); emre.bulbul@pharmazie.uni-halle.de (E.F.B.); anitavecchio95@gmail.com (A.V.); frank.erdmann@pharmazie.uni-halle.de (F.E.); matthias.schmidt@pharmazie.uni-halle.de (M.S.); dina.robaa@pharmazie.uni-halle.de (D.R.); 2 Department of Pharmaceutical Chemistry, Faculty of Pharmacy, Egyptian Russian University, Badr City, Cairo 11829, Egypt; 3 Department of Pharmaceutical Chemistry, Faculty of Pharmacy, Alexandria University, Alexandria 21521, Egypt; 4 Department of Toxicology, University Medical Center, 55131 Mainz, Germany; yanira.zeyn@uni-mainz.de (Y.Z.); alabdeen@uni-mainz.de (A.-H.M.M.); mfisch05@students.uni-mainz.de (M.A.F.); 5 Department of Enzymology, Institute of Biochemistry, Martin-Luther-University of Halle-Wittenberg, 06120 Halle (Saale), Germany; mike.schutkowski@biochemtech.uni-halle.de; 6 Department of Zoology, Faculty of Science, Aswan University, Aswan 81528, Egypt; 7 Institute of Biotechnology of the Czech Academy of Sciences, BIOCEV, Prumyslova 595, 25250 Vestec, Czech Republic; cyril.barinka@ibt.cas.cz; 8 Département de Biologie Structurale Intégrative, Institut de Génétique et de Biologie Moléculaire et Cellulaire (IGBMC), CNRS, INSERM, Université de Strasbourg, CEDEX, 67404 Illkirch, France; romier@igbmc.fr

**Keywords:** histone deacetylases, HDAC1, HDAC2, HDAC3, 2-aminobenzamides, SAR studies, acute myeloid leukemia (AML), docking

## Abstract

Class I histone deacetylases (HDACs) are key regulators of cell proliferation and they are frequently dysregulated in cancer cells. We report here the synthesis of a novel series of class-I selective HDAC inhibitors (HDACi) containing a 2-aminobenzamide moiety as a zinc-binding group connected with a central (piperazin-1-yl)pyrazine or (piperazin-1-yl)pyrimidine moiety. Some of the compounds were additionally substituted with an aromatic capping group. Compounds were tested in vitro against human HDAC1, 2, 3, and 8 enzymes and compared to reference class I HDACi (Entinostat (MS-275), Mocetinostat, CI994 and RGFP-966). The most promising compounds were found to be highly selective against HDAC1, 2 and 3 over the remaining HDAC subtypes from other classes. Molecular docking studies and MD simulations were performed to rationalize the in vitro data and to deduce a complete structure activity relationship (SAR) analysis of this novel series of class-I HDACi. The most potent compounds, including 19f, which blocks HDAC1, HDAC2, and HDAC3, as well as the selective HDAC1/HDAC2 inhibitors 21a and 29b, were selected for further cellular testing against human acute myeloid leukemia (AML) and erythroleukemic cancer (HEL) cells, taking into consideration their low toxicity against human embryonic HEK293 cells. We found that 19f is superior to the clinically tested class-I HDACi Entinostat (MS-275). Thus, 19f is a new and specific HDACi with the potential to eliminate blood cancer cells of various origins.

## 1. Introduction

Epigenetic regulation refers to heritable or long-term changes in gene expression that do not rely on an alteration of the DNA sequence [1]. Histone modification is a widely studied epigenetic modification, which involves the covalent alteration of histone tails through acetylation, methylation, phosphorylation, sumoylation, and ubiquitination [2]. Histone acetylation is one of the most well studied post-translational modifications. This process is controlled by the action of two opposing enzyme families. Histone acetyl transferases (HATs) catalyze the addition of the acetyl group on the protonated ε-amino group of lysine residues of histone proteins. This modification results in a loss of the positive charge and reduces the interactions between histone proteins and DNA [3]. The histone deacetylases (HDACs) catalyze the removal of acetyl groups and this can result in the formation of the condensed chromatin (heterochromatin) and a repression of gene transcription [3]. Human HDACs are classified according to their sequence homology and domain organization into four groups. These are the zinc-dependent deacetylases of class I (HDAC1, 2, 3, and 8), class II (HDAC4, 5, 6, 7, 9, and 10), and class IV (HDAC11), and the NAD+-dependent class III (sirtuins SIRT1-7) [4].

Class I (HDACs 1, 2, 3, and 8) are located mainly in the nucleus and have an essential role in cell proliferation, cell cycle progression and the establishment and maintenance of the aberrant phenotype of cancer cells [5]. Hypoacetylation of histone H4 is a common distinctive feature in early stages of human cancer [6]. Given that HDACs are important for tumor development and progression, HDAC inhibitors (HDACi) have been developed and studied as potential anticancer therapeutics over recent years [7]. HDACi have been tested against solid tumors and blood malignancies. Four class I/II/IV HDACi (pan-HDACi) were approved by authorities for the treatment of patients with cutaneous/peripheral T-cell lymphoma and multiple myeloma [8]. HDACi can be classified according to their zinc binding groups (ZBG) into five main groups: hydroxamates, 2-aminobenzamides, cyclic peptides, thiols, short-chain fatty acids, ketones and others [9]. The hydroxamate-based HDACi Vorinostat (SAHA) [10], Belinostat (PXD101), Panobinostat (LBH589), as well as the thiol-prodrug/depsipeptide Romidepsin (FK228) have been approved by the US Food and Drug Administration (FDA) for the treatment of different types of cancer and hematological malignancies [11,12,13]. Promising clinical results in phase I/II clinical trials were also obtained the pan-HDACi Givinostat in patients with the blood disorder polycythemia vera [14].

**Figure 1:**
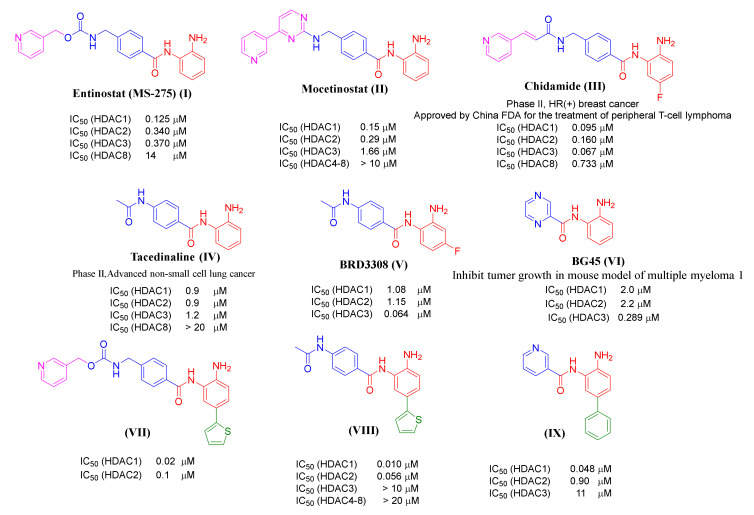
Examples of previously reported 2-aminobenzamides and their inhibitory activity towards different HDAC subtypes. The different pharmacophoric groups of the inhibitors are marked in color (ZBG in red, substitutions interacting with the HDAC1/HDAC2 foot pocket in green, linker colored in blue, capping group colored in pink).

Remarkably, the modulation of HDAC1, HDAC2, and HDAC3 presents a specific possibility to interfere with signaling pathways that are hijacked by tumor cells, and class-I HDACs are highly expressed in different cancers, including leukemia [15,16,17,18,19]. Class-I selective inhibitors have already reached clinical studies like MS-275 (I), phase II Hodgkin’s lymphoma, or Mocetinostat (II), and phase II in relapsed lymphoma (Figure 1) [20,21,22]. Notably, normal cells are largely unaffected by HDACi which verifies that HDACs are key for the development and maintenance of the tumor cell phenotype [23]. To fully exploit and achieve clinical expectations on these drugs, more potent and specific HDACi are required [24,25].

Most HDACi have a common pharmacophore consisting of three different fragments as follows: a ZBG, a capping group, and a linker connecting both groups [26]. The ZBG is responsible for chelating Zn^2+^ in the active site of HDACs. Modification of the ZBG often changes the potency of inhibitors significantly [27]. The capping group usually includes hydrophobic/bulky moieties, such as aromatic or heteroaromatic groups, mediating interactions at the rim of the HDAC enzyme. The interactions with the residues at the entrance of the binding pocket were also shown to contribute to HDAC subtype selectivity [28].

Although the hydroxamic acid is an often used and potent ZBG, it was observed that hydroxamic acid-based HDAC inhibitors often lack cellular potency and often show off-target effects [29,30]. Additionally, the progress of cell mutagenicity and genotoxicity by such compounds is still a main factor to exclude many potent candidates from further drug development steps [31].

It has been shown that 2-Aminobenzamides improve HDAC class I selectivity and strongly inhibit the class-I HDACs1, -2, and -3. The 2-aminobenzamide group acts as ZBG instead of the hydroxamate group in other HDACi. This replacement allows them to have selectivity towards the HDAC subtypes HDAC1, HDAC2, and HDAC3. According to the general skeleton of 2-aminobenzamides, such as HDACi, Entinostat (MS-275, I) and Mocetinostat (II) have an unsubstituted 2-aminobenzamide scaffold as ZBG connected through a linker to a pyridine ring acting as a capping group (Figure 1) [32,33]. Chidamide (CS005, III) shows the same general skeleton with an additional fluoro substitution on the 2-aminobenzamide moiety [34,35]. It has been found that 2-Aminobenzamides Tacedinaline (CI-994, IV), BRD3308 (V) and BG45 (VI) are examples of inhibitors without a capping group (Figure 1) [36,37,38]. The crystal structure of HDAC2 revealed that the 2-aminobenzamide part could access the foot pocket next to the catalytic region [39]. In this regard, another strategy to increase the selectivity towards HDAC1 and HDAC2 subtypes is the addition of an aromatic moiety, like thienyl or phenyl, to the position-5 of the 2-aminobenzamide group. This fills the internal cavity of the foot pocket near to the catalytic region, as in the case of compound VII–IX. These inhibitors are more active and selective against HDAC1/HDAC2 over HDAC3 (Figure 1) [40,41].

**Figure 2:**
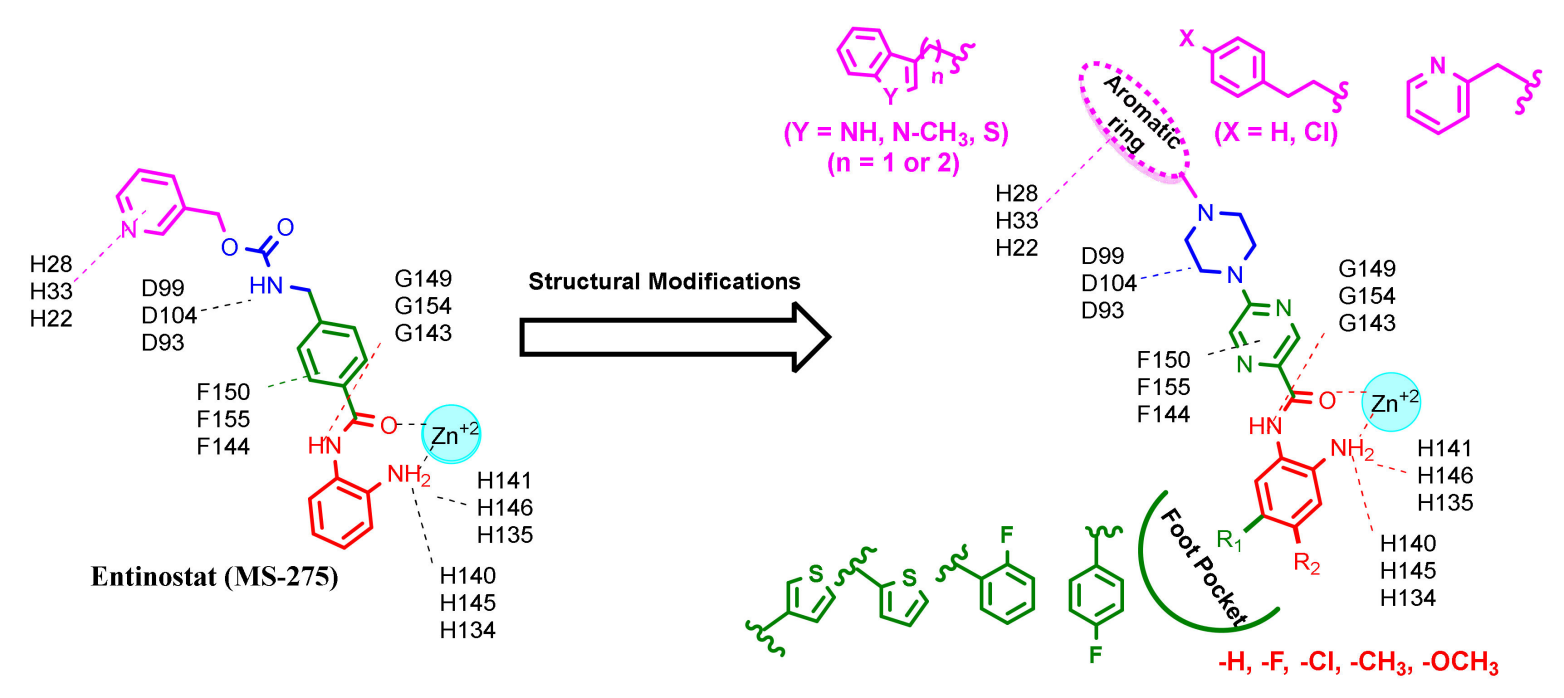
Structural modifications of Entinostat (MS-275) based on its interaction with the active site of HDAC 1 (red amino acid labels), HDAC 2 (blue amino acid labels) and HDAC 3 (green amino acid labels) subtypes to design novel compounds with different substitutions to obtain full SAR studies.

The aim of the current study was to develop novel class-I selective HDAC inhibitors with improved in vitro activity as well as stronger anti-leukemic effects compared to known reference inhibitors. Due to the above mentioned problems with hydroxamic acids and the good class-I selectivity of 2-aminobenzamides shown by, e.g., MS-275 [42], we focused on this chemotype. A first idea was to substitute the middle phenyl ring of MS-275 with more polar pyrazine or pyrimidine rings to result in better solubility of the final compounds. At the beginning of the study, we first docked the reference inhibitors shown in Figure 1 to the available crystal structures of HDAC1, HDAC2 and HDAC3 to obtain ideas for structural optimization. For example, attachment of a basic piperazine to a pyrimidine or pyrazine ring mimicking the middle ring of MS-275 showed ionic hydrogen bonding with an aspartate residue conserved in HDAC1, HDAC2 and HDAC3 (D99 in HDAC1, D104 in HDAC2, D93 in HDAC3). The capping groups of reported HDAC inhibitors interact with amino acids at the rim of the binding channel; in the case of MS-275, these are aromatic and hydrophobic interactions with H27/33/22 and P29/34/23 in HDAC1, HDAC2 and HDAC3, respectively (Supplementary Figure S1). Accordingly, we designed compounds with different aromatic rings as capping groups and different linker lengths to analyze the effect of capping groups on class-I HDAC activity. In addition, the 2-aminobenzamide scaffold was substituted at different positions to investigate the effect of increased subtype selectivity toward HDAC1/HDAC2 on the anti-leukemic activity. Mono- or di-substitutions with different small groups at position 4 and/or position 5 were performed to complete the SAR studies. The realized structural changes are summarized in Figure 2.

## 2. Results and Discussion

### 2.1. Chemistry

**Scheme 1:**
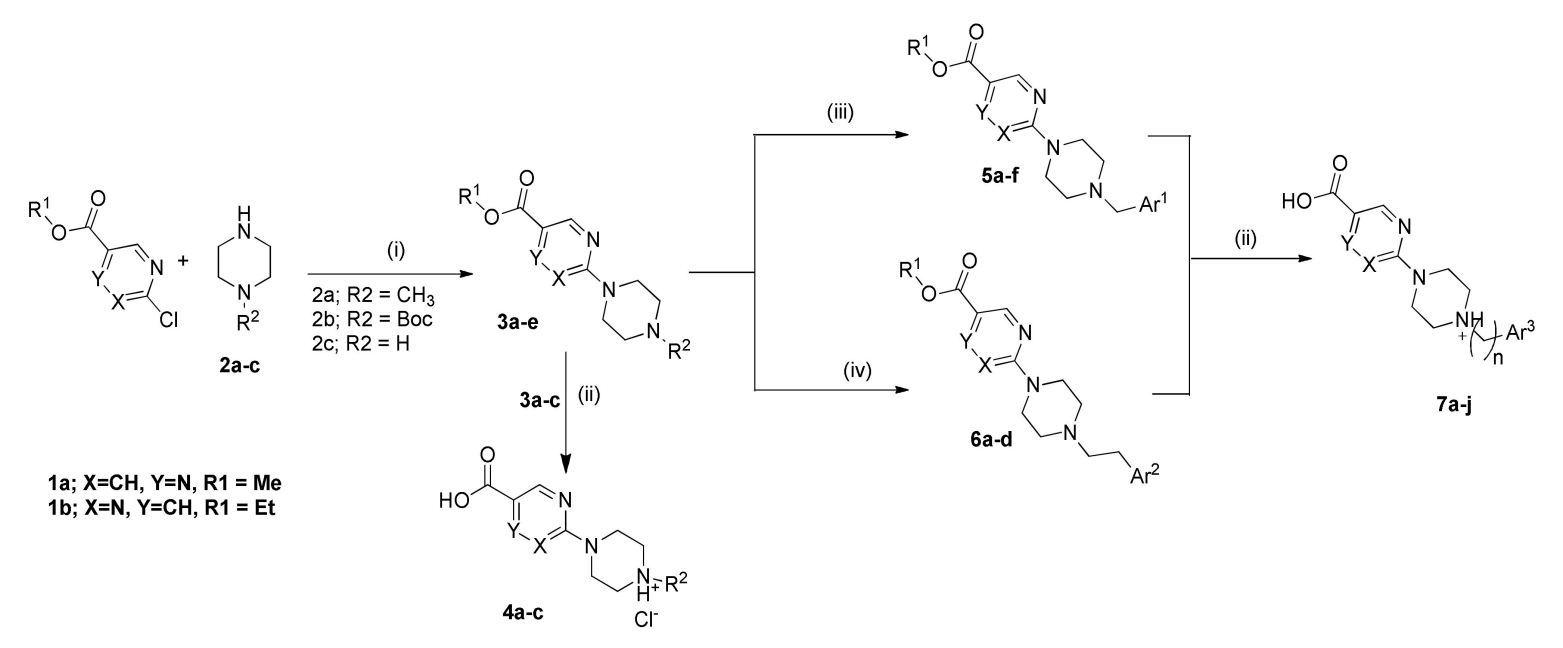
Synthesis of carboxylic acid intermediates **4a**–**c** and **7a**–**j**. Reagents and conditions: (**i**) Toluene, 130 °C, 1 h. (**ii**) Compounds **3a**–**c**, 2.5 eq 1N NaOH, MeOH, 70 °C, 3 h. (**iii**) Compounds **3d**,**e**, Ar^1^-CHO, Na(OAc)_3_BH, AcOH, DCM, RT, 5 h. (**iv**) Compound **3d**, BrCH_2_CH_2_Ar^2^, K_2_CO_3_, CH_3_CN, M.W, 120 °C, 90 min.

**Scheme 2:**
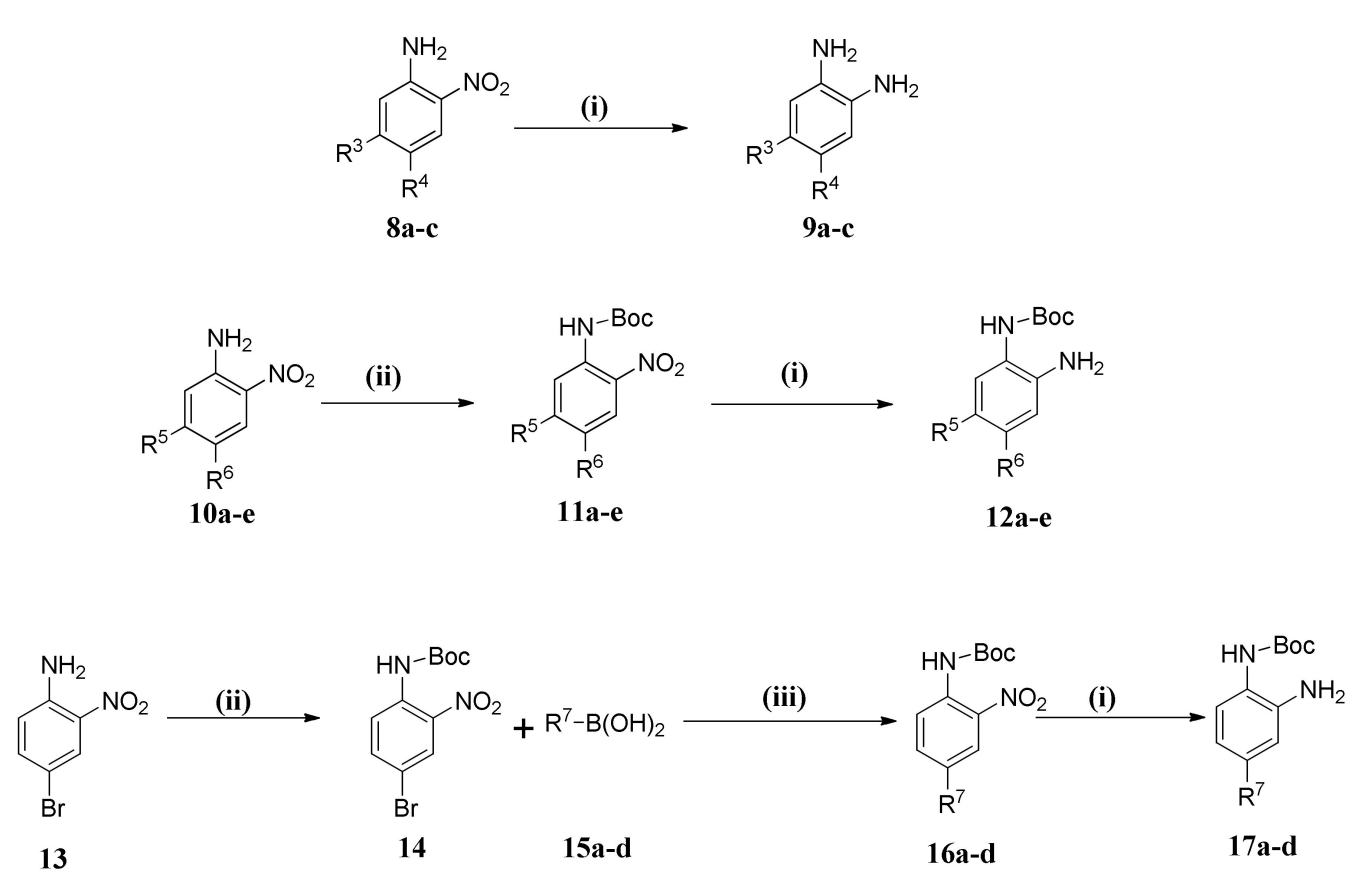
Synthesis of 1,2-phenylenediamine derivatives **9a**–**c**, **12a**–**e** and 1**7a**–**d**. Reagents and conditions: (**i**) HCOONH_4_, Pd/C 10%, MeOH. (**ii**) Boc_2_O, TEA, DMAP, DCM (**iii**) Tetrakis P(Ph)_3_Pd, Deg. DME, Na_2_CO_3_.

To obtain the designed compounds, we planned a three-step synthesis. The first step was the synthesis of carboxylic acid derivatives 4a–c and 7a–j, as illustrated in Scheme 1, (Supplementary Table S1a,b). The second step was the synthesis of different *o*-phenylenediamine derivatives (9a–c, 12a–e and 17a–d) as shown in Scheme 2. Finally, coupling of obtained acid derivatives and *o*-phenylenediamines was carried out, using Boc protection and de-protection, to obtain the final compounds 19a–o, 21a–c, 23a–c, 25a,b, 27a–c and 29a–d.

As shown in Scheme 1, esters 3a–e were obtained by direct alkylation of piperazine derivatives 2a–c using methyl 5-chloropyrazine-2-carboxylate (1a) or ethyl 2-chloropyrimidine-5-carboxylate (1b) in refluxing toluene. Esters 3d,e with a free piperazinyl moiety were further extended to the capping groups either by reductive amination or microwave-assisted alkylation, to yield the corresponding N-alkylated derivatives 5a–f and 6a–d. All synthesized esters in this scheme were converted into acids through alkaline hydrolysis by heating in 2.5 eq. 1N aq. NaOH.

Scheme 2, (Supplementary Table S2) illustrates the synthesis of three different types of 1,2-phenylenediamine derivatives. The first type includes free 1,2-phenylenediamine derivatives (9a–c), which were prepared by catalytic reduction of the corresponding nitro compounds (8a–c) using ammonium formate and Pd/C 10%. The second type (12a–e), which are Boc protected with simple substituents, were synthesized in two steps by protection of the amino group in the corresponding nitroaniline derivatives (10a–e) using Boc_2_O in presence of Et_3_N and DMAP (N,N-Dimethylpyridin-4-amin) followed by catalytic hydrogenation as previously described to produce the corresponding aniline derivative (12a–e). The third type represents mono Boc protected-1,2-phenylenediamines with aryl substituents (R^7^). These compounds were prepared through Suzuki coupling between the Boc protected-4-bromo-2-nitroaniline and the appropriate aromatic boronic acid by refluxing in 1,2-dimethoxyethane using tetrakis (triphenylphosphine)palladium(0) as a catalyst, followed by reduction of the nitro group to the corresponding aniline derivative (17a–d).

**Scheme 3:**
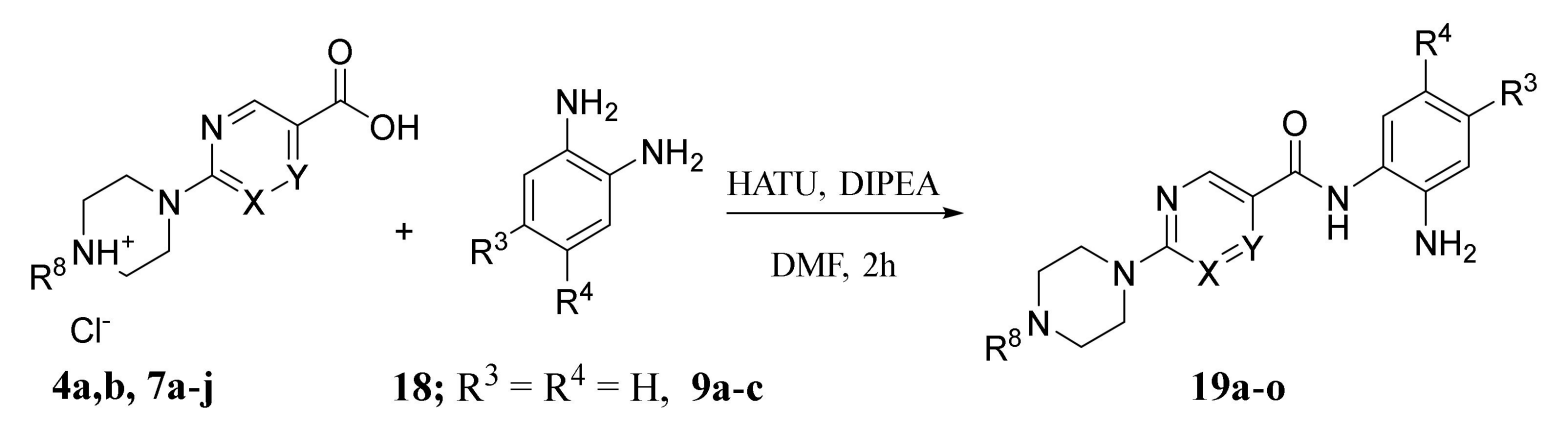
Synthesis of the final compounds **19a**–**o**. Reagents and conditions: HATU, DIPEA, DMF, 1–2 h, RT.

The third main step in the synthetic pathway to prepare the 2-aminoanilide compounds was an amide coupling between the carboxylic acid derivatives and 1,2-phenylenediamines using the coupling reagent HATU (O--N, N, N′, N′-tetramethyluronium-hexafluorphosphate) in the presence of DIPEA (N,N-Diisopropylethylamine) as the base. The time of this reaction depends on the reactivity of the amino group of the 1,2-phenylenediamine derivative. The coupling reaction to yield 19a–o, as shown in Scheme 3, (Supplementary Table S3) takes from 1 h to 2 h.

**Scheme 4:**
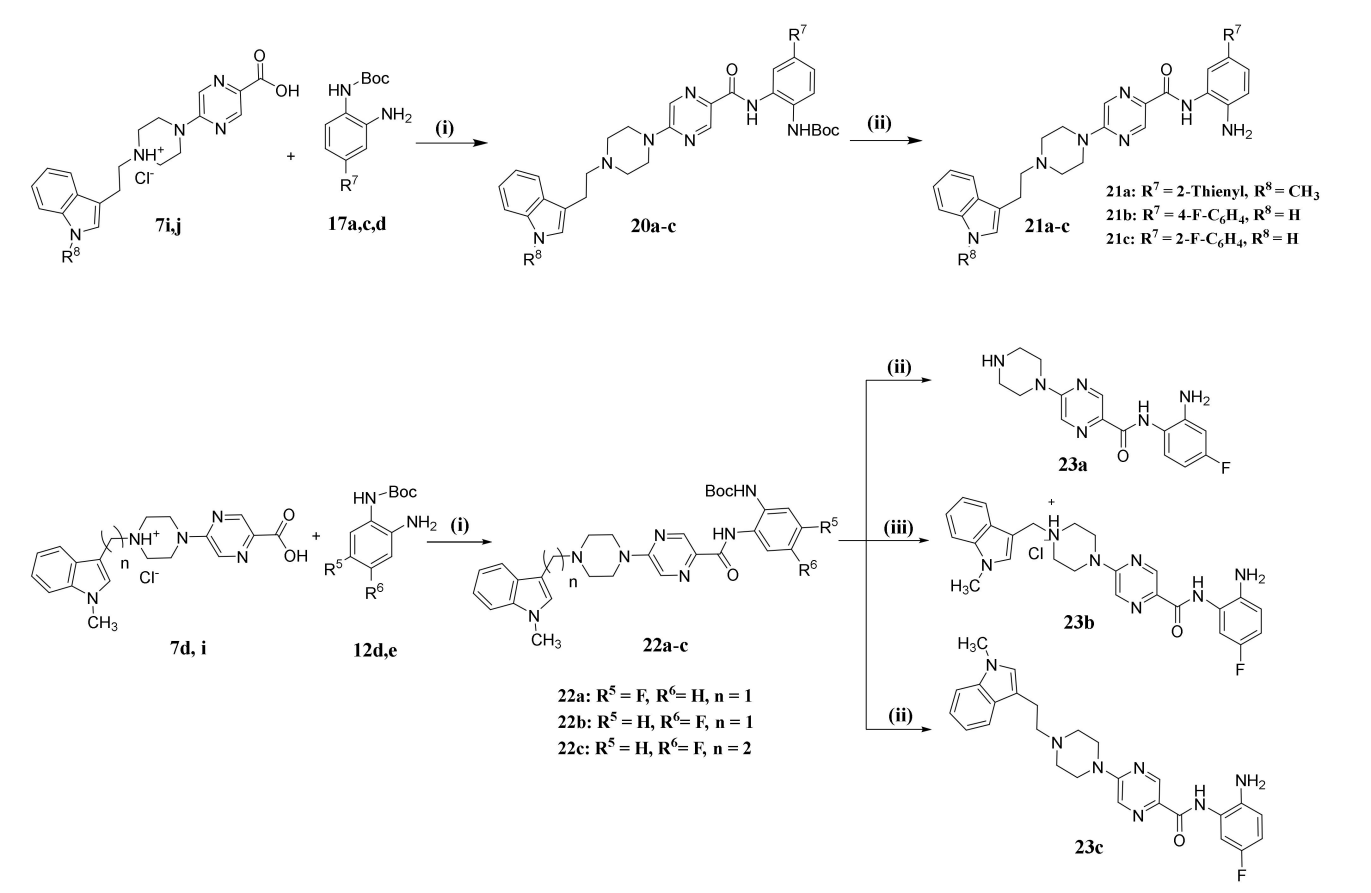
Synthesis of the final compounds **21a**–**c** and **23a**–**c**. Reagents and conditions: (**i**) HATU, DIPEA, Dry DMF, RT, Overnight. (**ii**) TFA, DCM, 0 °C, 30 min. (**iii**) **22b**, 4M HCl, Dioxane, 0 °C, 30 min.

**Scheme 5:**
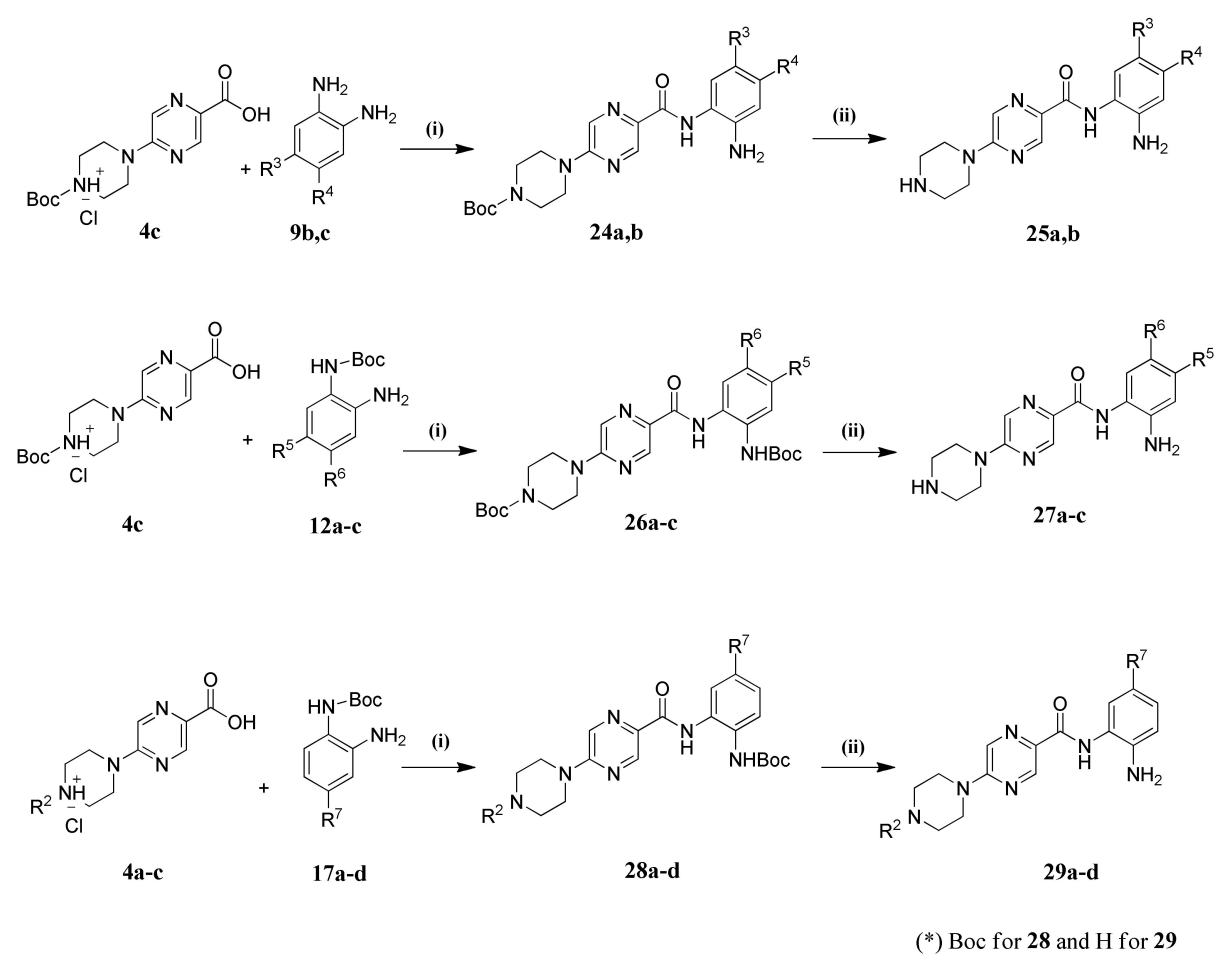
Synthesis of the final compounds **25a**,**b**, **27a**–**c** and **29a**–**d**. Reagents and conditions: (**i**) HATU, DIPEA, Dry DMF, RT. (**ii**) TFA, DCM, 0 °C, 30 min.

Coupling of the *o*-phenylene-diamines in which one of the amino groups is protected by Boc proceeded as previously described using HATU and DIPEA as a catalyst. However, this reaction needed overnight stirring at room temperature due to the low reactivity of the free amino group in compounds 20a–c. This reaction was followed by a deprotection step using trifluoroacetic acid (TFA) to obtain compounds 21a–c (Scheme 4). The deprotection step had an effect on compounds having a Mannich base in the capping group, as in compound 22a. In this case it was removed from the compound due to the acidic effect of TFA to yield compound 23a (Scheme 4). In another trial to make Boc deprotection for compound 22b using 4M HCl in dioxane, the capping group was also unstable. Therefore, we could only obtain the target compound 23b (with capping group) in a very low yield (5%) by preparative HPLC. On the other hand, to avoid the problem of the instability of the Mannich bases toward acidic condition required for Boc deprotection, the methylene carbon connecting the indole capping group and piperazine scaffold was replaced by an ethylene linker. We obtained the target compound 23c after Boc deprotection using TFA without any hydrolysis of the capping group (Scheme 5).

The cleavage of the capping group in compound 23a and its promising inhibitory activity against HDAC1, 2 and 3 motivated us to synthesize a series of compounds lacking the capping group as in case of 25a,b, 27a–c and 29b,c. This was achieved using Boc-protected piperazine intermediates 24a,b, 26a–c and 28b,c followed by deprotection using TFA (Scheme 5), (Supplementary Table S4). To extend our SAR studies, we synthesized further N-methyl piperazine derivatives (29a,d) using the previously established procedure, as illustrated in Scheme 5 (Supplementary Table S4).

### 2.2. Biological Evaluation

#### In Vitro Testing of HDAC Inhibitory Activity

The synthesized compounds were tested for inhibitory activity against human class I HDACs (HDAC1, -2, -3, and -8) using a fluorogenic peptide derived from p53 (Ac-RHKK(Acetyl)-AMC), as shown in Table 1 [43]. We included several reported inhibitors of HDAC1, HDAC2, and HDAC3 (CI994, MS-275, Mocetinostat and RGFP966) as reference compounds. The most promising inhibitors were also tested against a panel of HDAC subtypes (including HDAC4, -5, -6, -7, -9 and -11) and, as expected, none of these compounds showed strong inhibition of the other HDACs, as shown in Table 2.

The synthesized compounds can be categorized into four main groups based on the substitution pattern of the 2-aminobenzamide scaffold and presence or absence of capping groups. The first group comprises compounds with capping groups and unsubstituted 2-aminobenzamide scaffolds. The second group includes compounds with capping groups and substituted 2-aminobenzamide scaffolds. The third group contains compounds with unsubstituted 2-aminobenzamide groups without any capping group. The final group contains compounds with substituted 2-aminobenzamide functionalities and that lack a capping group.

Generally, the first group of compounds inhibit HDAC1, HDAC2, and HDAC3 in the low to submicromolar range. For instance, compound 19f, which has the 3-indolyl ring as a capping group, showed very good inhibitory activity against HDAC1, -2, and -3 with IC_50_ values (0.13 µM, 0.28 µM, 0.31 µM, respectively). It was found that 19f was more potent than the reference inhibitors MS-275, Mocetinostat, CI994 and RGFP-966. Modification in the capping group has different effects on the HDAC activity. For example, compound 19k with an N-methyl-3-indolyl ring has similar inhibitory profile with IC_50_ values of 0.14 µM, 0.56 µM, and 0.59 µM for HDAC1, HDAC2, and HDAC3, respectively). Similarly, 19e, which has a benzothiophene capping group, showed IC_50_ values of 0.21 µM, 0.71 µM, and 0.84 µM against HDAC1, HDAC2, and HDAC3, respectively. On the other hand, other capping groups, like phenethyl, 4-chlorophenethyl or 2-pyridyl, resulted in a reduced inhibitory activity, as in the case of compound 19b or slightly decreased activities, as in the case of compound 19a and 19d (Table 1).

Interestingly, the replacement of the pyrazine linker with pyrimidine resulted in a significant decrease of inhibitory activity, as in the case of compound 19c compared to compound 19d, or a slight decrease of HDAC1, HDAC2, and HDAC3 inhibitory activities, as in the case of compound 19j compared to compound 19k. It was also noticed that replacing the methylene connecting the indole capping group and piperazine with an ethylene resulted in a slight decrease in the HDAC inhibitory activity, as in the case of compound 19g compared with 19f (Table 1).

In the second group of the designed compounds, we investigated the effect of different substitution patterns of the 2-aminobenzamide on HDAC selectivity in the presence of 3-indolyl or (N-methyl)-3-indolyl capping groups. In line with reported studies, it was observed that the substitution of 2-aminobenzamides with aromatic or heterocyclic rings at position-5 improved HDAC subtype selectivity. For instance, compound 21a, which has a 2-thienyl ring at the position-5, has high selectivity for HDAC1 and HDAC2 (0.26 µM, 2.47 µM, respectively) over HDAC3. In addition, 21a is more potent than the reference inhibitors MS-275, Mocetinostat, CI994 and RGFP-966. In a similar manner, compounds 21b,c with 4- or 2-fluorophenyl substituents have a significant selectivity toward HDAC1/HDAC2 over HDAC3 compared to the parent unsubstituted derivative 19g. Compound 21b and 21c displayed submicromolar IC_50_ values in the case of HDAC1, and HDAC2 and only weak or no inhibition of HDAC3 (Table 1).

On the contrary, substitution of the 2-aminobenzamide with halogens did not result in marked improvement on HDAC subtype selectivity. For example, compound 19l and 19m, having mono- or disubstituted fluorophenyl, have almost similar subtype selectivity compared to their unsubstituted parent derivative 19k. In the case of compound 19i having a mono chloro substituent, it showed a decrease in HDAC inhibition compared to the unsubstituted parent derivative 19g.

The third group of synthesized compounds lacks the capping group and any substitution on the 2-aminobenzamide group. Compounds 19n and 19o showed only very weak enzymatic activity against HDAC1, HDAC2, and HDAC3.

The last group of inhibitors contains different substitution patterns on the phenyl ring of the ZBG and no capping group. Generally, the compounds possessing aromatic substituents at position-5, like 2-thienyl, 3-thienyl, 4-flourophenyl, and 2-fluorophenyl, showed good HDAC subtype selectivity and potency. Compound 29b showed strong inhibitory activity against HDAC1 and HDAC2 (IC_50_; 0.07 µM, 0.26 µM, respectively), with little activity against HDAC3 (IC_50_; 6.1 µM). Replacing the aromatic ring with trifluoromethyl group, as in compound 27a, diminished the HDAC inhibitory activity (Table 1). On the other hand, substitution of the phenyl group with a fluorine atom at position-4, resulted in a slight increase of HDAC3 selectivity over HDAC1 and HDAC2, as in case of compound 23a with IC_50_ values for HDAC1, HDAC2, and HDAC3 being 3.30 µM, 2.17 µM, and 0.40 µM, respectively. Similarly, replacing the fluorine atom with a chlorine atom, as in compound 25a, resulted in complete loss of inhibitory activity against HDAC1/HDAC2 and decreased activity against HDAC3 (IC_50_ 8.7 µM). Furthermore, replacing the fluorine atom with electron donating groups, like the methyl or methoxy group, resulted in a dramatic loss of HDAC inhibitory activity, as in the case of compound 27b,c (Table 1).

### 2.3. Molecular Docking Studies

To rationalize the binding mode of the synthesized compounds, we performed molecular docking studies using crystal structures of HDAC1 (PDB ID: 4BKX, apo-form), HDAC2 (PDB ID: 4LY1, co-crystallized with a 2-aminobenzamide), and HDAC3 (PDB ID: 4A69, apo-form) (Supplementary Figure S1).

**Figure 3:**
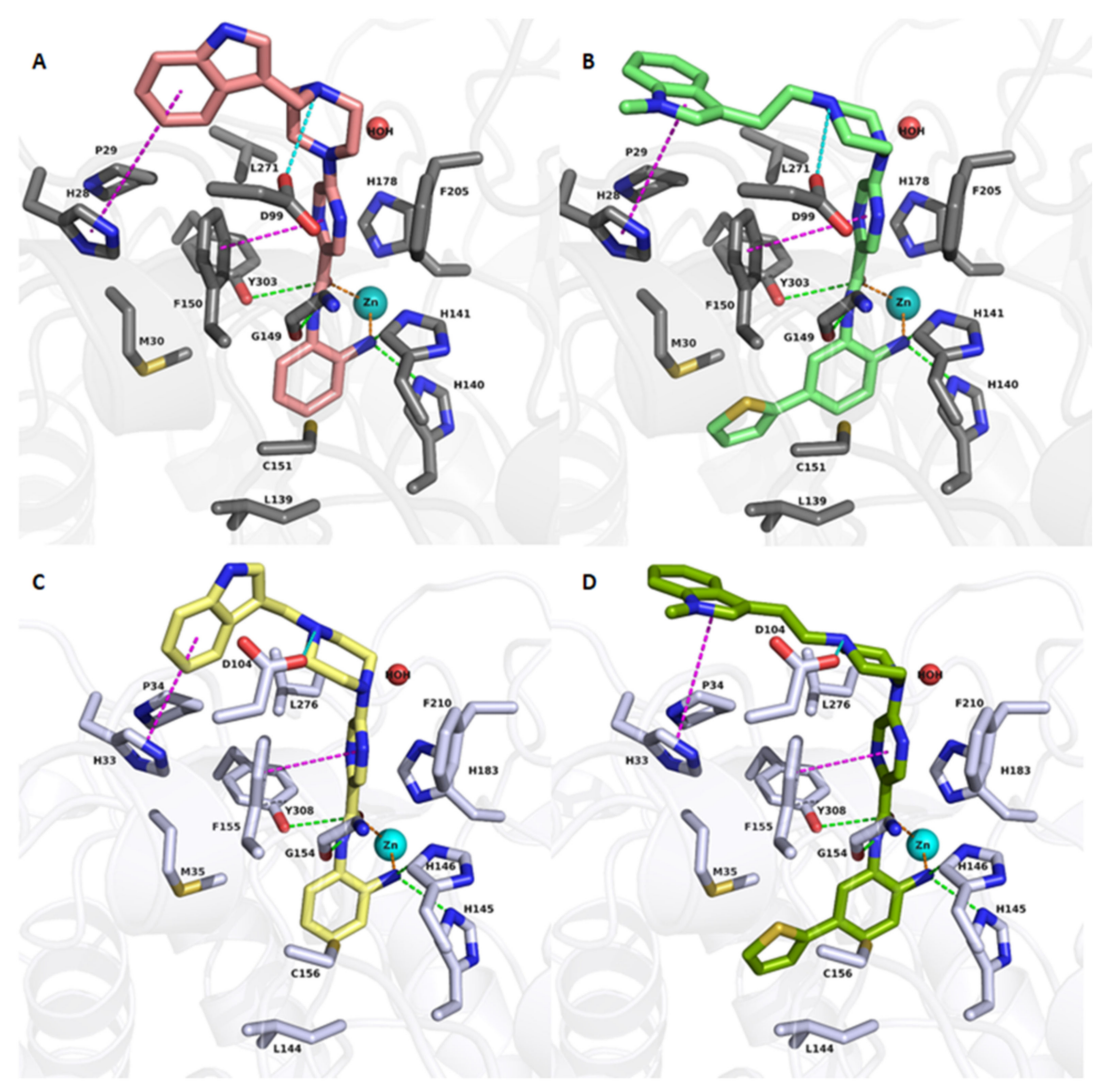
Docking poses of 19f (**A**, salmon colored sticks), 21a (**B**, light green colored sticks), in HDAC1 (PDB ID 4BKX), 19f (**C**, yellow colored sticks), 21a (**D**, dark green colored sticks), in HDAC2 (PDB ID 4LY1). Hydrogen bonds (green dashed lines), metal coordination (orange dashed lines), ionic interactions (cyan dashed lines) and aromatic interactions (magenta dashed lines) between inhibitors and the protein are shown. Relevant residues are shown in stick representation with dark grey carbon atoms in HDAC1and white carbon atoms in HDAC2. The zinc ion is shown as a cyan colored sphere. The conserved water molecule is shown as a red sphere. The zinc-carbonyl oxygen and zinc-amino distances, respectively, are: 2.54 Å and 2.45 Å for 19f/HDAC1, 2.51 Å and 2.44 Å for 21a/HDAC1, 2.17 and 2.27 Å for 19f/HDAC2, 2.22 Å and 2.29 Å for 21a/HDAC2.

The first group of the designed compounds (19a–19g), bearing different capping groups and an unsubstituted 2-aminobenzamide moiety, showed similar binding modes in HDAC1-3, as exemplified by the obtained docking results of compound of 19f in HDAC1/HDAC2 (Figure 3A,C). As observed in the resolved crystal structures of HDAC2 in a complex with 2-aminobenzamide derivatives (e.g., PDB 4LYI for HDAC2), the novel derivatives were able to chelate the catalytic zinc ion in a similar bidentate fashion through their carbonyl oxygen and the free amino group of the 2-aminobenzamide moiety (a cut off distance of 2.7 Å was determined for bidentate chelation in this study). In addition, the ZBG showed hydrogen bonds with the conserved H140/145/134, H141/146/135, G149/154/143, and Y303/308/298 in HDAC1, HDAC2, and HDAC3, respectively (Figure 3). The central pyrazine group of compound 19f was placed in the acetyl-lysine tunnel, consisting of G149/154/143, F150/155/144, H178/183/172, F205/210/200, L271/276/266 in HDAC1, HDAC2, and HDAC3, respectively. The attached basic piperazine group shows a salt bridge with the conserved aspartate residue located at the rim of the binding pocket (D99 in HDAC1, D104 in HDAC2, D93 in HDAC3). Meanwhile, the aromatic capping group was found to undergo aromatic interactions with the conserved H28 in HDAC1, H33 in HDAC2, and H22 in HDAC3, respectively.

The second group of compounds, bearing different substituents at the 2-aminobenzamide scaffold in the presence of the 3-indolyl or (N-methyl)-3-indolyl capping group, shows a different binding mode in the class I HDAC subtypes. As previously observed, substitution of the 2-aminobenzamide moiety by a 5-thienyl ring leads to a selectivity for HDAC1/2 over HDAC3 [44]. Docking results of 21a in HDAC1 and 2 (Figure 3) show that the thienyl moiety is embedded in the foot pocket of HDAC2, where it undergoes hydrophobic interactions with M35, L144, C156 in HDAC2. Meanwhile, HDAC3 has a narrower foot pocket created by pushing the L133 by Y107 in HDAC3 (replaced by S113/118 in HDAC1/2) [45], which does not allow the accommodation of bulky substituents (e.g., thiophene rings) as also substantiated by the docking results in HDAC3 (Supplementary Figure S2).

Compounds bearing no or small substituents, like fluoro substituents, at the 4- and 5- position of the benzamide moiety (19l, 19m, 19i and 19h) regain the inhibitory activity on HDAC3. Docking results show a similar binding mode in class I HDAC subtypes, where the 2-aminobenzamide moiety is placed in the foot pocket while showing a bidentate chelation of the zinc ion, as exemplified by compound 19l in Supplementary Figure S3.

**Figure 4:**
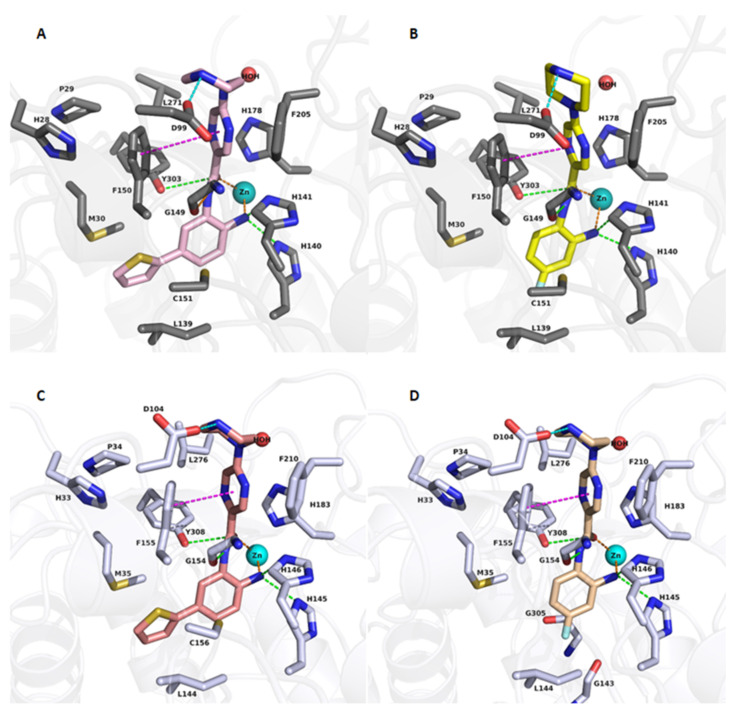
Docking poses of 29b (**A**, pink colored sticks), 23a (**B**, yellow colored sticks), in HDAC1 (PDB ID 4BKX) and 29b (**C**, salmon colored sticks), 23a (**D**, creme colored sticks) in HDAC2, (PDB ID 4LY1). Hydrogen bonds (green dashed lines), metal coordination (orange dashed lines), ionic interactions (cyan dashed lines) and aromatic interactions (magenta dashed lines) between inhibitors and the protein are shown. Relevant residues are shown in stick representation with dark grey carbon atoms in HDAC1 and white carbon atoms in HDAC2. The zinc ion is shown as a cyan colored sphere. The conserved water molecule is shown as a red sphere. The zinc-carbonyl oxygen and zinc-amino distances, respectively, are 2.43 Å and 2.41 Å for 29b/HDAC1, 2.39 Å and 2.58 Å for 23a/HDAC1, 2.22 and 2.28 Å for 29b/HDAC2, 2.23 Å and 2.41 Å for 23a/HDAC2.

A similar finding was observed with the last group of designed compounds containing different substitution on ZBG and no capping group. Aromatic substituents, like a thienyl or fluorophenyl moieties, at position-5 could not fit into the foot pocket of HDAC3 while showing a similar binding mode in HDAC1/HDAC2 (Figure 4). Hence, compounds having aromatic substituents on ZBG show good HDAC1/HDAC2 selectivity over HDAC3 as observed, for example, in compounds 21a and 29b (Figure 3B,D and Figure 4A,C).

While a small fluoro substituent at position 4 is well tolerated in the foot pocket as observed in compound 23a (Figure 4), larger or bulkier groups, like chloro (25a), and methyl (27b) methoxy (27c), and trifluoromethyl groups (27a) resulted in a significant loss in the inhibitory activity toward the HDAC1/-2/-3 subtypes. Docking poses of these derivatives, as exemplified by compound 25a in HDAC2 (Supplementary Figure S4), reveal that these substituents do not fit well in the foot pocket and result in clashes with surrounding residues (G143 and G305).

In addition to the docking study, we performed molecular dynamics (MD) simulations for the most promising inhibitors 19f, 21a, 23a, 29b and HDAC1, 2 and 3 crystal structures using Amber18 (University of California, San Francisco) to investigate the stability of the predicted binding modes. In all cases the bidentate zinc-chelation of the compounds was preserved during the MD for the studied four potent inhibitors.

The analysis of the MD simulations for the capless compounds 23a and 29b indicated a stable binding mode in HDAC1/HDAC2 and HDAC3 in terms of root-mean-square-deviation (RMSD) (Supplementary Figures S5 and S6) and the interaction of the basic piperazine and the conserved aspartate residue (D106 in HDAC1, D104 in HDAC2, D93 in HDAC3). The obtained docking poses for 23a and 29b (Figure 4) were preserved during the MD in all three HDAC subtypes. In the case of compounds 19f and 21a the solvent-exposed capping groups can adopt several energetically favorable conformations interacting with different parts of the rim region (Supplementary Figures S7 and S8). This is in accordance with the observed X-ray structures of cocrystallized flexible HDAC inhibitors (such as SAHA) where the capping group was found to bind to different regions of the rim. Hence, the RMSD of the 19f and 21a showed higher deviation throughout the MD simulation (Supplementary Figures S7 and S8). The observed flexibility of the capping group might also explain the similar inhibitory activities of the corresponding capless inhibitors. Further in silico studies are needed to find more suitable capping groups that have higher potency and selectivity. Further opportunities for chemical optimization arise via the search for bioisosteric groups for the aminopyrazine structure.

### 2.4. Cellular Assays to Analyze Our New Inhibitors

#### 2.4.1. Tests with Non-Transformed Cells

HDACi should have low toxicity to normal mammalian cells [23]. Therefore, we tested the potential cytotoxicity of the most promising inhibitors in a human epithelial kidney cell line (HEK293) that was derived from normal tissue. The cells were incubated for 48 h with the HDACi at a concentration of 50 µM, and cell viability was determined by the Alamar Blue assay. Most of the tested inhibitors caused only relatively low cytotoxicity in the human cell system and only 50 µM of 29b and 29c produced a significant reduction of cell viability (Table 3).

#### 2.4.2. Biological Tests with Leukemic Cells

Based on the in vitro activities and the low cellular toxicity for HEK293 cells, we selected the potent HDAC1/-2/-3 inhibitor 19f as well as the HDAC1/HDAC2 selective inhibitors 29b and 21a for further biological characterization in acute myeloid leukemia (AML) cells. The inhibitors were tested on FLT3-ITD positive MV4-11 cells (2 FLT3-ITD alleles). We chose such cells because FLT3-ITD positive AML is a clinically unresolved issue [46,47,48,49,50,51].

**Figure 5:**
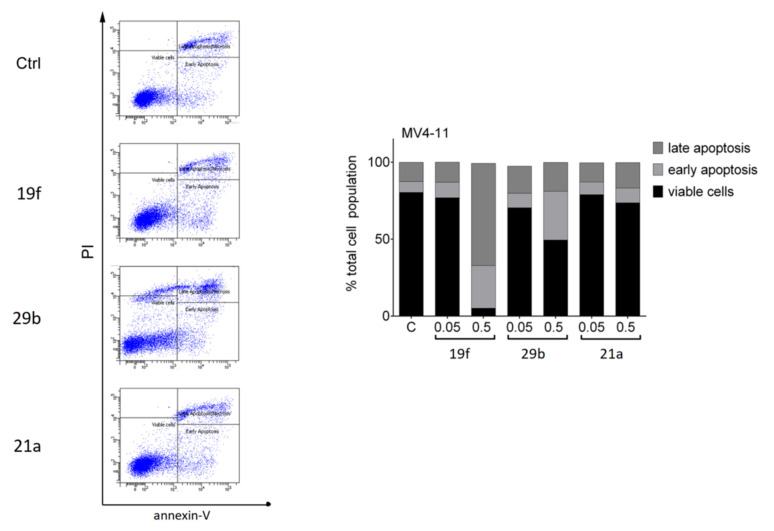
Impact of 19f, 21a and 29b on the survival of MV4-11 cells. The cells were treated with 0.5 µM of 19f, 21a, and 29b for 48 h or solvent (Ctrl). The left panel shows the original flow cytometry scans (x-axis, annexin-V-FITC; y-axis, propidium iodide, PI). The right panel shows the percentage distributions of intact, early apoptotic, and late apoptotic cells. Experiments were performed three times independently.

We measured the induction of early (increased exposure of phosphatidylserine on the cell surface and therefore positive staining for annexin-V-FITC) and late apoptosis (positivity for annexin-V and accumulation of propidium iodide, PI) by flow cytometry. Incubation of MV4-11 cells with the inhibitors showed that 0.5 µM 19f caused early and late apoptosis in nearly the whole MV4-11 cell population. We found that 0.5 µM 29b led to apoptosis in about half of the MV4-11 cell population and 21a slightly increased the number of MV4-11 cells in late apoptosis (Figure 5). Due to these data, we focused further analyses on 19f.

**Figure 6:**
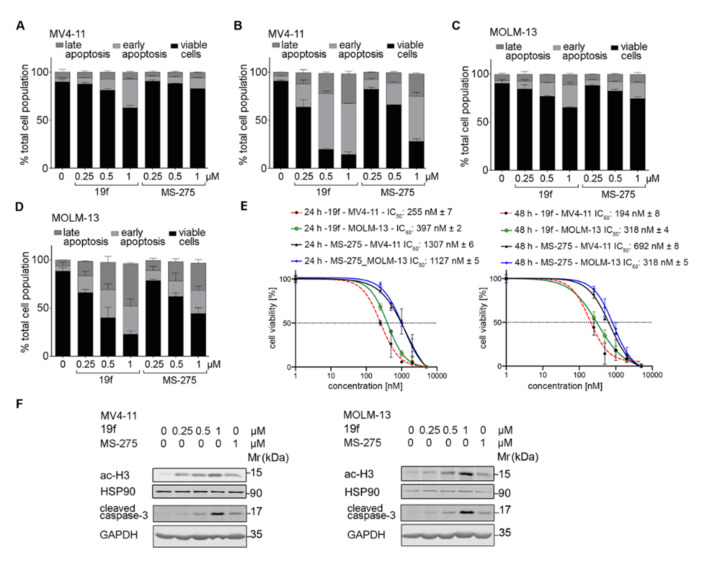
Pro-apoptotic effects of 19f and MS-275 in MV4-11 and MOLM-13 cells. A, B MV4-11 cells were treated with 0.25, 0.5, 1 µM 19f or MS-275 for 24 h (**A**) or 48 h (**B**) and analyzed for annexin-V/PI by flow cytometry. (**C**,**D**) The same experiments were conducted with MOLM-13 cells. (**E**) IC_50_ values were determined for 19f. (**F**) Immunoblot was done with the stated antibodies and lysates of MV4-11 and MOLM-13 cells that were incubated with the HDACi for 24 h. Cells were incubated with 0.25, 0.5, 1 µM 19f, or 1 µM MS-275. Graphs show representatives of three independent experiments.

**Figure 7:**
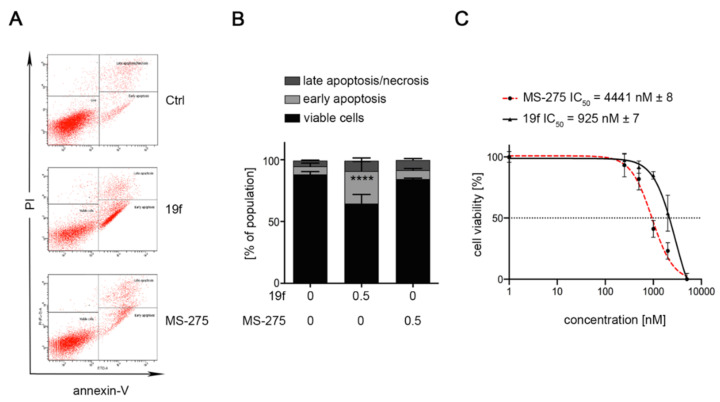
Cytotoxicity of MS-275 and 19f in leukemic HEL cells expressing JAK2V617F. (**A**,**B**) HEL cells were treated with 0.5 µM of MS-275 or 19f for 48 h. (**B**) Cells were stained with annexin V-FITC/PI and analyzed via flow cytometry. Graphs show mean ± SD of three independent experiments (one-way ANOVA; two-way ANOVA; Bonferroni correction; **** *p*  <  0.0001). (**C**) HEL cells were treated with increasing concentrations of MS-275 or 19f from 0.25 µM to 5 µM for 24 h. IC_50_ values were determined for 19f. Results represent three independent experiments.

To extend these analyses, we incubated MV4-11 cells and MOLM-13 cells (1 FLT3 wild-type allele and 1 FLT3-ITD allele) with 19f. To compare the potency of 19f with an established class I HDACi, we chose the 2-aminobenzamide MS-275. This agent, which specifically inhibits HDAC1, -2, and -3, is tested in clinical trials [51] (https://www.cancer.gov/about-cancer/treatment/clinical-trials/intervention/entinostat, accessed date 10 November 2021). We used 1 µM of 19f and 1 µM MS-275, because this is the maximal clinically achievable concentration of MS-275 in patients [52]. We found that 19f was more effective than MS-275 and those cytotoxic effects of these compounds that occurred were time- and dose-dependently (Figure 6A and Figure 7D). We found that 19f (IC_50_ for apoptosis induction 255 nM) was at least 4-fold more effective than the HDACi MS-275 (IC_50_ for apoptosis induction 1307 nM) in MV4-11 (Figure 6E). In MOLM-13 cells, 19f displayed a higher potency 4-fold (IC_50_ for apoptosis induction 397 nM) than the HDACi MS-275 (IC_50_ for apoptosis induction 1127 nM). The IC_50_ values for growth inhibition of MV4-11 and MOLM-13 cells after 48 h were around 0.3 µM (Figure 6E).

Immunoblot analyses of MV4-11 and MOLM-13 cells illustrated that 19f triggered the expected accumulation of acetylated histone H3 dose-dependently and more potently than MS-275 did. This was associated with the processing of the ultimate death executioner enzyme caspase-3 to its cleaved active form (Figure 6F).

Compound 19f was also superior to MS-275 in human erythroleukemia (HEL) cells. This erythroleukemia cell model carries a mutation in Janus kinase-2 (JAK2V617F) and HDACi are considered as therapeutic options to control the transformation of this disease into AML [53]. While 0.5 µM MS-275 did not cause apoptosis in HEL cell cultures, 0.5 µM 19f significantly induced apoptosis to 26% (Figure 7A). Dose escalation studies revealed an IC_50_ of 4441 nM for MS-275 and IC_50_ of 925 nM for 19f for toxic effects on HEL cells (Figure 7B).

#### 2.4.3. In Silico Prediction of Pharmacokinetic and Tox Data

To analyze the further in vivo potential of the most promising candidates, 19f and 21a, we calculated several physicochemical properties and predicted pharmacokinetic parameters (Table 4). For predicting the properties the PreADMET (https://preadmet.bmdrc.kr/admetox/, accessed on 10 Novermber 2021) web service was used. The in silico pharmacokinetic data (e.g., human intestinal absorption (HIA%), plasma protein binding) as well as physicochemical data (e.g., water solubility, AlogP) showed that 19f has high predicted oral bioavailability, reduced plasma protein binding and better solubility compared to the reference Entinostat (MS-275). The toxicity prediction using ProTox-II (https://tox-new.charite.de/protox_II/, accessed on 10 November 2021) showed a very low toxicity of 19f (LD_50_ 1500 mg/kg compared to 22 mg/kg for MS-275). ProTox-II uses molecular similarity, fragment propensities, the most frequent features and (fragment similarity based cross-validation) machine-learning, based on a total of 33 models for the prediction of various toxicity endpoints, such as acute toxicity, hepatotoxicity, cytotoxicity, carcinogenicity, mutagenicity, immunotoxicity, adverse outcomes pathways and toxicity targets. None of the toxicity targets included in ProTox-II were predicted for 19f.

## 3. Conclusions

A new series of 2-aminobenzamides was synthesized based on different lead structures and biologically tested for their inhibition against HDAC1, HDAC2, and HDAC3. Docking studies were carried out to guide the design of linker and capping groups, as well as the 2-aminobenzamide substitution. Of the various capping groups, indole and N-methylindole were found to be the best choices. Docking studies showed that the indole capping group, e.g., in the potent inhibitor 19f, interacts with the conserved F150/H28 in HDAC1, F150/H33 in HDAC2, and F150/H22 in HDAC3, respectively. In the case of 21a and 29b, the 2-thienyl group on the 2-aminobenzamide fits perfectly to the foot pocket in HDAC1/HDAC2, whereas in the case of HDAC3 the narrow footpocket does not allow such bulky substituents. In the case of HDAC3, the docking solutions showed that inhibitors with bulky substitutions on the 2-aminobenzamide ring could not bind to the zinc ion due to the smaller foot pocket. The most potent compounds, 19f and 29b, were subjected to a cellular biological assay against the cancer cell lines MV4-11, MOLM-13, and HEL. The inhibitors showed strong hyperacetylation of the HDAC1-3 substrate histone H3 in agreement with the in vitro HDAC inhibitory data. The best inhibitor 19f strongly induced apoptosis in these leukemic cells and was found to be superior to the clinically evaluated HDACi MS-275. This work demonstrates that we successfully synthesized and evaluated novel class-I HDACi. Of these, 19f turned out as a specific inhibitor of HDAC1, HDAC2, and HDAC3 with superior activity and promising physicochemical properties.

## 4. Materials and Methods

### 4.1. Chemistry

#### 4.1.1. General

All of the specifications regarding the standard materials, equipment and devices used in the experimental methods are included in the Supplementary Materials. In addition, the experimental procedures for synthesis of intermediates are included in the Supplementary Materials.

#### 4.1.2. General Procedure for Amide Coupling

A mixture of the appropriate carboxylic acid (1.0 eq.) and HATU (1.2 eq.) was dissolved in dry DMF (5 mL) and stirred at RT for 30 min. The corresponding amine (0.9 eq.) and DIPEA (5.0 eq.) in THF (3 mL) were added and the reaction mixture was stirred for 18 h at RT. The reaction mixture was diluted with EtOAc (15 mL) and the reaction mixture was washed with 1 N NH_4_Cl and saturated NaHCO_3_, respectively. The organic extracts were washed with brine, dried over anhydrous Na_2_SO_4_, filtered and concentrated under vacuum. The residue was purified by using MPLC (CHCl_3_:MeOH) to provide the corresponding amide. Reaction yields, and chromatographic and spectrometric data of the final compounds are reported below.

#### 4.1.3. General Procedure for the Preparation of Final Target Compounds X through N-Boc Cleavage

The N-Boc-protected aniline derivative (1 mmol) was solubilized at 0 ^0^C in dry DCM (5 mL), and then TFA (5 mL) was added. The reaction mixture was stirred at RT for 30 min. The solvent was evaporated to dryness, the residue was dissolved in 1 mL MeOH, 1 N NaOH (10 mL) was added, and the mixture was stirred for 1 h, before being extracted with EtOAc. The organic extracts were washed with brine, dried over anhydrous Na_2_SO_4_, filtered and concentrated under vacuum. The residue was purified by using MPLC (CHCl_3_:MeOH) to provide the corresponding amide. Reaction yields, chromatographic and spectrometric data of the final compounds are reported below.

#### 4.1.4. Spectral Analysis of Final Compounds

N-(2-Aminophenyl)-5-(4-phenethylpiperazin-1-yl)pyrazine-2-carboxamide (19a).

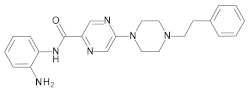



^1^ H NMR (400 MHz, DMSO-d_6_) δ 9.58 (s, 1H, -CO-NH-Ar), 8.70 (d, J = 1.3 Hz, 1H, Ar-H of Pyrazine), 8.34 (d, J = 1.4 Hz, 1H, Ar-H of Pyrazine), 7.46 (dd, J = 7.9, 1.5 Hz, 1H, Ar-H), 7.30–7.21 (m, 4H, Ar-H), 7.20–7.14 (m, 1H, Ar-H), 6.92 (td, J = 7.6, 1.6 Hz, 1H, Ar-H), 6.80 (dd, J = 8.0, 1.5 Hz, 1H, Ar-H), 6.62 (td, J = 7.5, 1.5 Hz, 1H, Ar-H), 4.82 (s, 2H, -NH_2_), 3.72 (t, J = 5.0 Hz, 4H, Piperazine Hs), 2.77 (dd, J = 9.5, 6.1 Hz, 2H, -N-CH_2_CH_2_-Ar), 2.57–2.54 (m, 6H, -N-CH_2_CH_2_-Ar + Piperazine Hs). ^13^C NMR (101 MHz, DMSO) δ 162.32, 155.54, 142.41, 141.98, 140.76, 133.14, 129.19, 129.09, 128.68, 126.30, 125.99, 124.79, 117.50, 117.23, 59.98, 52.65, 44.41, 33.10. HRMS m/z: 403.2239 [M + H]^+^; calculated C_23_H_27_N_6_O^+^: 403.2246. HPLC: rt 5.68 min (purity 97.6%), yield: 73%.

N-(2-Aminophenyl)-5-(4-(4-chlorophenethyl)piperazin-1-yl)pyrazine-2-carboxamide (19b).

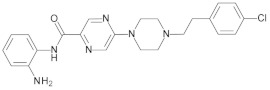



^1^ H NMR (400 MHz, DMSO-d_6_) δ 9.59 (s, 1H, -CO-NH-Ar), 8.70 (d, J = 1.3 Hz, 1H, Ar-H of Pyrazine), 8.35 (d, J = 1.4 Hz, 1H, Ar-H of Pyrazine), 7.46 (dd, J = 7.9, 1.5 Hz, 1H, Ar-H), 7.35–7.25 (m, 4H, Ar-H), 6.92 (td, J = 7.6, 1.6 Hz, 1H, Ar-H), 6.80 (dd, J = 7.9, 1.5 Hz, 1H, Ar-H), 6.63 (td, J = 7.6, 1.5 Hz, 1H, Ar-H), 4.83 (s, 2H, -NH_2_), 3.71 (t, J = 5.0 Hz, 4H, Piperazine Hs), 2.77 (t, J = 7.6 Hz, 2H, -N-CH_2_CH_2_-Ar), 2.56 (q, J = 6.8, 4.8 Hz, 6H, -N-CH_2_CH_2_-Ar + Piperazine Hs). ^13^C NMR (101 MHz, DMSO) δ 162.32, 155.53, 142.41, 141.98, 139.86, 133.15, 130.99, 130.92, 129.19, 128.56, 125.99, 124.79, 117.51, 117.23, 59.55, 52.60, 44.39, 32.25. MS m/z: 435.26 [M − H]^−^, 437.61 [M + H]^+^, HRMS m/z: 459.1680 [M+Na]^+^; calculated C_24_H_25_N_7_O^+^: 459.1676. HPLC: rt 6.57 min (purity 97%), yield: 70%.

N-(2-aminophenyl)-2-(4-(pyridin-2-yl-methyl)piperazin-1-yl)pyrimidine-5-carboxamide (19c).

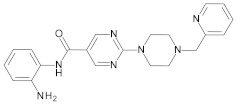



^1^ H NMR (400 MHz, DMSO-d_6_) δ 9.48 (s, 1H, -CO-NH-Ar), 8.89 (s, 2H, Ar-H of Pyrimidine), 8.58–8.51 (m, 1H, Ar-H), 7.81 (td, J = 7.7, 1.8 Hz, 1H, Ar-H), 7.49 (dt, J = 7.8, 1.1 Hz, 1H, Ar-H), 7.32 (t, J = 6.3 Hz, 1H, Ar-H), 7.12 (dd, J = 7.8, 1.5 Hz, 1H, Ar-H), 6.95 (ddd, J = 8.0, 7.2, 1.6 Hz, 1H, Ar-H), 6.75 (dd, J = 8.0, 1.4 Hz, 1H, Ar-H), 6.56 (td, J = 7.5, 1.4 Hz, 1H, Ar-H), 5.05 (s, 2H, -NH_2_), 4.00–3.76 (m, 6H, N-CH_2_-pyridine + Piperazine Hs), 2.78–2.61 (m, 4H, Piperazine Hs). ^13^C NMR (101 MHz, DMSO) δ 161.81, 158.53, 149.30, 143.75, 137.02, 127.31, 126.98, 123.32, 123.20, 122.71, 116.80, 116.48, 116.30, 64.01, 52.94, 43.93.MS m/z: 388.40 [M − H]^−^, 390.20 [M + H]^+^, HRMS m/z: 390.2034 [M + H]^+^; calculated C_21_H_24_N_7_O^+^: 390.2042. HPLC: rt 5.78 min (purity 99.8%), yield: 73%.

N-(2-Aminophenyl)-5-(4-(pyridin-2-ylmethyl)piperazin-1-yl)pyrazine-2-carboxamide (19d).

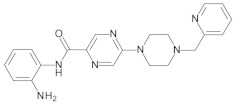



^1^ H NMR (400 MHz, DMSO-d_6_) δ 9.58 (s, 1H, -CO-NH-Ar), 8.69 (d, J = 1.3 Hz, 1H, Ar-H of Pyrazine), 8.49 (ddd, J = 4.9, 1.8, 0.9 Hz, 1H, Ar-H), 8.33 (d, J = 1.4 Hz, 1H, Ar-H of Pyrazine), 7.77 (td, J = 7.7, 1.9 Hz, 1H, Ar-H), 7.51–7.42 (m, 2H, Ar-H), 7.26 (ddd, J = 7.5, 4.8, 1.2 Hz, 1H, Ar-H), 6.96–6.87 (m, 1H, Ar-H), 6.79 (dd, J = 8.0, 1.5 Hz, 1H, Ar-H), 6.62 (td, J = 7.6, 1.5 Hz, 1H, Ar-H), 4.83 (s, 2H, -NH_2_), 3.76–3.73 (m, 4H, Piperazine Hs), 3.66 (s, 2H, N-CH_2_-pyridine), 2.57–2.54 (m, 4H, Piperazine Hs). ^13^C NMR (101 MHz, DMSO) δ 162.31, 158.44, 155.51, 149.30, 142.39, 142.00, 136.99, 133.16, 129.17, 126.00, 124.80, 124.78, 123.32, 122.69, 117.50, 117.22, 64.01, 52.72, 44.42. MS m/z: 440.38 [M − H]^−^, 388.84 [M + H]^+^, HRMS m/z: 390.2037 [M + H]^+^; calculated C_21_H_24_N_7_O^+^: 390.2042. HPLC: rt 11.33 min (purity 95%), yield: 74%.

N-(2-Aminophenyl)-5-(4-(benzo[b]thiophen-3-ylmethyl)piperazin-1-yl)pyrazine-2-carboxamide (19e).

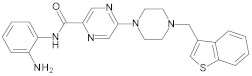



^1^ H NMR (400 MHz, DMSO-d_6_) δ 9.58 (s, 1H, -CO-NH-Ar), 8.69 (d, J = 1.3 Hz, 1H, Ar-H of Pyrazine), 8.33 (d, J = 1.4 Hz, 1H, Ar-H of Pyrazine), 8.09–7.84 (m, 2H, Ar-H), 7.63 (s, 1H, Ar-H), 7.53–7.29 (m, 3H, Ar-H), 6.98–6.87 (m, 1H, Ar-H), 6.80 (dd, J = 8.0, 1.5 Hz, 1H, Ar-H), 6.62 (td, J = 7.6, 1.5 Hz, 1H, Ar-H), 4.84 (s, 2H, -NH_2_), 3.90–3.61 (m, 6H, N-CH_2_-benzothiophene + Piperazine Hs), 2.57–2.55 (m, 4H, Piperazine Hs). MS m/z: 443.41 [M − H]^−^, 445.02 [M + H]^+^, HRMS m/z: 445.1810 [M + H]^+^; calculated C_24_H_25_N_6_OS^+^: 445.1810. HPLC: rt 7.19 min (purity 97.4%), yield: 75%.

5-(4-((1H-indol-3-yl)methyl)piperazin-1-yl)-N-(2-aminophenyl)pyrazine-2-carboxamide (19f).

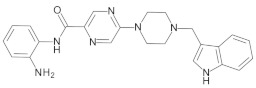



^1^ H NMR (400 MHz, DMSO-d_6_) δ 10.93 (s, 1H,-NH indole), 9.56 (s, 1H, -CO-NH-Ar), 8.67 (d, J = 1.3 Hz, 1H, Ar-H of Pyrazine), 8.31 (d, J = 1.4 Hz, 1H, Ar-H of Pyrazine), 7.65 (d, J = 7.9 Hz, 1H, Ar-H), 7.45 (d, J = 7.9, 1H, Ar-H), 7.34 (d, J = 8.1 Hz, 1H, Ar-H), 7.24 (d, J = 2.3 Hz, 1H, Ar-H), 7.10–7.02 (m, 1H, Ar-H), 7.02–6.93 (m, 1H, Ar-H), 6.91 (td, J = 7.9, 1.5 Hz, 1H, Ar-H), 6.79 (dd, J = 7.9, 1.4 Hz, 1H, Ar-H), 6.62 (td, J = 7.6, 1.5 Hz, 1H, Ar-H), 4.81 (s, 2H, -NH_2_), 3.70–3.67 (m, 6H, -N-CH_2_-Indole + Piperazine Hs), 2.60–2.50 (m, 4H, Piperazine Hs). ^13^C NMR (101 MHz, DMSO) δ 162.32, 155.48, 142.39, 141.98, 136.76, 133.07, 129.17, 128.04, 125.99, 124.79, 121.44, 119.46, 118.95, 117.50, 117.21, 111.83, 53.32, 52.38, 44.47. MS m/z: 426.41 [M − H]^−^, 428.26 [M + H]^+^, HRMS m/z: 428.2198 [M + H]^+^; calculated C_24_H_26_N_7_O^+^: 428.2198. HPLC: rt 7.89 min (97.6%), yield: 25%.

5-(4-(2-(1.H-indol-3-yl)ethyl)piperazin-1-yl)-N-(2-aminophenyl)pyrazine-2-carboxamide (19g).

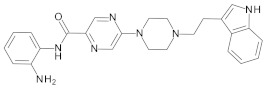



^1^ H NMR (400 MHz, DMSO-d_6_) δ 10.76 (s, 1H, -NH of indole), 9.59 (s, 1H, -CO-NH-Ar), 8.71 (d, J = 1.3 Hz, 1H, Ar-H of Pyrazine), 8.36 (d, J = 1.4 Hz, 1H, Ar-H of Pyrazine), 7.52 (dd, J = 7.8, 1.1 Hz, 1H, Ar-H), 7.46 (dd, J = 7.9, 1.5 Hz, 1H, Ar-H), 7.32 (dt, J = 8.1, 1.0 Hz, 1H, Ar-H), 7.16 (d, J = 2.4 Hz, 1H, Ar-H), 7.04 (ddd, J = 8.1, 7.0, 1.2 Hz, 1H, Ar-H), 6.99–6.88 (m, 2H, Ar-H), 6.80 (dd, J = 8.0, 1.5 Hz, 1H, Ar-H), 6.62 (td, J = 7.5, 1.5 Hz, 1H, Ar-H), 4.83 (s, 2H, -NH_2_), 3.75 (s, 4H, Piperazine Hs), 2.95–2.81 (m, 2H, -N-CH_2_CH_2_-indole), 2.66-2.60 (m, 6H, -N-CH_2_CH_2_-indole + Piperazine Hs). ^13^C NMR (101 MHz, DMSO) δ 162.33, 155.56, 142.43, 141.99, 136.63, 133.12, 129.19, 127.67, 125.99, 124.80, 122.95, 121.27, 118.72, 118.58, 117.51, 117.23, 112.89, 111.77, 59.14, 52.75, 44.48, 22.85. MS m/z: 440.38 [M − H]^−^, 442.19 [M + H]^+^, HRMS m/z: 464.2173 [M+Na]^+^; calculated C_25_H_27_N_7_ONa^+^: 464.2174. HPLC: rt 6.54 min (purity 98.3%), yield: 76%.

5-(4-(2-(1H-indol-2-yl)ethyl)piperazin-1-yl)-N-(2-amino-4,5-difluorophenyl)pyrazine-2-carboxamide (19h).

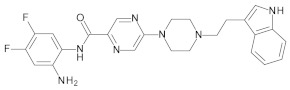



^1^ H NMR (400 MHz, DMSO-d_6_) δ 10.76 (s, 1H, NH of indole), 9.64 (s, 1H, CO-NH-Ar), 8.70 (d, J = 1.3 Hz, 1H, Ar-H of Pyrazine), 8.34 (d, J = 1.2 Hz, 1H, Ar-H of Pyrazine), 7.63–7.47 (m, 2H, Ar-H), 7.32 (d, J = 8.0 Hz, 1H, Ar-H), 7.16 (s, 1H, Ar-H), 7.12–6.86 (m, 2H, Ar-H), 6.77 (dd, J = 12.8, 8.2 Hz, 1H, Ar-H), 5.02 (s, 2H, NH_2_), 3.82–3.69 (m, 4H, Piperazine Hs), 2.91–2.87 (m, 2H, -N-CH_2_CH_2_-Ar), 2.69–2.62 (m, 6H, -N-CH_2_CH_2_-Ar + Piperazine Hs). ^13^C NMR (101 MHz, DMSO) δ 162.56, 155.59, 142.62, 136.63, 132.65, 129.20, 127.67, 122.95, 121.27, 118.72, 118.58, 113.34, 113.14, 112.89, 111.77, 104.61, 104.41, 59.22, 52.75, 44.47, 22.91. MS m/z: 478.43 [M + H]. HRMS m/z: 478.2163 [M + H]^+^; calculated C_25_H_26_F_2_N_7_O^+^: 478.2166. HPLC: rt 7.18 min (purity 98.3%), yield: 32%.

5-(4-(2-(1H-indol-3-yl)ethyl)piperazin-1-yl)-N-(2-amino-4-chlorophenyl)pyrazine-2-carboxamide (19i).

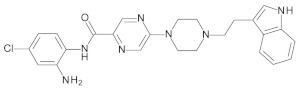



^1^ H NMR (400 MHz, DMSO-d_6_) δ 10.76 (s, 1H, -NH of indole), 9.59 (s, 1H, -CO-NH-Ar), 8.69 (d, J = 1.3 Hz, 1H, Ar-H of Pyrazine), 8.35 (d, J = 1.3 Hz, 1H, Ar-H of Pyrazine), 7.52 (d, J = 7.8 Hz, 1H, Ar-H), 7.41 (d, J = 8.5 Hz, 1H, Ar-H), 7.32 (d, J = 8.1 Hz, 1H, Ar-H), 7.16 (s, 1H, Ar-H), 7.00 (dt, J = 34.2, 7.5 Hz, 2H, Ar-H), 6.83 (s, 1H, Ar-H), 6.66–6.57 (m, 1H, Ar-H), 5.16 (s, 2H, -NH_2_), 3.84–3.67 (m, 4H, Piperazine Hs), 2.97–2.81 (m, 2H, -N-CH_2_CH_2_-Ar), 2.72–2.52 (m, 6H, -N-CH_2_CH_2_-Ar + Piperazine Hs). ^13^C NMR (101 MHz, DMSO) δ 162.60, 155.56, 144.03, 142.54, 136.63, 132.97, 129.98, 129.17, 127.67, 126.63, 123.32, 122.96, 121.27, 118.72, 118.58, 116.55, 115.90, 112.87, 111.77, 59.11, 52.73, 44.45, 22.83. MS m/z: 476.26 [M + H]^+^. HRMS m/z: 498.1782 [M+Na]^+^; calculated C_25_H_26_ClN_7_ONa^+^: 498.1785. HPLC: rt 11.32 min (purity 96.9%), yield: 32%.

N-(2-aminophenyl)-2-(4-((1-methyl-1H-indol-3-yl)methyl)piperazin-1-yl)pyrimidine-5-carboxamide (19j).

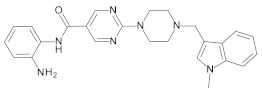



^1^ H NMR (400 MHz, DMSO-d_6_) δ 9.44 (s, 1H, -CO-NH-Ar), 8.85 (s, 2H, Ar-H of Pyrimidine), 7.66 (dt, J = 7.9, 1.0 Hz, 1H, Ar-H), 7.39–7.35 (m, 1H, Ar-H), 7.23 (s, 1H, Ar-H), 7.15–7.09 (m, 2H, Ar-H), 7.02 (ddd, J = 8.0, 7.0, 1.0 Hz, 1H, Ar-H), 6.94 (td, J = 7.6, 1.6 Hz, 1H, Ar-H), 6.74 (dd, J = 8.0, 1.5 Hz, 1H, Ar-H), 6.55 (td, J = 7.5, 1.5 Hz, 1H, Ar-H), 4.90 (s, 2H, -NH_2_), 3.82 (t, J = 5.3 Hz, 4H, Piperazine Hs), 3.74 (s, 3H, N-CH_3_), 3.66 (s, 2H, N-CH_2_-Indole), 2.45 (t, J = 5.3 Hz, 4H, Piperazine Hs). MS m/z: 440.42 [M − H]^−^. HRMS m/z: 442.2349 [M + H]^+^; calculated C_25_H_28_N_7_O^+^: 442.2355. HPLC: rt 7.96 min (purity 97.7%), yield: 60%.

N-(2-aminophenyl)-5-(4-((1-methyl-1H-indol-3-yl)methyl)piperazin-1-yl)pyrazine-2-carboxamide (19k).

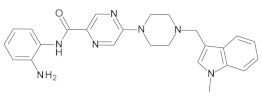



^1^ H NMR (400 MHz, DMSO-d_6_) δ 9.56 (s, 1H, -CO-NH-Ar), 8.67 (d, J = 1.3 Hz, 1H, Ar-H of Pyrazine), 8.31 (d, J = 1.4 Hz, 1H, Ar-H of Pyrazine), 7.66 (d, J = 7.9 Hz, 1H, Ar-H), 7.45 (dd, J = 7.9, 1.5 Hz, 1H, Ar-H), 7.38 (d, J = 8.2 Hz, 1H, Ar-H), 7.24 (s, 1H, Ar-H), 7.13 (ddd, J = 8.2, 7.0, 1.2 Hz, 1H, Ar-H), 7.02 (ddd, J = 7.9, 7.0, 1.0 Hz, 1H, Ar-H), 6.95–6.86 (m, 1H, Ar-H), 6.79 (dd, J = 8.0, 1.5 Hz, 1H, Ar-H), 6.62 (td, J = 7.6, 1.5 Hz, 1H, Ar-H), 4.81 (s, 2H, -NH_2_), 3.74 (s, 3H, -NCH_3_), 3.71–3.68 (m, 6H, N-CH_2_-Indole + Piperazine Hs), 2.51–2.48 (m, 4H, Piperazine Hs). ^13^C NMR (101 MHz, DMSO) δ 162.31, 155.49, 142.40, 141.97, 137.18, 133.04, 129.53, 129.15, 128.38, 125.98, 124.79, 124.77, 121.54, 119.67, 119.04, 117.50, 117.22, 110.02, 53.21, 52.42, 44.51, 32.74. MS m/z: 440.38 [M − H]^−^, 442.04 [M + H]^+^, HRMS m/z: 442.2350 [M + H]^+^; calculated C_25_H_28_N_7_O^+^: 442.2355, HPLC: rt 14.22 min (purity 95.4%), yield: 56%.

N-(2-amino-4,5-difluorophenyl)-2-(4-((1-methyl-1H-indol-3-yl)methyl)piperazin-1-yl)pyrimidine-5-carboxamide (19l).

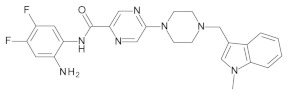



^1^ H NMR (400 MHz, DMSO-d_6_) δ 9.60 (s, 1H, CO-NH-Ar), 8.66 (d, J = 1.3 Hz, 1H, Ar-H of Pyrazine), 8.30 (d, J = 1.3 Hz, 1H, Ar-H of Pyrazine), 7.66 (d, J = 7.8 Hz, 1H, Ar-H), 7.54 (dd, J = 12.4, 8.7 Hz, 1H, Ar-H), 7.37 (d, J = 8.2 Hz, 1H, Ar-H), 7.23 (s, 1H, Ar-H), 7.17–7.08 (m, 1H, Ar-H), 7.06–6.96 (m, 1H, Ar-H), 6.76 (dd, J = 12.8, 8.2 Hz, 1H, Ar-H), 5.01 (s, 2H, NH_2_), 3.77–3.59 (m, 9H, N-CH_2_-Indole +-N-CH_3_ + Piperazine Hs), 2.58–2.45 (m, 4H, Piperazine Hs). 13C NMR (101 MHz, DMSO) δ 162.54, 155.52, 142.59, 139.40, 137.18, 132.56, 129.52, 129.17, 128.38, 121.53, 120.61, 120.56, 119.67, 119.04, 113.31, 113.11, 110.02, 104.60, 104.40, 53.20, 52.42, 44.51, 32.74. MS m/z: 476.30 [M − H]. HRMS m/z: 500.1979 [M+Na]^+^; calculated C_25_H_25_F_2_N_7_ONa^+^: 500.1986. HPLC: rt 7.94 min (purity 98.8%), yield: 30%.

N-(2-Amino-4-fluorophenyl)-5-(4-(2-(1-methyl-1H-indol-3-yl)ethyl)piperazin-1-yl)pyrazine-2-carboxamide (19m).

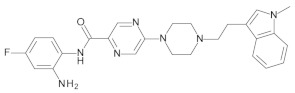



^1^ H NMR (400 MHz, DMSO-d_6_) δ 9.53 (s, 1H, -CO-NH-Ar), 8.69 (d, J = 1.3 Hz, 1H, Ar-H of Pyrazine), 8.35 (d, J = 1.4 Hz, 1H, Ar-H of Pyrazine), 7.53 (dt, J = 7.9, 1.0 Hz, 1H, Ar-H), 7.36 (d, J = 8.2 Hz, 1H, Ar-H), 7.30 (dd, J = 8.7, 6.3 Hz, 1H, Ar-H), 7.16–7.08 (m, 2H, Ar-H), 7.00 (ddd, J = 7.9, 6.9, 1.0 Hz, 1H, Ar-H), 6.56 (dd, J = 11.1, 2.9 Hz, 1H, Ar-H), 6.38 (td, J = 8.6, 2.9 Hz, 1H, Ar-H), 5.16 (s, 2H, -NH_2_), 3.79–3.73 (m, 4H, Piperazine Hs), 2.88 (dd, J = 9.8, 5.8 Hz, 2H, -N-CH_2_CH_2_-Ar), 2.70–2.55 (m, 6H, -N-CH_2_CH_2_-Ar + Piperazine Hs). ^13^C NMR (101 MHz, DMSO) δ 162.73, 159.76, 155.55, 144.96, 144.84, 142.46, 137.02, 133.11, 129.15, 127.99, 127.42, 127.37, 127.26, 121.42, 120.30, 118.97, 118.68, 112.28, 109.94, 103.06, 102.84, 102.63, 102.39, 59.16, 52.75, 44.47, 32.66, 22.67. HRMS m/z: 474.2420 [M + H]^+^; calculated C_26_H_29_N_7_FO^+^: 474.2417. HPLC: rt 8.05 min (purity 93.7%), yield: 66%.

N-(2-aminophenyl)-2-(4-methylpiperazin-1-yl)pyrimidine-5-carboxamide (19n).

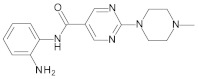



^1^ H NMR (400 MHz, DMSO-d_6_) δ 9.47 (s, 1H, -CO-NH-Ar), 8.87 (s, 2H, Ar-H of Pyrimidne), 7.15–7.08 (m, 1H, Ar-H), 6.99–6.90 (m, 1H, Ar-H), 6.74 (dd, J = 8.0, 1.4 Hz, 1H, Ar-H), 6.56 (td, J = 7.6, 1.4 Hz, 1H, Ar-H), 4.91 (s, 2H, -NH_2_), 3.88–3.78 (m, 4H, Piperazine Hs), 2.43–2.33 (m, 4H, Piperazine Hs), 2.22 (s, 3H, -N-CH_3_). ^13^C NMR (101 MHz, DMSO) δ 163.06, 161.84, 158.55, 143.74, 127.31, 126.96, 123.23, 116.81, 116.48, 116.32, 54.66, 46.00, 43.72. MS m/z: 313.36 [M + H]. HRMS m/z: 313.1770 [M + H]^+^; C_16_H_20_N_6_O^+^: 313.1776. HPLC: rt 5.58 min (purity 100%), yield: 55%.

N-(2-aminophenyl)-5-(4-methylpiperazin-1-yl)pyrazine-2-carboxamide (19o).

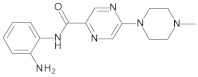



^1^ H NMR (400 MHz, DMSO-d_6_) δ 9.58 (s, 1H, -CO-NH-Ar), 8.69 (d, J = 1.3 Hz, 1H, Ar-H of Pyrazine), 8.34 (d, J = 1.4 Hz, 1H, Ar-H of Pyrazine), 7.45 (dd, J = 7.9, 1.4 Hz, 1H, Ar-H), 6.96–6.87 (m, 1H, Ar-H), 6.80 (dd, J = 8.0, 1.4 Hz, 1H, Ar-H), 6.62 (td, J = 7.7, 1.4 Hz, 1H, Ar-H), 4.82 (s, 2H, -NH_2_), 3.76–3.68 (m, 4H, Piperazine Hs), 2.45–2.37 (m, 4H, Piperazine Hs), 2.22 (s, 3H, -NCH_3_). ^13^C NMR (101 MHz, DMSO) δ 162.32, 155.54, 142.40, 142.00, 133.13, 129.19, 125.99, 124.81, 117.49, 117.22, 54.57, 46.15, 44.29. MS m/z: 313.36[M + H]. HRMS m/z: 313.1770 [M + H]^+^; C_16_H_20_N_6_O^+^: 313.1776. HPLC: rt 5.91 min (purity 99.7%), yield: 66%.

N-(2-Amino-5-(thiophen-2-yl)phenyl)-5-(4-(2-(1-methyl-1H-indol-3-yl)ethyl)piperazin-1-yl)pyrazine-2-carboxamide (21a).

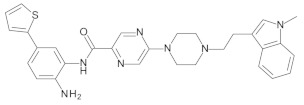



^1^ H NMR (400 MHz, DMSO-d_6_) δ 9.67 (s, 1H, -CO-NH-Ar), 8.73 (d, J = 1.3 Hz, 1H, Ar-H of Pyrazine), 8.37 (d, J = 1.4 Hz, 1H, Ar-H of Pyrazine), 7.78 (d, J = 2.2 Hz, 1H, Ar-H), 7.54 (dt, J = 7.9, 1.0 Hz, 1H, Ar-H), 7.39–7.31 (m, 2H, Ar-H), 7.25–7.22 (m, 2H, Ar-H), 7.16–7.09 (m, 2H, Ar-H), 7.04 (dd, J = 5.1, 3.6 Hz, 1H, Ar-H), 7.00 (ddd, J = 8.0, 7.0, 1.0 Hz, 1H, Ar-H), 6.83 (d, J = 8.3 Hz, 1H, Ar-H), 5.08 (s, 2H, -NH_2_), 3.77 (t, J = 5.1 Hz, 4H, Piperazine Hs), 3.72 (s, 3H, -NCH_3_), 2.93–2.83 (m, 2H, -N-CH_2_CH_2_-Ar), 2.70–2.56 (m, 6H, -N-CH_2_CH_2_-Ar + Piperazine Hs). ^13^C NMR (101 MHz, dmso) δ 162.59, 155.57, 144.77, 142.55, 142.03, 137.02, 133.02, 129.20, 128.63, 127.99, 127.42, 124.81, 123.82, 123.40, 122.27, 121.61, 121.42, 118.98, 118.68, 117.39, 112.28, 109.94, 59.16, 52.75, 44.47, 32.66, 22.67. MS m/z: 536.52 [M − H]^−^, 538.48 [M + H]^+^, HRMS m/z: 538.2386 [M + H]^+^; calculated C_30_H_32_N_7_OS ^+^: 538.2389. HPLC: rt 9.64 min (95%), yield: 65%.

5-(4-(2-(1H-indol-3-yl)ethyl)piperazin-1-yl)-N-(4-amino-4’-fluoro-[1,1’-biphenyl]-3-yl)pyrazine-2-carboxamide (21b).

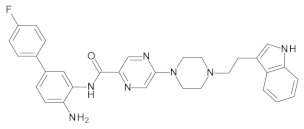



^1^ H NMR (400 MHz, DMSO-d_6_) δ 10.76 (s, 1H, -NH of indole), 9.67 (s, 1H, CO-NH-Ar), 8.72 (d, J = 1.3 Hz, 1H, Ar-H of Pyrazine), 8.37 (d, J = 1.3 Hz, 1H, Ar-H of Pyrazine), 7.77 (s, 1H, Ar-H), 7.60–7.48 (m, 3H, Ar-H), 7.32 (d, J = 8.0 Hz, 1H, Ar-H), 7.28–7.13 (m, 4H, Ar-H), 7.06–6.94 (m, 2H, Ar-H), 6.88 (d, J = 8.3 Hz, 1H, Ar-H), 5.02 (s, 2H, -NH_2_), 3.84–3.68 (m, 4H, Piperazine Hs), 2.97–2.80 (m, 2H, -N-CH_2_CH_2_-Ar), 2.67–2.59 (m, 6H, -N-CH_2_CH_2_-Ar + Piperazine Hs). ^13^C NMR (101 MHz, DMSO) δ 162.54, 160.29, 155.58, 142.52, 141.79, 137.28, 136.63, 133.05, 129.20, 128.34, 127.83, 124.93, 124.29, 123.19, 122.96, 121.27, 118.72, 118.58, 117.53, 116.04, 115.83, 112.90, 111.77, 59.14, 52.76, 44.49, 22.85.MS m/z: 536.16 [M + H]. HRMS m/z: 536.2573 [M + H]^+^; calculated C_31_H_31_FN_7_O ^+^: 536.2574. HPLC: rt 9.01 min (purity 96.3%), yield: 88%.

5-(4-(2-(1H-indol-3-yl)ethyl)piperazin-1-yl)-N-(4-amino-2’-fluoro-[1,1’-biphenyl]-3-yl)pyrazine-2-carboxamide (21c).

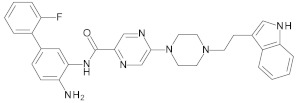



^1^ H NMR (400 MHz, DMSO-d_6_) δ 10.76 (s, 1H, -NH of indole), 9.68 (s, 1H, CO-NH-Ar), 8.72 (s, 1H, Ar-H of Pyrazine), 8.37 (s, 1H, Ar-H of Pyrazine), 7.69 (s, 1H, Ar-H), 7.53–7.15 (m, 8H, Ar-H), 7.05 (t, J = 8 Hz, 1H, Ar-H), 6.96 (t, J = 8 Hz, 1H, Ar-H), 6.89 (d, J = 8.0 Hz, 1H, Ar-H), 5.09 (s, 2H, -NH_2_), 3.77–3.73 (m, 4H, Piperazine Hs), 3.02–2.76 (m, 2H, -N-CH_2_CH_2_-Ar), 2.76–2.49 (m, 6H, -N-CH_2_CH_2_-Ar + Piperazine Hs). 13C NMR (101 MHz, DMSO) δ 162.57, 158.27, 155.57, 142.52, 142.17, 136.63, 133.06, 130.55, 130.51, 129.19, 127.67, 125.49, 125.22, 125.19, 124.45, 123.86, 122.95, 121.31, 118.72, 118.58, 116.99, 116.56, 116.33, 112.90, 111.77, 59.14, 52.76, 44.49, 22.85. MS m/z: 536.36 [M + H]. HRMS m/z: 558.2393 [M+Na]^+^; calculated C_31_H_30_FN_7_ONa^+^: 558.2393. HPLC: rt 10.75 min (purity 96.4%), yield: 85%.

N-(2-Amino-4-fluorophenyl)-5-(piperazin-1-yl)pyrazine-2-carboxamide (23a).

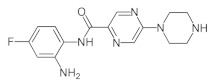



^1^ H NMR (400 MHz, DMSO-d_6_) δ 9.51 (s, 1H, -CO-NH-Ar), 8.66 (d, J = 1.3 Hz, 1H, Ar-H of Pyrazine), 8.29 (d, J = 1.4 Hz, 1H, Ar-H of Pyrazine), 7.29 (dd, J = 8.7, 6.3 Hz, 1H, Ar-H), 6.56 (dd, J = 11.1, 2.9 Hz, 1H, Ar-H), 6.37 (td, J = 8.6, 2.9 Hz, 1H, Ar-H), 5.15 (s, 2H, -NH_2_), 3.72–3.58 (m, 4H, Piperazine Hs), 3.29 (s, 1H, NH), 2.92–2.68 (m, 4H, Piperazine Hs). MS m/z: 315.46 [M − H]^−^. ^13^C NMR (101 MHz, DMSO) δ 162.75, 162.12, 159.74, 155.61, 144.93, 144.82, 142.46, 129.03, 127.33, 127.23, 120.33, 103.06, 102.84, 102.64, 102.39, 45.68, 45.53. HRMS m/z: 317.1525 [M + H]^+^; calculated C_15_H_18_FN_6_O^+^: 317.1526. HPLC: rt 3.72 min (purity 97.9%), yield: 30%.

4-(5-((2-Amino-5-fluorophenyl)carbamoyl)pyrazin-2-yl)-1-((1-methyl-1H-indol-3-yl)methyl)piperazin-1-ium chloride (23b).

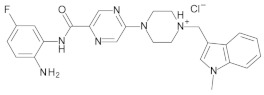



^1^ H NMR (400 MHz, DMSO-d_6_) δ 11.22 (s, 1H, Piperazinium H), 10.27 (s, 1H, -CO-NH-Ar), 8.76 (d, J = 1.3 Hz, 1H, Ar-H of Pyrazine), 8.43 (d, J = 1.5 Hz, 1H, Ar-H of Pyrazine), 7.84 (dt, J = 7.9, 1.0 Hz, 1H, Ar-H), 7.63 (s, 1H, Ar-H), 7.55 (dd, J = 10.3, 2.9 Hz, 1H, Ar-H), 7.51–7.46 (m, 1H, Ar-H), 7.39–7.32 (m, 1H, Ar-H), 7.22 (ddd, J = 8.2, 7.0, 1.2 Hz, 1H, Ar-H), 7.14 (ddd, J = 8.0, 7.0, 1.0 Hz, 1H, Ar-H), 7.08 (td, J = 8.4, 3.0 Hz, 1H, Ar-H), 4.67 (d, J = 14.2 Hz, 2H, -N-CH_2_-Indole), 4.50 (s, 2H, -NH_2_), 3.82 (s, 3H, -N-CH_3_), 3.49 (t, J = 12.3 Hz, 4H, Piperazine Hs), 3.36–2.93 (m, 4H, Piperazine Hs). HRMS m/z: 460.2260 [M + H]^+^; calculated C_25_H_27_FN_7_O^+^: 460.2261. HPLC: rt 7.47 min (purity 96.7%), yield: 56%.

N-(2-Amino-5-fluorophenyl)-5-(4-(2-(1-methyl-1H-indol-3-yl)ethyl)piperazin-1-yl)pyrazine-2-carboxamide (23c).

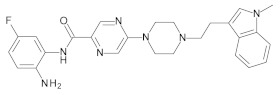



^1^ H NMR (400 MHz, DMSO-d_6_) δ 9.72 (s, 1H, -CO-NH-Ar), 8.72 (d, J = 1.3 Hz, 1H, Ar-H of Pyrazine), 8.38 (d, J = 1.4 Hz, 1H, Ar-H of Pyrazine), 7.59 (dd, J = 10.8, 3.0 Hz, 1H, Ar-H), 7.53 (dt, J = 7.9, 1.0 Hz, 1H, Ar-H), 7.38–7.32 (m, 1H, Ar-H), 7.17–7.08 (m, 2H, Ar-H), 7.00 (ddd, J = 7.9, 7.0, 1.0 Hz, 1H, Ar-H), 6.83 (dd, J = 8.8, 5.9 Hz, 1H, Ar-H), 6.76 (td, J = 8.5, 3.0 Hz, 1H, Ar-H), 4.73 (s, 2H, -NH2), 3.79–3.74 (m, 4H, Piperazine Hs), 3.72 (s, 3H, -NCH_3_), 2.88 (dd, J = 9.0, 6.6 Hz, 2H, -N-CH_2_CH_2_-Ar), 2.67–2.58 (m, 6H, -N-CH_2_CH_2_-Ar + Piperazine Hs). ^13^C NMR (101 MHz, DMSO) δ 162.26, 142.59, 129.28, 127.42, 121.42, 120.29, 118.97, 118.68, 117.04, 109.94, 59.14, 52.74, 44.45, 32.66, 22.67. MS m/z: 472.56 [M − H]^−^, 474.50 [M + H]^+^, HRMS m/z: 474.2414 [M + H]^+^; calculated C_26_H_29_FN_7_O^+^: 474.2417 HPLC: rt 7.44 min (purity 96.6%), yield: 68%.

N-(2-amino-4-chlorophenyl)-5-(piperazin-1-yl)pyrazine-2-carboxamide (25a).

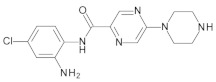



^1^ H NMR (400 MHz, DMSO-d_6_) δ 9.57 (s, 1H, -CO-NH-Ar), 8.67 (d, J = 1.3 Hz, 1H, Ar-H of Pyrazine), 8.30 (d, J = 1.3 Hz, 1H, Ar-H of Pyrazine), 7.41 (d, J = 8.5 Hz, 1H, Ar-H), 6.83 (d, J = 2.4 Hz, 1H, Ar-H), 6.62 (dd, J = 8.5, 2.4 Hz, 1H, Ar-H), 5.15 (s, 2H, -NH_2_), 3.65–3.60 (m, 4H, Piperazine Hs), 3.29 (s, 1H, Piperazine NH), 2.84–2.74 (m, 4H, Piperazine Hs). ^13^C NMR (101 MHz, DMSO) δ 162.63, 155.63, 143.99, 142.55, 129.94, 129.04, 126.58, 123.36, 118.29, 116.55, 115.90, 45.75, 45.63. MS m/z: 333.34 [M + H] ^+^. HRMS m/z: 333.1230 [M + H]^+^; calculated C_15_H_18_ClN_6_O^+^: 333.1230. HPLC: rt 4.77 min (purity 96.6%), yield: 90%.

N-(2-amino-4,5-difluorophenyl)-5-(piperazin-1-yl)pyrazine-2-carboxamide (25b).

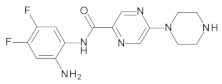



^1^ H NMR (400 MHz, DMSO-d_6_) δ 9.62 (s, 1H, CO-NH-Ar), 8.67 (d, J = 1.3 Hz, 1H, Ar-H of Pyrazine), 8.30 (d, J = 1.3 Hz, 1H, Ar-H of Pyrazine), 7.55 (dd, J = 12.4, 8.7 Hz, 1H, Ar-H), 6.77 (dd, J = 12.8, 8.2 Hz, 1H, Ar-H), 5.02 (s, 2H, NH_2_), 3.70–3.60 (m, 4H, Piperazine Hs), 3.30 (s, 1H, NH), 2.85–2.75 (m, 4H, Piperazine Hs). ^13^C NMR (101 MHz, DMSO) δ 162.58, 155.66, 142.63, 139.28, 132.36, 129.09, 120.61, 113.28, 113.08, 104.62, 104.42, 45.68, 45.53. MS m/z: 335.60 [M + H]. HRMS m/z: 335.1430 [M + H]^+^; calculated C_15_H_17_F_2_N_6_O ^+^: 335.1431. HPLC: rt 3.57 min (purity 95.6%), yield: 50%.

N-(2-amino-5-(trifluoromethyl)phenyl)-5-(piperazin-1-yl)pyrazine-2-carboxamide (27a).

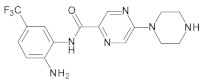



^1^ H NMR (400 MHz, DMSO-d_6_) δ 9.65 (s, 1H, CO-NH-Ar), 8.68 (d, J = 1.2 Hz, 1H, Ar-H of Pyrazine), 8.31 (d, J = 1.0 Hz, 1H, Ar-H of Pyrazine), 7.73 (d, J = 1.9 Hz, 1H, Ar-H), 7.23 (d, J = 8.4 Hz, 1H, Ar-H), 6.89 (d, J = 8.2 Hz, 1H, Ar-H), 5.57 (s, 2H, NH2), 3.72–3.57 (m, 4H, Piperazine Hs), 3.35 (s, 1H, NH), 2.86–2.71 (m, 4H, Piperazine Hs). ^13^C NMR (101 MHz, DMSO) δ 162.98, 155.64, 146.18, 142.66, 132.53, 129.05, 126.77, 124.08, 123.56, 123.20, 122.44, 116.18, 45.70, 45.57.MS m/z: 367.46 [M + H]. HRMS m/z: 367.1493 [M + H]^+^; calculated C_16_H_18_F_3_N_6_O ^+^: 367.1494. HPLC: rt 7.16 min (purity 95.7%), yield: 80%.

N-(2-amino-4-methylphenyl)-5-(piperazin-1-yl)pyrazine-2-carboxamide (27b).

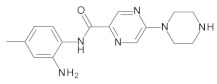



^1^ H NMR (400 MHz, DMSO-d_6_) δ 9.48 (s, 1H, CO-NH-Ar), 8.66 (d, J = 1.3 Hz, 1H, Ar-H of Pyrazine), 8.29 (d, J = 1.2 Hz, 1H, Ar-H of Pyrazine), 7.29 (d, J = 8.0 Hz, 1H, Ar-H), 6.60 (s, 1H, Ar-H), 6.43 (d, J = 8.2 Hz, 1H, Ar-H), 4.74 (s, 2H, NH_2_), 3.69–3.57 (m, 4H, Piperazine Hs), 3.22 (s, 1H, NH), 2.79–2.76 (m, 4H, Piperazine Hs), 2.16 (s, 3H, CH_3_). ^13^C NMR (101 MHz, DMSO) δ 162.32, 155.62, 142.36, 141.92, 135.04, 132.91, 129.03, 124.87, 122.24, 118.19, 117.60, 45.74, 45.61, 21.23.HRMS m/z: 313.1771 [M + H]^+^; calculated C_16_H_21_N_6_O^+^: 313.1776. HPLC: rt 3.01 min (purity 98.2%), yield: 85%.

N-(2-amino-4-methoxyphenyl)-5-(piperazin-1-yl)pyrazine-2-carboxamide (27c).

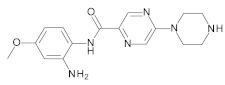



^1^ H NMR (400 MHz, DMSO-d_6_) δ 9.41 (s, 1H, CO-NH-Ar), 8.65 (d, J = 1.2 Hz, 1H, Ar-H of Pyrazine), 8.28 (d, J = 1.2 Hz, 1H, Ar-H of Pyrazine), 7.20 (d, J = 8.7 Hz, 1H, Ar-H), 6.36 (d, J = 2.8 Hz, 1H, Ar-H), 6.19 (dd, J = 8.7, 2.8 Hz, 1H, Ar-H), 4.86 (s, 2H, NH_2_), 3.68–3.55 (m, 7H, Piperazine Hs + OCH_3_), 3.32 (s, 1H, NH), 2.87–2.68 (m, 4H, Piperazine Hs). ^13^C NMR (101 MHz, DMSO) δ 162.51, 158.10, 155.59, 143.98, 142.31, 133.02, 129.01, 126.67, 117.62, 102.75, 101.90, 55.31, 45.70, 45.56. MS m/z: 329.38 [M + H]^+^. HRMS m/z: 329.1725 [M + H]^+^; calculated C_16_H_21_N_6_O_2_^+^: 329.1725. HPLC: rt 3.14 min (purity 100%), yield: 82%.

N-(2-amino-5-(thiophen-3-yl)phenyl)-5-(4-methylpiperazin-1-yl)pyrazine-2-carboxamide (29a).

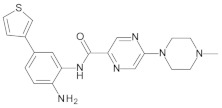



^1^ H NMR (400 MHz, DMSO-d_6_) δ 9.65 (s, 1H, CO-NH-Ar), 8.71 (d, J = 1.3 Hz, 1H, Ar-H of Pyrazine), 8.35 (d, J = 1.3 Hz, 1H, Ar-H of Pyrazine), 7.77 (d, J = 2.1 Hz, 1H, Ar-H), 7.57–7.51 (m, 2H, Ar-H), 7.39 (dd, J = 4.2, 2.3 Hz, 1H, Ar-H), 7.30 (dd, J = 8.3, 2.1 Hz, 1H, Ar-H), 6.83 (d, J = 8.3 Hz, 1H, Ar-H), 4.96 (s, 2H, NH2), 3.77–3.66 (m, 4H, Piperazine Hs), 2.46–2.36 (m, 4H, Piperazine Hs), 2.22 (s, 3H, CH_3_). ^13^C NMR (101 MHz, DMSO) δ 162.52, 155.56, 142.47, 142.25, 141.65, 133.10, 129.21, 127.00, 126.25, 124.99, 124.70, 124.17, 123.04, 118.31, 117.34, 54.57, 46.16, 44.30. MS m/z: 395.20 [M + H]. HRMS m/z: 395.1652 [M + H]^+^; calculated C_20_H_23_N_6_OS ^+^: 395.1654. HPLC: rt 6.88 min (purity 100%), yield: 80%.

N-(2-Amino-5-(thiophen-2-yl)phenyl)-5-(piperazin-1-yl)pyrazine-2-carboxamide (29b).

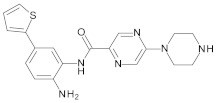



^1^ H NMR (400 MHz, DMSO-d_6_) δ 9.65 (s, 1H, -CO-NH-Ar), 8.70 (d, J = 1.3 Hz, 1H, Ar-H of Pyrazine), 8.32 (d, J = 1.4 Hz, 1H, Ar-H of Pyrazine), 7.78 (d, J = 2.2 Hz, 1H, Ar-H), 7.35 (dd, J = 5.1, 1.1 Hz, 1H, Ar-H), 7.27–7.21 (m, 2H, Ar-H), 7.04 (dd, J = 5.1, 3.6 Hz, 1H, Ar-H), 6.83 (d, J = 8.3 Hz, 1H, Ar-H), 5.07 (s, 2H, -NH_2_), 3.72–3.60 (m, 4H, Piperazine Hs), 2.82–2.71 (m, 4H, Piperazine Hs). ^13^C NMR (101 MHz, dmso) δ 162.60, 155.62, 144.77, 142.55, 141.99, 132.76, 129.10, 128.63, 124.84, 123.82, 123.41, 122.23, 121.61, 117.40, 45.61, 45.42. MS m/z: 379.36 [M − H]^−^, 381.39 [M + H]^+^, HRMS m/z: 381.1494 [M + H]^+^; calculated C_19_H_21_N_6_OS^+^: 381.1497. HPLC: rt 7.20 min (purity 98.5%), yield: 76%.

N-(4-amino-4’-fluoro-[1,1’-biphenyl]-3-yl)-5-(piperazin-1-yl)pyrazine-2-carbox- amide (29c).

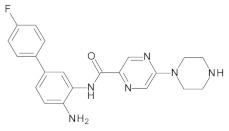



^1^ H NMR (400 MHz, DMSO-d_6_) δ 9.65 (s, 1H, CO-NH-Ar), 8.70 (d, J = 1.3 Hz, 1H, Ar-H of Pyrazine), 8.32 (d, J = 1.2 Hz, 1H, Ar-H of Pyrazine), 7.77 (s, 1H, Ar-H), 7.58–7.53 (m, 2H, Ar-H), 7.25–7.17 (m, 3H, Ar-H), 6.87 (d, J = 8.3 Hz, 1H, Ar-H), 5.02 (s, 2H, NH_2_), 3.71–3.59 (m, 4H, Piperazine Hs), 3.27 (s, 1H, NH), 2.86–2.74 (m, 4H, Piperazine Hs). ^13^C NMR (101 MHz, DMSO) δ 162.56, 160.29, 155.64, 142.52, 141.75, 137.29, 132.76, 129.08, 128.35, 127.90, 124.96, 124.25, 123.13, 117.54, 116.07, 45.69, 45.54. MS m/z: 393.47 [M + H]^+^. HRMS m/z: 393.1837 [M + H]^+^; calculated C_21_H_22_FN_6_O^+^: 393.1839. HPLC: rt 7.07 min (purity 99.6%), yield: 88%.

N-(4-amino-2’-fluoro-[1,1’-biphenyl]-3-yl)-5-(4-methylpiperazin-1-yl)pyrazine-2-carboxamide (29d).

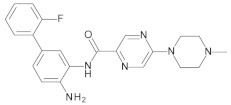



^1^ H NMR (400 MHz, DMSO-d_6_) δ 9.67 (s, 1H, CO-NH-Ar), 8.70 (s, 1H, Ar-H of Pyrazine), 8.35 (s, 1H, Ar-H of Pyrazine), 7.68 (s, 1H, Ar-H), 7.44 (td, J = 7.9, 1.7 Hz, 1H, Ar-H), 7.34–7.11 (m, 4H, Ar-H), 6.88 (d, J = 8.3 Hz, 1H, Ar-H), 5.09 (s, 2H, NH_2_), 3.77–3.65 (m, 4H, Piperazine Hs), 2.64–2.35 (m, 4H, Piperazine Hs), 2.21 (s, 3H, CH_3_). ^13^C NMR (101 MHz, DMSO) δ 162.55, 160.70, 158.27, 155.56, 142.49, 142.18, 133.08, 130.55, 129.19, 126.63, 125.50, 125.19, 124.44, 123.85, 116.99, 116.56, 116.33, 54.57, 46.15, 44.30. HRMS m/z: 407.1992 [M + H]^+^; calculated C_22_H_24_FN_6_O^+^: 407.1995. HPLC: rt 7.44 min (purity 97.9%), yield: 85%.

### 4.2. Biological Evaluation

#### 4.2.1. In Vitro HDAC Inhibition Assay

Recombinant human HDAC1, HDAC2, and HDAC3/NCOR1 were purchased from ENZO Life Sciences AG (Lausen, CH). Recombinant human HDAC 4, HDAC5, HDAC7, HDAC9 and HDAC11 were produced by Barinka lab in Prague, as described before [43,54]. Recombinant human HDAC8 was produced by Romier et al. (IGBMC, Univ. Strasbourg), as described in [55].

The in vitro testing on recombinant HDACs 1-3 were performed with a fluorogenic peptide derived from p53 (Ac-RHKK(Acetyl)-AMC). The measurements were performed in an assay buffer (50 mM Hepes, 150 mM NaCl, 5 mM MgCl_2_, 1 mM TCEP and 0.2 mg/mL BSA, pH 7.4 adjusted with NaOH) at 37 °C. Inhibitors at different concentrations were incubated with 10 nM HDAC1, 3 nM HDAC2 or 3 nM HDAC3 (final concentration) for at least 5 min. The reaction was started with the addition of the fluorogenic substrate (20 µM final concentration) and incubated for 30 min for HDAC2 and HDAC3 and 90 min for HDAC1. The reaction was stopped with a solution of 1 mg/mL trypsin and 20 µM SAHA in 1 mM HCl and incubated for 1 h at 37 °C. The fluorescence intensity was recorded with an Envision 2104 Multilabel Plate Reader (PerkinElmer, Waltham, MA, USA) with an excitation wavelength of 380 ± 8 nm and an emission wavelength of 430 ± 8 nm. The received fluorescence intensities were normalized with uninhibited reaction as 100% and the reaction without enzyme as 0%. A nonlinear regression analysis was done to determine the IC50 value.

The determination of dose response curves for HDAC4, 5, 7 and 9 was performed as previously described [43] with compound 4 as substrate (Abz-SRGGK(thio-TFA)FFRR-NH2). The substrate concentration was 50 µM and the enzyme concentration was 10 nM for HDAC4 and HDAC5, 5 nM for HDAC7 and 20 nM for HDAC9. HDAC11 inhibition assay was performed as described before [56]. The fluorescence intensity was recorded with an Envision 2104 Multilabel Plate Reader (PerkinElmer, Waltham, MA) with an excitation wavelength of 330 ± 75 nm and an emission wavelength of 430 ± 8 nm.

The enzyme inhibition of HDAC8 was determined by using a reported homogenous fluorescence assay 2 [57], The enzymes were incubated for 90 min at 37 °C, with the fluorogenic substrate ZMAL (Z (Ac)Lys-AMC) in a concentration of 10.5 μM and increasing concentrations of inhibitors. Fluorescence intensity was measured at an excitation wavelength of 390 nm and an emission wavelength of 460 nm in a microtiter plate reader (BMG Polarstar).

#### 4.2.2. Cellular Assay

To determine the cytotoxicity of the developed compounds on the human epithelial kidney, cell line HEK293 was used. HEK293 cells (DSMZ Braunschweig, ACC305) were incubated at 37 °C in a humidified incubator with 5% CO_2_ in Dulbecco’s Modified Eagle Medium (DMEM) supplemented with 10% FCS and 5 mM glutamine. Cells were seeded out at 1.5 x 103 cells per well in a 96-well cell culture plate (TPP, Switzerland). The compounds to be tested were added immediately to the medium at 50 µM. After 24 h, Alamar Blue reagent (Invitrogen, CA) was added according to the manufacturer’s instructions and incubated again for 21 h before samples were analyzed. Detection of viable cells which convert the resazurine reagent into the highly fluorescent resorufin was performed by using a FLUOstarOPTIMA microplate reader (BMG Labtec) with the following filter set: Ex 530 nm/Em 590 nm. Measurements were performed in triplicate and data are means with standard deviation <12%. Daunorubicin was used as a positive control and an IC50 value of 12.55 ± 0.07 µM was measured.

All leukemic cells were kept in RPMI-1640 medium supplemented with 10% FBS and 1%penicillin/streptomycin (Sigma-Aldrich, Taufkirchen, Germany) under standard culture conditions at 37 °C and a 5% CO_2_ humidified atmosphere. Cells were authenticated as mentioned by us [51].

### 4.3. Computational Studies

Protein structures were retrieved from the Protein Data bank (PDB) (PDB ID: 4BKX, 4LY1. 4A69) [58]. The HDAC1 (PDB ID: 4BKX) in apo-form and HDAC3 (PDB ID: 4A69) in apo-form were minimized with the ligand of HDAC2 (PDB ID: 4LY1) and BG-45 molecules [59], respectively. All protein structures were prepared using the Protein Preparation Wizard module in Schrödinger Suite [60]. Hydrogen atoms and missing side chains were added. With the exception of a conserved water molecule bound to a conserved histidine in HDAC1, 2 and 3, all water molecules were removed from the X-ray structures. The protonation states and tautomeric forms of the amino acids were optimized using PROPKA tool at pH 7.0. The potential energy of the three optimized structures was minimized using OPLS3e force-field [61]. Ligands were prepared using the LigPrep module in Schrödinger Suite using OPLS3e force-field. Conformations of prepared ligands were generated using the Confgen tool in Schrödinger Suite by applying 64 conformers per each ligand and minimizing the conformers. Molecular docking studies were conducted by applying the Glide program in Schrödinger Suite. The grid box was generated with 10*10*10 Å size using the Receptor Grid Generation module in Schödinger19. Standard Precision (SP) mode with flexible ligand sampling was utilized for docking. To validate the docking protocol, re-docking studies were done. The RMSD values of the re-docking studies corresponding to the binding mode in HDAC1, HDAC2 and HDAC3 are observed as 0.17, 0.29, 0.28 Å, respectively. Docking poses were visualized in the MOE2018.01 program [62].

Molecular dynamics (MD) simulations were carried out using Amber18 [63]. MD systems were generated using the obtained docking poses of 19f, 21a, 23a, and 29b. First, the antechamber module was used to prepare the topologies and force field parameters of the ligands using the general Amber Force Field (GAFF) [63] and AM1-BCC as the atomic charges method semi empirical (AM1), and bond charge correction (BCC) [64,65]. Then, MD systems were generated using the TLeaP package and the ff03 force field for protein and GAFF for the ligands. The systems were solvated with the TIP3P solvation model. The octahedral box was generated with 10 Å. The prepared systems were used to run MD simulation. The simulation protocol includes different steps. Initially, two minimization steps were carried out. In each step, 4000 iterations, including the first 3000 steepest descent and 1000 conjugate gradient, were subjected to the MD systems. First, only solvent atoms were minimized in the first minimization step, while protein and ligand atoms were kept in their initial coordinates with a force constant of 10 kcal mol -1Å -1. Then, in the second minimization, the whole system, including the protein and ligand, were minimized. Subsequently, the system was heated from 0 K to 300 K through 100 ps MD. The complex atoms were again restrained with a force constant of 10 kcal mol -1Å -1 to prevent the large structural deviation. Next, the density was evaluated during 100ps MD. Afterwards, the systems were equilibrated through 200 ps MD before the MD step. The SHAKE algorithm was used to restrain all bonds involving hydrogens [66]. Temperature was controlled by Langevin Dynamics using a collision frequency of 2 ps-1 and pressure of 1 bar. In the MD step, 100 ns MD simulation were performed for each system. The trajectories were analyzed using the CPPTRAJ module and VMD [67].

### 4.4. PAINS Filter

All the herein described compounds were filtered for pan-assay interference compounds (PAINS) [68]. For this purpose, PAINS1, PAINS2 and PAINS3 filters, as implemented in Schrödinger’s Canvas program, were employed. None of the compounds were flagged as a PAIN.

### 4.5. In Silico Prediction of Pharmacokinetic and Tox Data

For the in silico prediction the PreADMET web application was used (https://preadmet.bmdrc.kr/admetox/ accessed date 10 November 2021). The PreADMET approach is based on different classes of molecular descriptors that are considered for generating the quantitative structure property relationship or binary classification models. The following properties were calculated: human intestinal absorption (% HIA) [69], plasma protein binding, water solubility, AlogP classification models which were used to predict the inhibition of several cytochromes, hERG and para-glycoprotein (p-gp). To predict the human toxicity the ProTox-II approach [70] which is available as web service (https://tox-new.charite.de/protox_II/ accessed date 10 November 2021) was used. ProTox-II uses molecular similarity, fragment propensities, most frequent features and (fragment similarity based cross-validation) machine-learning, based on a total of 33 models for the prediction of various toxicity endpoints, such as acute toxicity, hepatotoxicity, cytotoxicity, carcinogenicity, mutagenicity, immunotoxicity, adverse outcomes (Tox21) pathways and toxicity targets.

## Figures and Tables

**Table 1:**
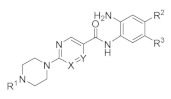
Inhibitory activity of the synthesized compounds against class I HDACs.

Cpd. No.	X	Y	R ^1^	R ^2^	R ^3^	HDAC1 (IC_50_ µM)	HDAC2 (IC_50_ µM)	HDAC3 (IC_50_ µM)	HDAC8 (% Inhib.)
19a	CH	N	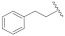	H	H	0.51 ± 0.05	0.80 ± 0.07	1.12 ± 0.07	0% @ 1 µM
19b	CH	N	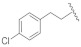	H	H	25.8% @ 2 µM	30.3% @ 2 µM	65.2% @2 µM	3.4% @ 1 µM
19c	N	CH	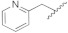	H	H	33.9% @ 2 µM	20.1% @ 2 µM	26.8% @2 µM	0% @ 1 µM
19d	CH	N	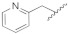	H	H	0.52 ± 0.07	1.43 ± 0.08	1.06 ± 0.04	0% @ 1 µM
19e	CH	N	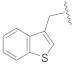	H	H	0.21 ± 0.07	0.71 ± 0.04	0.84 ± 0.03	0% @ 1 µM
19f	CH	N	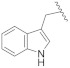	H	H	0.13 ± 0.01	0.28 ± 0.01	0.31 ± 0.01	0% @ 1 µM
19g	CH	N	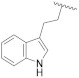	H	H	0.31 ± 0.03	0.96 ± 0.05	0.49 ± 0.06	0% @ 1 µM
19h	CH	N	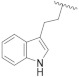	F	F	0.81 ± 0.07	0.74 ± 0.03	0.57 ± 0.02	n.d.
19i	CH	N	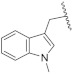	Cl	H	3.0 ± 0.2	2.7 ± 0.2	1.9 ± 0.1	n.d.
19j	N	CH	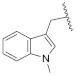	H	H	0.45 ± 0.06	0.93 ± 0.04	1.75 ± 0.06	0% @ 1 µM
19k	CH	N	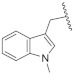	H	H	0.14 ± 0.02	0.56 ± 0.04	0.59 ± 0.03	0% @ 1 µM
19l	CH	N	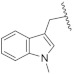	F	F	0.29 ± 0.03	0.56 ± 0.02	0.81 ± 0.05	n.d.
19m	CH	N	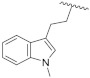	F	H	0.40 ± 0.06	1.48 ± 0.19	0.40 ± 0.02	n.d.
19n	N	CH	CH_3_	H	H	5% @ 1 µM	7% @ 1 µM	13% @ 1 µM	n.d.
19o	CH	N	CH_3_	H	H	27% @ 1 µM	15% @ 1 µM	30% @ 1 µM	n.d.
21a	CH	N	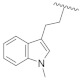	H	2-Thienyl	0.26 ± 0.01	2.47 ± 0.22	0% @ 1 µM	n.d.
21b	CH	N	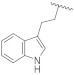	H	4-F-C_6_H_4_	0.70 ± 0.08	0.77 ± 0.06	0% @ 1 µM	0% @ 1 µM
21c	CH	N	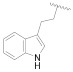	H	2-F-C_6_H_4_	0.76 ± 0.07	0.76 ± 0.04	15 ± 1	0% @ 1 µM
23a	CH	N	H	F	H	3.30 ± 0.18	2.20 ± 0.20	0.40 ± 0.01	0% @ 1 µM
23b	CH	N	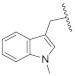	H	F	0.27 ± 0.03	0.50 ± 0.03	0.50 ± 0.02	38% @ 1 µM
23c	CH	N	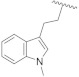	H	F	0.33 ± 0.02	1.37 ± 0.08	0.59 ± 0.04	n.d.
25a	CH	N	H	Cl	H	0% @ 1 µM	0% @ 1 µM	8.7 ± 0.4	n.d.
25b	CH	N	H	F	F	4.3 ± 0.3	4.2 ± 0.10	1.6 ± 0.1	n.d.
27a	CH	N	H	H	CF_3_	0% @ 1 µM	0% @ 1 µM	0% @ 1 µM	n.d.
27b	CH	N	H	CH_3_	H	0% @ 1 µM	0% @ 1 µM	0% @ 1 µM	n.d.
27c	CH	N	H	OCH_3_	H	20.0 ± 1.0	14.0 ± 2.0	14.0 ± 1.0	n.d.
29a	CH	N	CH_3_	H	3-Thienyl	0.11 ± 0.01	0.18 ± 0.06	4.4 ± 0.1	n.d.
29b	CH	N	H	H	2-Thienyl	0.07 ± 0.01	0.26 ± 0.01	6.1 ± 0.7	n.d.
29c	CH	N	H	H	4-F-C_6_H_4_	0.16 ± 0.03	0.34 ± 0.01	6.7 ± 0.5	n.d.
29d	CH	N	CH_3_	H	2-F-C_6_H_4_	0.18 ± 0.01	0.26 ± 0.07	12.0 ± 1.0	n.d.
CI994	--	--	--	--	--	37% @ 1 µM	36% @ 1 µM	32% @ 1 µM	n.d.
RGFP-966	--	--	--	--	--	16 ± 2	11 ± 1	1.3 ± 0.1	n.d.
MS-275	--	--	--	--	--	0.93 ± 0.1	0.95 ± 0.03	1.8 ± 0.1	n.d.
Mocetinostat	--	--	--	--	--	0.33 ± 0.04	0.34 ± 0.01	0.93 ± 0.05	n.d.

n.d. not determined, -- no substituents.

**Table 2:** Inhibitory activity of compounds 19f and 21a against class II and class IV HDACs.

Cpd. No.	HDAC4 (IC_50_ µM)	HDAC5 (IC_50_ µM)	HDAC6 (IC_50_ µM)	HDAC7 (IC_50_ µM)	HDAC9 (IC_50_ µM)	HDAC11 (IC_50_ µM)
19f	>20	>20	>20	>20	>20	>20
21a	>20	>20	>20	>20	>20	7.5 ± 1

**Table 3:** Cytotoxicity studies in human HEK293 Cells.

Name	% Viability	SD	Name	% Viability	SD
19a	73.1	3.4	19m	52.5	0.5
19b	78.6	6.8	21a	94.4	5.5
19c	78.7	7.5	21b	71.0	4.1
19d	67.7	7.3	21c	72.3	5.8
19e	50.9	4.0	23a	67.0	3.4
19f	76.1	8.0	23b	58.7	6.5
19g	71.8	3.0	23c	55.7	1.5
19h	54.5	2.8	25b	83.7	4.4
19i	47.5	1.5	29a	65.8	6.8
19j	62.5	5.8	29b	2.4	0.0
19k	71.4	3.0	29c	2.2	0.1
19l	20.1	0.4	29d	54.0	2.2

**Table 4:** In silico ADME and Tox prediction for the most promising candidates 19f and 21a as well as the reference inhibitor MS-275. For predicting the properties PreADMET (https://preadmet.bmdrc.kr/admetox/ accessed date 10 Novermber 2021) and ProTox-II (https://tox-new.charite.de/protox_II/ accessed date 10 Novermber 2021) web applications were used.

	MS-275	19f	21a
Human intestinal absorption%	92.54	93.53	96.14
AlogP98 value	2.07	0.78	3.11
Plasma Protein Binding%	91.07	34.78	77.36
Pure water solubility mg/L	19.92	434.20	3.69
CYP_2C19_inhibition	None	None	None
CYP_2C9_inhibition	Inhibitor	Inhibitor	Inhibitor
CYP_2D6_inhibition	None	None	Non
CYP_2D6_substrate	None	Substrate	Substrate
CYP_3A4_inhibition	Inhibitor	None	None
CYP_3A4_substrate	Substrate	Substrate	Substrate
hERG_inhibition	high risk	moderate risk	high risk
Pgp inhibition	None	None	None
Predicted LD_50_	22 mg/kg	1500 mg/kg	600 mg/kg
Toxicity prediction ProTox-II	0/17	0/17	1/17

## Data Availability

Not applicable.

## Supplementary Materials

The following are available online at https://www.mdpi.com/article/10.3390/ijms23010369/s1.
