# Supplementary material for: Synthesis, Molecular Docking and Biological Characterization of Pyrazine Linked 2-Aminobenzamides as New Class I Selective Histone Deacetylase (HDAC) Inhibitors with Anti-Leukemic Activity
Source: Int J Mol Sci. 2021 Dec 29;23(1):369. doi: 10.3390/ijms23010369 (PMC8745332; doi:10.3390/ijms23010369)
Supplement: Supplementary file 1 [file ijms-23-00369-s001.zip › ijms-1527920-supplementary.pdf]

Synthesis, molecular docking and biological characterization of pyrazine linked 2-aminobenzamides as new class I selective histone deacetylase (HDAC) inhibitors with anti-leukemic activity

Hany S. Ibrahim,[1,2#] Mohamed Abdelsalam,[1,3#] Yanira Zeyn,[4] Matthes Zessin,[1,5] Al-Hassan M. Mustafa,[4,6], Marten A. Fischer,[4] Patrik Zeyen,[1] Ping Sun,[1] Emre F. Bülbül,[1] Anita Vecchio,[1] Frank Erdmann,[1] Matthias Schmidt,[1] Dina Robaa,[1] Cyril Barinka,[7] Christophe Romier,[8] Mike Schutkowski,[5] Oliver H. Krämer,[4*] and Wolfgang Sippl,[1*]

[1]*Department of Medicinal Chemistry, Institute of Pharmacy, Martin-Luther-University of Halle-Wittenberg, Halle (Saale), Germany*

[2]*Department of Pharmaceutical Chemistry, Faculty of Pharmacy, Egyptian Russian University, Badr City, Cairo 11829, Egypt*

[3]*Department of Pharmaceutical Chemistry, Faculty of Pharmacy, Alexandria University, Alexandria 21521, Egypt*

[4]*Department of Toxicology, University Medical Center, 55131 Mainz, Germany.*

[5]*Department of Enzymology, Institute of Biochemistry, Martin-Luther-University of Halle-Wittenberg, Halle (Saale), Germany*

[6]*Department of Zoology, Faculty of Science, Aswan University 81528, Aswan, Egypt*

[7]*Institute of Biotechnology of the Czech Academy of Sciences, BIOCEV, Prumyslova 595, 252 50 Vestec, Czech Republic*

[8]*Université de Strasbourg, CNRS, INSERM, Institut de Génétique et de Biologie Moléculaire et Cellulaire (IGBMC), Département de Biologie Structurale Intégrative, 67404 Illkirch Cedex, France*

Table of contents

	Topic	Page
1	**List of tables**	**S2**
2	**Experimental procedure and data for synthesized intermediates**	**S5**
3	**^{1}H NMR charts of final compounds**	**S33**
4	**^{13}C NMR charts of final compounds**	**S48**
5	**HRMS spectra of final compounds**	**S61**
6	**HPLC Spectra for final compounds**	**S75**
7	**IC_{50} plots of compound 19f and 21a against different HDACs**	**S91**

isoforms

8	IC_{50} plots of reference inhibitors against HDAC1, -2, and -3	S95
9	IC_{50} plots of tested inhibitors against HDAC1, -2, and -3	S96
10	Molecular docking studies	S102
11	References	S108

1. List of Tables

Table S1a. *Carboxylic acid intermediates 3a-e and 4a-c*

Cpd. No.	X	Y	R^1	R^2
**3a**	CH	N	Me	Me
**3b**	N	CH	Et	Me
**3c**	CH	N	Me	Boc
**3d**	CH	N	Me	H
**3e**	N	CH	Et	H
**4a**	CH	N	-	Me
**4b**	N	CH	-	Me
**4c**	CH	N	-	Boc

Table S1b. Carboxylic acid intermediates 5a-f,6a-d and 7a-j

No.	X	Y	R^1	Ar^1	Ar^2	No.	X	Y	n	Ar^3
**5a**	**N**	**CH**	Et	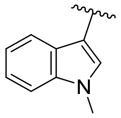	--	**7a**	**N**	**CH**	1	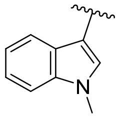
**5b**	**N**	**CH**	Et	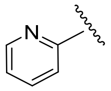	--	**7b**	**N**	**CH**	1	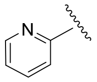
**5c**	**CH**	**N**	Me	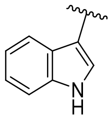	--	**7c**	**CH**	**N**	1	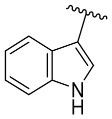
**5d**	**CH**	**N**	Me	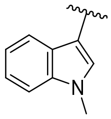	--	**7d**	**CH**	**N**	1	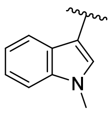

**5e**	**CH**	**N**	Me	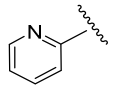	--	**7e**	**CH**	**N**	1	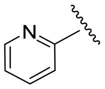
**5f**	**CH**	**N**	Me	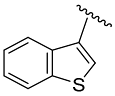	--	**7f**	**CH**	**N**	1	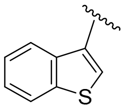
**6a**	**CH**	**N**	Me	--	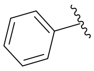	**7g**	**CH**	**N**	2	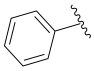
**6b**	**CH**	**N**	Me	--	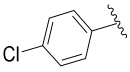	**7h**	**CH**	**N**	2	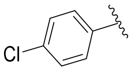
**6c**	**CH**	**N**	Me	--	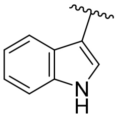	**7i**	**CH**	**N**	2	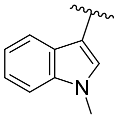
**6d**	**CH**	**N**	Me	--	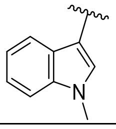	**7j**	**CH**	**N**	2	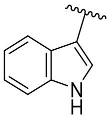

Table S2. *Substituted 1,2-phenylenediamine derivatives in* ***Scheme 2****.*

**Cpd. No.**	**R**^{3}	**R**^{4}	**R**^{5}	**R**^{6}	**R**^{7}
**8,9a**	H	F	-	-	-
**8,9b**	F	F	-	-	-
**8,9c**	H	Cl	-	-	-
**10-12a**	-	-	CH_3	H	-
**10-12b**	-	-	OCH_3	H	-
**10-12c**	-	-	H	CF_3	-
**10-12d**	-	-	F	H	-
**10-12e**	-	-	H	F	-
**15-17a**	-	-	-	-	2-Thienyl
**15-17b**	-	-	-	-	3-Thienyl
**15-17c**	-	-	-	-	4-F-C_6H_4
**15-17d**	-	-	-	-	2-F-C_6H_4

Table S3. *Substitutions of compounds 19a-o*

**No.**	**X**	**Y**	**R**^{3}	**R**^{4}	**R**^{8}	**No.**	**X**	**Y**	**R**^{3}	**R**^{4}	**R**^{8}
**19a**	CH	N	H	H	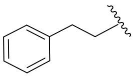	**19h**	CH	N	F	F	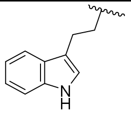
**19b**	CH	N	H	H	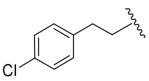	**19i**	CH	N	Cl	H	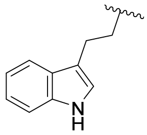

**19c**	N	CH	H	H	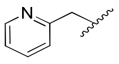	**19j**	N	CH	H	H	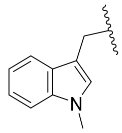
**19d**	CH	N	H	H	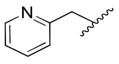	**19k**	CH	N	H	H	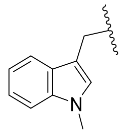
**19e**	CH	N	H	H	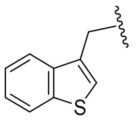	**19l**	CH	N	F	F	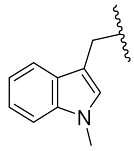
**19f**	CH	N	H	H	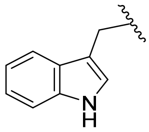	**19 m**	CH	N	F	H	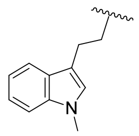
**19g**	CH	N	H	H	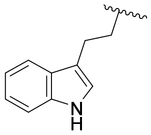	**19n**	N	CH	H	H	CH_3
**19o**	CH	N	H	H	CH_3						

Table S4. Substitutions of intermediates 24a,b, 26a-c and 28a-d and final compounds 25a,b, 27a-c and 29a-d

Cpd. No.	R[2]	R[3]	R[4]	R[5]	R[6]	R[7]
**24a,25a**	-	H	Cl	-	-	-
**24b, 25b**	-	F	F	-	-	-
**26a,27a**	-	-	-	H	CF_3	-
**26b,27b**	-	-	-	CH_3	H	-
**26c,27c**	-	-	-	OCH_3	H	-
**28a,29a**	CH_3	-	-	-	-	3-Thienyl
**28b,29b**	(Boc)H*	-	-	-	-	2-Thienyl
**28c,29c**	(Boc)H*	-	-	-	-	4-F-C_6H_4
**28d,29d**	CH_3	-	-	-	-	2-F-C_6H_4

(*)Boc for 28b,c and H for 29b,c.

2. Experimental procedure and data for synthesized intermediates

Materials and reagents were purchased from Sigma-Aldrich Co. Ltd. and abcr GmbH. All solvents were analytically pure and dried before use. Thin layer chromatography was carried out on aluminum sheets coated with silica gel 60 F254 (Merck, Darmstadt, Germany). For medium pressure chromatography (MPLC) silica gel 60 (0.036e0.200 mm) was used. Final compounds were confirmed to be of >95% purity based on HPLC. Purity was measured by UV absorbance at 254 nm. The HPLC consists of an XTerra RP18 column (3.5 µm, 3.9 mm x 100 mm) from the manufacturer Waters (Milford, MA, USA) and two LC-10AD pumps, a SPD-M10A VP PDA detector, and a SIL-HT autosampler, all from the manufacturer Shimadzu (Kyoto, Japan). Mass spectrometry analyses were performed with a Finnigan MAT710C (Thermo Separation Products, San Jose, CA, USA) for the ESIMS spectra and with a LTQ (linear ion trap) Orbitrap XL hybrid mass spectrometer (Thermo Fisher Scientific, Bremen, Germany) for the HRMS-ESI (high resolution mass spectrometry) spectra. For the HRMS analyses the signal for the isotopes with the highest prevalence was given and calculated (^{35}Cl, ^{79}Br). ^{1}H NMR spectra were taken on a Varian Inova 400 using deuterated DMSO as solvent. Chemical shifts are referenced to the residual solvent signals. The following abbreviations and formulas for solvents and reagents were used: ethyl acetate (EtOAc), dimethylformamide (DMF), dimethylsulfoxide (DMSO), methanol (MeOH), tetrahydrofuran (THF), chloroform ($CHCl_3$), water (H_2O), dichloromethane (DCM), N,N-diisopropylethylamine (DIPEA), trimethylamine (TEA), hydrochloric acid (HCl) and trifluoroacetic acid (TFA).

*General procedure for synthesis of different methyl/ethyl 5/2-(piperazin-1-yl) pyrazine/ pyrimidine -2/5-carboxylate (**3a-e**)*

To a solution of methyl 5-chloropyrazine-2-carboxylate (**1a**) or ethyl 2-chloropyrimidine-5-carboxylate (**1b**) 10 mmol in 20 ml of toluene, 30 mmol the appropriate piperazine derivative **2a-c** was added and the reaction mixture was heated at 130°C. After one hour, the reaction was poured on ice and the resulted mixture was extracted with chloroform, washed with brine, dried and concentrated. The resulted solid was washed with diethyl ether to obtain the targeted products **3a-e**.

*Methyl 5-(4-methylpiperazin-1-yl)pyrazine-2-carboxylate (**3a**)*

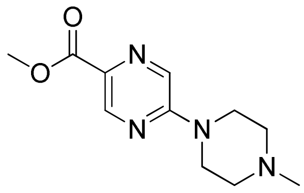

^{1}H NMR (400 MHz, DMSO-*d*$_6$) δ 8.63 (d, *J* = 1.3 Hz, 1H, Ar-***H*** of Pyrazine), 8.35 (d, *J* = 1.3 Hz, 1H, Ar-***H*** of Pyrazine), 3.79 (s, 3H, -OC***H***$_3$), 3.73 – 3.67 (m, 4H, Piperazine ***H***s), 2.41 – 2.36 (m, 4H, Piperazine ***H***s), 2.20 (s, 3H, -NC***H***$_3$), Yield: 85.1%.

*Ethyl 2-(4-methylpiperazin-1-yl)pyrimidine-5-carboxylate (**3b**)*

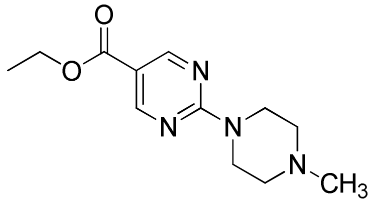

^{1}H NMR (400 MHz, DMSO-d_6) δ 8.75 (s, 2H, Ar-***H*** of Pyrimidine), 4.25 (q, *J* = 7.1 Hz, 2H, -OC***H***$_2$CH$_3$), 3.82 – 3.75 (m, 4H, Piperazine ***H***s), 2.39 – 2.29 (m, 4H, Piperazine ***H***s), 2.19 (s, 3H, -NC***H***$_3$), 1.27 (t, *J* = 7.1 Hz, 3H, -OCH$_2$C***H***$_3$), Yield: 75 %

*Methyl 5-(4-(tert-butoxycarbonyl)piperazin-1-yl)pyrazine-2-carboxylate (**3c**)*

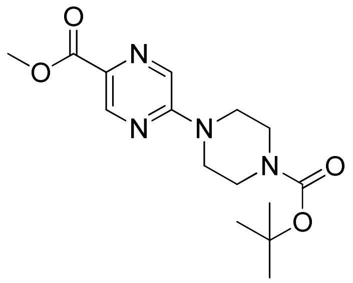

^{1}H NMR (400 MHz, DMSO-d_6) δ 8.64 (d, *J* = 1.3 Hz, 1H, Ar-***H*** of Pyrazine), 8.35 (d, *J* = 1.4 Hz, 1H, Ar-***H*** of Pyrazine), 3.80 (s, 3H, -OC***H***$_3$), 3.75 – 3.68 (m, 4H, Piperazine ***H***s), 3.45 – 3.42 (m, 4H, Piperazine ***H***s), 1.41 (s, 9H, -OC(C***H***$_3$)$_3$). MS m/z: 323.0 $[M+H]^+$, Yield: 79 %

*Methyl 5-(piperazin-1-yl)pyrazine-2-carboxylate (**3d**)*

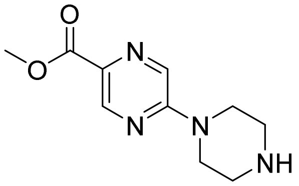

^{1}H NMR (400 MHz, DMSO-d_6) δ 8.61 (d, *J* = 1.3 Hz, 1H, Ar-***H*** of Pyrazine), 8.31 (d, *J* = 1.4 Hz, 1H, Ar-***H*** of Pyrazine), 3.79 (s, 3H, -OC***H***$_3$), 3.75 – 3.49 (m, 4H, Piperazine ***H***s), 3.29 (s, 1H, -N***H***), 2.82 – 2.66 (m, 4H, Piperazine ***H***s). MS m/z: 223.10 $[M+H]^+$. Yield: 87%

Ethyl 2-(piperazin-1-yl)pyrimidine-5-carboxylate **(3e)**

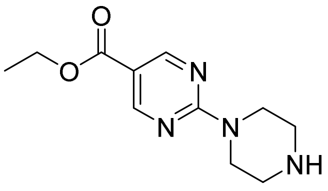

^1H NMR (400 MHz, DMSO-d_6) δ 8.74 (s, 2H, Ar-***H*** of pyrimidine), 4.24 (q, *J* = 7.1 Hz, 2H, -OC$\boldsymbol{H}_2$CH$_3$), 3.80 – 3.69 (m, 4H, Piperazine ***H***s), 3.29 (s, 1H, -N***H***), 2.75 – 2.68 (m, 4H, Piperazine ***H***s), 1.27 (t, *J* = 7.1 Hz, 3H, -OCH$_2$C$\boldsymbol{H}_3$). MS m/z: 237.10 $[M+H]^+$. Yield: 73 %.

*General procedure for synthesis of methyl/ethyl 5-(4-(methylaryl)piperazin-1-yl)pyrazine/pyrimidine-2/5-carboxylate (**5a-f**)*

To 20 ml dry DCM, dissolve (5.0 mmol) of methyl 5-(piperazin-1-yl)pyrazine-2-carboxylate (**3d**) or ethyl 2-(piperazin-1-yl)pyrimidine-5-carboxylate (**3e**) and (5.0 mmol) of the corresponding aryl aldehyde followed by 1ml glacial acetic acid and stir at RT for 30 min. Three equivalent of Sodium triacetoxy borohydride (15 mmol, 3.16 g) were added to the reaction mixture and stirred at RT. The progress of reaction was monitored by TLC till the disappearance of starting materials. Consequently, the reaction was quenched by addition of 10 ml 1N NaOH and stirring for 30 min. The organic layer was separated, washed with brine and concentrated under vacuum. The obtained solid was purified using MPLC ($CHCl_3$:MeOH).

Ethyl 2-(4-((1-methyl-1H-indol-3-yl)methyl)piperazin-1-yl)pyrimidine-5-carboxylate **(5a)**

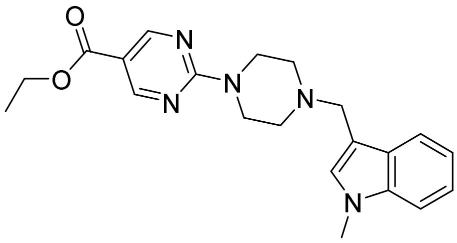

^{1}H NMR (400 MHz, DMSO-d_6) δ 8.73 (s, 2H, Ar-***H*** of Pyrimidine), 7.64 (dt, *J* = 7.9, 1.0 Hz, 1H, Ar-***H*** of indole), 7.37 (dt, *J* = 8.2, 0.9 Hz, 1H, Ar-***H*** of indole), 7.22 (s, 1H, Ar-***H*** of indole), 7.12 (ddd, *J* = 8.2, 7.0, 1.2 Hz, 1H, Ar-***H*** of indole), 7.01 (ddd, *J* = 7.9, 7.0, 1.0 Hz, 1H, Ar-***H*** of indole), 4.24 (q, *J* = 7.1 Hz, 2H, -OC$\boldsymbol{H}_2$CH$_3$), 3.82 (t, *J* = 5.1 Hz, 4H, Piperazine ***H***s), 3.73 (s, 3H, -N-C$\boldsymbol{H}_3$), 3.65 (s, 2H, -N-C$\boldsymbol{H}_2$-Indole), 2.45 (t, *J* = 5.1 Hz, 4H, Piperazine ***H***s), 1.26 (t, *J* = 7.1 Hz, 3H, -OCH$_2$C$\boldsymbol{H}_3$). MS m/z: 378.43 [M-H]$^-$. Yield: 40 %

Ethyl 2-(4-(pyridin-2-ylmethyl)piperazin-1-yl)pyrimidine-5-carboxylate **(5b)**

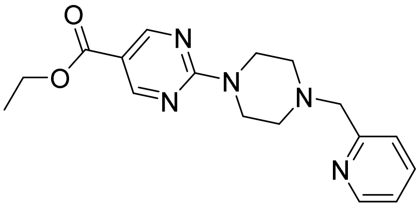

^{1}H NMR (400 MHz, Chloroform-*d*) δ 8.82 (s, 2H, Ar-***H*** of Pyrimidine), 8.59 (ddd, *J* = 4.9, 1.8, 0.9 Hz, 1H, Ar-***H*** of Pyridine), 7.69 (td, *J* = 7.7, 1.8 Hz, 1H, Ar-***H*** of Pyridine), 7.48 (s, 1H, Ar-***H*** of Pyridine), 7.21 (dd, *J* = 7.4, 5.1 Hz, 1H, Ar-***H*** of Pyridine), 4.33 (q, *J* = 7.1 Hz, 2H, -OC$\boldsymbol{H}_2$CH$_3$), 4.01 (br. s, 4H, Piperazine ***H***s), 3.76 (s, 2H, -N-C$\boldsymbol{H}_2$-Indole), 2.63 (br. s, 4H, Piperazine ***H***s), 1.35 (t, *J* = 7.1 Hz, 3H, -OCH$_2$C$\boldsymbol{H}_3$). MS m/z: 328.28 [M+H]$^+$, Yield: 57 %.

Methyl 5-(4-((1H-indol-3-yl)methyl)piperazin-1-yl)pyrazine-2-carboxylate **(5c)**

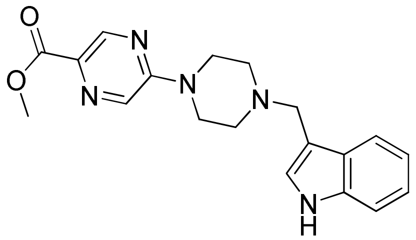

^{1}H NMR (400 MHz, DMSO-d_6) δ 10.93 (s, 1H, -N***H***), 8.61 (d, *J* = 1.3 Hz, 1H, Ar-***H*** of Pyrazine), 8.32 (d, *J* = 1.4 Hz, 1H, Ar-***H*** of Pyrazine), 7.64 (d, *J* = 8.0 Hz, 1H, Ar-***H*** of indole), 7.33 (d, *J* = 8.0 Hz, 1H, Ar-***H*** of indole), 7.23 (s, 1H, Ar-***H*** of indole), 7.05 (ddd, *J* = 8.1, 7.0, 1.2 Hz, 1H, Ar-***H*** of indole), 6.97 (ddd, *J* = 8.2, 7.0, 1.1 Hz, 1H, Ar-***H*** of indole), 3.78 (s, 3H, -OC$\boldsymbol{H}_3$), 3.74 – 3.53 (m, 6H, -NC$\boldsymbol{H}_2$-Indole + Piperazine ***H***s), 2.51 – 2.49 (m, 4H, Piperazine ***H***s). Yield: 75 %.

Methyl 5-(4-((1-methyl-1H-indol-3-yl)methyl)piperazin-1-yl)pyrazine-2-carboxylate **(5d)**

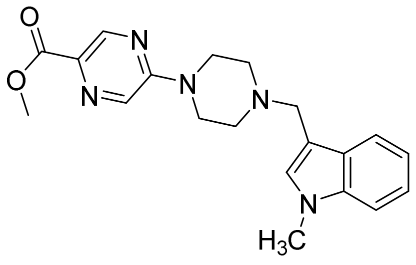

^{1}H NMR (400 MHz, DMSO-d_6) δ 8.61 (d, *J* = 1.3 Hz, 1H, Ar-***H*** of Pyrazine), 8.32 (d, *J* = 1.4 Hz, 1H, Ar-***H*** of Pyrazine), 7.65 (d, *J* = 7.8 Hz, 1H, Ar-***H*** of indole), 7.37 (dt, *J* = 8.2, 0.9 Hz, 1H, Ar-***H*** of indole), 7.22 (s, 1H, Ar-***H*** of indole), 7.12 (ddd, *J* = 8.2, 7.0, 1.2 Hz, 1H, Ar-***H*** of indole), 7.01 (ddd, *J* = 8.0, 7.0, 1.0 Hz, 1H, Ar-***H*** of indole), 3.78 (s, 3H, -NC$\boldsymbol{H}_3$), 3.74 (s, 3H, -OC$\boldsymbol{H}_3$), 3.70 – 3.68 (m, 4H, Piperazine ***H***s), 3.66 (s, 2H, -N-C$\boldsymbol{H}_2$-Indole), 2.51 – 2.49 (m, 4H, Piperazine ***H***s). MS m/z: 365.96 $[M+H]^+$, Yield: 62 %.

Methyl 5-(4-(pyridin-2-yl)methyl)piperazin-1-yl)pyrazine-2-carboxylate **(5e)**

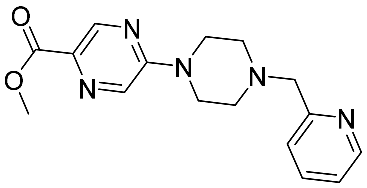

^{1}H NMR (400 MHz, DMSO-d_6) δ 8.62 (d, *J* = 1.3 Hz, 1H, Ar-***H*** of Pyrazine), 8.49 (ddd, *J* = 4.9, 1.8, 0.9 Hz, Ar-***H*** of Pyridine), 8.34 (d, *J* = 1.4 Hz, 1H, Ar-***H*** of Pyrazine), 7.77 (td, *J* = 7.7, 1.8 Hz, 1H, Ar-***H*** of Pyridine), 7.50 – 7.43 (m, 1H, Ar-***H*** of Pyridine), 7.26 (ddd, *J* = 7.5, 4.9, 1.2 Hz, 1H, Ar-***H*** of Pyridine), 3.79 (s, 3H, -OC$\boldsymbol{H}_3$), 3.76 – 3.69 (m, 4H, Piperazine ***H***s), 3.65 (s, 2H, -NC$\boldsymbol{H}_2$-Pyridine), 2.54 – 2.52 (m, 4H, Piperazine ***H***s). MS m/z: 314.36 $[M+H]^+$, Yield: 72 %.

Methyl 5-(4-(benzo[b]thiophen-3-ylmethyl)piperazin-1-yl)pyrazine-2-carboxylate **(5f)**

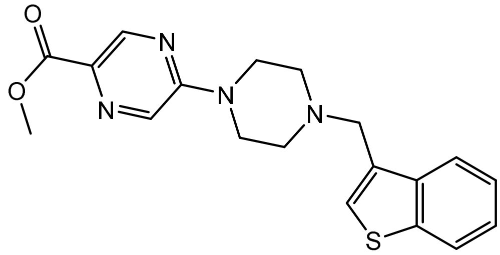

^{1}H NMR (400 MHz, DMSO-d_6) δ 8.62 (d, *J* = 1.3 Hz, 1H, Ar-***H*** of Pyrazine), 8.34 (d, *J* = 1.4 Hz, 1H, Ar-***H*** of Pyrazine), 8.03 – 7.99 (m, 1H, Ar-***H*** of benzothiophene), 7.98 – 7.94 (m, 1H, Ar-***H***, of benzothiophene), 7.60 (s, 1H, Ar-***H*** of benzothiophene), 7.43 – 7.28 (m, 2H, Ar-***H*** of benzothiophene), 3.79 (s, 3H, -OC$\boldsymbol{H}_3$), 3.77 (s, 2H, -N-C$\boldsymbol{H}_2$- benzothiophene), 3.71 (t, *J* = 5.1 Hz, 4H, Piperazine ***H***s), 2.53 (t, *J* = 5.1 Hz, 4H, Piperazine ***H***s). MS m/z: 369.17 $[M+H]^+$, Yield: 52 %

General procedure for the synthesis of methyl 5-(4-Arylethylpiperazin-1-yl)pyrazine-2-carboxylate

Methyl 5-(piperazin-1-yl)pyrazine-2-carboxylate (**3d**) (1 mmol, 0.22 g) was dissolved with (1.1 mmol) of the corresponding arylethyl bromide in 5 ml dry

acetonitrile. 3 eq. K_2CO_3 was added and the reaction mixture was reacted in MW at 120 °C for 1 h. The obtained mixture was filtered and the precipitate was washed with excess acetonitrile. The filtrate was concentrated under vacuum and the given solid was purified using MPLC (CH_3Cl: MeOH).

Methyl 5-(4-phenethylpiperazin-1-yl)pyrazine-2-carboxylate **(6a)**

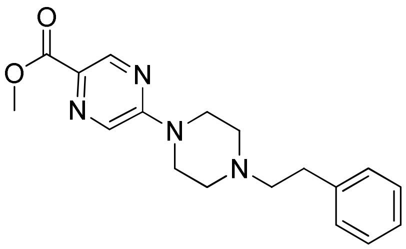

^{1}H NMR (400 MHz, DMSO-d_6) δ 8.63 (d, J = 1.3 Hz, 1H, Ar-***H*** of Pyrazine), 8.36 (d, J = 1.4 Hz, 1H, Ar-***H*** of Pyrazine), 7.33 – 7.19 (m, 4H, Ar-***H***), 7.20 – 7.12 (m, 1H, Ar-***H***), 3.79 (s, 3H, -OC$\boldsymbol{H}_3$), 3.74 – 3.66 (m, 4H, Piperazine ***H***s), 2.75 (dd, J = 9.4, 6.2 Hz, 2H, -N-C$\boldsymbol{H}_2$$CH_2$-Ar), 2.60 – 2.51 (m, 6H, -N-CH_2C$\boldsymbol{H}_2$-Ar + Piperazine ***H***s). MS m/z: 327.19 $[M+H]^+$, Yield: 51 %

Methyl 5-(4-(4-chlorophenethyl)piperazin-1-yl)pyrazine-2-carboxylate **(6b)**

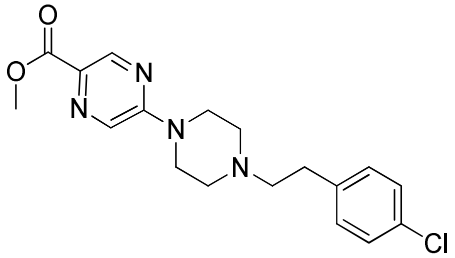

^{1}H NMR (400 MHz, DMSO-d_6) δ 8.63 (d, J = 1.3 Hz, 1H, Ar-***H*** of Pyrazine), 8.35 (d, J = 1.4 Hz, 1H, Ar-***H*** of Pyrazine), 7.31 (d, J = 8.5 Hz, 2H, Ar-***H***), 7.26 (d, J = 8.5 Hz, 2H, Ar-***H***), 3.79 (s, 3H, -OC$\boldsymbol{H}_3$), 3.73 – 3.62 (m, 4H, Piperazine ***H***s), 2.75 (dd, J = 8.9,

6.4 Hz, 2H, -N-CH_2CH$_2$-Ar), 2.59 – 2.48 (m, 6H, -N-CH$_2$CH_2-Ar + Piperazine ***H***s). MS m/z: 361.03 $[M+H]^+$,Yield: 61 %

Methyl 5-(4-(2-(1H-indol-3-yl)ethyl)piperazin-1-yl)pyrazine-2-carboxylate **(6c)**

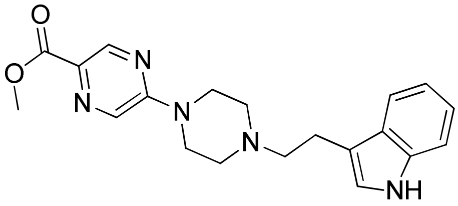

^{1}H NMR (400 MHz, DMSO-d_6) δ 10.75 (s, 1H, -N***H***), 8.64 (d, *J* = 1.3 Hz, 1H, Ar-***H*** of Pyrazine), 8.37 (d, *J* = 1.4 Hz, 1H, Ar-***H*** of Pyrazine), 7.51 (dd, *J* = 7.8, 1.1 Hz, 1H, Ar-***H*** of indole), 7.31 (dt, *J* = 8.1, 0.9 Hz, 1H, Ar-***H*** of indole), 7.15 (d, *J* = 2.3 Hz, 1H, Ar-***H*** of indole), 7.04 (ddd, *J* = 8.1, 7.0, 1.2 Hz, 1H, Ar-***H*** of indole), 6.95 (ddd, *J* = 7.9, 7.0, 1.1 Hz, 1H, Ar-***H*** of indole), 3.80 (s, 3H, -OCH_3), 3.77 – 3.69 (m, 4H, Piperazine ***H***s), 2.93 – 2.81 (m, 2H, -N-CH_2CH$_2$-Ar), 2.68 – 2.60 (m, 2H, -N-CH$_2$CH_2-Ar), 2.57 (t, *J* = 5.1 Hz, 4H, Piperazine ***H***s). MS m/z: 366.15 $[M+H]^+$, Yield: 54 %

Methyl 5-(4-(2-(1-methyl-1H-indol-3-yl)ethyl)piperazin-1-yl)pyrazine-2-carboxylate **(6d)**

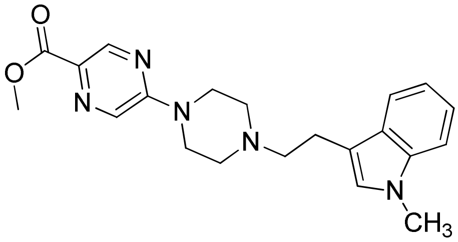

^{1}H NMR (400 MHz, DMSO-d_6) δ 8.64 (d, *J* = 1.3 Hz, 1H, Ar-***H*** of Pyrazine), 8.37 (d, *J* = 1.4 Hz, 1H, Ar-***H*** of Pyrazine), 7.52 (dt, *J* = 7.9, 1.0 Hz, 1H, Ar-***H*** of indole), 7.39 – 7.31 (m, 1H, Ar-***H*** of indole), 7.14 (s, 1H, Ar-***H*** of indole), 7.12 – 7.08 (m, 1H, Ar-

H of indole), 6.99 (ddd, *J* = 7.9, 7.0, 1.0 Hz, 1H, Ar-***H*** of indole), 3.80 (s, 3H, -OC$\boldsymbol{H}_3$), 3.75 (t, *J* = 5.1 Hz, 4H, Piperazine ***H***s), 3.71 (s, 3H, -N-C$\boldsymbol{H}_3$), 2.90 – 2.84 (m, 2H, -N-C$\boldsymbol{H}_2$$CH_2$-Ar), 2.66 – 2.60 (m, 2H, -N-CH_2C$\boldsymbol{H}_2$-Ar), 2.58 (t, *J* = 5.1 Hz, 4H, Piperazine ***H***s). MS m/z: 380.40 $[M+H]^+$, Yield: 58 %

General Procedure for the preparation of different carboxylic acid salts through the hydrolysis of corresponding esters

A mixture of the appropriate ester (**3a-c**, **5a-f**, **6a-d**) (1.0 mmol) and 1 N NaOH (2.5 mmol) in MeOH (20 ml) was stirred at 70° C for 3 h. The mixture was cooled and neutralized with 1 N HCl. The given solid was filtered to give the corresponding carboxylic acid salts. Spectral data of the given compounds were represented below.

1-(5-Carboxypyrazin-2-yl)-4-methylpiperazin-1-ium chloride **(4a)**

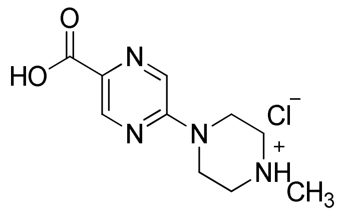

^{1}H NMR (400 MHz, DMSO-d_6) δ 12.84 (s, 1H, -COO***H***), 11.49 (s, 1H, Piperazinium ***H***), 8.68 (d, *J* = 1.3 Hz, 1H, Ar-***H*** of Pyrazine), 8.45 (d, *J* = 1.3 Hz, 1H, Ar-***H*** of Pyrazine), 4.59 (br. s, 2H, Piperazine ***H***s), 3.46 (br. s, 4H, Piperazine ***H***s), 3.09 (br. s, 2H, Piperazine ***H***s), 2.75 (s, 3H, -NC$\boldsymbol{H}_3$). Yield: 70 %

(5-Carboxypyrimidin-2-yl)-4-methylpiperazin-1-ium chloride (**4b**)

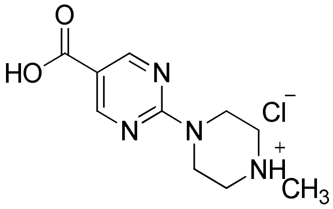

^{1}H NMR (400 MHz, DMSO-d_6) δ 12.99 (s, 1H, -COO***H***), 11.29 (s, 1H, Piperazinium ***H***), 8.82 (s, 2H, Ar-***H*** of Pyrimidine), 4.78 (s, 2H, Piperazine ***H***s), 3.55 – 3.00 (m, 6H, Piperazine ***H***s), 2.75 (s, 3H). MS m/z: 223.24 [M+H]$^+$. Yield: 85 %

4-(tert-Butoxycarbonyl)-1-(5-carboxypyrazin-2-yl)piperazin-1-ium chloride **(4c)**

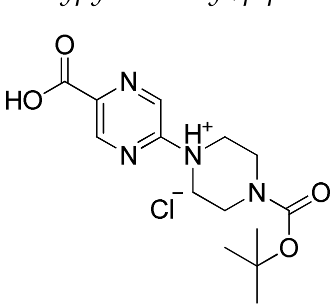

.

^{1}H NMR (400 MHz, DMSO-d_6) δ 12.70 (s, 1H, -COO***H***), 8.64 (d, *J* = 1.3 Hz, 1H, Ar-***H*** of Pyrazine), 8.34 (d, *J* = 1.4 Hz, 1H, Ar-***H*** of Pyrazine), 3.74 – 3.65 (m, 4H, Piperazine ***H***s), 3.51 – 3.41 (m, 4H, Piperazine ***H***s), 1.41 (s, 9H, -OC(C***H***$_3$)$_3$). MS m/z: 307.30 [M-H]$^-$. Yield: 88 %.

4-(5-Carboxypyrimidin-2-yl)-1-((1-methyl-1H-indol-3-yl)methyl)piperazin-1-ium chloride **(7a)**

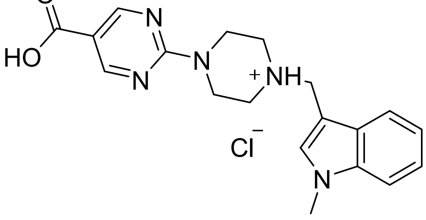

^{1}H NMR (400 MHz, D_2O) δ 8.72 (s, 2H, Ar-***H*** of pyrimidine), 7.65 (dt, *J* = 7.9, 1.0 Hz, 1H, Ar-***H*** of indole), 7.37 (dt, *J* = 8.3, 0.9 Hz, 1H, Ar-***H*** of indole), 7.22 (s, 1H, Ar-***H*** of indole), 7.12 (ddd, *J* = 8.2, 7.0, 1.2 Hz, 1H, Ar-***H*** of indole), 7.01 (ddd, *J* = 8.0, 7.0, 1.0 Hz, 1H, Ar-***H*** of indole), 3.81 (t, *J* = 5.1 Hz,, 4H, Piperazine ***H***s), 3.73 (s, 3H, -NC$\boldsymbol{H}_3$), 3.66 (s, 2H, N-C$\boldsymbol{H}_2$-Indole), 2.45 (t, *J* = 5.1 Hz, 4H, Piperazine ***H***s). MS m/z: 350.28 $[M-H]^-$. Yield: 55 %

4-(5-Carboxypyrimidin-2-yl)-1-(pyridin-2-ylmethyl)piperazin-1-ium chloride **(7b)**

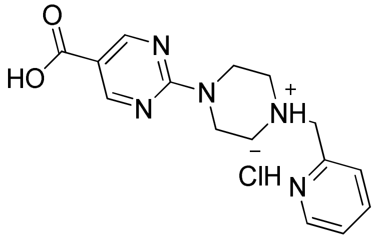

^{1}H NMR (400 MHz, DMSO-d_6) δ 12.97 (s, 1H, -COO***H***), 11.43 (s, 1H, Piperazinium ***H***), 8.81 (s, 2H, Ar-***H*** of Pyrimidine), 8.70 – 8.58 (m, 1H, Ar-***H*** of Pyridine), 7.92 (td, *J* = 7.7, 1.8 Hz, 1H, Ar-***H*** of Pyridine), 7.69 (dd, *J* = 7.8, 1.1 Hz, 1H, Ar-***H*** of Pyridine), 7.47 (ddd, *J* = 7.7, 4.8, 1.2 Hz, 1H, Ar-***H*** of Pyridine), 4.47 (s, 2H, N-C$\boldsymbol{H}_2$-Pyridine), 4.16 (br. s, 4H, Piperazine ***H***s), 3.35 – 3.32 (m, 4H, Piperazine ***H***s). MS m/z: 298.28 $[M-H]^-$, 300.18 $[M+H]^+$. Yield: 67 %

1-((1H-indol-3-yl)methyl)-4-(5-carboxypyrazin-2-yl)piperazin-1-ium Chloride **(7c)**

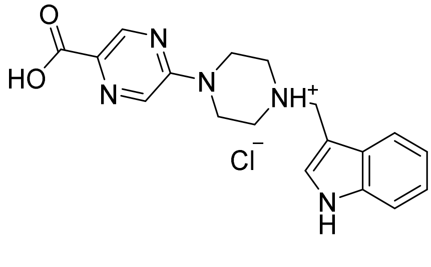

^{1}H NMR (400 MHz, DMSO-d_6) δ 11.24 (s, 1H, -N***H***), 8.63 (d, *J* = 1.3 Hz, 1H, Ar-***H*** of Pyrazine), 8.36 (d, *J* = 1.4 Hz, 1H, Ar-***H*** of Pyrazine), 7.72 (d, *J* = 8.0 Hz, 1H, Ar-***H*** of indole), 7.44 (s, 1H, Ar-***H*** of indole), 7.39 (d, *J* = 8.0 Hz, 1H, Ar-***H*** of indole), 7.10 (ddd, *J* = 8.2, 6.9, 1.2 Hz, 1H, Ar-***H*** of indole), 7.06 – 7.00 (m, 1H, Ar-***H*** of indole), 4.48 – 2.67 (m, 10H, -N-CH_2-Indole + Piperazine ***H***s overlapped with DMSO and H_2O). MS m/z: 336.23 $[M-H]^-$, 337.89 $[M+H]^+$, Yield: 60 %

4-(5-Carboxypyrazin-2-yl)-1-((1-methyl-1H-indol-3-yl)methyl)piperazin-1-ium chloride **(7d)**

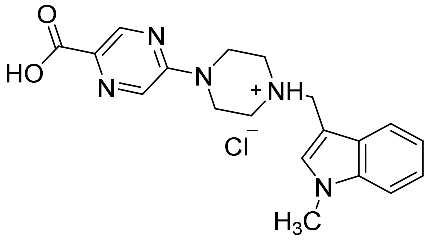

^{1}H NMR (400 MHz, DMSO-d_6) δ 12.81 (s, 1H, -COO***H***), 11.13 (s, 1H, -Piperazinium ***H***), 8.65 (d, *J* = 1.3 Hz, 1H, Ar-***H*** of Pyrazine), 8.41 (d, *J* = 1.4 Hz, 1H, Ar-***H*** of Pyrazine), 7.81 (d, *J* = 7.8 Hz, 1H, Ar-***H*** of indole), 7.61 (s, 1H, Ar-***H*** of indole), 7.48 (d, *J* = 8.2 Hz, 1H, Ar-***H*** of indole), 7.21 (ddd, *J* = 8.2, 7.0, 1.2 Hz, 1H, Ar-***H*** of indole), 7.13 (ddd, *J* = 8.0, 7.0, 1.0 Hz, 1H, Ar-***H*** of indole), 4.63 – 4.59 (m, 4H, Piperazine ***H***s), 4.47 (s, 2H, -N-CH_2-Indole), 3.81 (s, 3H, -NCH_3), 3.48 – 3.44 (m, 4H, Piperazine ***H***s). MS m/z: 350.41 $[M-H]^-$, Yield: 44 %.

4-(5-Carboxypyrazin-2-yl)-1-(pyridin-2-ylmethyl)piperazin-1-ium chloride **(7e)**

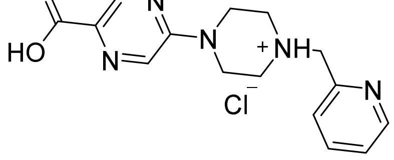

^{1}H NMR (400 MHz, DMSO-d_6) δ 12.82 (s, 1H, -COO***H***), 11.48 (s, 1H, -Piperazinium ***H***), 8.67 (d, *J* = 1.4 Hz, 1H, Ar-***H*** of Pyrazine), 8.66 – 8.64 (m, 1H, Ar-***H*** of Pyridine), 8.43 (d, *J* = 1.4 Hz, 1H, Ar-***H*** of Pyrazine), 7.93 (td, *J* = 7.7, 1.8 Hz, 1H, Ar-***H*** of Pyridine), 7.69 (dt, *J* = 7.9, 1.1 Hz, 1H, Ar-***H*** of Pyridine), 7.48 (ddd, *J* = 7.7, 4.9, 1.2 Hz, 1H, Ar-***H*** of Pyridine), 4.49 (s, 2H, N-C***H***$_2$-Pyridine), 4.08 – 4.05 (m, 4H, Piperazine ***H***s), 3.40 – 3.37 (m, 4H, Piperazine ***H***s). MS m/z: 298.22 [M-H]$^-$, 300.43 [M+H]$^+$. Yield: 44 %

1-(Benzo[b]thiophen-3-ylmethyl)-4-(5-carboxypyrazin-2-yl)piperazin-1-ium chloride **(7f)**

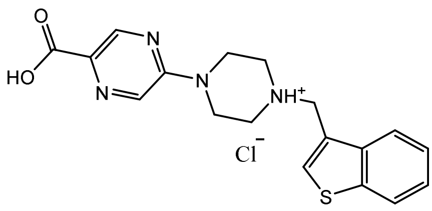

^{1}H NMR (400 MHz, DMSO-d_6) δ 12.81 (s, 1H, -COO***H***), 11.42 (s, 1H, Piperazinium ***H***), 8.67 (d, *J* = 1.3 Hz, 1H, Ar-***H*** of Pyrazine), 8.42 (s, 1H, Ar-***H*** of Pyrazine), 8.22 (s, 1H, Ar-***H*** of benzothiophene), 8.15 (d, *J* = 7.8 Hz, 1H, Ar-***H*** of benzothiophene), 8.06 (d, *J* = 7.8 Hz, 1H, Ar-***H*** of benzothiophene), 7.45 (dt, *J* = 18.2, 7.1 Hz, 2H, Ar-***H*** of benzothiophene), 4.62 (s, 2H, N-C***H***$_2$- benzothiophene), 3.49 – 3.45 (m, 4H, Piperazine ***H***s), 3.32 – 3.28 (m, 4H, Piperazine ***H***s). MS m/z: 354.23 [M-H]$^-$, 356.23 [M+H]$^+$. Yield: 88 %

4-(5-Carboxypyrazin-2-yl)-1-phenethylpiperazin-1-ium chloride **(7g)**

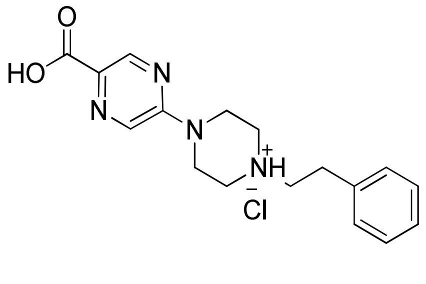

^{1}H NMR (400 MHz, DMSO-d_6) δ 12.85 (s, 1H, -COO***H***), 11.53 (s, 1H, Piperazinium ***H***), 8.69 (d, *J* = 1.3 Hz, 1H, Ar-***H*** of Pyrazine), 8.47 (d, *J* = 1.4 Hz, 1H, Ar-***H*** of Pyrazine), 7.33 (t, *J* = 7.5 Hz, 2H, Ar-***H***), 7.30 – 7.21 (m, 3H, Ar-***H***), 4.62 (d, *J* = 14.4 Hz, 2H, N-C$\boldsymbol{H}_2$$CH_2$-Ar), 3.65 (d, *J* = 12.1 Hz, 2H, N-CH_2C$\boldsymbol{H}_2$-Ar), 3.55 (t, *J* = 13.2 Hz, 4H, Piperazine ***H***s), 3.18 – 3.07 (m, 4H, Piperazine ***H***s). MS m/z: 311.22 $[M-H]^-$, 313.38 $[M+H]^+$. Yield: 86 %

4-(5-Carboxypyrazin-2-yl)-1-(4-chlorophenethyl)piperazin-1-ium chloride **(7h)**

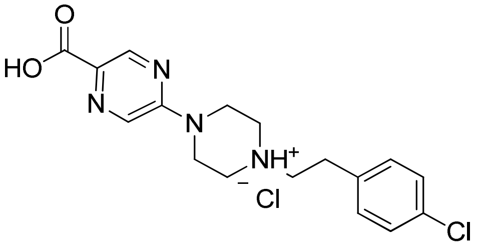

^{1}H NMR (400 MHz, DMSO-d_6) δ 12.84 (s, 1H, -COO***H***), 11.52 (s, 1H, Piperazinium ***H***), 8.68 (d, *J* = 1.3 Hz, 1H, Ar-***H*** of Pyrazine), 8.46 (d, *J* = 1.4 Hz, 1H, Ar-***H*** of Pyrazine), 7.39 (d, *J* = 8.4 Hz, 2H, Ar-***H***), 7.30 (d, *J* = 8.4 Hz, 2H, Ar-***H***), 4.61 (d, *J* = 14.2 Hz, 2H, N-C$\boldsymbol{H}_2$$CH_2$-Ar), 3.63 (d, *J* = 12.4 Hz, 2H, N-CH_2C$\boldsymbol{H}_2$-Ar), 3.54 (t, *J* = 13.2 Hz, 4H, Piperazine ***H***s), 3.18 – 3.03 (m, 4H, Piperazine ***H***s). MS m/z: 345.45 $[M-H]^-$, 347.37 $[M+H]^+$. Yield: 92 %

4-(5-Carboxypyrazin-2-yl)-1-(2-(1-methyl-1H-indol-3-yl)ethyl)piperazin-1-ium chloride **(7i)**

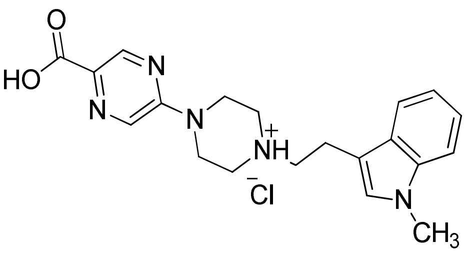

^{1}H NMR (400 MHz, DMSO-d_6) δ 12.84 (s, 1H, -COO***H***), 11.24 (s, 1H, Piperazinium ***H***), 8.70 (d, *J* = 1.3 Hz, 1H, Ar-***H*** of Pyrazine), 8.48 (d, *J* = 1.4 Hz, 1H, Ar-***H*** of Pyrazine), 7.64 (d, *J* = 7.9 Hz, 1H, Ar-***H*** of indole), 7.40 (d, *J* = 8.2 Hz, 1H, Ar-***H*** of indole), 7.21 (s, 1H, Ar-***H*** of indole), 7.16 (ddd, *J* = 8.2, 7.0, 1.2 Hz, 1H, Ar-***H*** of indole), 7.04 (ddd, *J* = 8.0, 6.9, 1.0 Hz, 1H, Ar-***H*** of indole), 4.62 (s, 3H, -NC$\boldsymbol{H}_3$), 3.63 – 3.43 (m, 6H, -N-C$\boldsymbol{H}_2$$CH_2$-Ar + Piperazine ***H***s), 3.23 – 3.12 (m, 6H, -N-CH_2C$\boldsymbol{H}_2$-Ar + Piperazine ***H***s). MS m/z: 364.35 $[M-H]^-$, 366.43 $[M+H]^+$. Yield: 92 %

1-(2-(1H-indol-3-yl)ethyl)-4-(5-carboxypyrazin-2-yl)piperazin-1-ium chlolride **(7j)**

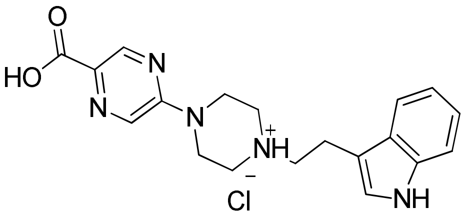

^{1}H NMR (400 MHz, D_2O) δ 8.41 (s, 1H, Ar-***H*** of Pyrazine), 7.96 (s, 1H, Ar-***H*** of Pyrazine), 7.44 (d, *J* = 7.7 Hz, 1H, Ar-***H*** of indole), 7.24 (d, *J* = 8.0 Hz, 1H, Ar-***H*** of indole), 6.97 (h, *J* = 7.2 Hz, 3H, Ar-***H*** of indole), 3.38 (t, *J* = 5.1 Hz, 4H, Piperazine ***H***s), 2.71 (dd, *J* = 10.2, 6.3 Hz, 2H, -N-C$\boldsymbol{H}_2$$CH_2$-Ar), 2.41 (dd, *J* = 10.2, 6.3 Hz, 2H, -N-CH_2C$\boldsymbol{H}_2$-Ar), 2.29 (t, *J* = 5.1 Hz, 4H, Piperazine ***H***s). MS m/z: 350.44 $[M-H]^-$. Yield: 90 %

*General Procedure for the synthesis of tert-butyl (substituted-2-nitrophenyl) carbamate (**11a-e**) and **14**.*

The corresponding 2-nitroaniline (4.61 mmol) was dissolved in dry $CHCl_3$ (15 mL), and then DIPEA (13.82 mmol) was added followed by addition of $(Boc)_2O$ (9.22 mmol) and DMAP (0.50 mmol) dissolved in 5 mL dry $CHCl_3$. The

reaction mixture was stirred overnight at RT. The solution was washed with 1 M HCl (30 mL). The organic layer was separated, dried with Na_2SO_4, filtered and concentrated under vacuum. The residue was purified by MPLC (n-Hexane: EtOAc) to afford the corresponding boc-protected nitroaniline as yellow solids. Spectrometric data and reaction Yield:of compounds **11a,c,d** were found as reported. [1, 2]

tert-Butyl 5-methoxy-2-nitrophenylcarbamate **(11b)**

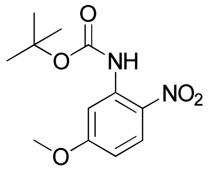

^{1}H NMR (400 MHz, DMSO-d_6) δ 9.72 (s, 1H, -CON***H***), 8.06 (d, *J* = 9.3 Hz, 1H, Ar-***H***), 7.58 (d, *J* = 2.7 Hz, 1H, Ar-***H***), 6.78 (dd, *J* = 9.4, 2.7 Hz, 1H, Ar-***H***), 3.85 (s, 3H, -OC$\boldsymbol{H}_3$), 1.46 (s, 9H, -OC(C$\boldsymbol{H}_3$)$_3$), Yield:66 %

tert-Butyl 4-fluoro-2-nitrophenylcarbamate **(11e)**

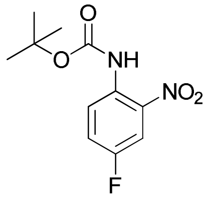

^{1}H NMR (400 MHz, DMSO-d_6) δ 9.54 (s, 1H, -CON***H***), 7.86 (dt, *J* = 13.8, 5.7 Hz, 1H, Ar-***H***), 7.66 – 7.48 (m, 2H, Ar-***H***), 1.42 (s, 9H, OC(C$\boldsymbol{H}_3$)$_3$), Yield:55 %

tert- Butyl (4-Bromo-2-nitrophenyl) carbamate **(14)**

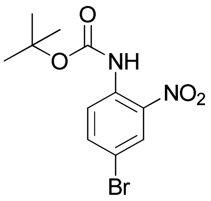

^{1}H NMR (400 MHz, DMSO-d_6) δ 9.64 (s, 1H, -CON***H***), 8.43 (d, *J* = 2.2 Hz, 1H, Ar-***H***), 8.11 (dd, *J* = 8.8, 2.5 Hz, 1H, Ar-***H***), 7.64 (d, *J* = 8.3 Hz, 1H, Ar-***H***), 1.35 (s, 9H, -OC(C$\boldsymbol{H}_3$)$_3$). MS m/z: 315.17 $[M-H]^-$, Yield: 80 %.

General procedure for C-C coupling using Suzuki reaction

tert- Butyl (4-Bromo-2-nitrophenyl) carbamate (**14**) (1.26 mmol) and the corresponding boronic acid were dissolved in degassed 1,1 dimethoxyethane (DME) followed by the addition of tetrakis Pd[P(Ph$_3$)$_4$] (0.40 g, 1.26 mmol). Degassed 3M aqueous Na_2CO_3 (1.25 mL) was added into the mixture then it was degassed for 10 min by sparging with argon gas. The reaction mixture was heated at 90 ^{0} C for 8h under argon atmosphere. The solvent was evaporated followed by addition of ethyl acetate, washed with brine, dried over anhydrous Na_2SO_4 and finally purified by MPLC (n-Hexane/EtOAc) to obtain the corresponding products (**16a-d**). Spectrometric data and reaction Yield:of compounds **16a, c**, were found as reported.[3, 4]

tert-Butyl [2-nitro-4-(thiophen-3-yl)phenyl]carbamate **(16b)**

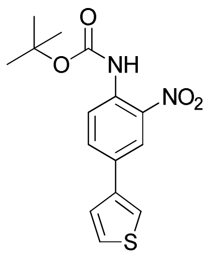

^{1}H NMR (400 MHz, DMSO-d_6) δ 9.57 (s, 1H, -CON***H***), 8.23 (d, *J* = 2.0 Hz, 1H, Ar-***H***), 8.01 (dd, *J* = 8.4, 2.2 Hz, 2H, Ar-***H***), 7.64 (m, 3H, Ar-***H***), 1.44 (s, 9H, OC(CH_3)$_3$). MS m/z: 319.29 [M-H]$^-$, Yield: 70 %

tert-butyl (2′-Fluoro-3-nitro-biphenyl-4-yl)-carbamate **(16d)**

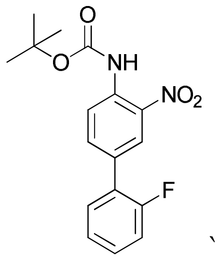

^{1}H NMR (500 MHz, DMSO-d_6) δ 9.69 (s, 1H, -CON***H***), 8.10 (s, 1H, Ar-***H***), 7.91 – 7.84 (m, 1H, - Ar-***H***), 7.76 (d, *J* = 8.6 Hz, 1H, Ar-***H***), 7.65 – 7.56 (m, 1H, Ar-***H***), 7.51 – 7.45 (m, 1H, Ar-***H***), 7.37 – 7.27 (m, 2H, Ar-***H***), 1.45 (s, 9H, OC(CH_3)$_3$), Yield: 70 %

General Procedure for the reduction of nitro compounds **(9a, 12a-e, 17a-d)**

A mixture of the appropriate nitro compound (1.0 g, 1.0 eq.) and ammonium formate (4.0 eq.) were dissolved in MeOH (30.0 mL) and stirred at RT with 10% Pd/C (0.2 - 0.3 g). The progress of reaction was monitored by TLC which indicated the disappearance of the starting material. The catalyst was filtered off through celite and washed with MeOH, and the filtrate was collected and concentrated under vacuum. The obtained residue was diluted with partitioned between water (50.0 mL) and ethyl acetate (50.0 mL), the organic layer was separated, evaporated under reduced pressure to give the corresponding amine which was used without further purification.[1] Spectrometric data and reaction Yield of compounds **12a**, **12c**, **12d**, **17a** and **17c** were found as reported.[2-5]

tert-Butyl 2-amino-5-methoxyphenylcarbamate **(12b)**

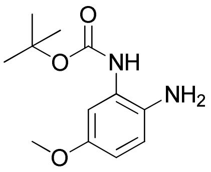

^{1}H NMR (400 MHz, DMSO-d_6) δ 8.25 (s, 1H, -CON***H***), 6.91 (s, 1H, Ar-***H***), 6.61 (d, *J* = 8.6 Hz, 1H, Ar-***H***), 6.50 – 6.40 (m, 1H, Ar-***H***), 4.41 (s, 2H, -N$\boldsymbol{H}_2$), 3.60 (s, 3H, -OC$\boldsymbol{H}_3$), 1.43 (s, 9H, OC(C$\boldsymbol{H}_3$)$_3$), Yield: 84 %

tert-Butyl 2-amino-4-fluorophenylcarbamate **(12e)**

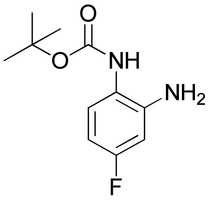

^{1}H NMR (400 MHz, DMSO-d_6) δ 8.19 (s, 1H, -CON***H***), 7.12 – 7.03 (m, 1H, Ar-***H***), 6.44 (dd, *J* = 11.2, 2.9 Hz, 1H, Ar-***H***), 6.27 (td, *J* = 8.6, 2.9 Hz, 1H, Ar-***H***), 5.09 (s, 2H), 1.43 (s, 9H, OC(C$\boldsymbol{H}_3$)$_3$). MS m/z: 249.16 $[M+Na]^+$, Yield: 82 %.

tert-Butyl [2-amino-4-(thien-3-yl)phenyl]carbamate **(17b)**

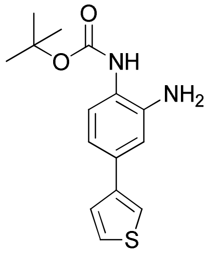

^1H NMR (400 MHz, DMSO-d_6) δ 8.48 (s, 1H, -CON***H***), 7.89 – 7.16 (m, 6H, Ar-***H***), 5.06 (s, 2H, -N$\boldsymbol{H}_2$), 1.63 (s, 9H, OC(C$\boldsymbol{H}_3$)$_3$), Yield:75 %.

tert-Butyl (3-Amino-2′-fluorobiphenyl-4-yl)carbamate **(17d)**

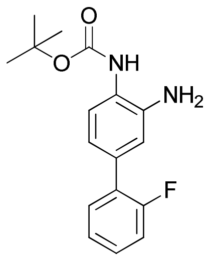

^1H NMR (400 MHz, DMSO-d_6) δ 8.35 (s, 1H, -CON***H***), 7.46 – 7.19 (m, 5H, Ar-***H***), 6.88 (t, *J* = 1.8 Hz, 1H, Ar-***H***), 6.69 (ddd, *J* = 7.0, 4.4, 2.6 Hz, 1H, Ar-***H***), 4.96 (s, 2H, -N$\boldsymbol{H}_2$), 1.46 (s, 9H, OC(C$\boldsymbol{H}_3$)$_3$), Yield: 90 %

Spectral data of boc protected pre-final compounds

tert-Butyl (2-(5-(4-(2-(1-methyl-1H-indol-3-yl)ethyl)piperazin-1-yl)pyrazine-2-carboxamido)-4-(thiophen-2-yl)phenyl)carbamate **(20a)**

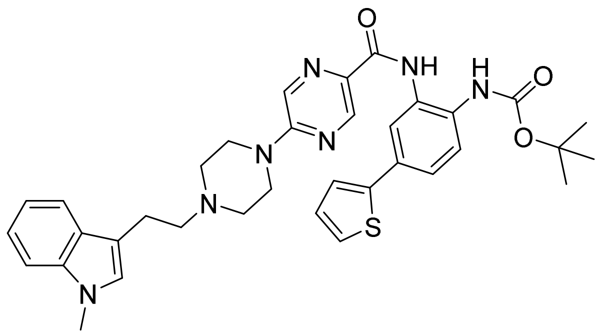

^1H NMR (400 MHz, DMSO-d_6) δ 10.02 (s, 1H, -CO-N***H***-Ar), 9.06 (s, 1H, -CO-N***H***-Ar), 8.75 (d, *J* = 1.3 Hz, 1H, Ar-***H*** of Pyrazine), 8.27-8.26 (m, 2H, Ar-***H***s), 7.55 – 7.53 (m, 1H, Ar-***H***), 7.53 (d, *J* = 1.1 Hz, 1H, Ar-***H***), 7.46 – 7.44 (m, 1H, Ar-***H***), 7.42 (d, *J* = 2.2 Hz, 1H, Ar-***H***), 7.36 (dt, *J* = 8.3, 0.9 Hz, 1H, Ar-***H***), 7.30 (d, *J* = 8.3 Hz, 1H, Ar-***H***), 7.16 – 7.08 (m, 3H, Ar-***H***), 7.00 (ddd, *J* = 7.9, 7.0, 1.0 Hz, 1H, Ar-***H***), 3.76 (t, *J*

= 5.1 Hz, 4H, Piperazine ***H***s), 3.72 (s, 3H, -NC***H***$_3$), 2.93 – 2.83 (m, 2H, -N-C***H***$_2$CH$_2$-Ar), 2.71 – 2.56 (m, 6H, -N-CH$_2$C***H***$_2$-Ar + Piperazine ***H***s), 1.48 (s, 9H, -OC(C***H***$_3$)$_3$). MS m/z: 636.24 [M-H]$^-$, 638.33 [M+H]$^+$, Yield: 68 %

tert-Butyl (2-(5-(4-(2-(1H-indol-3-yl)ethyl)piperazin-1-yl)pyrazine-2-carboxamido)-5-(4-fluorophenyl)phenyl)carbamate **(20b)**

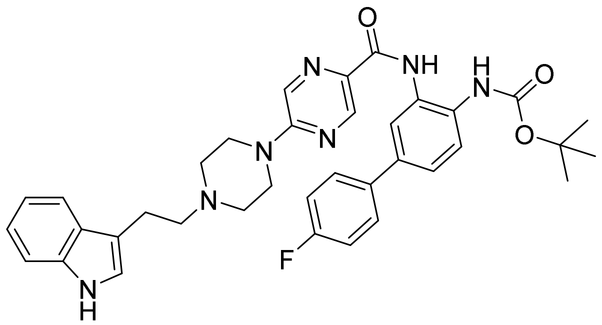

^{1}H NMR (400 MHz, DMSO-d_6) δ 10.76 (s, 1H, -N***H*** of indole), 10.05 (s, 1H, CO-N***H***-Ar), 9.08 (s, 1H, CO-N***H***-Ar), 8.74 (d, *J* = 1.3 Hz, 1H, Ar-***H*** of Pyrazine), 8.29 – 8.23 (d, 1H, Ar-***H*** of Pyrazine), 7.69 –7.65 (m, 2H, Ar-***H***), 7.52 (d, *J* = 7.9 Hz, 1H, Ar-***H***), 7.42 – 7.27 (m, 6H, Ar-***H***), 7.16 – 6.95 (m, 3H, Ar-***H***), 3.80 – 3.75 (m, 4H, Piperazine ***H***s), 2.95 – 2.58 (m, 8H, -N-C***H***$_2$C***H***$_2$-Ar + Piperazine ***H***s), 1.49 (s, 9H, -OC(C***H***$_3$)$_3$). MS m/z: 634.30 [M-H]$^-$, Yield: 75 %.

tert-Butyl (2-(5-(4-(2-(1H-indol-3-yl)ethyl)piperazin-1-yl)pyrazine-2-carboxamido)-5-(2-fluorophenyl)phenyl)carbamate **(20c)**

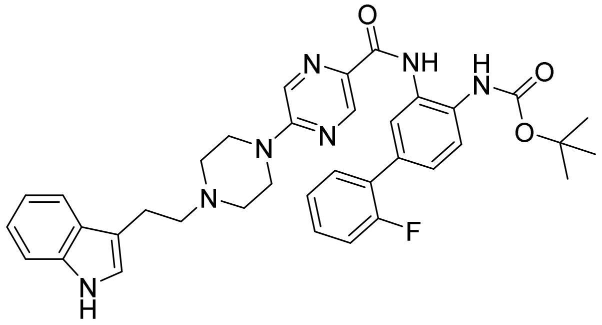

^{1}H NMR (400 MHz, DMSO-d_6) δ 11.97 (s, 1H, -N***H*** of indole), 10.76 (s, 1H, CO-N***H***-Ar), 10.05 (s, 1H, CO-N***H***-Ar), 8.72 (s, 1H, Ar-***H*** of Pyrazine), 8.27 (s, 1H, Ar-***H*** of Pyrazine), 6.94 – 7.69 (m, 12H, Ar-***H***), 3.75 – 3.62 (m, 4H, Piperazine ***H***s), 2.96 – 2.85 (m, 2H, -N-C$\boldsymbol{H_2}$CH$_2$-Ar), 2.65 – 2.58 (m, 6H, -N-CH$_2$C$\boldsymbol{H_2}$-Ar + Piperazine ***H***s), 1.49 (s, 9H, -OC(C$\boldsymbol{H_3}$)$_3$). MS m/z: 634.39 [M-H]$^-$. Yield: 66 %

tert-Butyl (4-fluoro-2-(5-(4-((1-methyl-1H-indol-3-yl)methyl)piperazin-1-yl)pyrazine-2-carboxamido)phenyl)carbamate **(22b)**

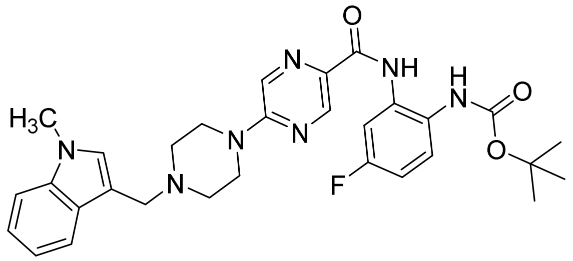

^{1}H NMR (400 MHz, DMSO-d_6) δ 9.96 (s, 1H, -CO-N***H***-Ar), 8.99 (s, 1H, -CO-N***H***-Ar), 8.71 (d, *J* = 1.3 Hz, 1H, Ar-***H*** of Pyrazine), 8.21 (d, *J* = 1.4 Hz, 1H, Ar-***H*** of Pyrazine), 7.94 (dd, *J* = 11.3, 3.0 Hz, 1H, Ar-***H***), 7.66 (dt, *J* = 7.9, 1.0 Hz, 1H, Ar-***H***), 7.38 (d, *J* = 8.2 Hz, 1H, Ar-***H***), 7.29 – 7.20 (m, 2H, Ar-***H***), 7.13 (ddd, *J* = 8.2, 7.0, 1.2 Hz, 1H, Ar-***H***), 7.02 (ddd, *J* = 8.0, 6.9, 1.0 Hz, 1H, Ar-***H***), 6.95 (td, *J* = 8.4, 3.0 Hz, 1H, Ar-***H***), 3.75 (s, 3H, -NC$\boldsymbol{H_3}$), 3.71 (t, *J* = 5.2 Hz, 4H, Piperazine ***H***s), 3.68 (s, 2H, -N-C$\boldsymbol{H_2}$-Indole), 2.54 – 2.50 (m, 4H, Piperazine ***H***s) 1.45 (s, 9H, -OC(C$\boldsymbol{H_3}$)$_3$). MS m/z: 558.30 [M-H]$^-$, Yield: 73 %.

tert-Butyl (4-fluoro-2-(5-(4-(2-(1-methyl-1H-indol-3-yl)ethyl)piperazin-1-yl)pyrazine-2-carboxamido)phenyl)carbamate **(22c)**

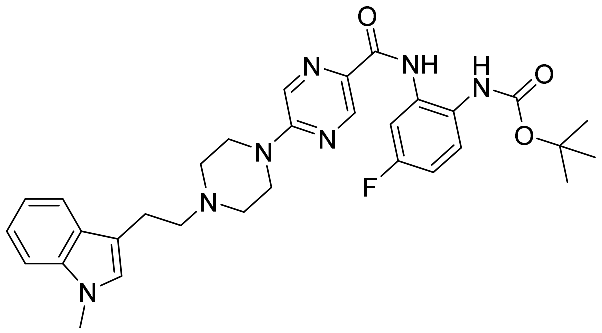

^{1}H NMR (400 MHz, DMSO-d_6) δ 9.98 (s, 1H, -CO-N***H***-Ar), 9.01 (s, 1H, -CO-N***H***-Ar), 8.74 (d, *J* = 1.3 Hz, 1H, Ar-***H*** of Pyrazine), 8.25 (d, *J* = 1.4 Hz, 1H, Ar-***H*** of Pyrazine), 7.96 (dd, *J* = 11.3, 3.3 Hz, 1H, Ar-***H***), 7.53 (dt, *J* = 7.9, 1.0 Hz, 1H, Ar-***H***), 7.38 – 7.34 (m, 1H, Ar-***H***), 7.23 (dd, *J* = 8.9, 6.1 Hz, 1H, Ar-***H***), 7.14 (s, 1H, Ar-***H***), 7.13 – 7.09 (m, 1H, Ar-***H***), 7.00 (ddd, *J* = 7.9, 6.9, 1.0 Hz, 1H, Ar-***H***), 6.97 – 6.92 (m, 1H, Ar-***H***), 3.76 (t, *J* = 5.0 Hz, 4H, Piperazine ***H***s), 3.72 (s, 3H, -NC$\boldsymbol{H}_3$), 2.92 – 2.83 (m, 2H, -N-C$\boldsymbol{H}_2$$CH_2$-Ar), 2.64 (dd, *J* = 9.0, 6.7 Hz, 2H, -N-CH_2C$\boldsymbol{H}_2$-Ar), 2.59 (t, *J* = 5.1 Hz, 4H, Piperazine ***H***s), 1.47 (s, 9H, -OC(C$\boldsymbol{H}_3$)$_3$). MS m/z: 572.28 $[M-H]^-$, 574.40 $[M+H]^+$,Yield: 70 % .

tert-Butyl 4-(5-(2-amino-4-chlorophenylcarbamoyl)pyrazin-2-yl)piperazine-1-carboxylate **(24a)**

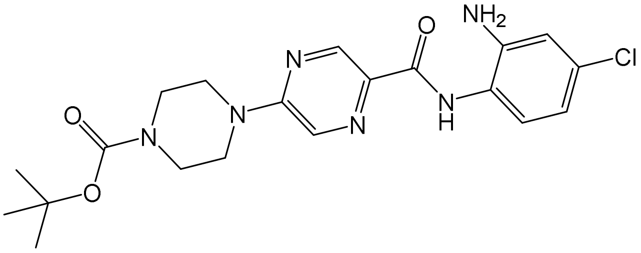

^{1}H NMR (400 MHz, DMSO-d_6) δ 9.61 (s, 1H, -CO-N***H***-Ar), 8.70 (d, *J* = 1.3 Hz, 1H, Ar-***H*** of Pyrazine), 8.33 (d, *J* = 1.3 Hz, 1H, Ar-***H*** of Pyrazine), 7.40 (d, *J* = 8.5 Hz, 1H, Ar-***H***), 6.83 (d, *J* = 2.4 Hz, 1H, Ar-***H***), 6.62 (dd, *J* = 8.4, 2.4 Hz, 1H, Ar-***H***), 5.16 (s, 2H, -N$\boldsymbol{H}_2$), 3.73 – 3.68 (m, 4H, Piperazine ***H***s), 3.53 – 3.37 (m, 4H, Piperazine ***H***s), 1.42 (s, 9H, -OC(C$\boldsymbol{H}_3$)$_3$). Yield: 71 %

tert-Butyl 4-(5-(2-amino-4,5-difluorophenylcarbamoyl)pyrazin-2-yl)piperazine-1-carboxylate **(24b)**

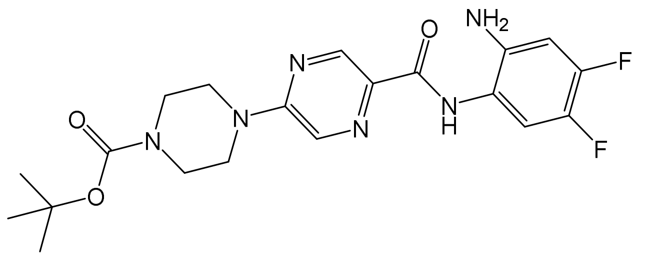

^{1}H NMR (400 MHz, DMSO-d_6) δ 9.65 (s, 1H, CO-N***H***-Ar), 8.70 (d, *J* = 1.3 Hz, 1H, Ar-***H*** of Pyrazine), 8.33 (d, *J* = 1.3 Hz, 1H, Ar-***H*** of Pyrazine), 7.54 (dd, *J* = 12.4, 8.7 Hz, 1H, Ar-***H***), 6.77 (dd, *J* = 12.8, 8.2 Hz, 1H, Ar-***H***), 5.02 (s, 2H, N***H***₂), 3.74 – 3.72 (m, 4H, Piperazine ***H***s), 3.38 – 3.52 (m, 4H, Piperazine ***H***s), 1.41 (s, 9H, -OC(C***H***₃)₃). MS m/z: 433.41 [M-H]⁻. Yield: 69 %

tert-Butyl 4-(5-((2-((tert-butoxycarbonyl)amino)-5-(trifluoromethyl)phenyl)carbamoyl)pyrazin-2-yl)piperazine-1-carboxylate **(26a)**

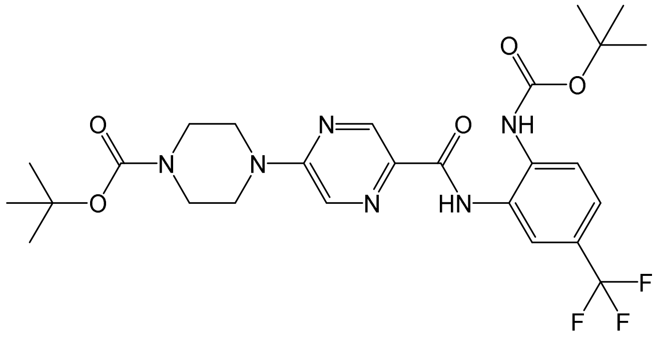

^{1}H NMR (500 MHz, DMSO-d_6) δ 10.13 (s, 1H, CO-N***H***-Ar), 9.33 (s, 1H, CO-N***H***-Ar), 8.74 (d, *J* = 1.3 Hz, 1H, Ar-***H*** of Pyrazine), 8.29 – 8.26 (m, 2H, Ar-***H*** of Pyrazine + Ar-***H***), 7.55 (d, *J* = 8.4 Hz, 1H, Ar-***H***), 7.48 (dd, *J* = 8.5, 1.8 Hz, 1H, Ar-***H***), 3.74 – 3.64 (m, 4H, Piperazine ***H***s), 3.52 – 3.40 (m, 4H, Piperazine ***H***s), 1.48 (s, 9H, -OC(C***H***₃)₃), 1.42 (s, 9H, -OC(C***H***₃)₃). MS m/z: 565.30 [M-H]⁻. Yield: 70 %

tert-Butyl 4-(5-((2-((tert-butoxycarbonyl)amino)-4-methylphenyl)carbamoyl)pyrazin-2-yl)piperazine-1-carboxylate **(26b)**

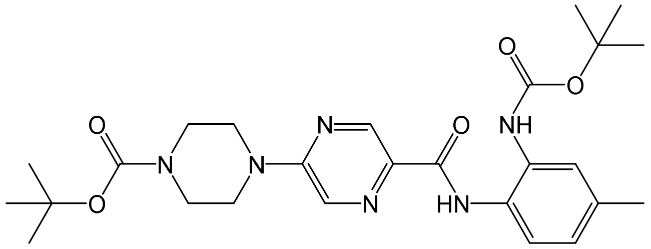

^{1}H NMR (400 MHz, DMSO-d_6) δ 9.89 (s, 1H, CO-N***H***-Ar), 8.81 (s, 1H, CO-N***H***-Ar), 8.71 (s, 1H, Ar-***H*** of Pyrazine), 8.23 (s, 1H, Ar-***H*** of Pyrazine), 7.77 (d, *J* = 8.2 Hz, 1H, Ar-***H***), 7.06 (s, 1H, Ar-***H***), 6.99 (d, *J* = 8.2 Hz, 1H, Ar-***H***), 3.72 – 3.55 (m, 4H, Piperazine ***H***s), 3.52 – 3.38 (m, 4H, Piperazine ***H***s), 2.26 (s, 3H, C$\boldsymbol{H}_3$), 1.44 (s, 9H, -OC(C$\boldsymbol{H}_3$)$_3$), 1.41 (s, 9H, -OC(C$\boldsymbol{H}_3$)$_3$). MS m/z: 511.19 [M-H]$^-$. Yield: 72 %.

tert-Butyl 4-(5-((2-((tert-butoxycarbonyl)amino)-4-methoxyphenyl)carbamoyl)pyrazin-2-yl)piperazine-1-carboxylate **(26c)**

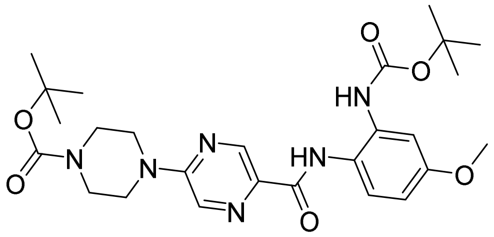

^{1}H NMR (400 MHz, DMSO-d_6) δ 9.85 (s, 1H, CO-N***H***-Ar), 8.92 (s, 1H, CO-N***H***-Ar), 8.70 (s, 1H, Ar-***H*** of Pyrazine), 8.23 (s, 1H, Ar-***H*** of Pyrazine), 7.67 (d, *J* = 8.9 Hz, 1H, Ar-***H***), 6.88 (d, *J* = 2.9 Hz, 1H, Ar-***H***), 6.78 (dd, *J* = 8.9, 2.9 Hz, 1H, Ar-***H***), 3.79 – 3.70 (m, 7H, Piperazine ***H***s + OC$\boldsymbol{H}_3$), 3.54 – 3.33 (m, 4H, Piperazine ***H***s), 1.45 (s, 9H, -OC(C$\boldsymbol{H}_3$)$_3$), 1.43 (s, 9H, -OC(C$\boldsymbol{H}_3$)$_3$). MS m/z: 527.28 [M-H]$^-$. Yield: 62 %.

tert-Butyl 2-(5-(4-methylpiperazin-1-yl)pyrazine-2-carboxamido)-4-(thiophen-3-yl)phenylcarbamate **(28a)**

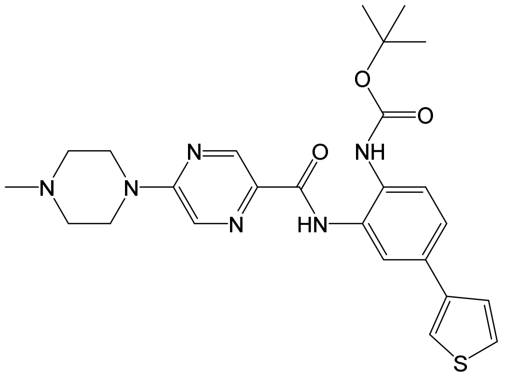

^{1}H NMR (400 MHz, DMSO-d_6) δ 10.02 (s, 1H, CO-N***H***-Ar), 9.02 (s, 1H, CO-N***H***-Ar), 8.73 (d, *J* = 1.3 Hz, 1H, Ar-***H*** of Pyrazine), 8.25 – 8.23 (m, 2H, Ar-***H*** of Pyrazine + Ar-***H***), 7.79 (dd, *J* = 2.9, 1.3 Hz, 1H, Ar-***H***), 7.63 (dd, *J* = 5.0, 2.9 Hz, 1H, Ar-***H***), 7.52 – 7.43 (m, 2H, Ar-***H***), 7.30 (d, *J* = 8.3 Hz, 1H, Ar-***H***), 3.79 – 3.65 (m, 4H, Piperazine ***H***s), 2.49 – 2.35 (m, 4H, Piperazine ***H***s), 2.22 (s, 3H, -NC$\boldsymbol{H}_3$), 1.47 (s, 9H, -OC(C$\boldsymbol{H}_3$)$_3$). MS m/z: 493.18 $[M+H]^+$. Yield: 70 %

tert-Butyl 4-(5-((2-((tert-butoxycarbonyl)amino)-5-(thiophen-2-yl)phenyl)carbamoyl)pyrazin-2-yl)piperazine-1-carboxylate. **(28b)**

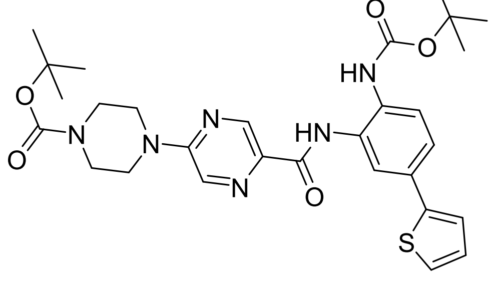

^{1}H NMR (400 MHz, DMSO-d_6) δ 10.04 (s, 1H, -CO-N***H***-Ar), 9.06 (s, 1H, -CO-N***H***-Ar), 8.76 (d, *J* = 1.3 Hz, 1H, Ar-***H*** of Pyrazine), 8.34 – 8.20 (m, 2H, Ar-***H*** + Ar-***H*** of Pyrazine), 7.53 (dd, *J* = 5.1, 1.1 Hz, 1H, Ar-***H***), 7.47 – 7.41 (m, 2H, Ar-***H***), 7.30 (d, *J*

= 8.4 Hz, 1H, Ar-***H***), 7.13 (dd, *J* = 5.1, 3.6 Hz, 1H, Ar-***H***), 3.80 – 3.69 (m, 4H, Piperazine ***H***s), 3.51 – 3.39 (m, 4H, Piperazine ***H***s), 1.48 (s, 9H, -OC(C$\boldsymbol{H}_3$)$_3$), 1.42 (s, 9H, -OC(C$\boldsymbol{H}_3$)$_3$). MS m/z: 579.09 [M-H]$^-$. Yield: 66 %.

tert-Butyl 4-(5-((4-((tert-butoxycarbonyl)amino)-4′-fluoro-[1,1′-biphenyl]-3-yl)carbamoyl)pyrazin-2-yl)piperazine-1-carboxylate **(28c)**

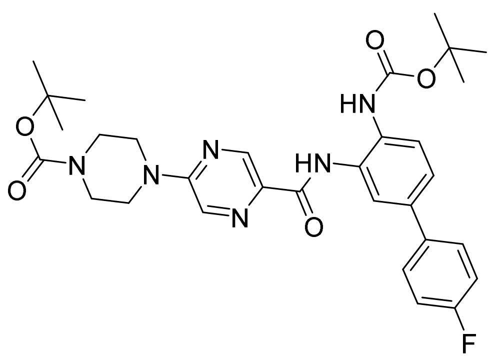

^{1}H NMR (400 MHz, DMSO-*d*$_6$) δ 10.06 (s, 1H, CO-N***H***-Ar), 9.07 (s, 1H, CO-N***H***-Ar), 8.74 (s, 1H, Ar-***H*** of Pyrazine), 8.32 – 8.22 (m, 2H, Ar-***H*** of Pyrazine + Ar-***H***), 7.71 – 7.62 (m, 2H, Ar-***H***), 7.45 – 7.23 (m, 4H, Ar-***H***), 3.75 – 3.72 (m, 4H, Piperazine ***H***s), 3.54 – 3.34 (m, 4H, Piperazine ***H***s), 1.48 (s, 9H, -OC(C$\boldsymbol{H}_3$)$_3$), 1.42 (s, 9H, -OC(C$\boldsymbol{H}_3$)$_3$). MS m/z: 591.29 [M-H]$^-$. Yield: 45 %.

tert-Butyl (2′-fluoro-3-(5-(4-methylpiperazin-1-yl)pyrazine-2-carboxamido)-[1,1′-biphenyl]-4-yl)carbamate **(28d)**

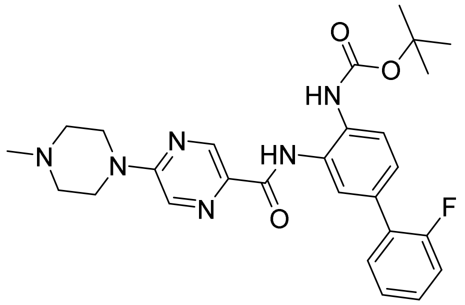

^{1}H NMR (400 MHz, DMSO-*d*$_6$) δ 10.05 (s, 1H, CO-N***H***-Ar), 9.12 (s, 1H, CO-N***H***-Ar), 8.72 (s, 1H, Ar-***H*** of Pyrazine), 8.26 (s, 1H, Ar-***H*** of Pyrazine), 8.15 (s, 1H, Ar-

H), 7.54 – 7.27 (m, 6H, Ar-***H***), 3.79 – 3.74 (m, 4H, Piperazine ***H***s), 2.50 – 2.45 (m, 4H, Piperazine ***H***s overlapped with DMSO), 2.27 (s, 3H, C$\boldsymbol{H}_3$), 1.49 (s, 9H, -OC(C$\boldsymbol{H}_3$)$_3$). MS m/z: 505.20 $[M-H]^-$. Yield: 60 %.

3. ^{1}HNMR charts of final compounds

^{1}H NMR chart of compound **19a**

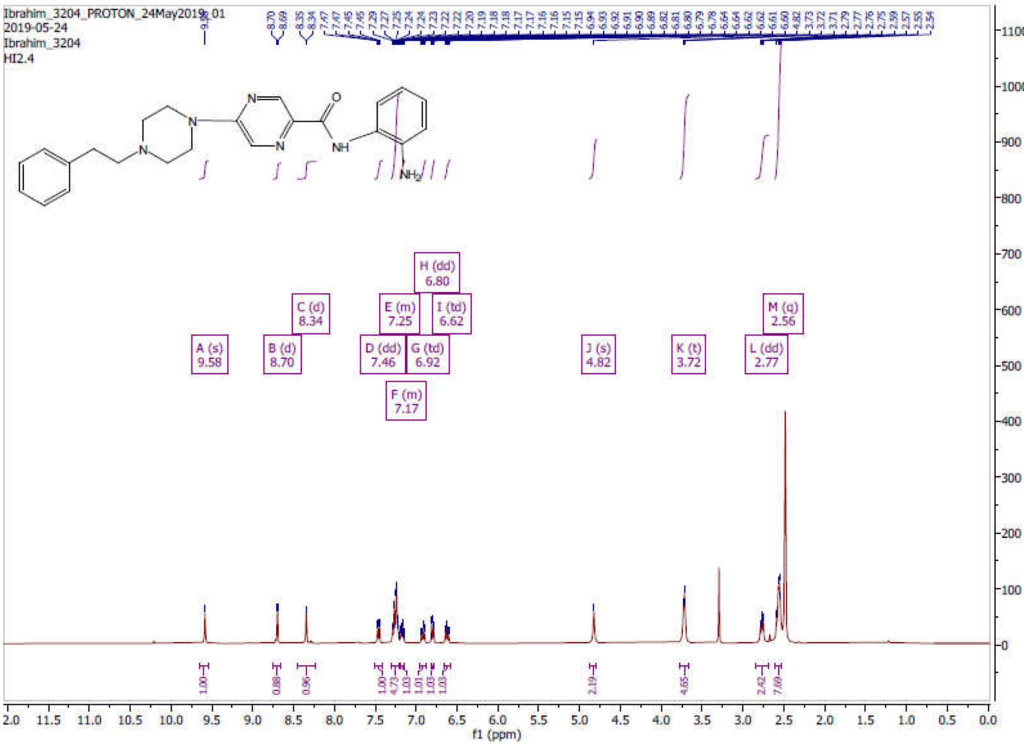

^{1}H *NMR chart of compound* **19b**

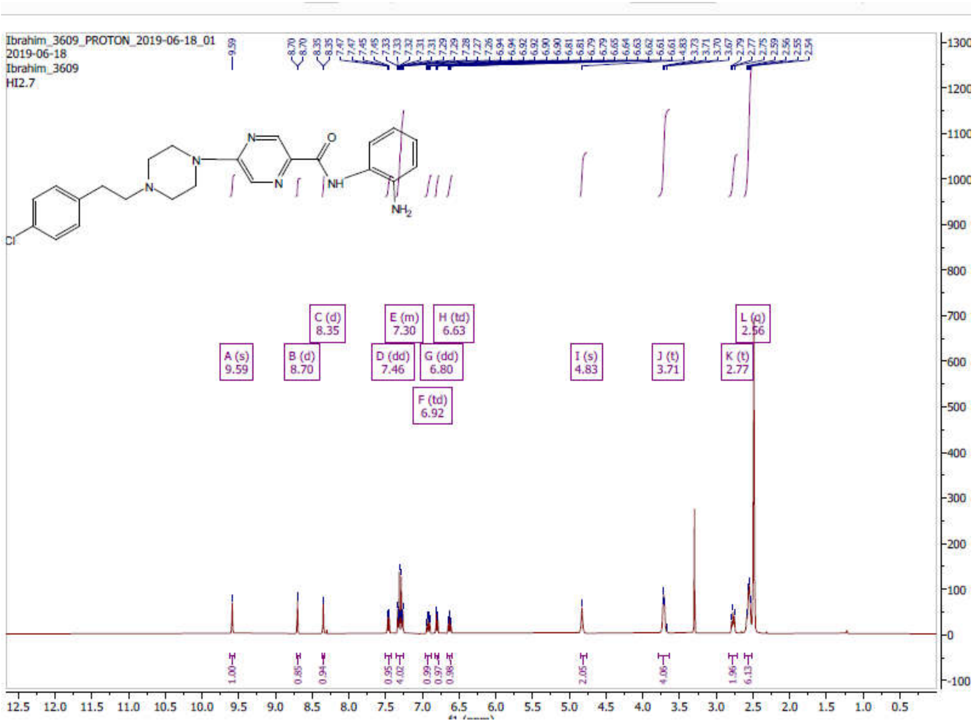

^{1}H *NMR chart of compound* **19c**

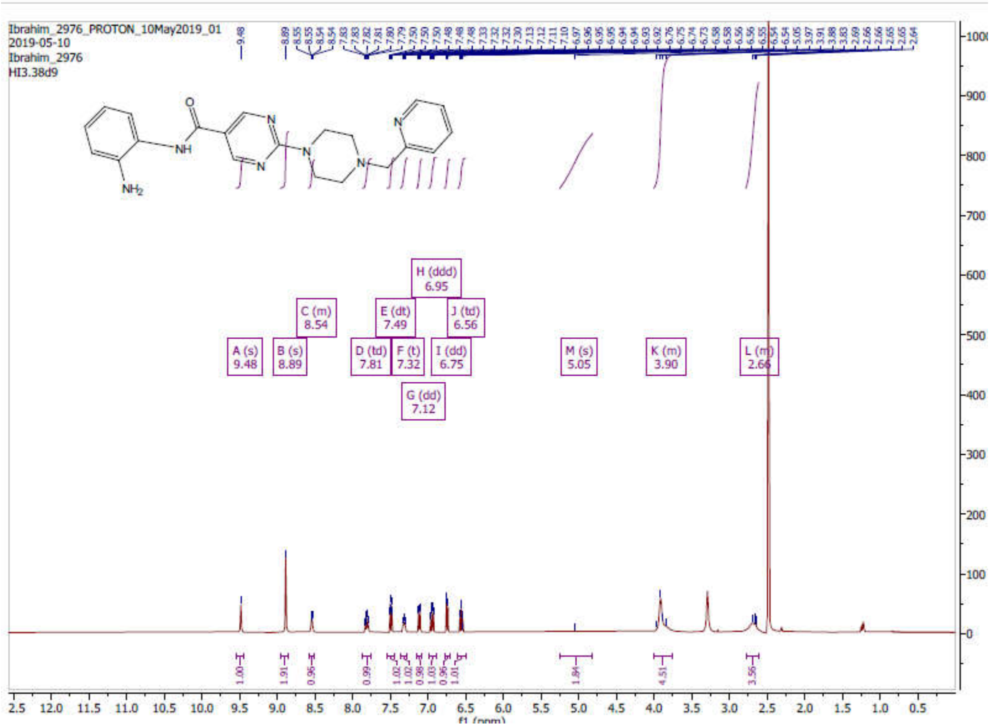

¹H NMR chart of compound **19d**

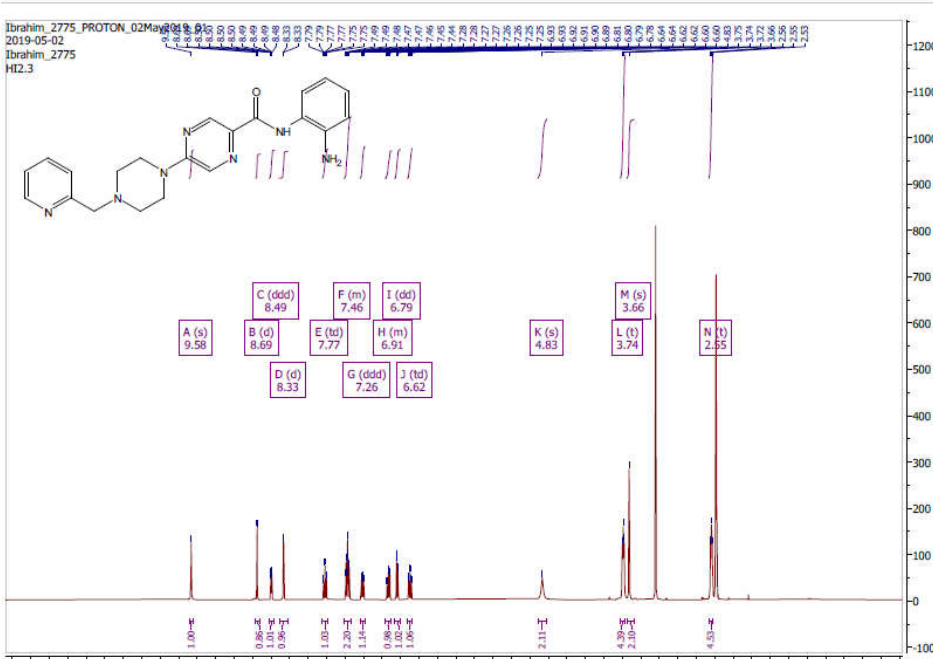

¹H NMR chart of compound **19e**

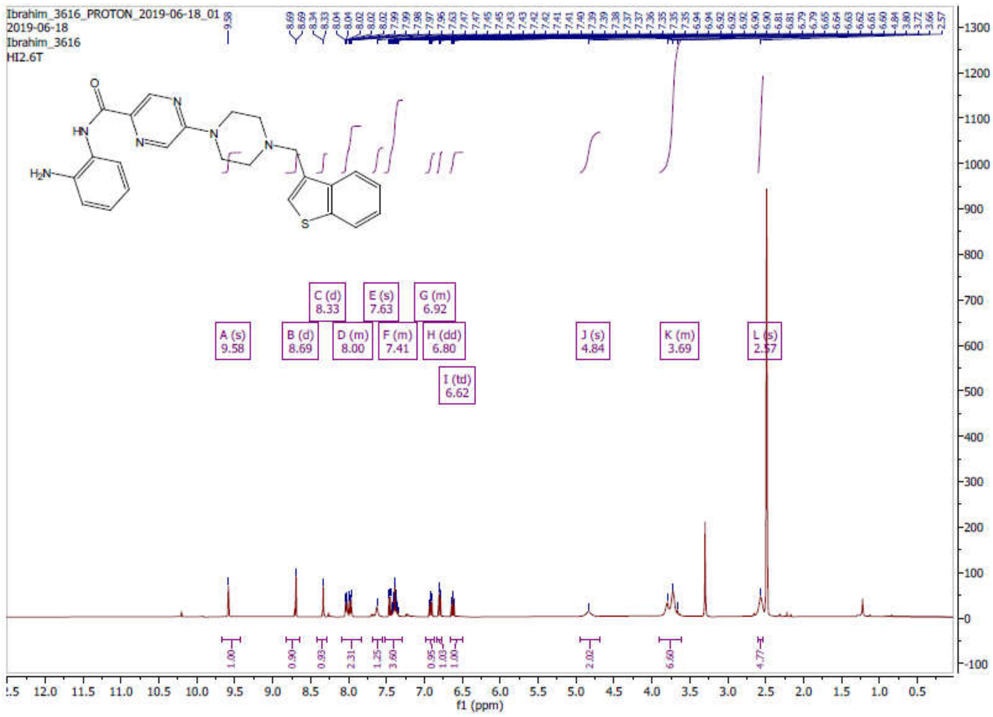

^{1}H NMR chart of compound **19f**

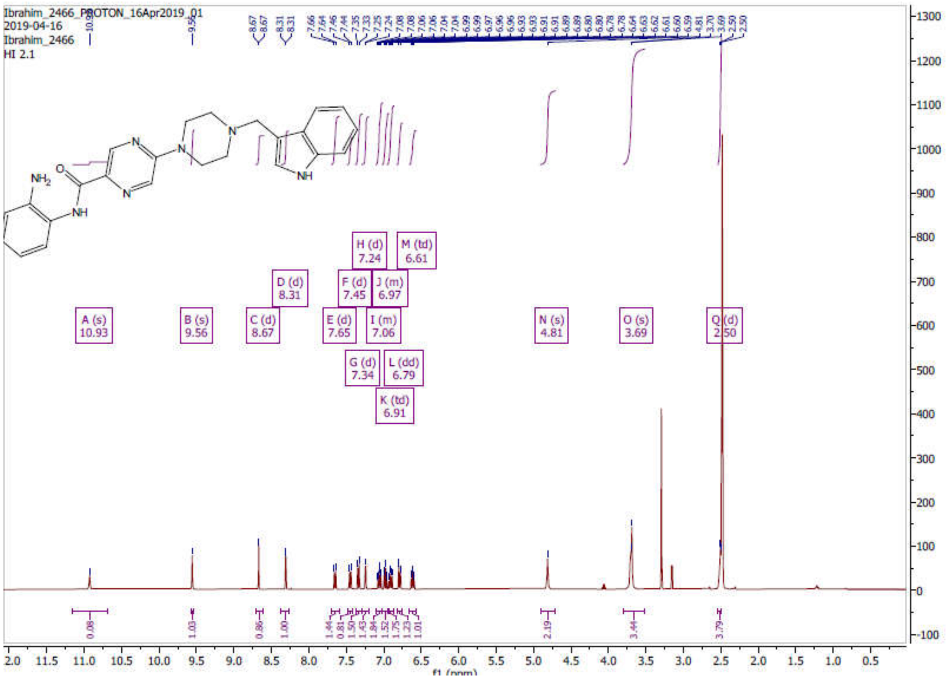

^{1}H NMR chart of compound **19g**

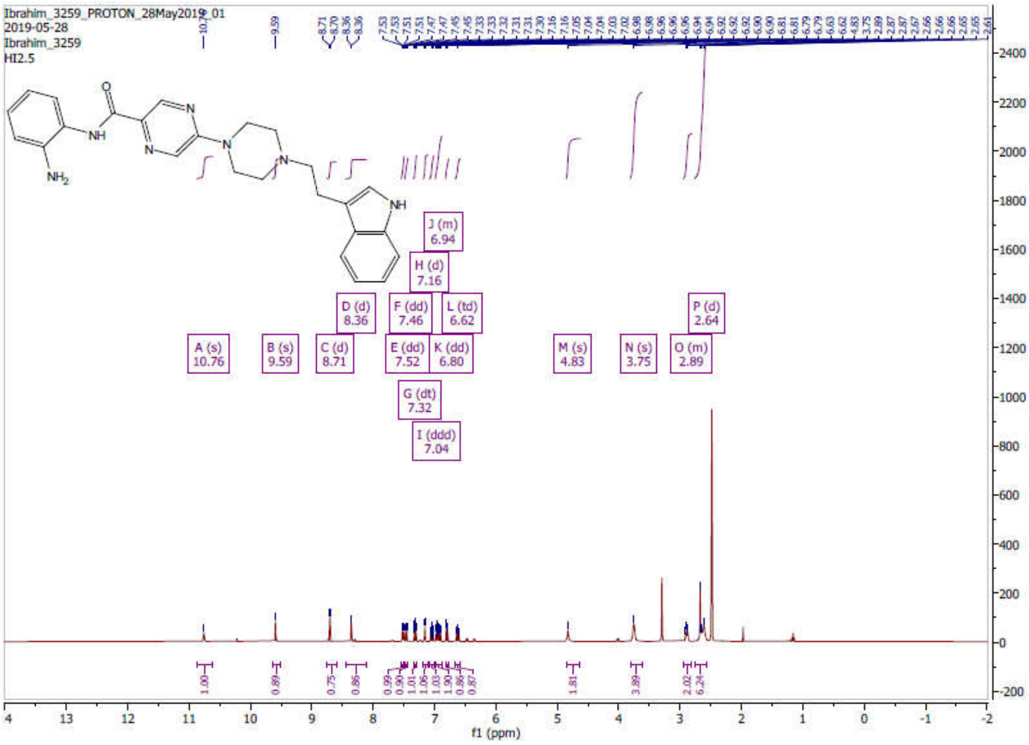

^{1}H NMR chart of compound **19h**

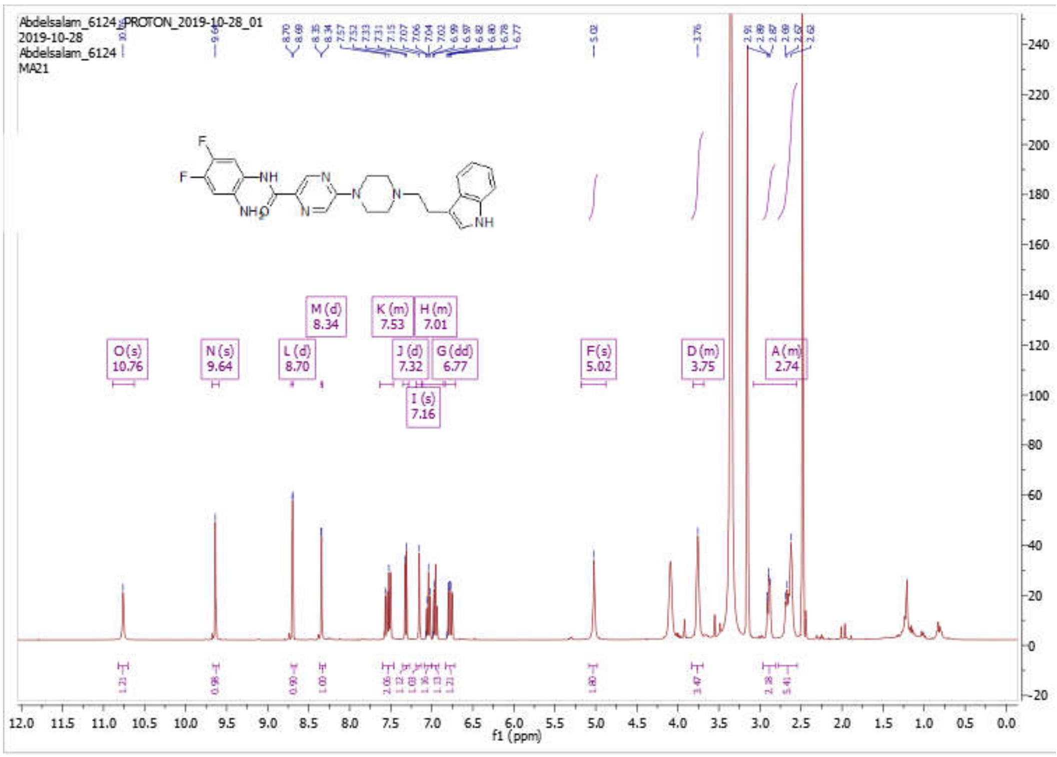

^{1}H NMR chart of compound **19i**

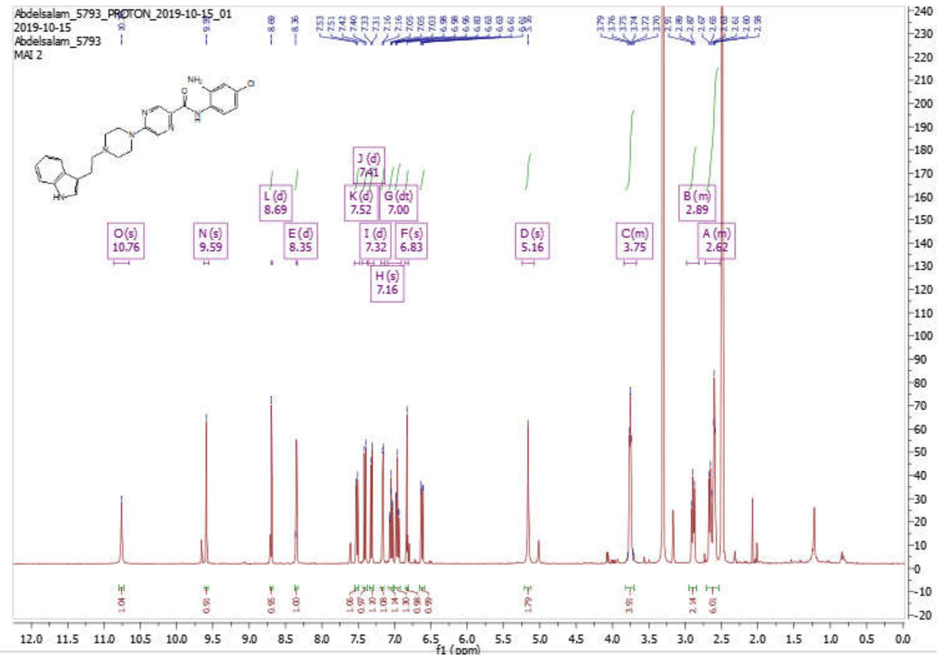

^{1}H NMR chart of compound **19j**

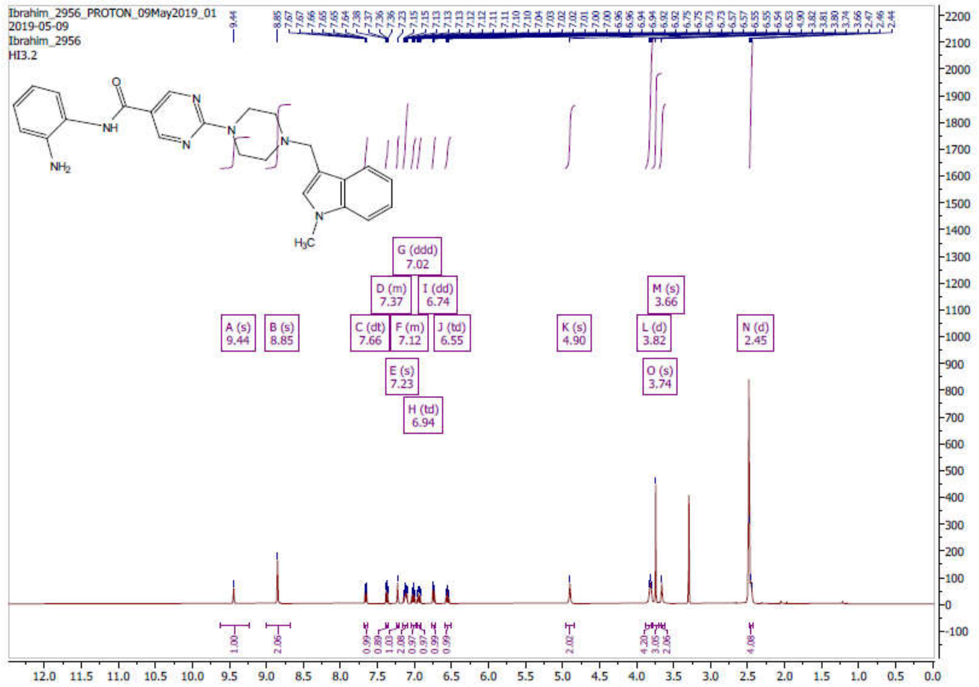

^{1}H NMR chart of compound **19k**

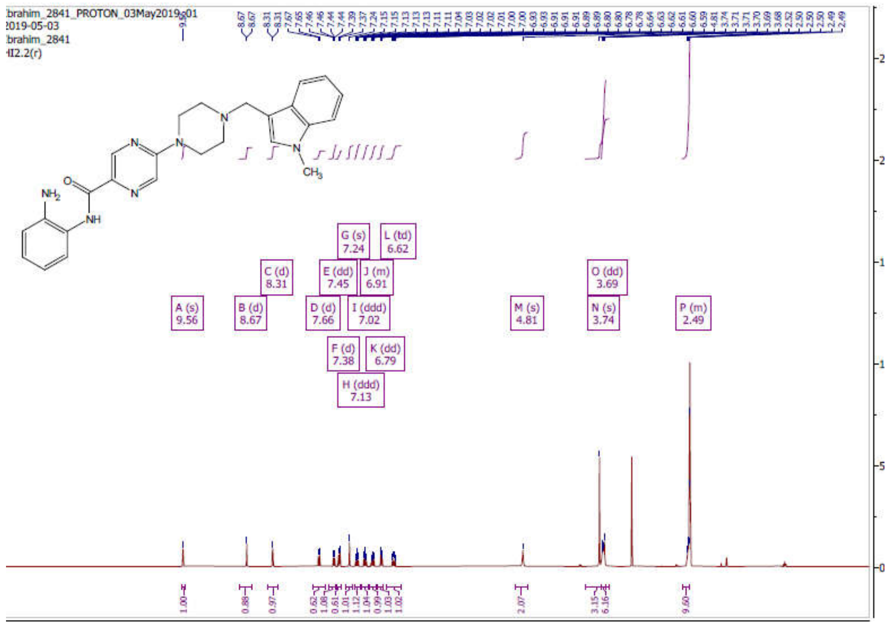

^{1}H NMR chart of compound **19l**

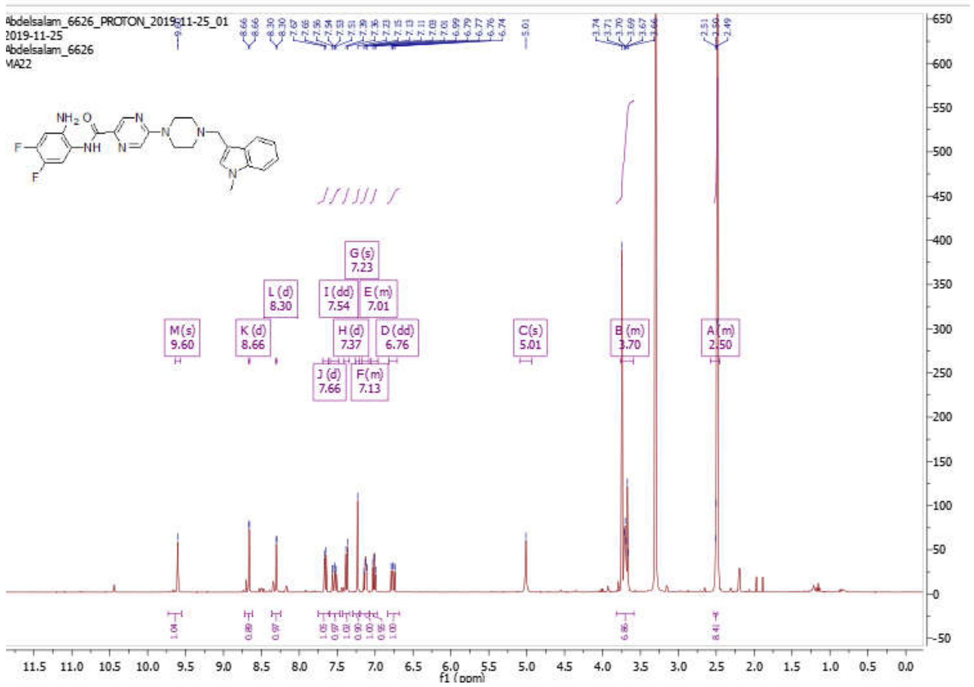

^{1}H NMR chart of compound **19m**

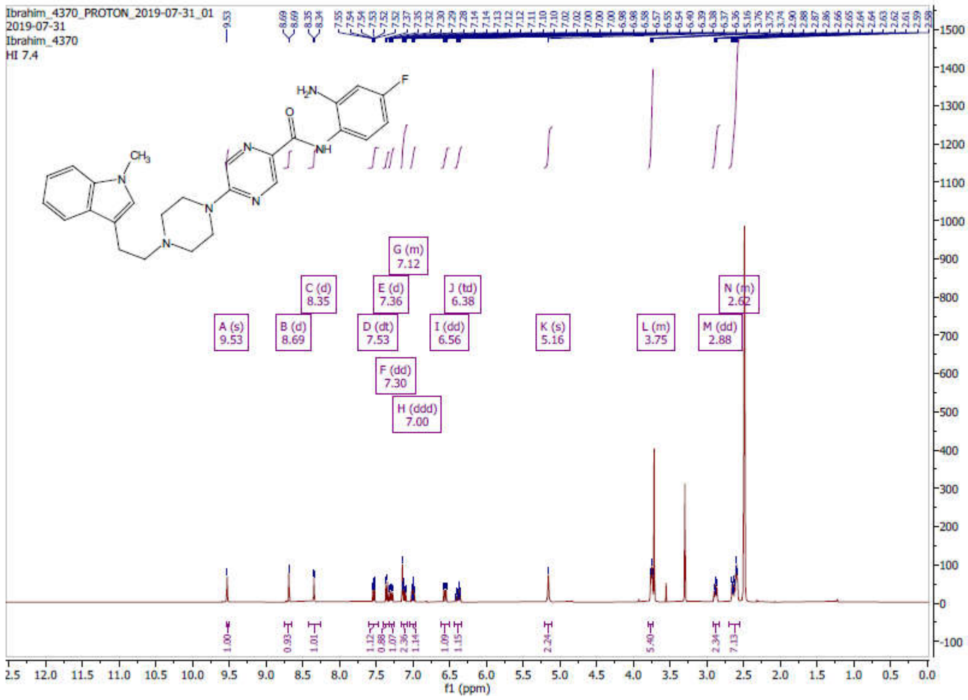

¹H NMR chart of compound **19n**

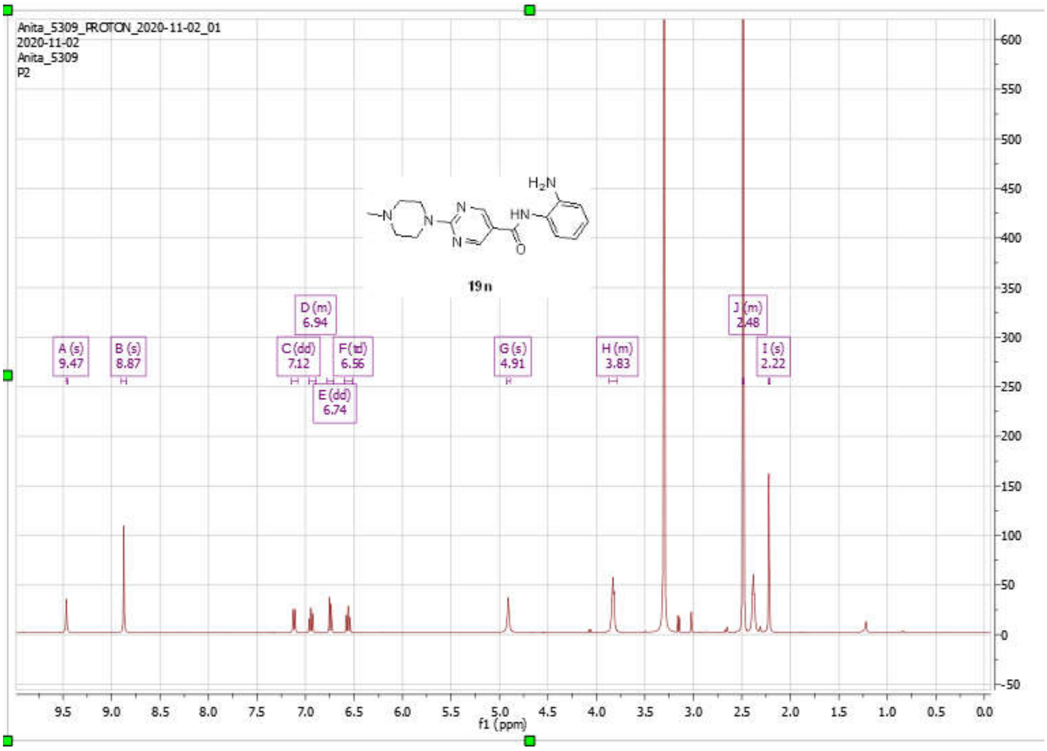

¹H NMR chart of compound **19o**

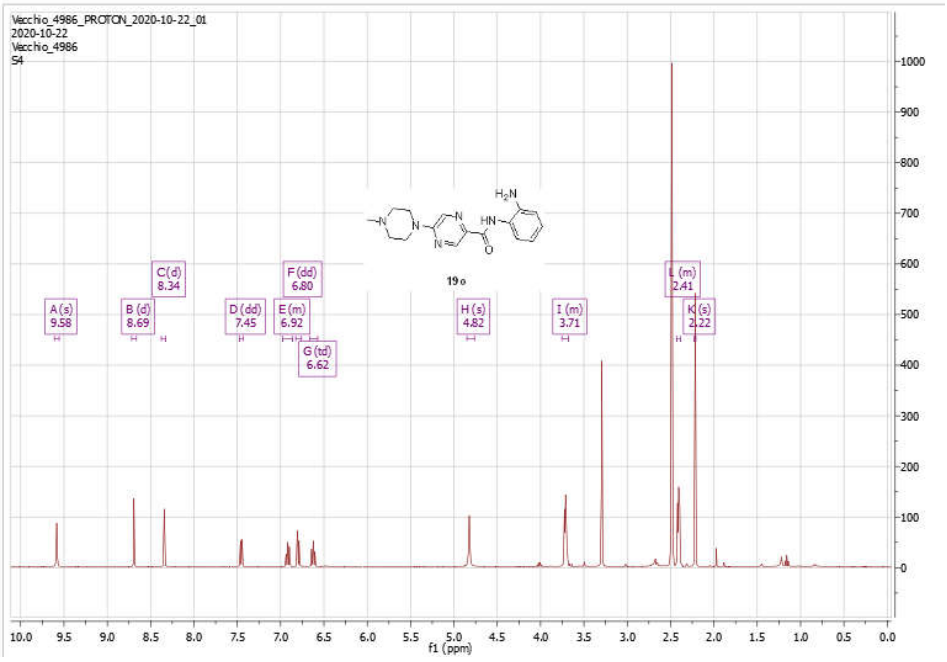

^{1}H NMR chart of compound **21a**

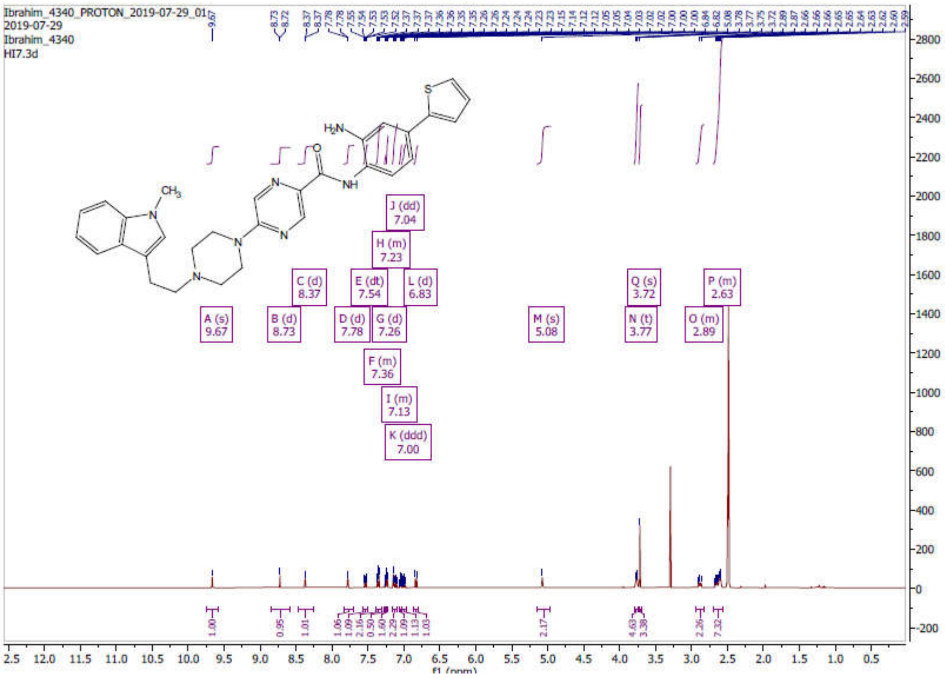

^{1}H NMR chart of compound **21b**

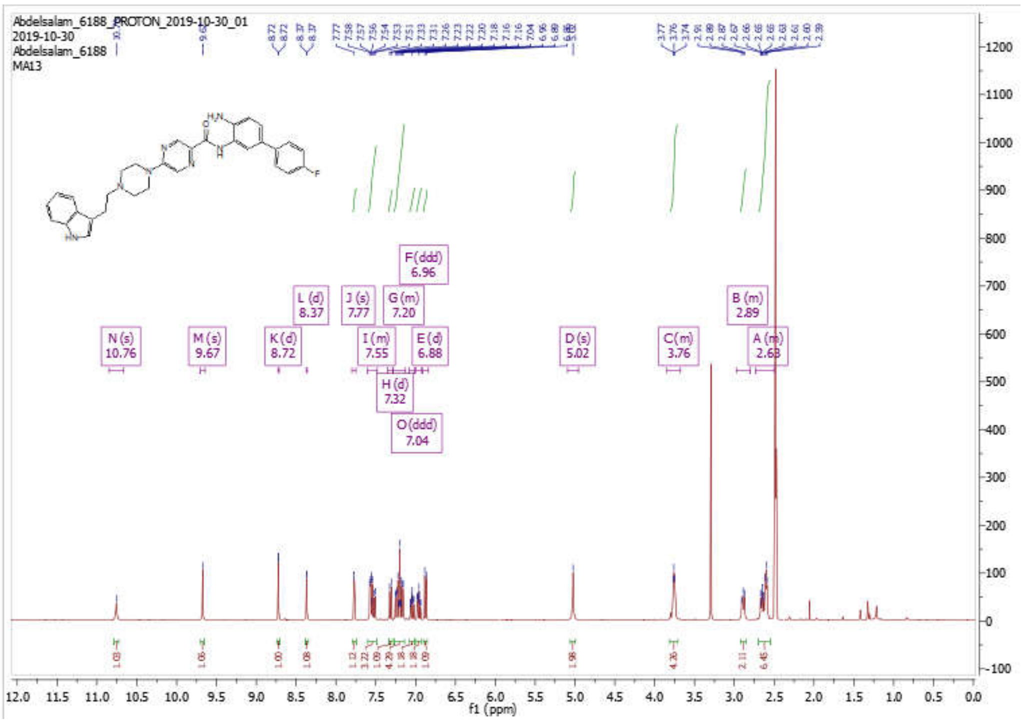

^{1}H NMR chart of compound **21c**

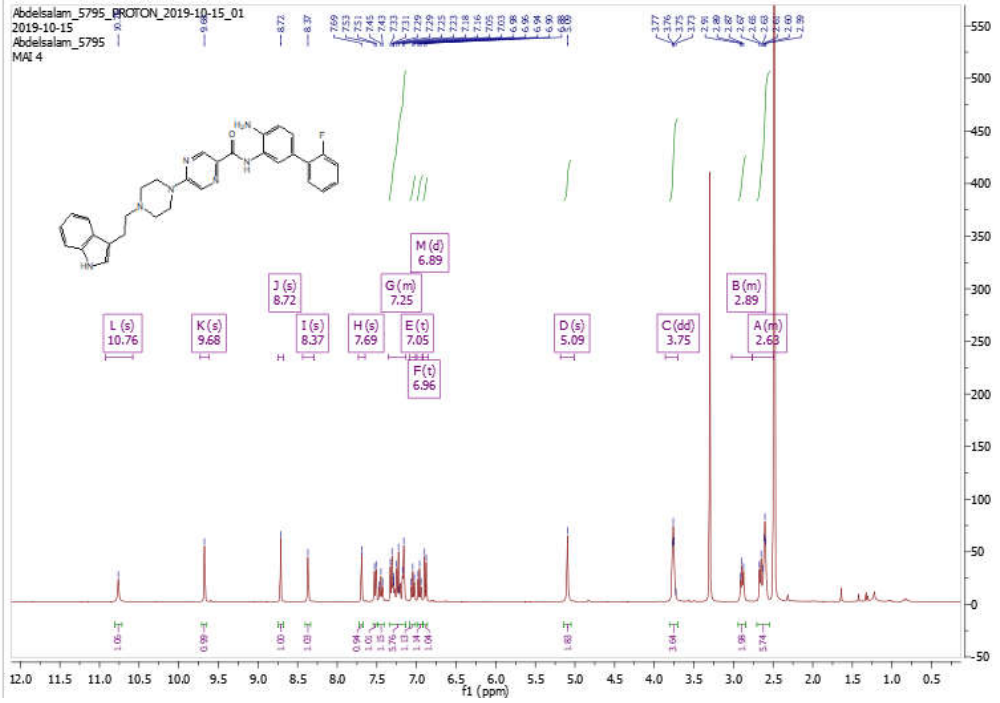

^{1}H NMR chart of compound **23a**

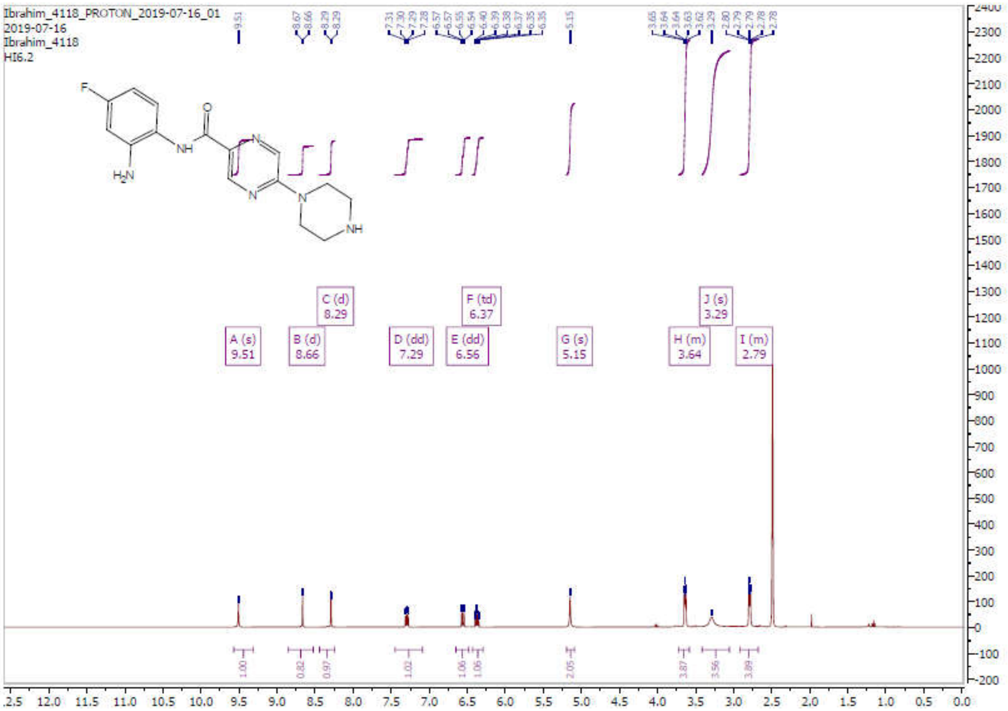

^{1}H NMR chart of compound **23b**

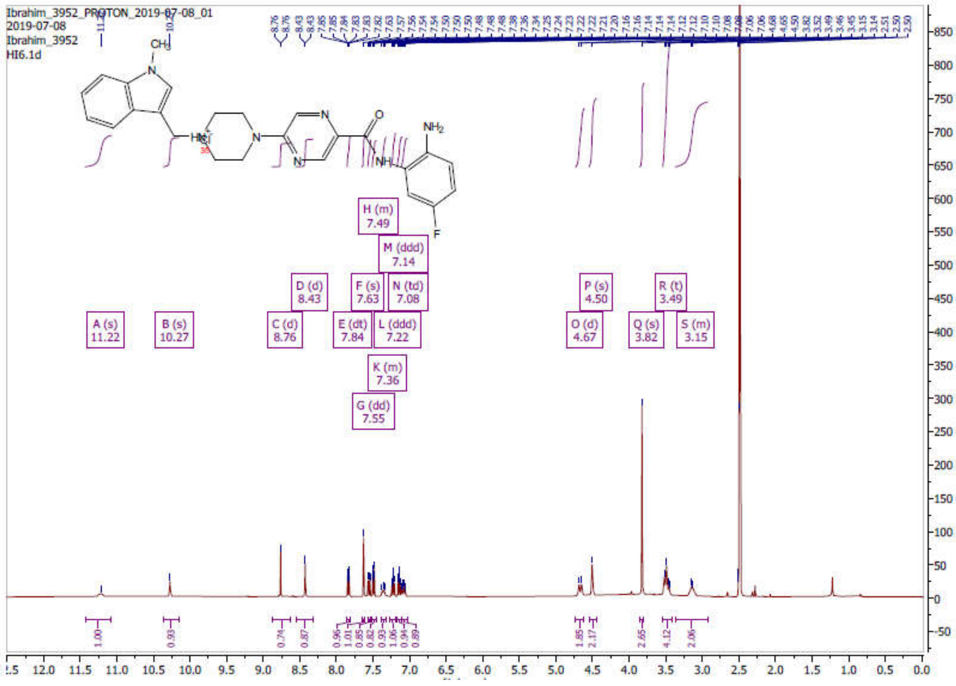

^{1}H NMR chart of compound **23c**

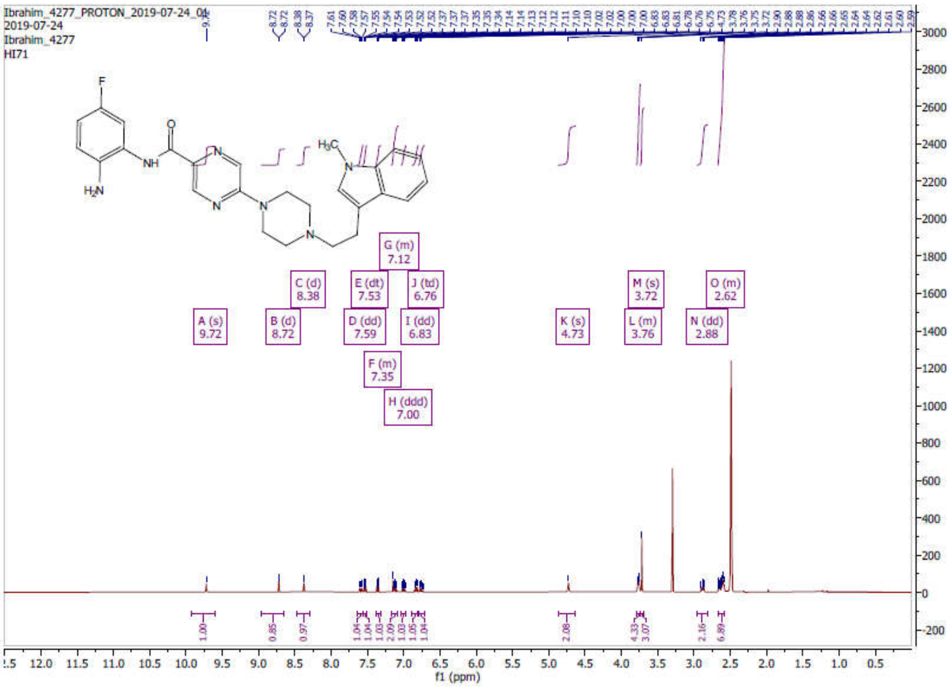

^{1}H NMR chart of compound **25a**

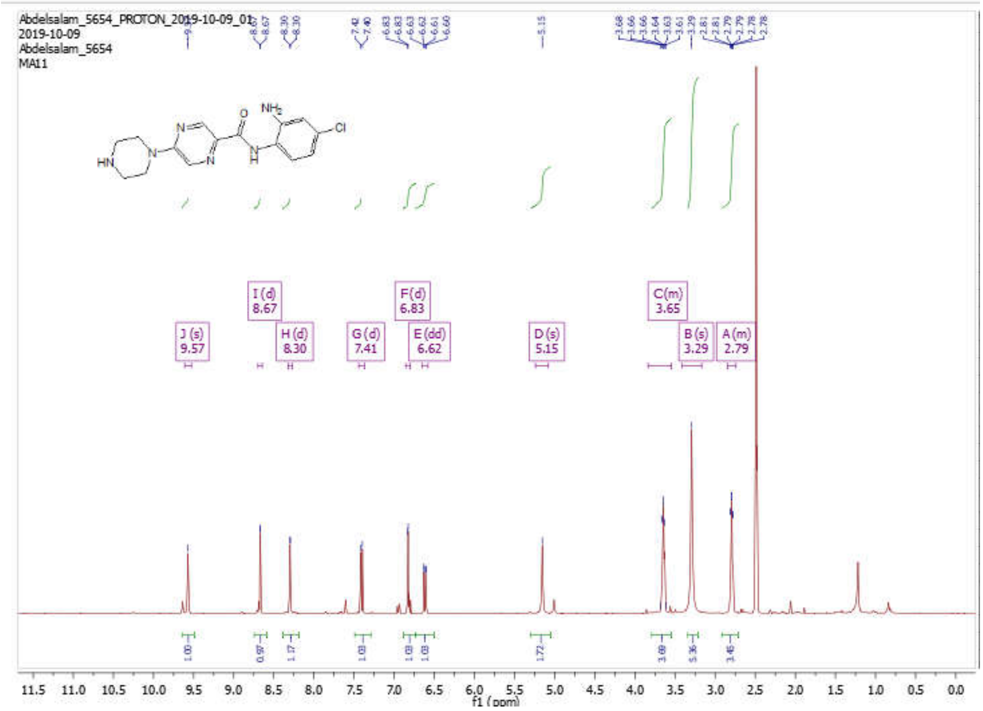

^{1}H NMR chart of compound **25b**

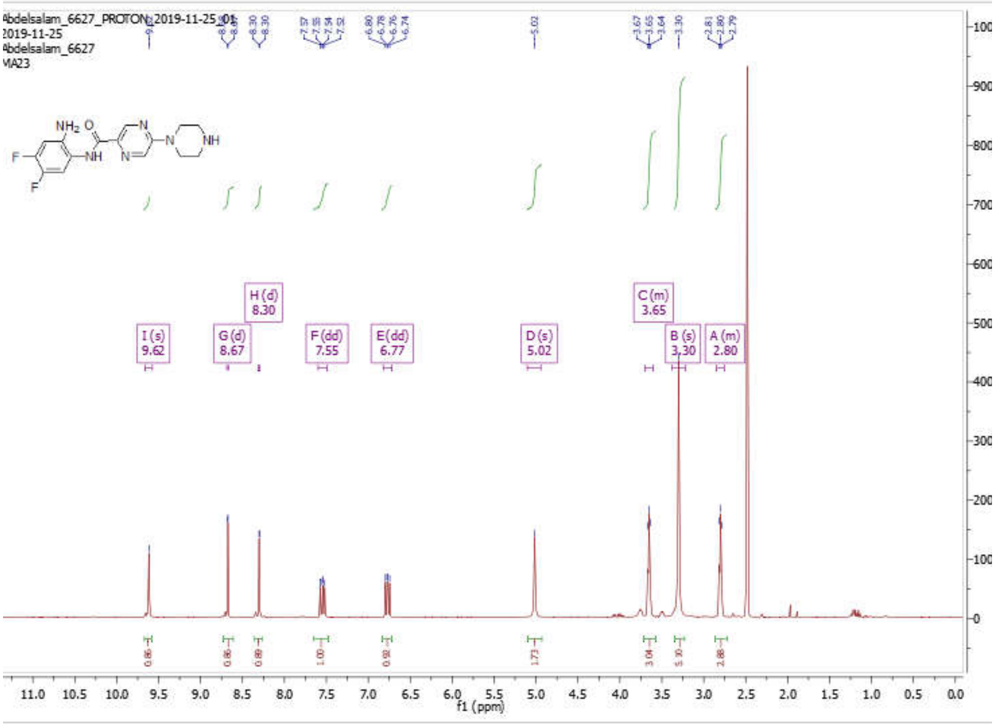

^{1}H NMR chart of compound **27a**

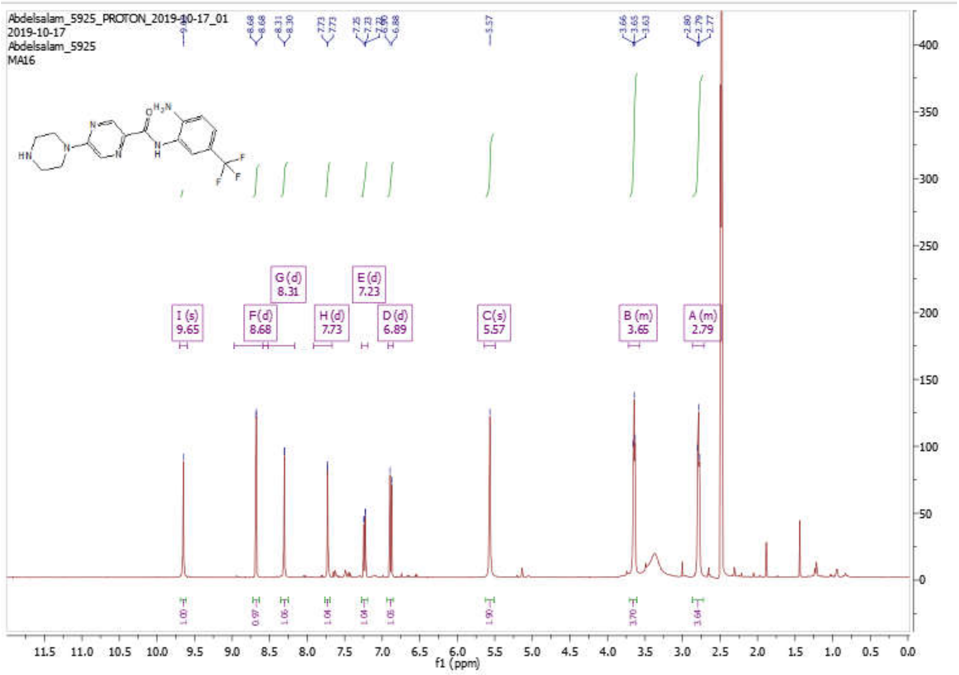

^{1}H NMR chart of compound **27b**

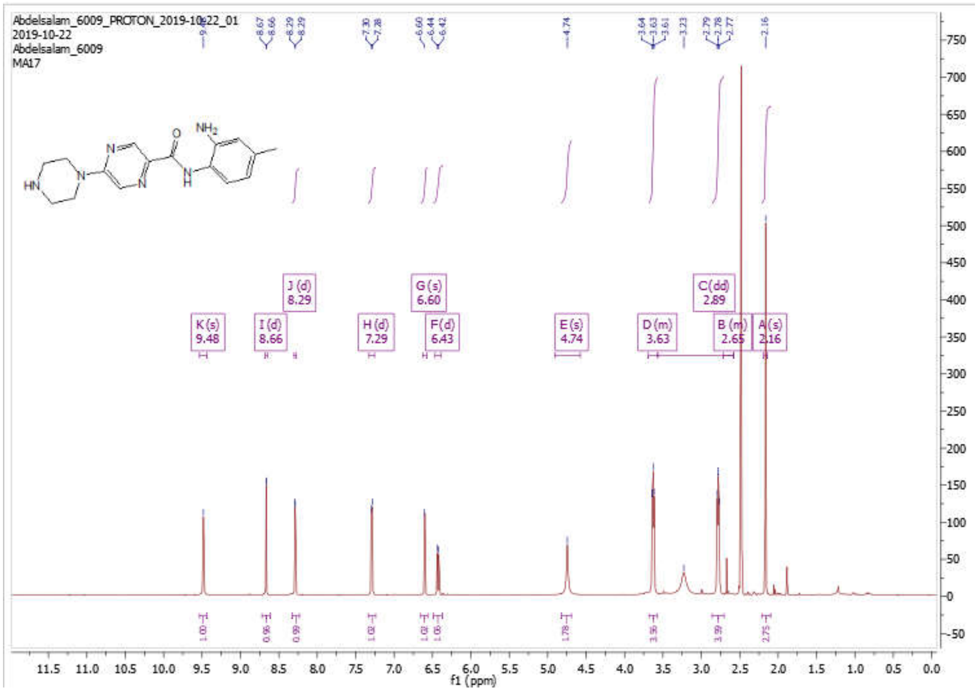

^{1}H NMR chart of compound **27c**

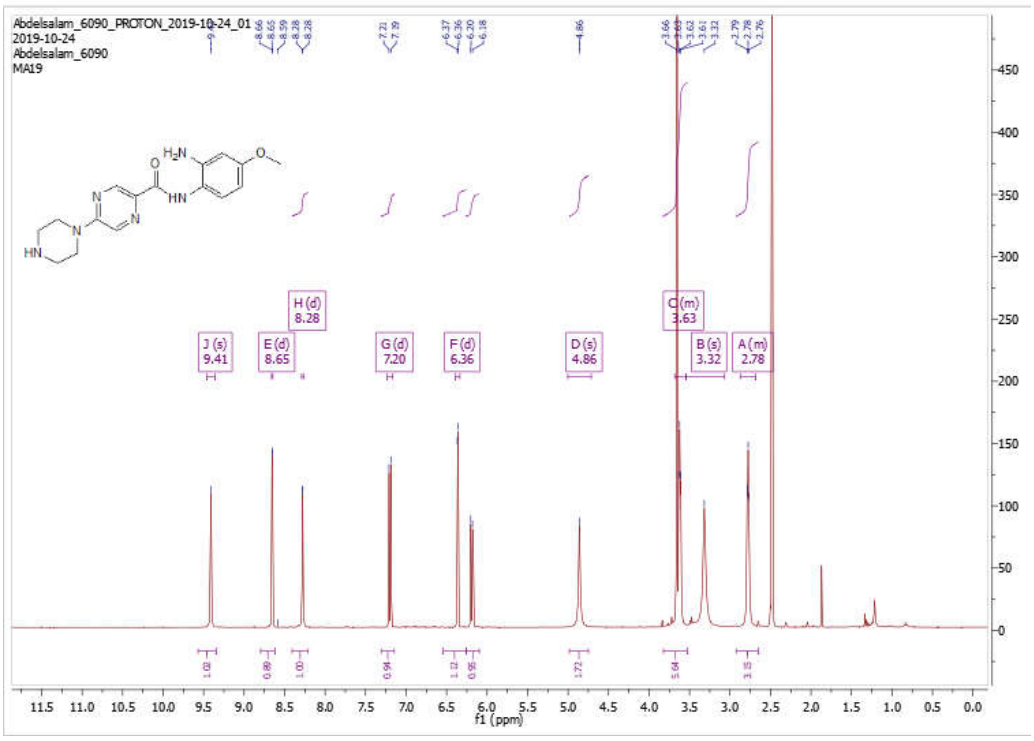

^{1}H NMR chart of compound **29a**

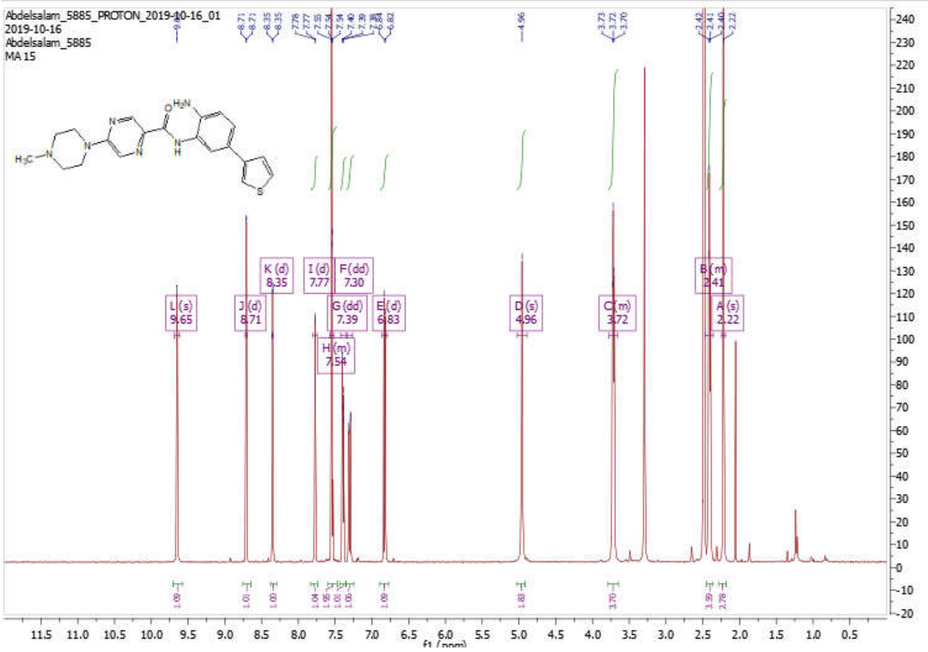

^{1}H NMR chart of compound **29b**

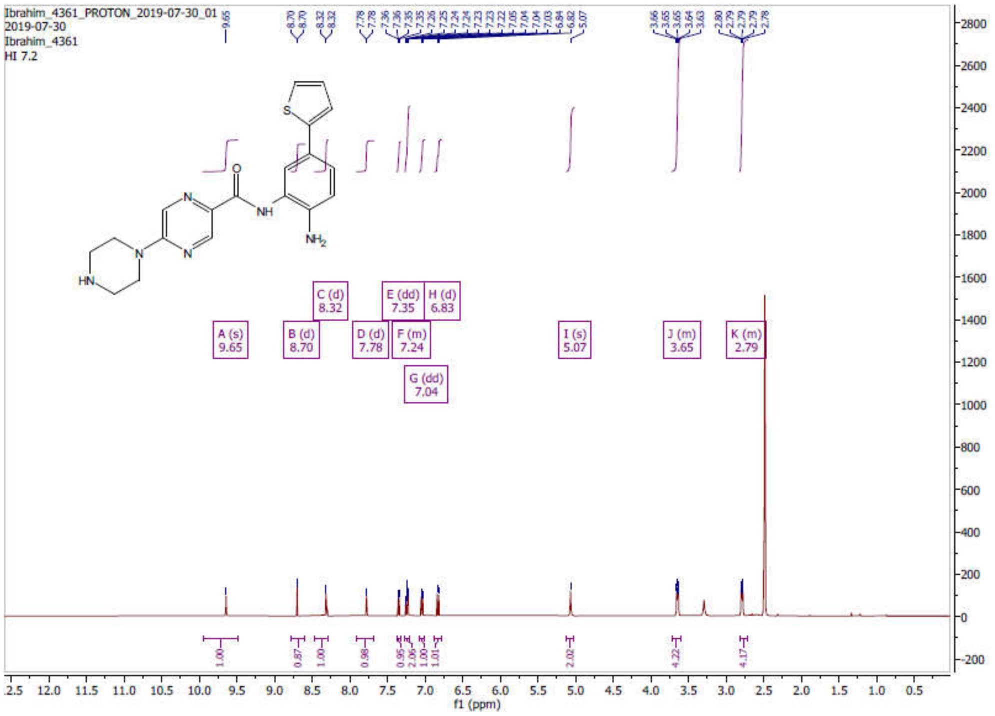

^{1}H NMR chart of compound **29c**

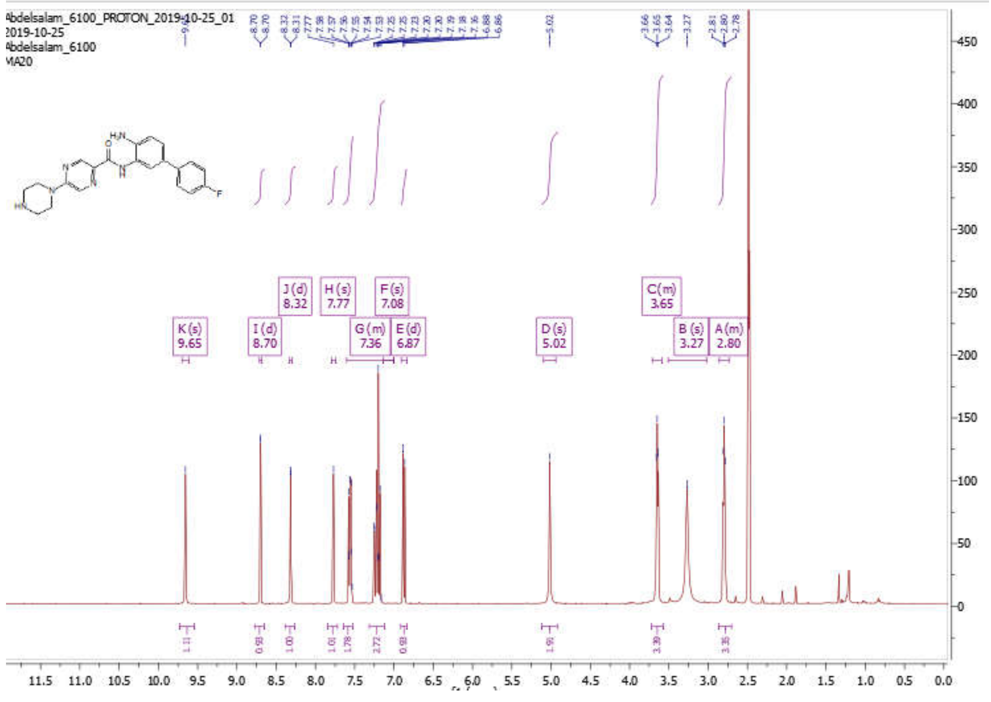

4. ^{13}C NMR charts of final compounds

^{13}C NMR chart of compound **19a**

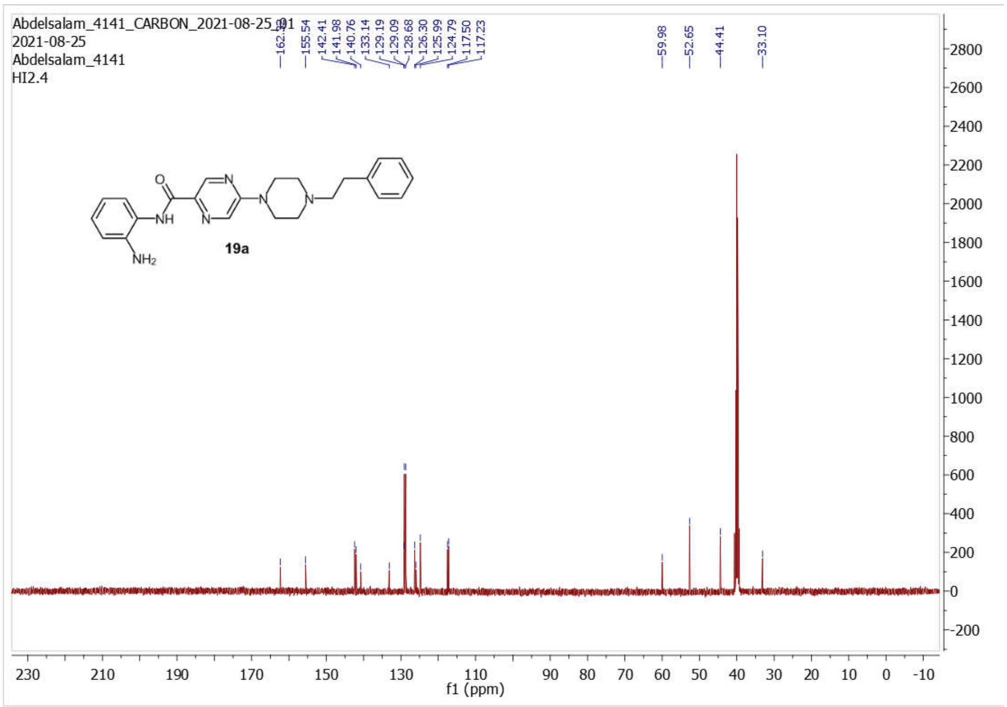

^{13}C NMR chart of compound **19b**

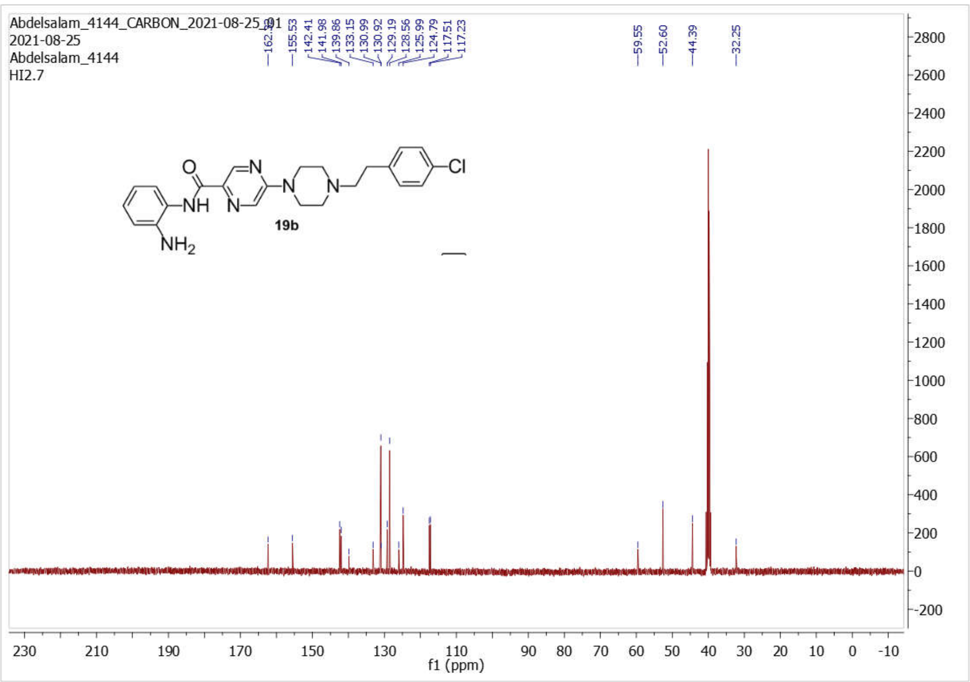

^{13}C *NMR chart of compound* **19c**

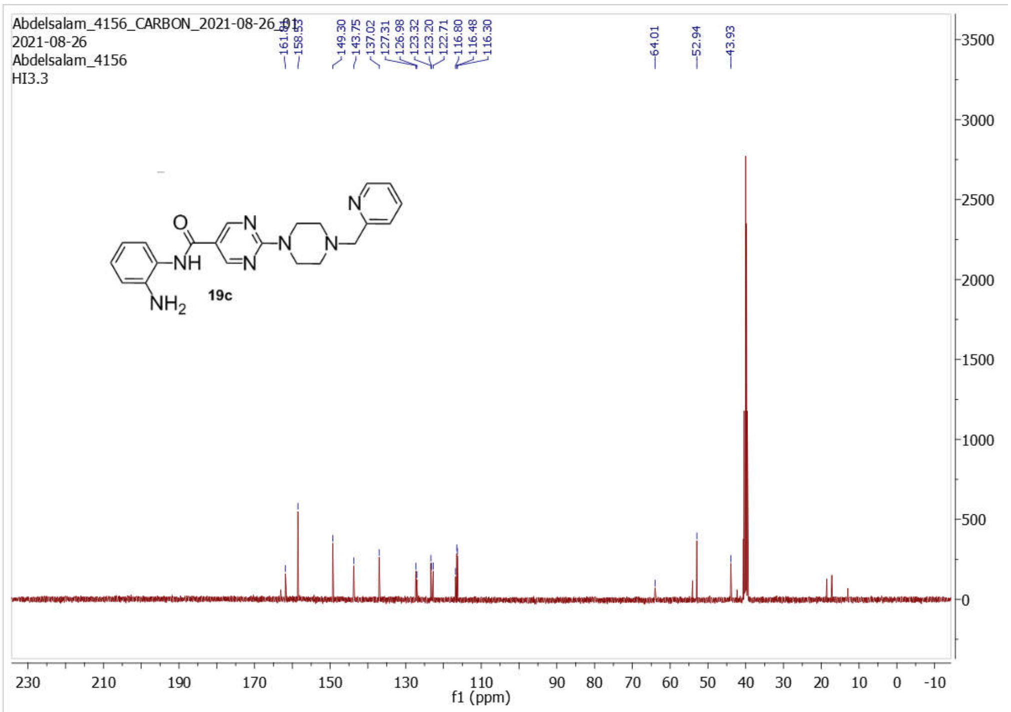

^{13}C *NMR chart of compound* **19d**

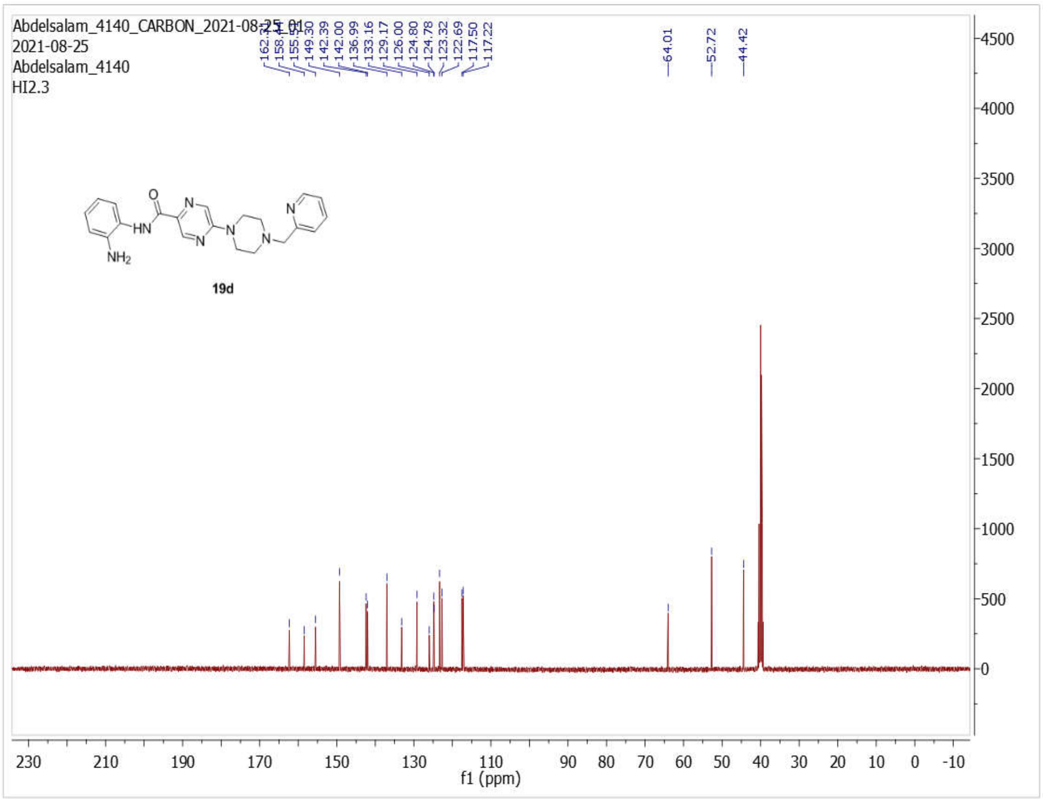

^{13}C *NMR chart of compound* **19f**

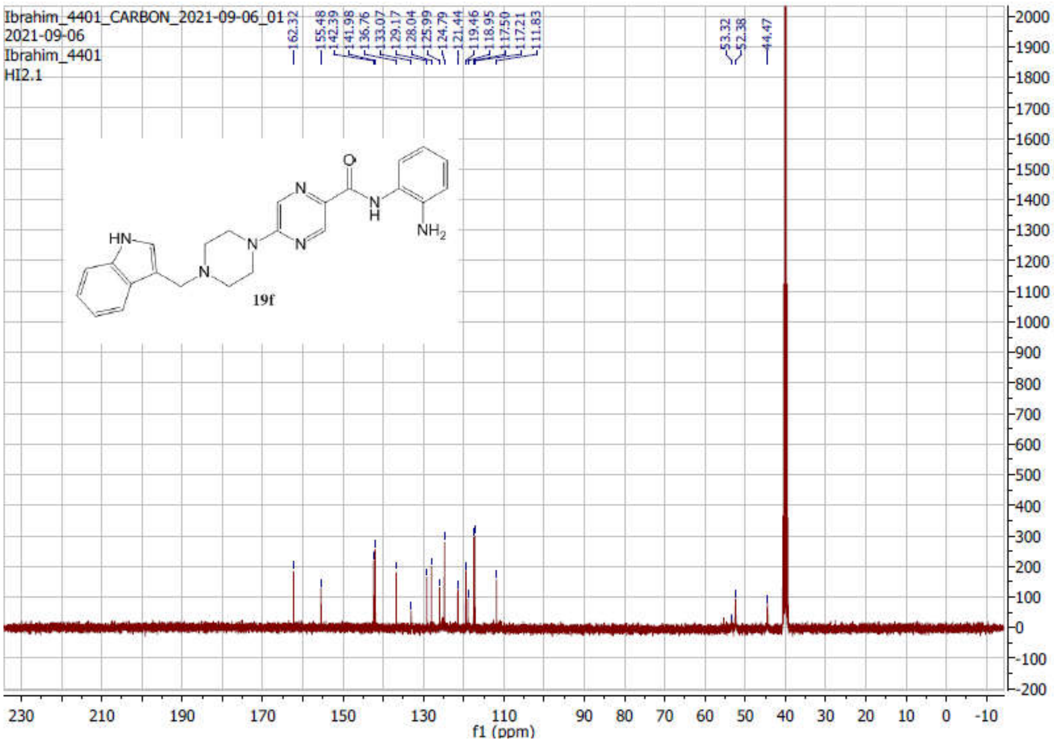

^{13}C *NMR chart of compound* **19g**

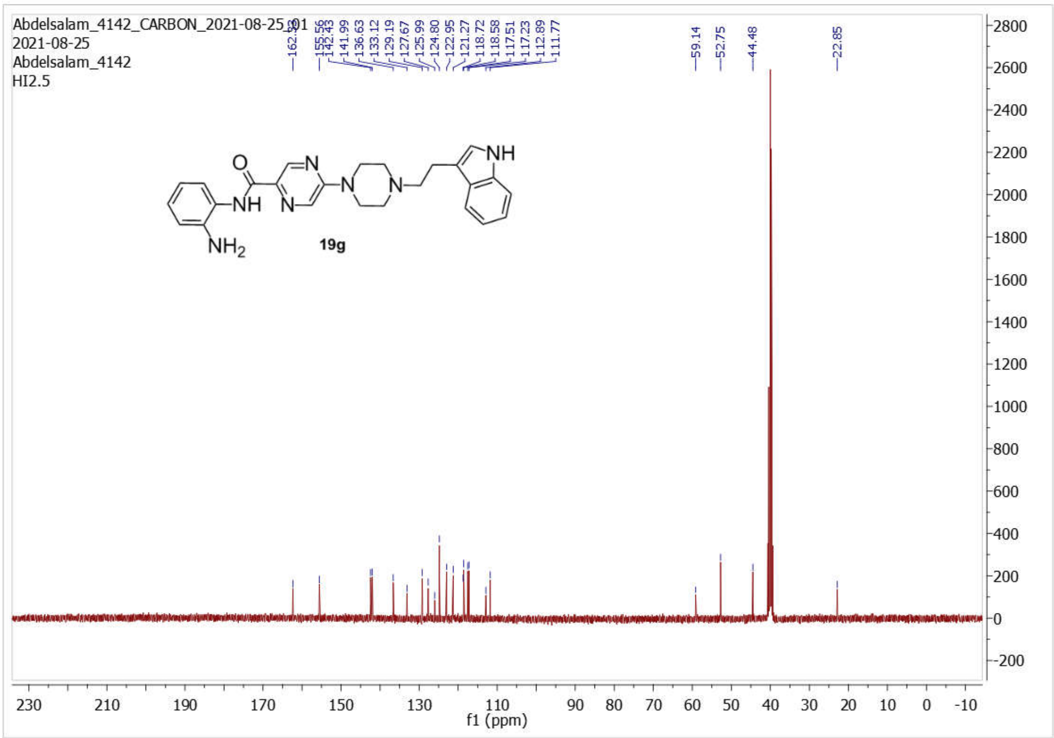

^{13}C *NMR chart of compound* **19h**

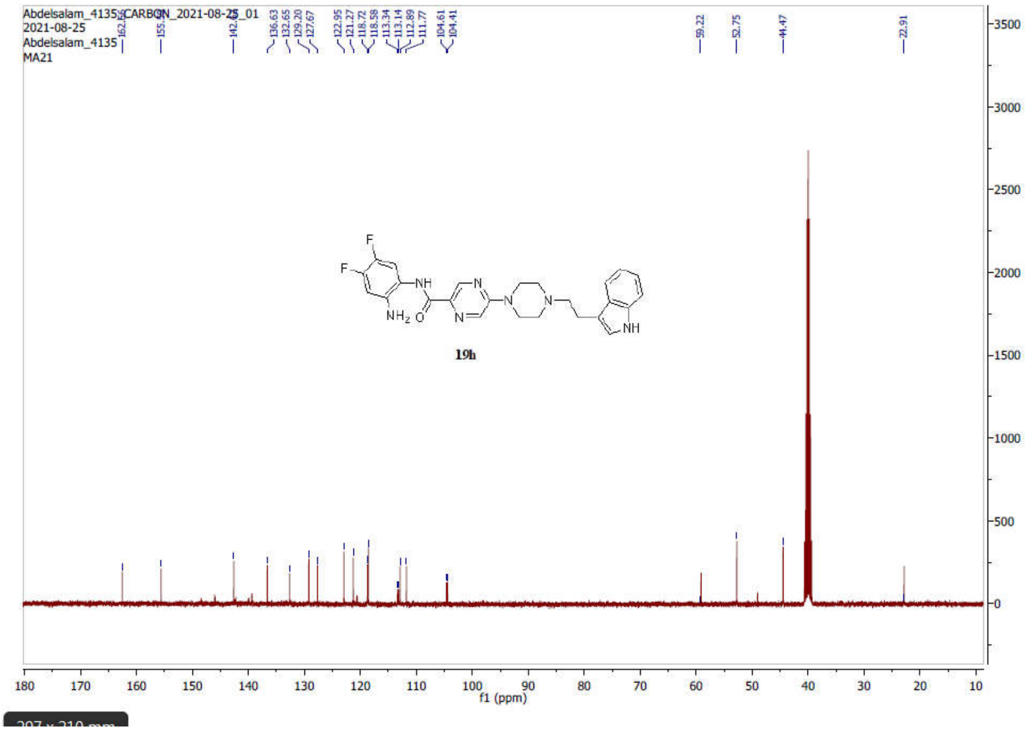

^{13}C *NMR chart of compound* **19i**

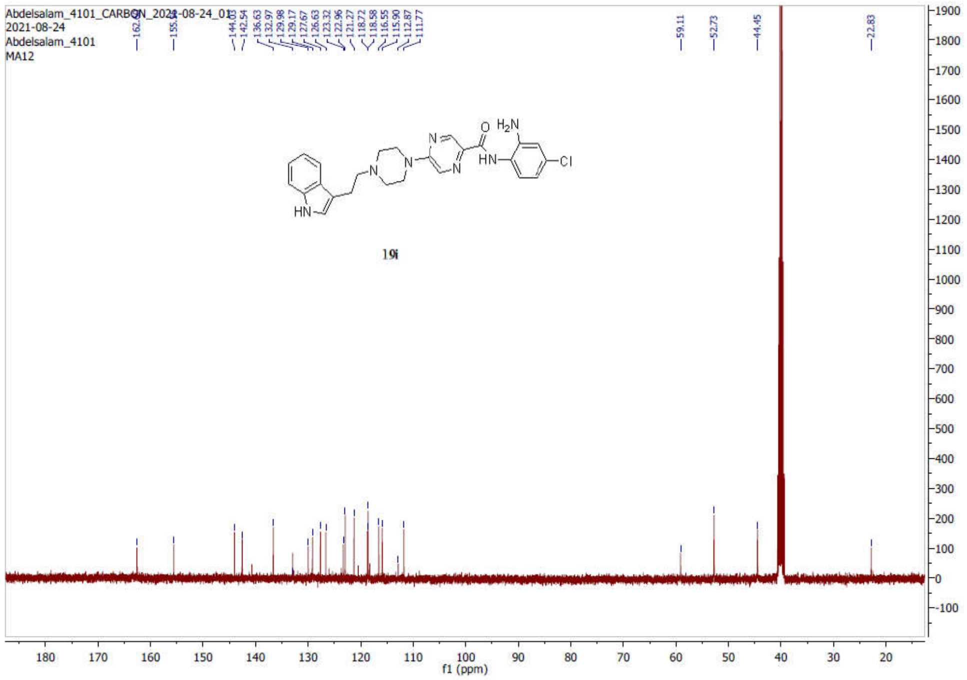

^{13}C NMR chart of compound **19k**

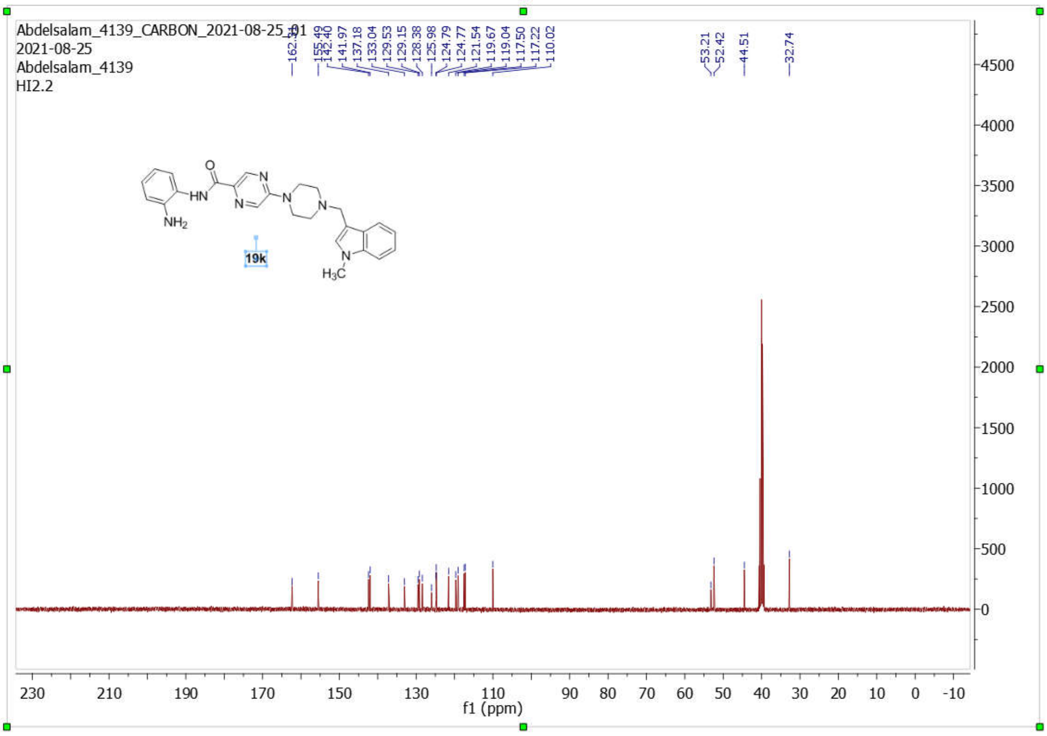

^{13}C NMR chart of compound **19l**

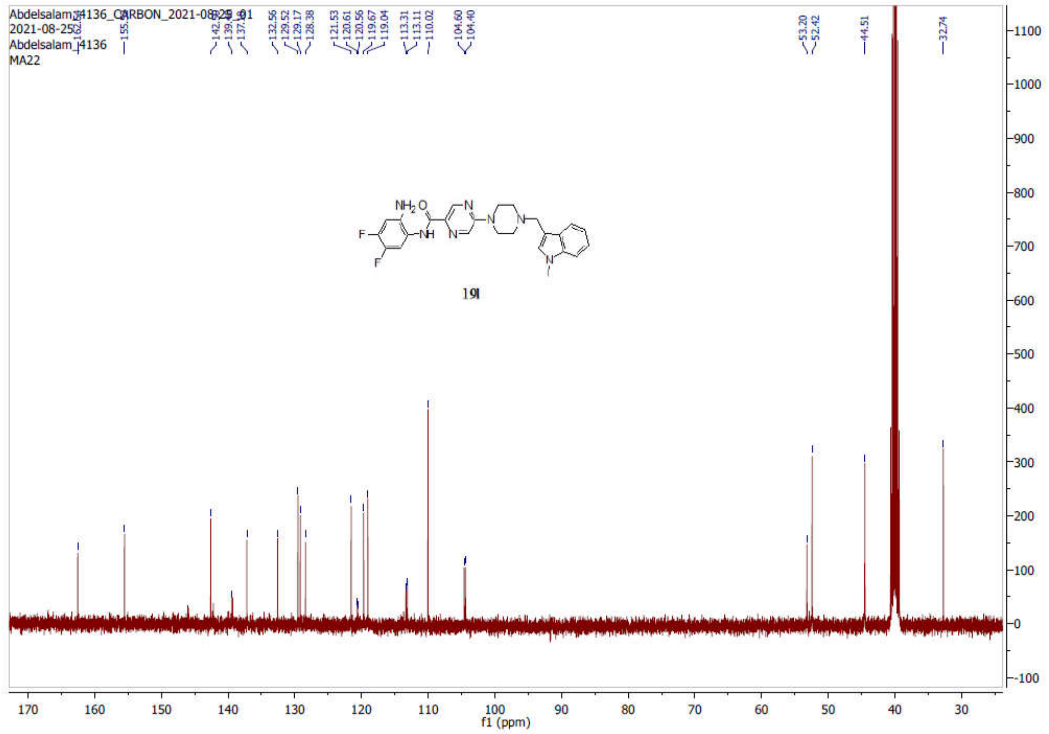

^{13}C NMR chart of compound **19m**

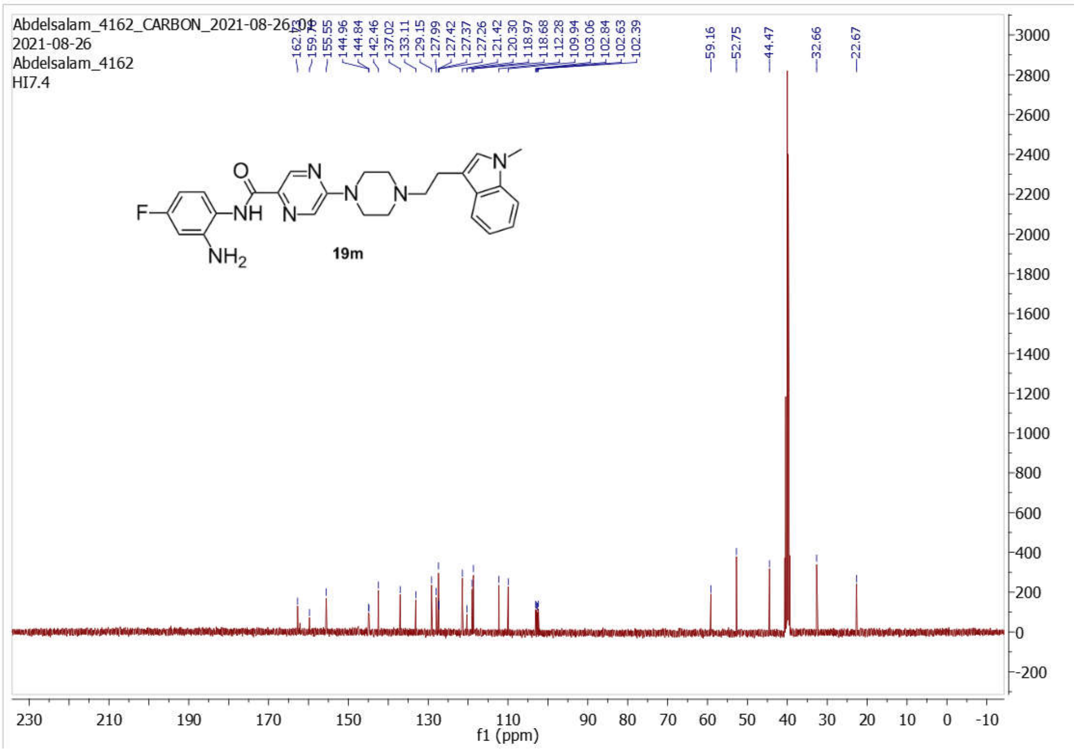

^{13}C NMR chart of compound **19n**

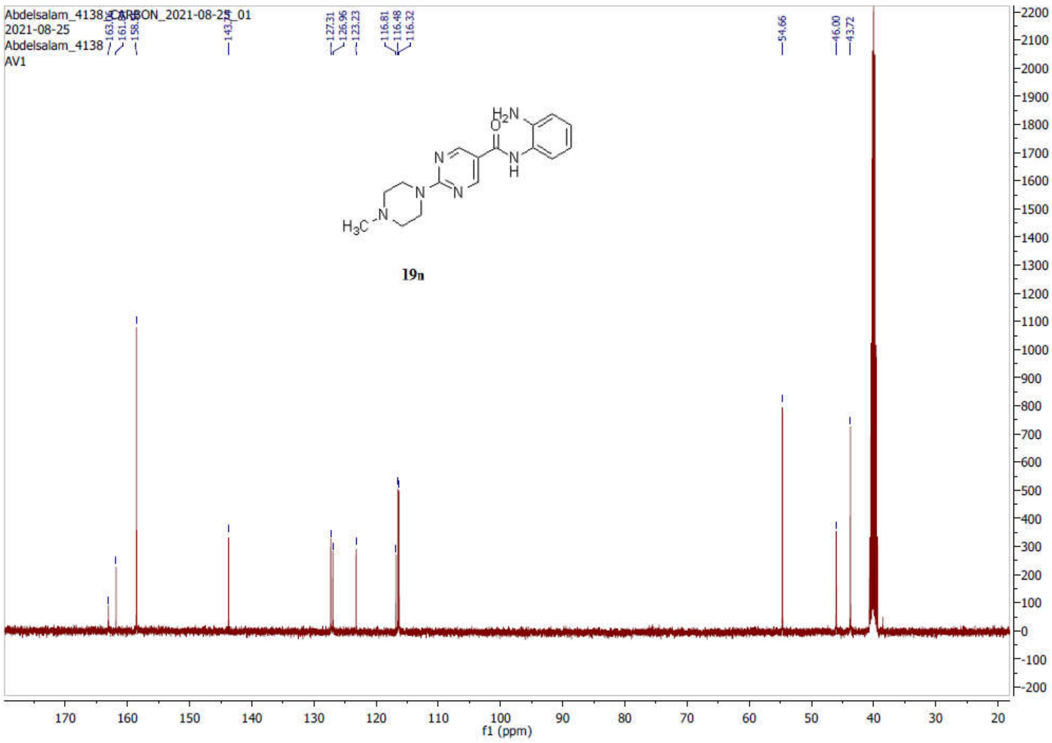

^{13}C NMR chart of compound **19o**

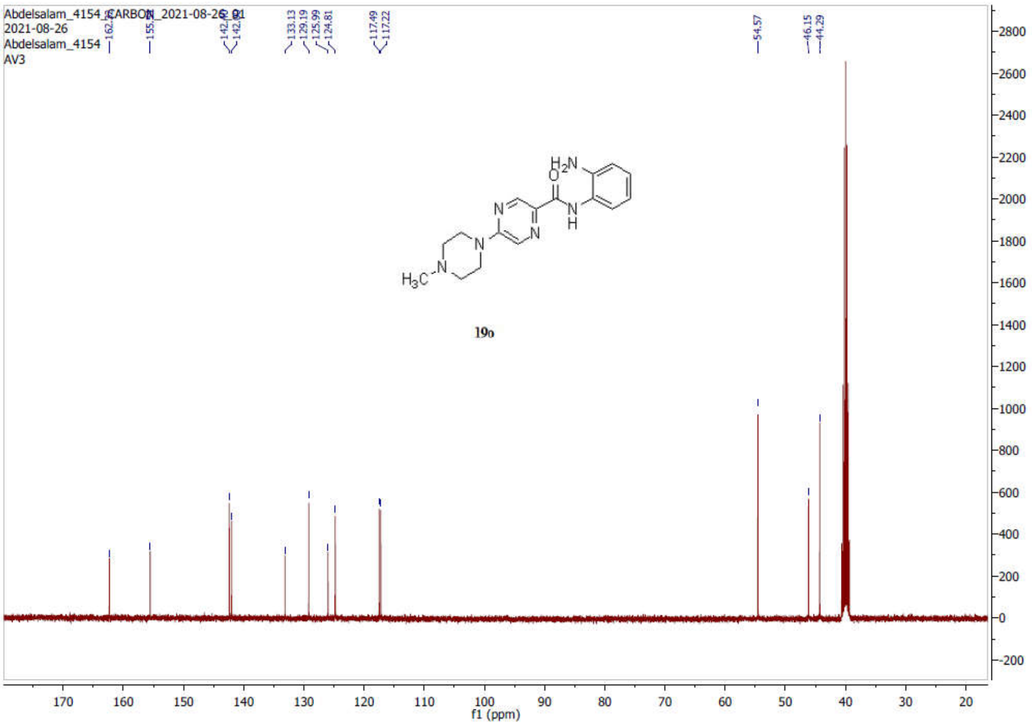

^{13}C NMR chart of compound **21a**

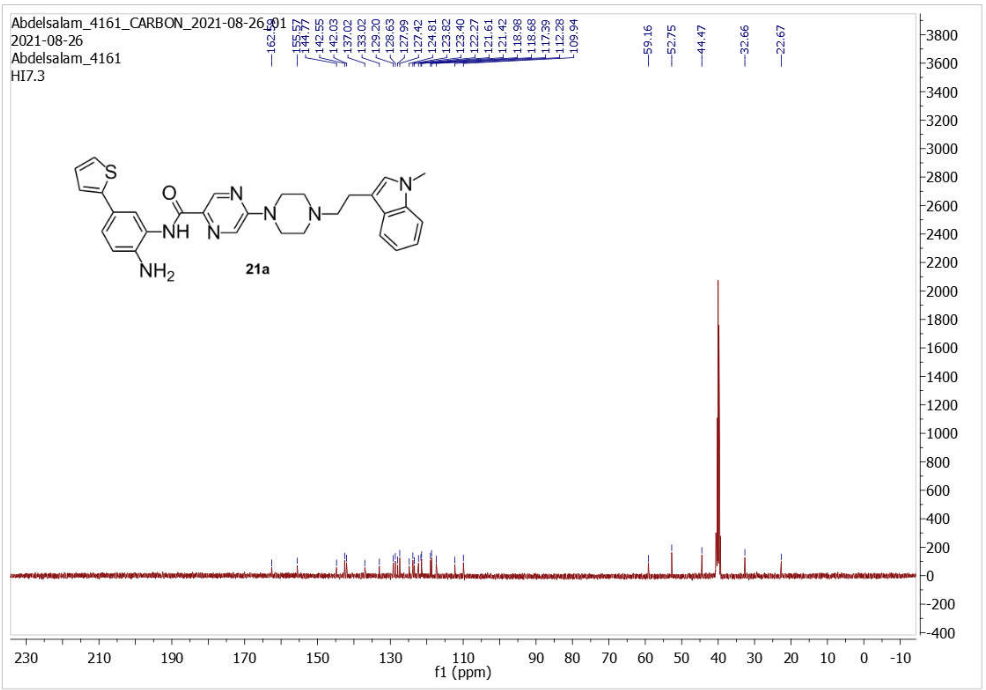

^{13}C *NMR chart of compound* **21b**

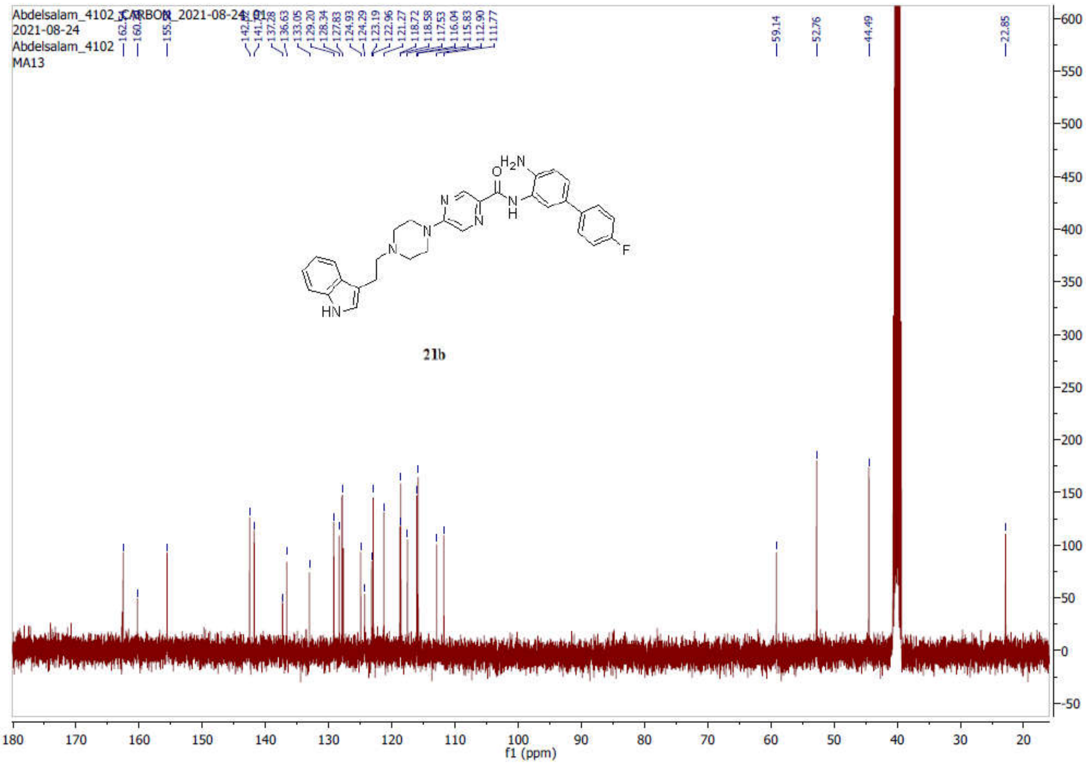

6.1. ^{13}C *NMR chart of compound* **21c**

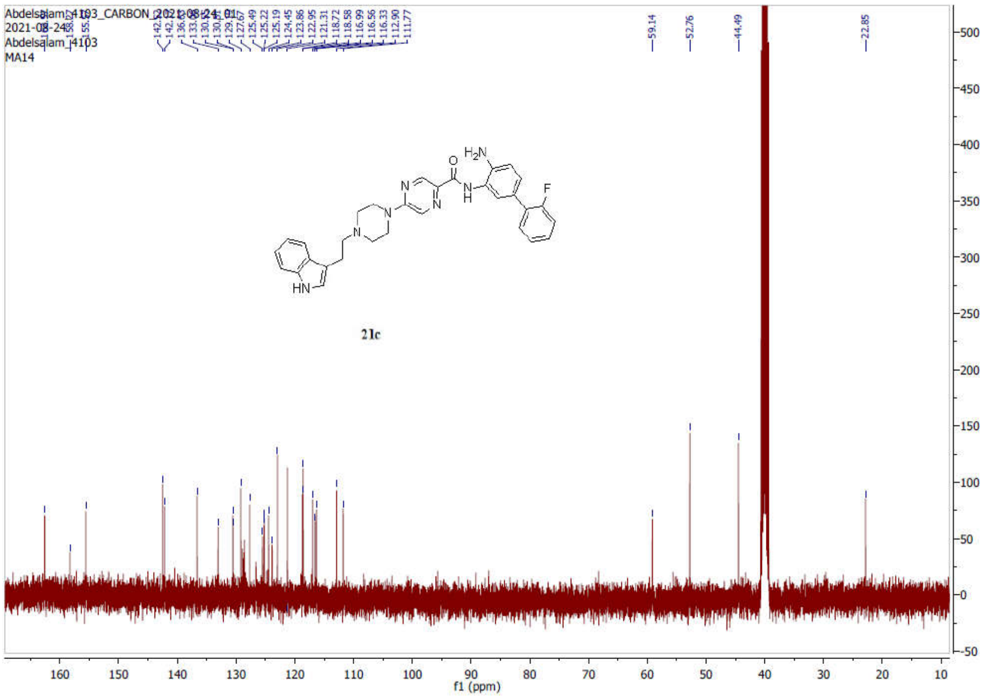

^{13}C *NMR chart of compound* **23a**

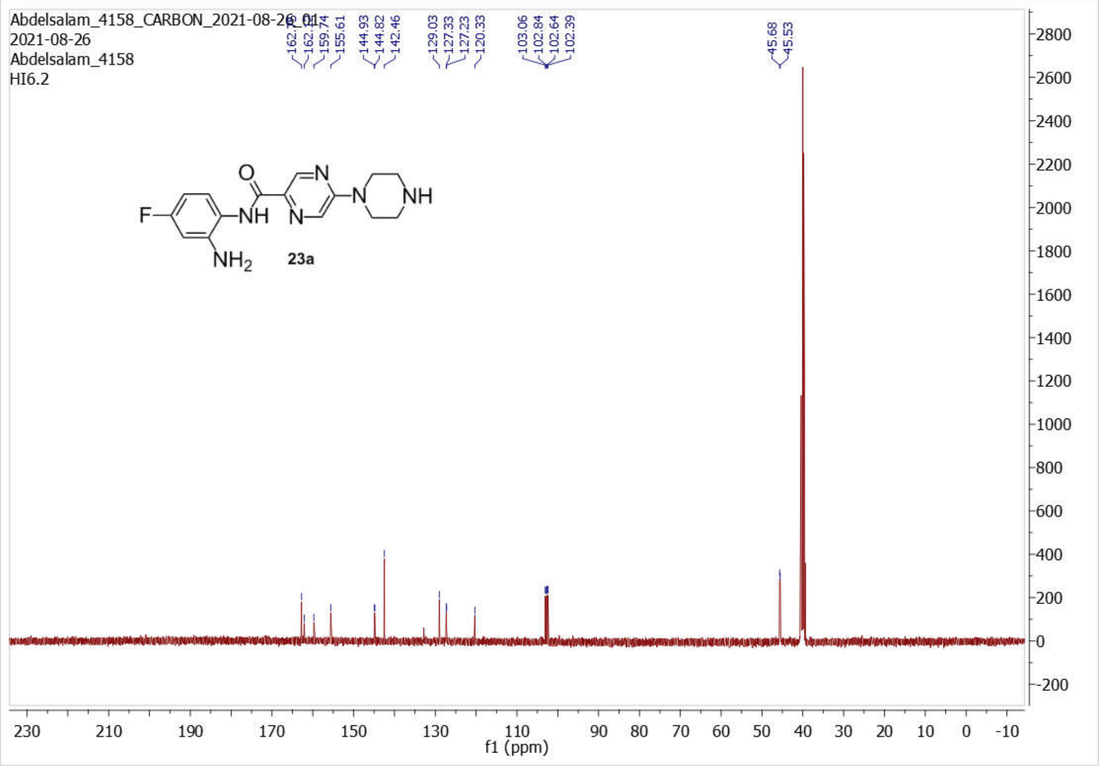

^{13}C *NMR chart of compound* **23c**

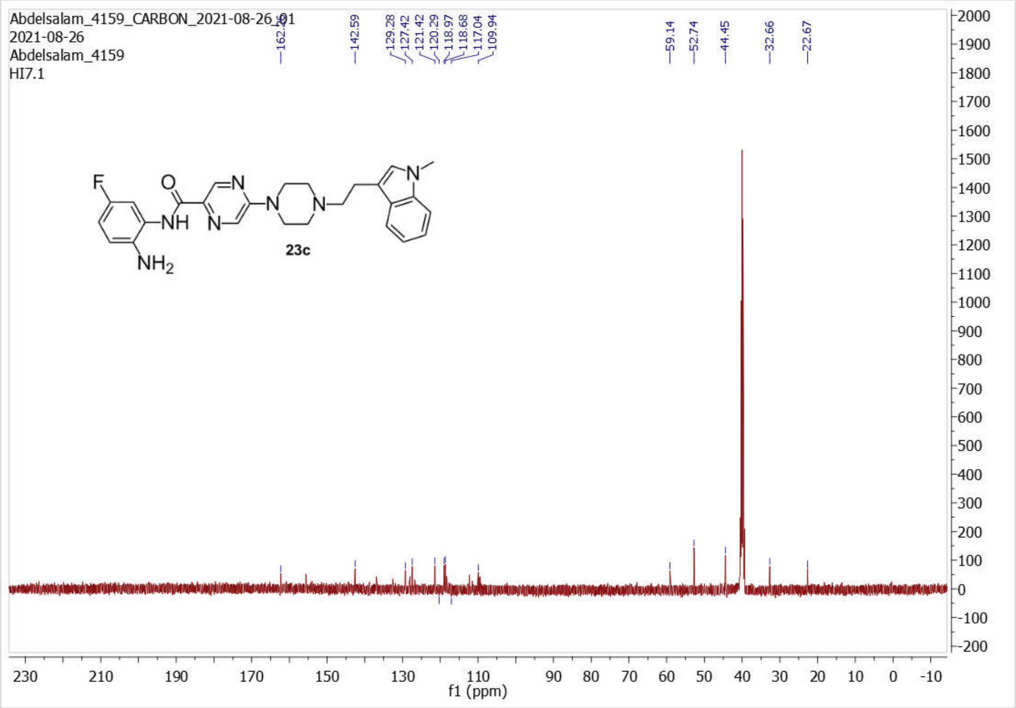

13C NMR chart of compound **25a**

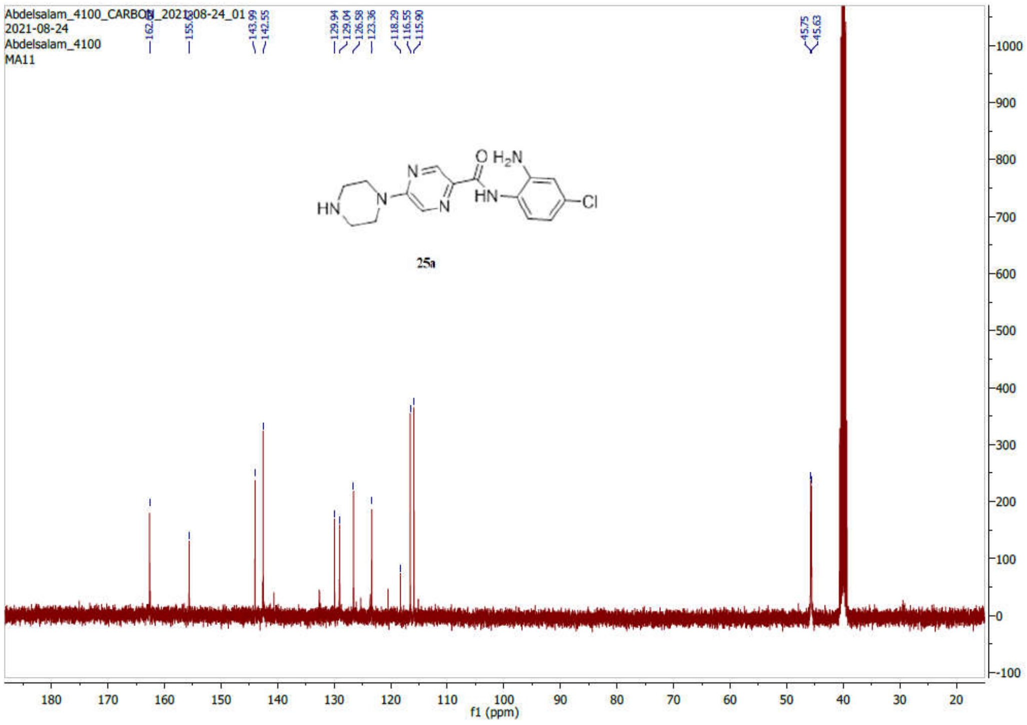

13C NMR chart of compound **25b**

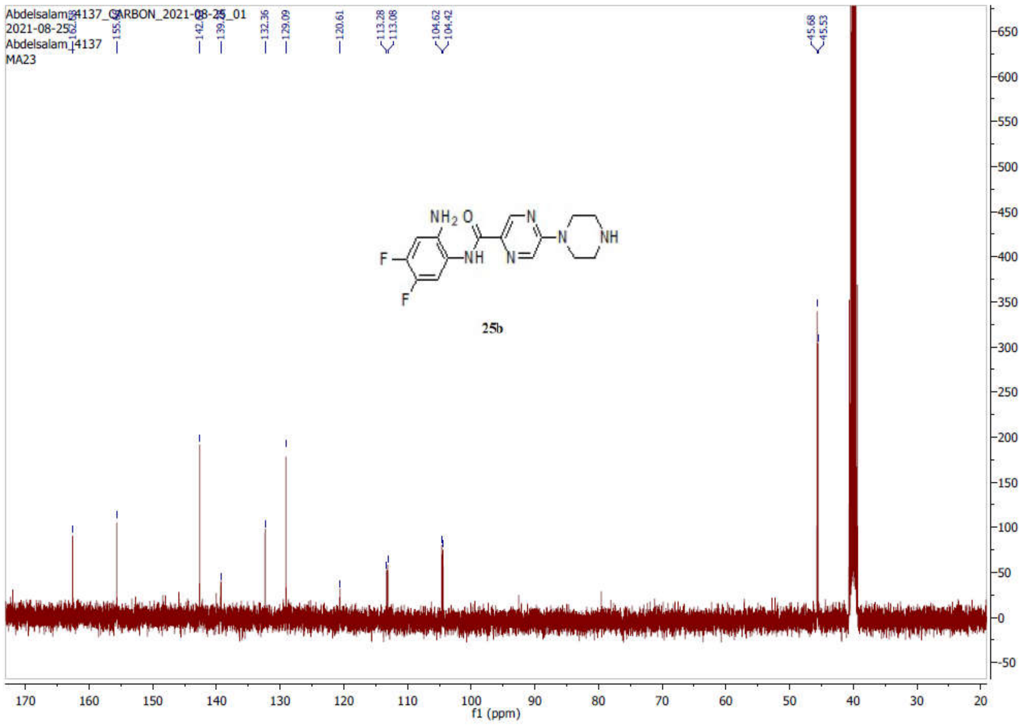

^{13}C *NMR chart of compound* **27a**

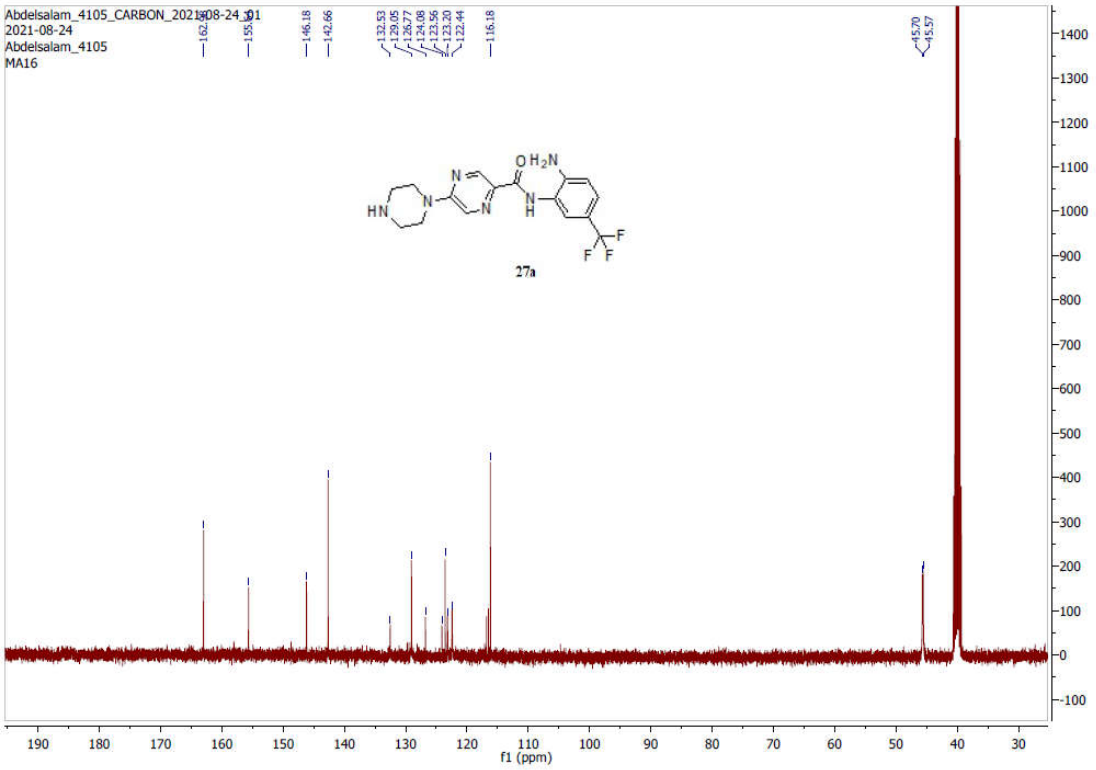

^{13}C *NMR chart of compound* **27b**

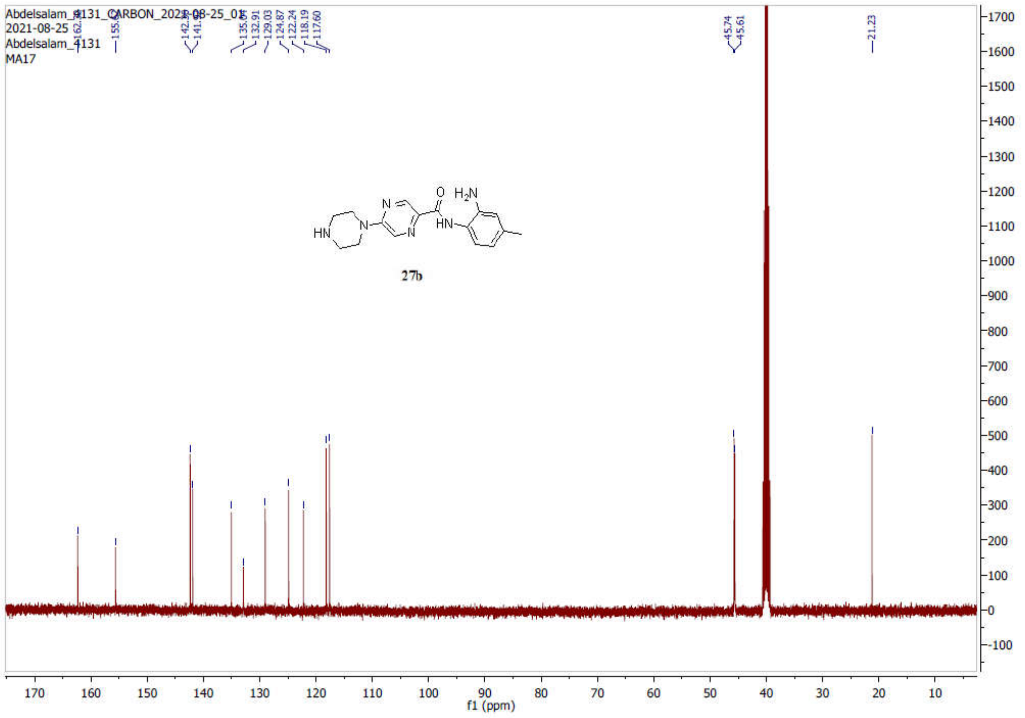

^{13}C *NMR chart of compound* **29a**

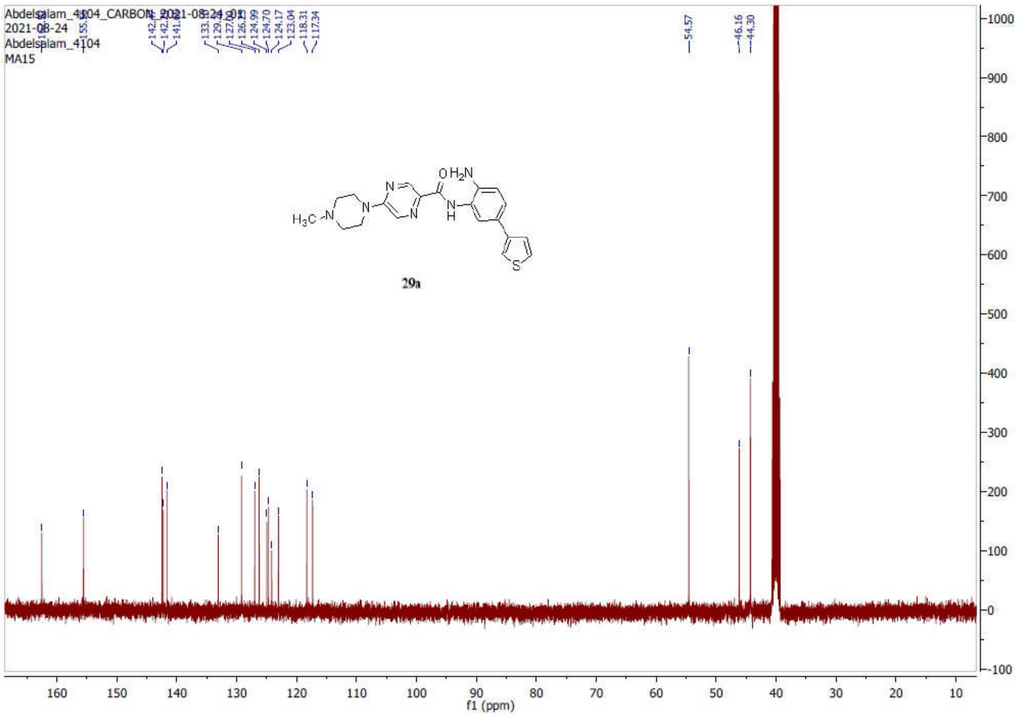

^{13}C *NMR chart of compound* **29b**

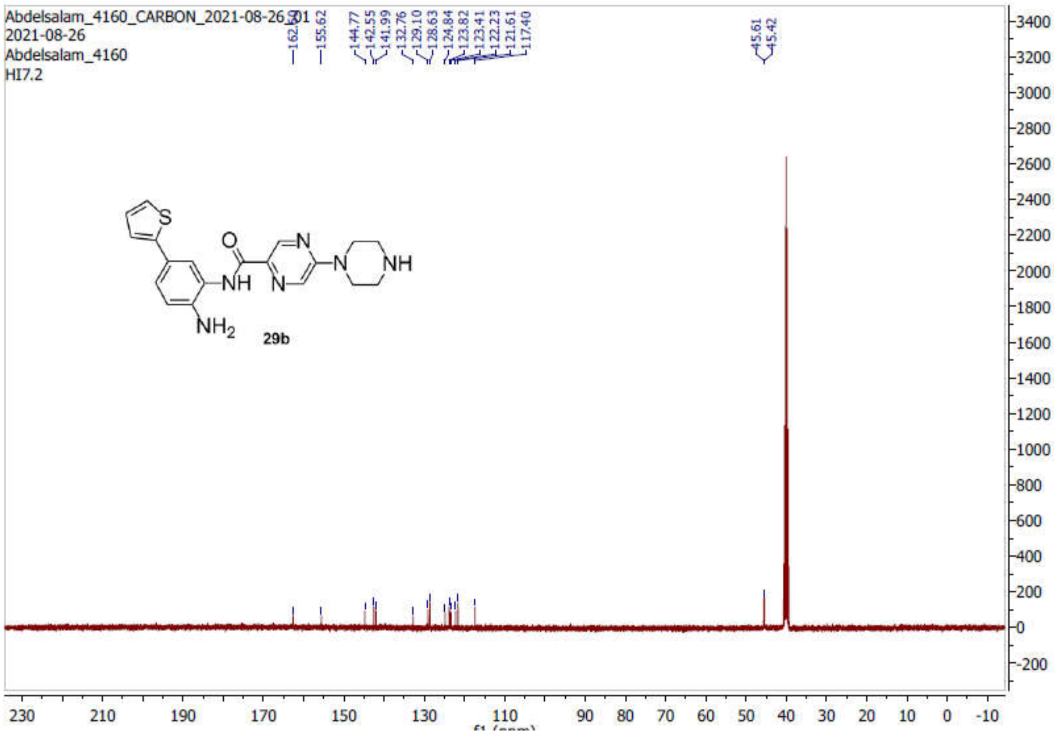

^{13}C NMR chart of compound **29c**

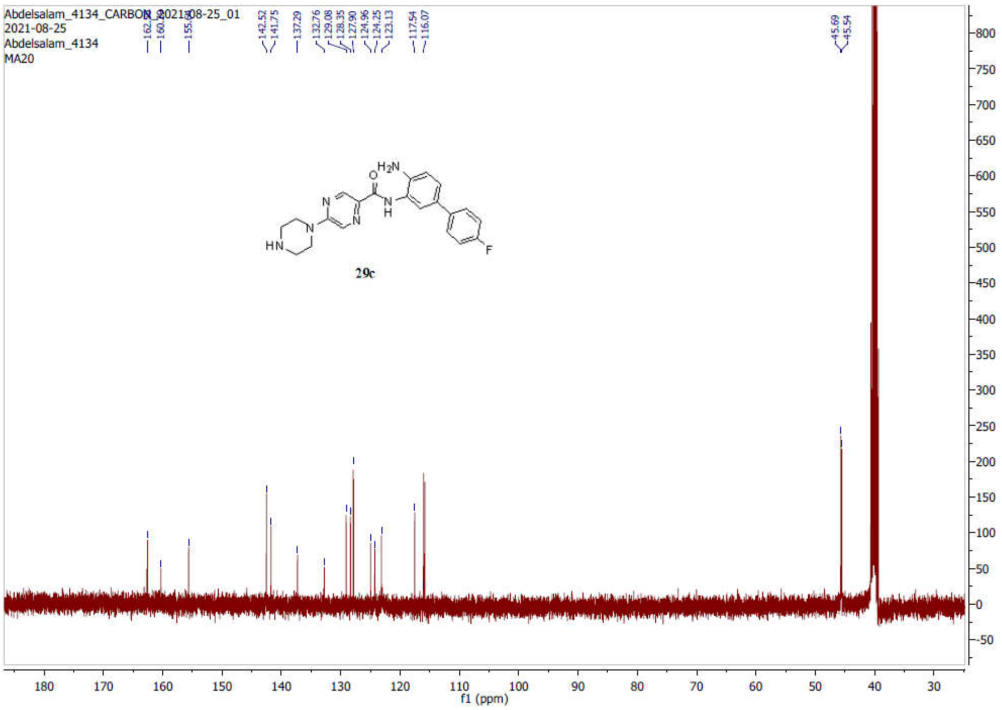

^{13}C NMR chart of compound **29d**

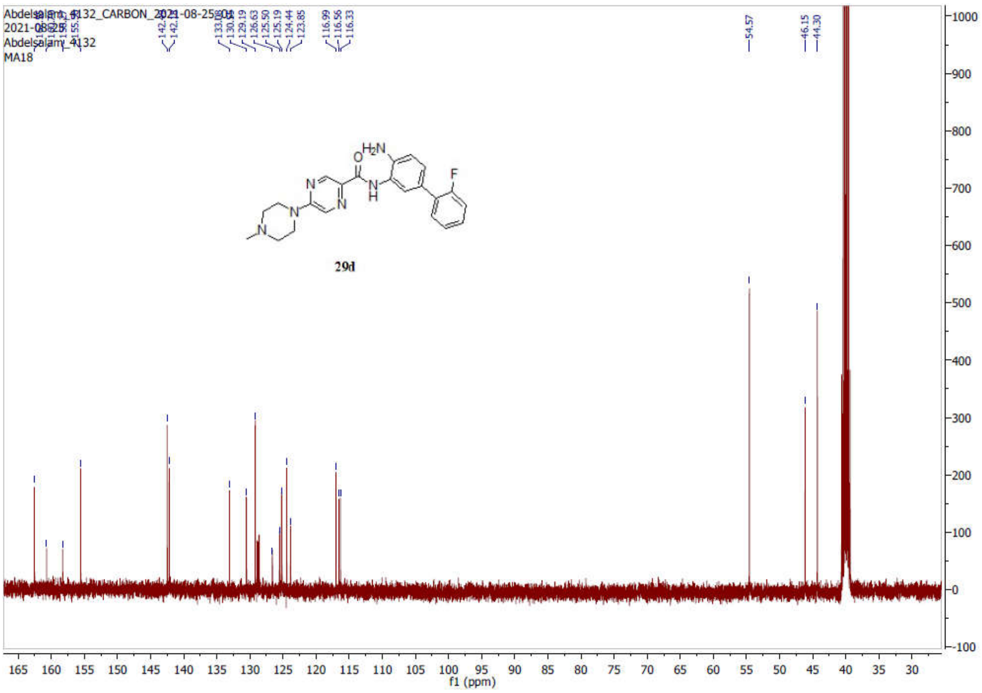

5. HRMS Spectra of final compounds

HRMS spectrum of compound **19a**

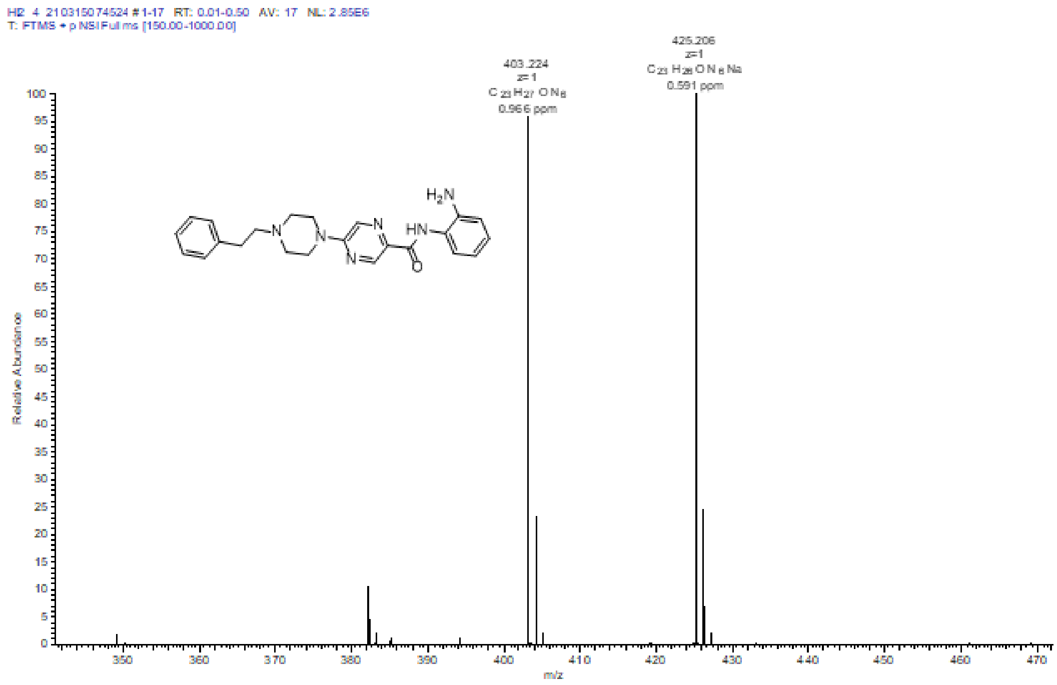

HRMS spectrum of compound **19b**

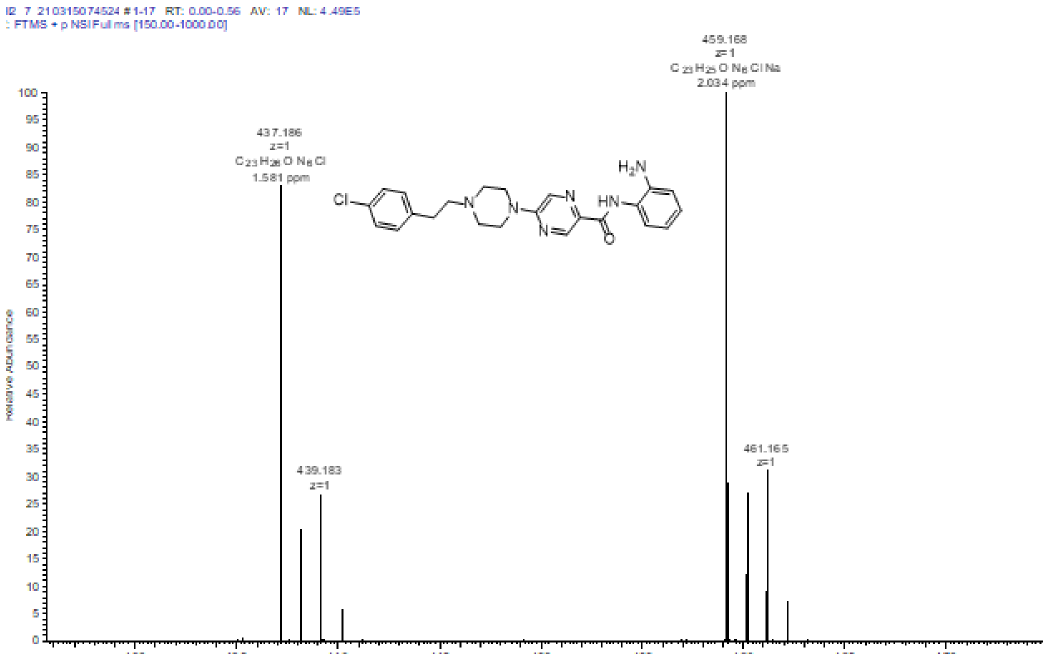

HRMS spectrum of compound **19c**

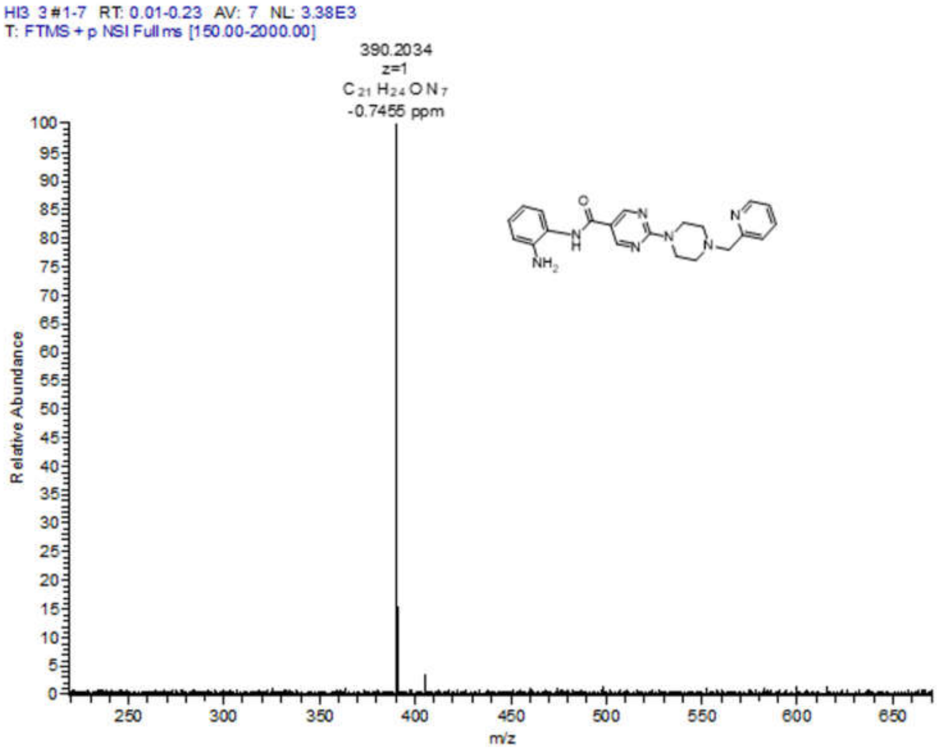

HRMS spectrum of compound **19d**

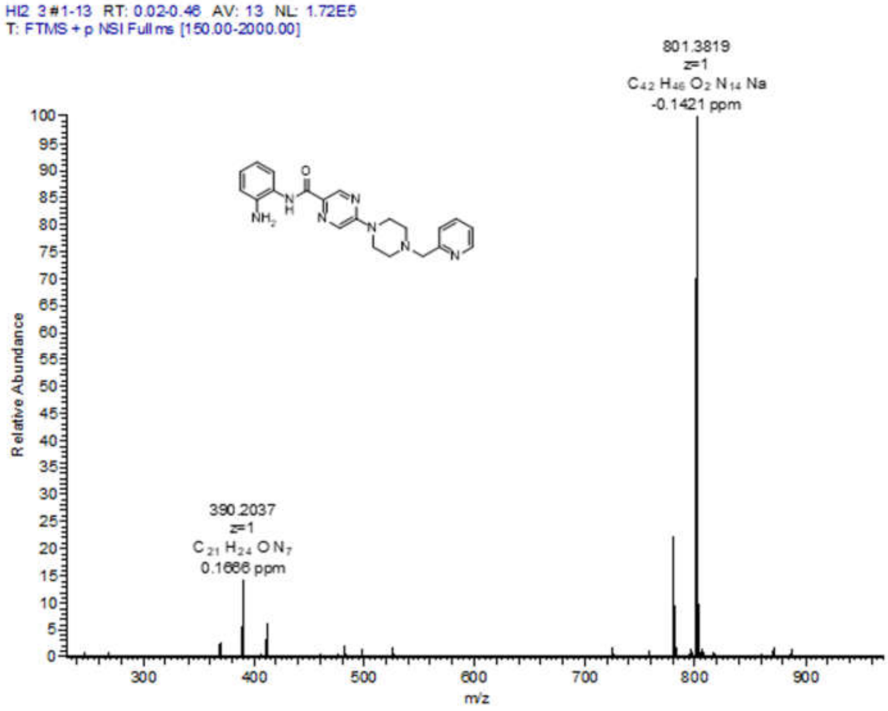

HRMS spectrum of compound **19e**

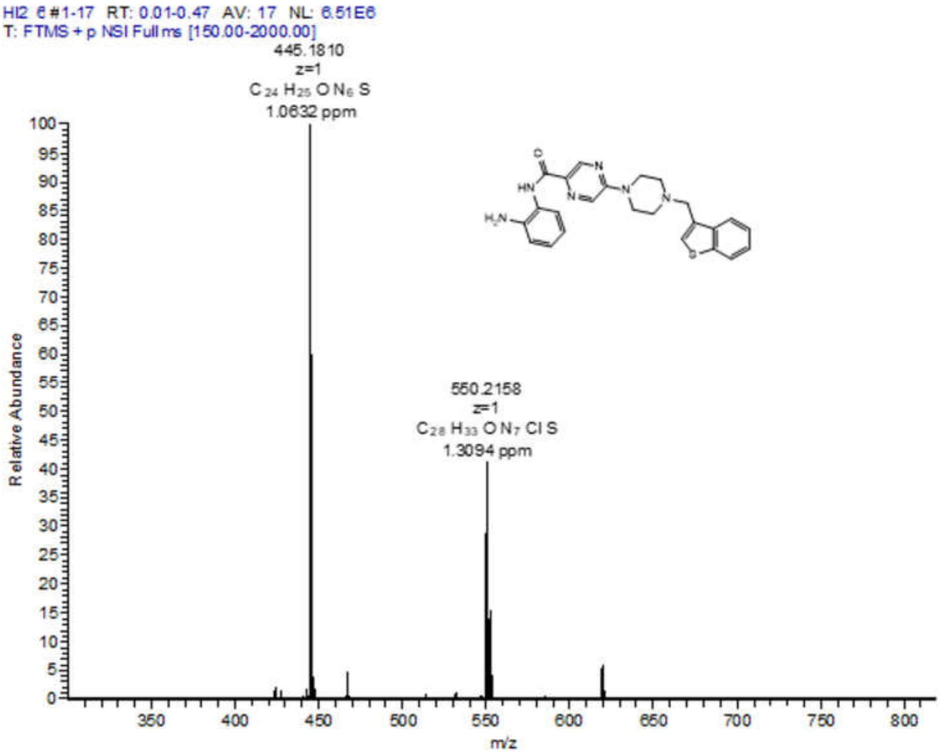

HRMS spectrum of compound **19f**

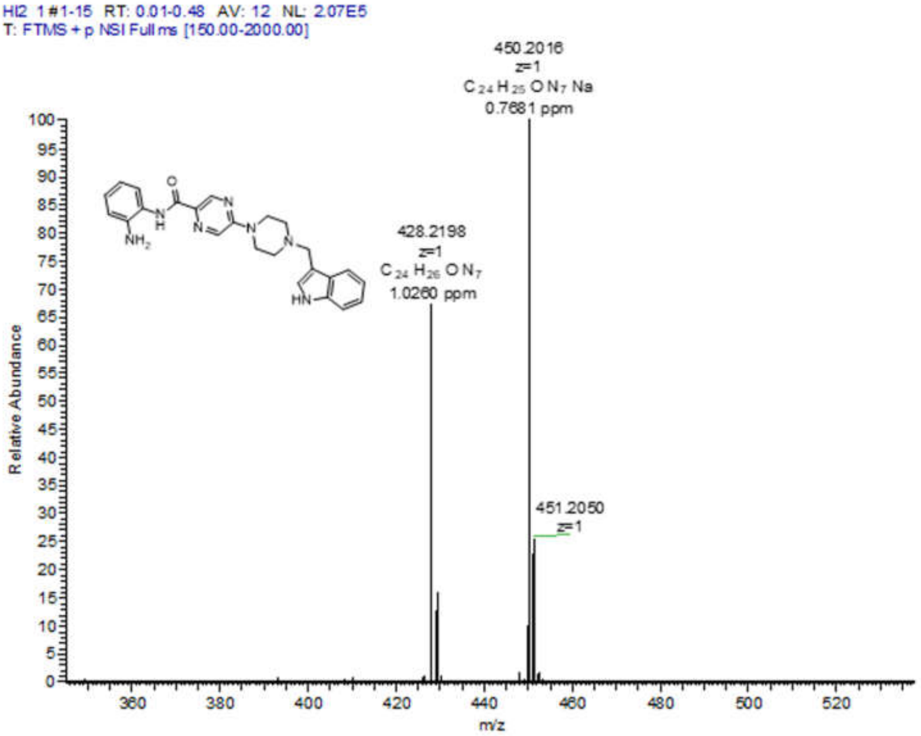

HRMS spectrum of compound **19g**

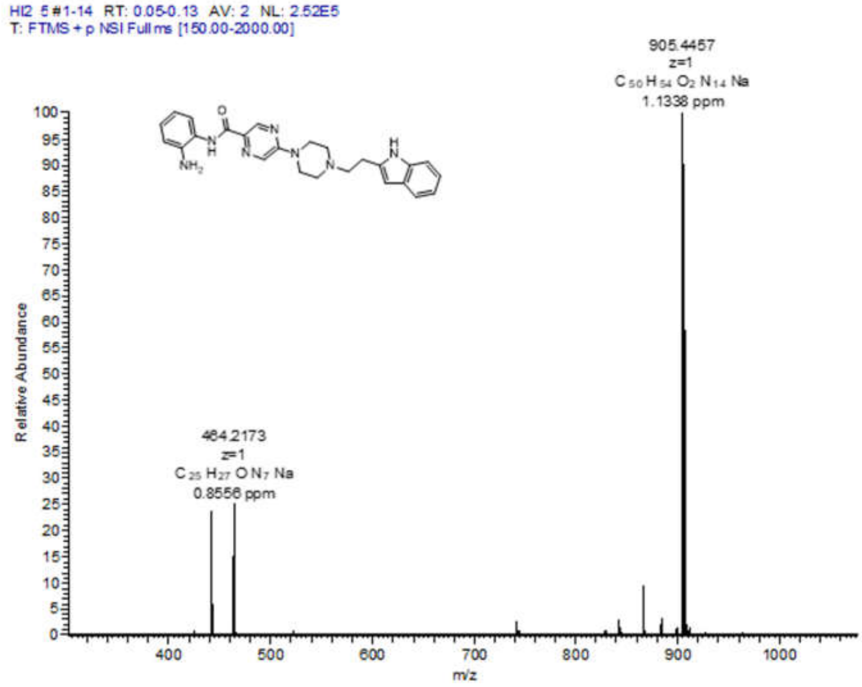

HRMS spectrum of compound **19h**

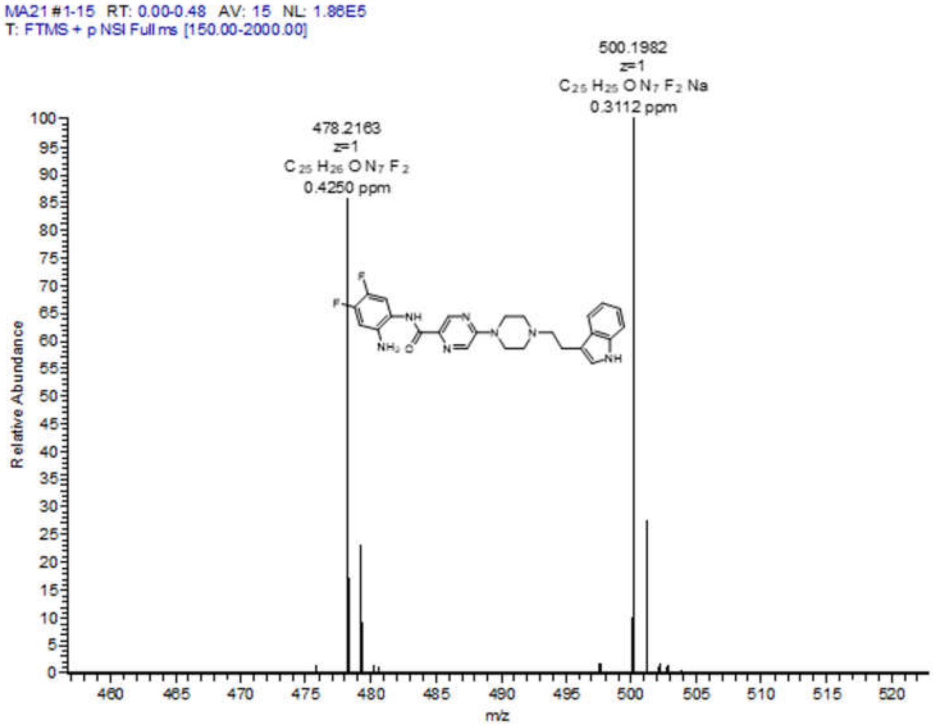

HRMS spectrum of compound **19i**

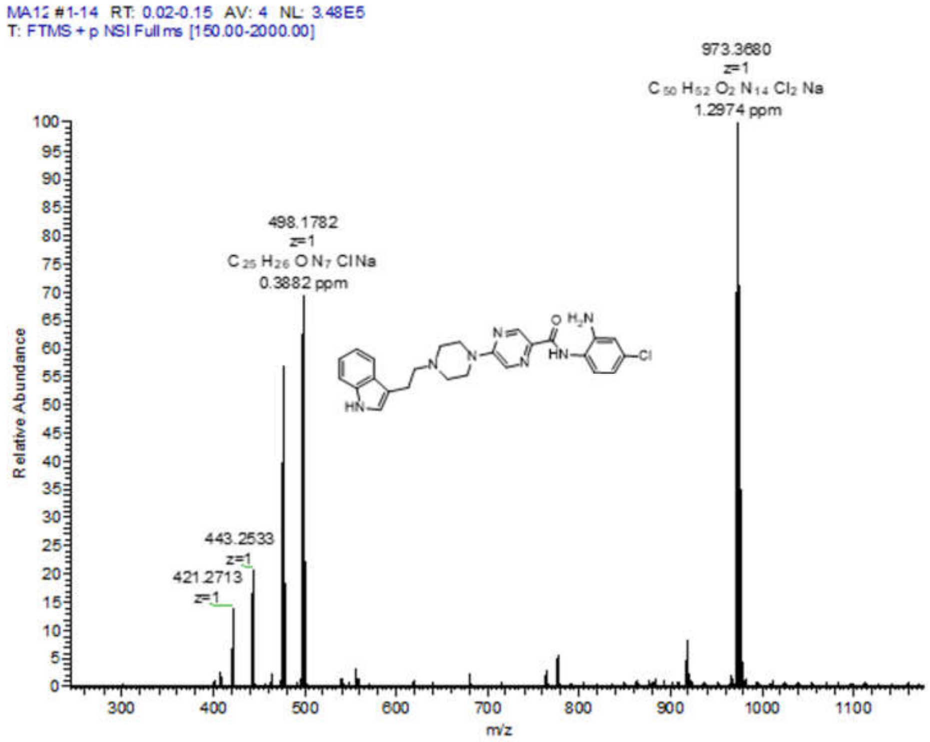

HRMS spectrum of compound **19j**

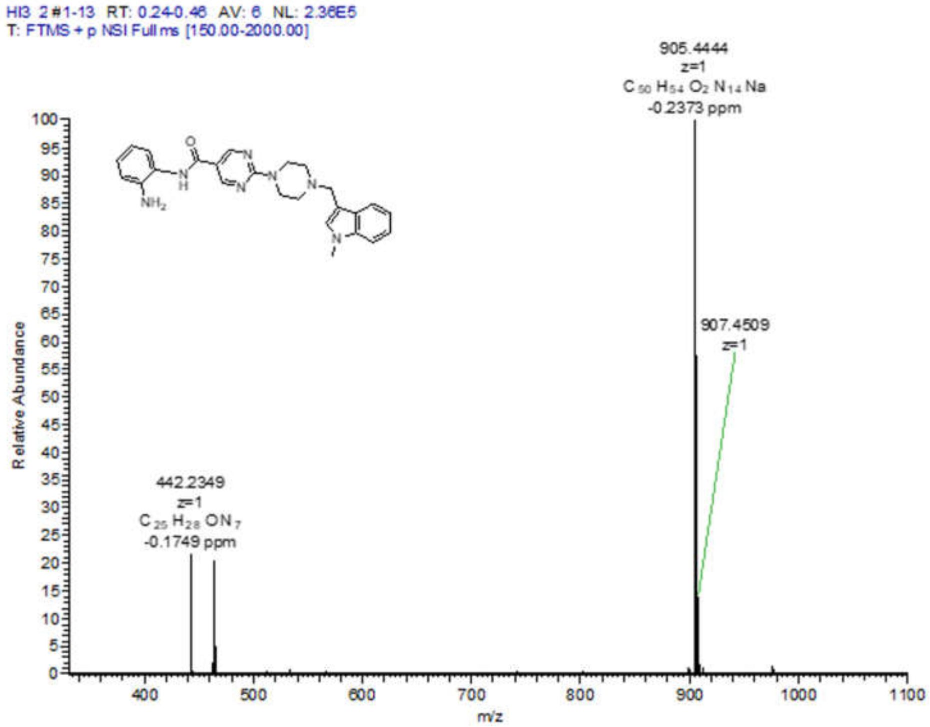

HRMS spectrum of compound **19k**

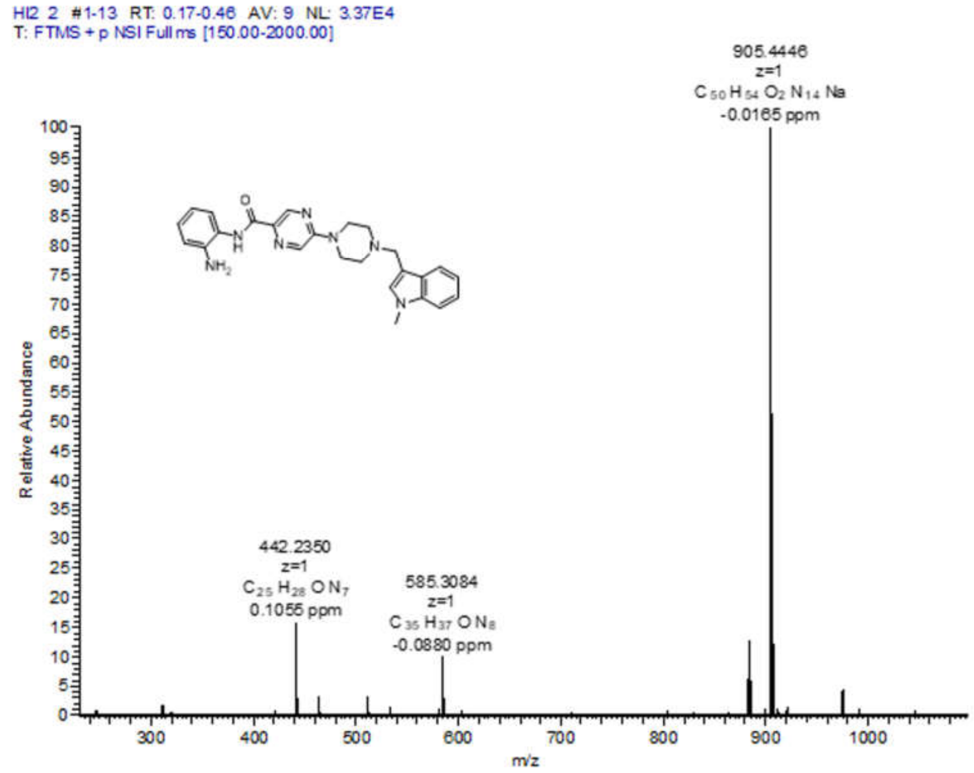

HRMS spectrum of compound **19l**

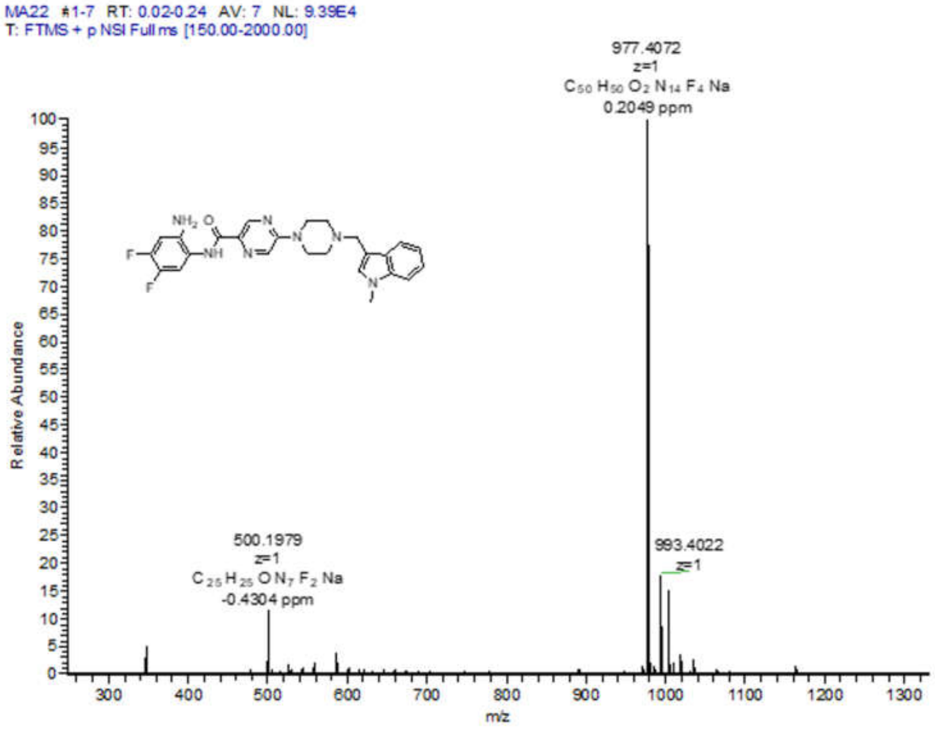

HRMS spectrum of compound **19m**

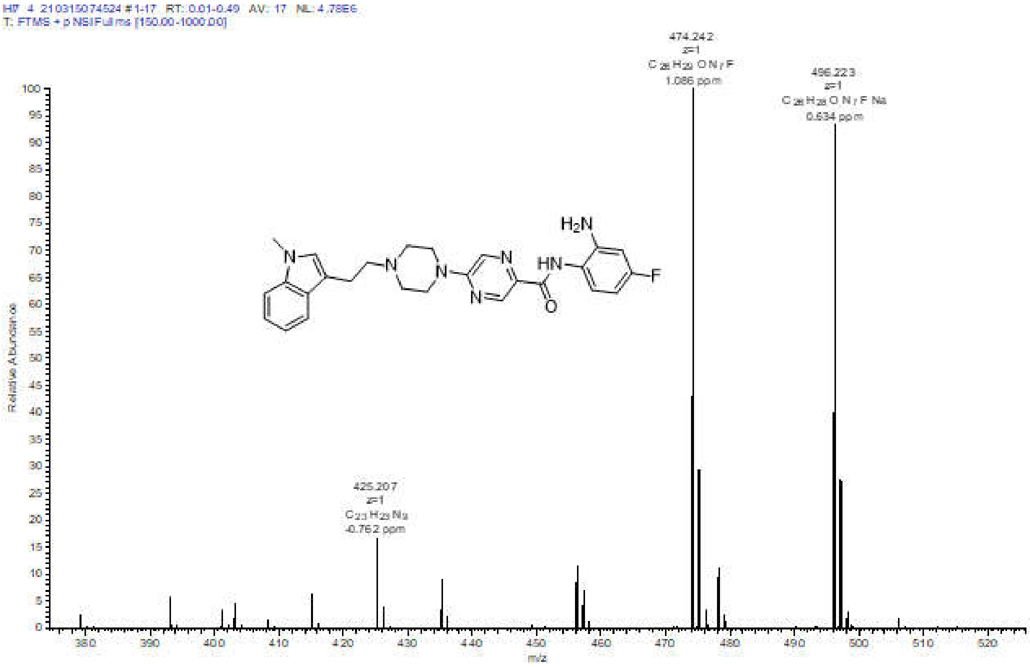

HRMS spectrum of compound **19n**

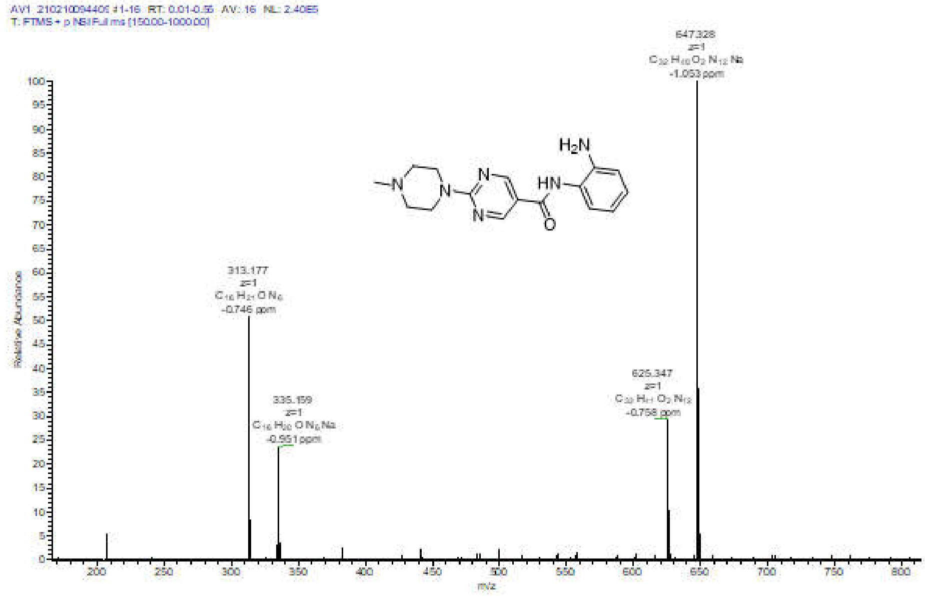

HRMS spectrum of compound **19o**

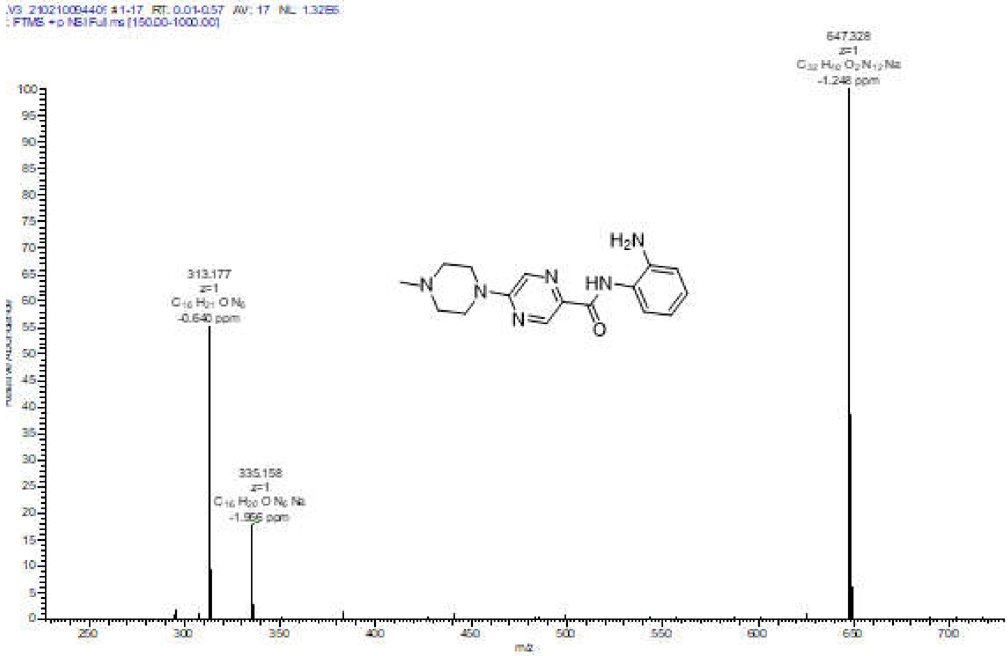

HRMS spectrum of compound **21a**

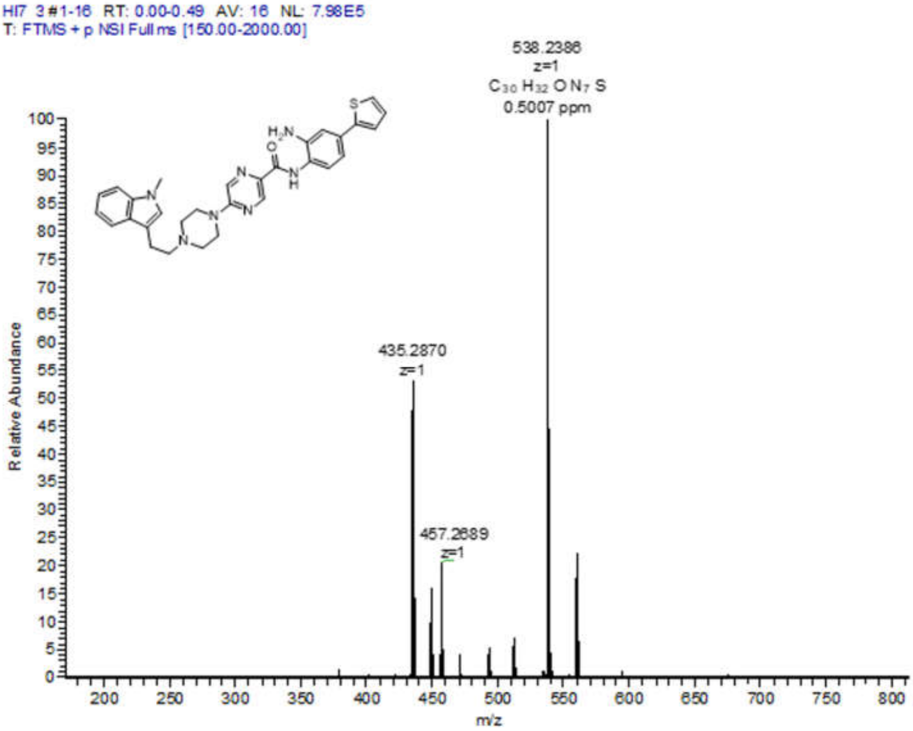

HRMS spectrum of compound **21b**

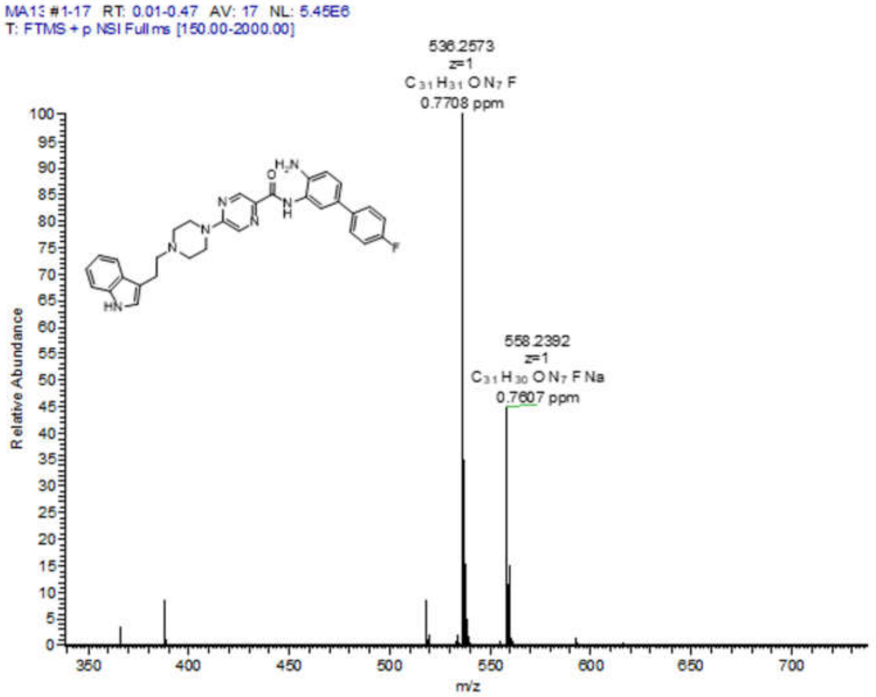

HRMS spectrum of compound **21c**

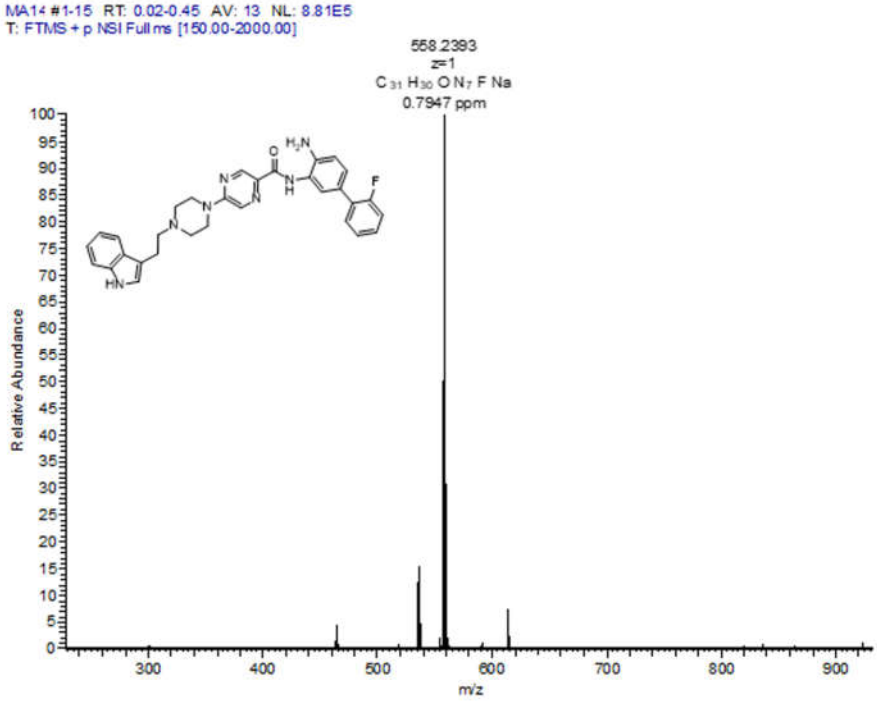

HRMS spectrum of compound **23a**

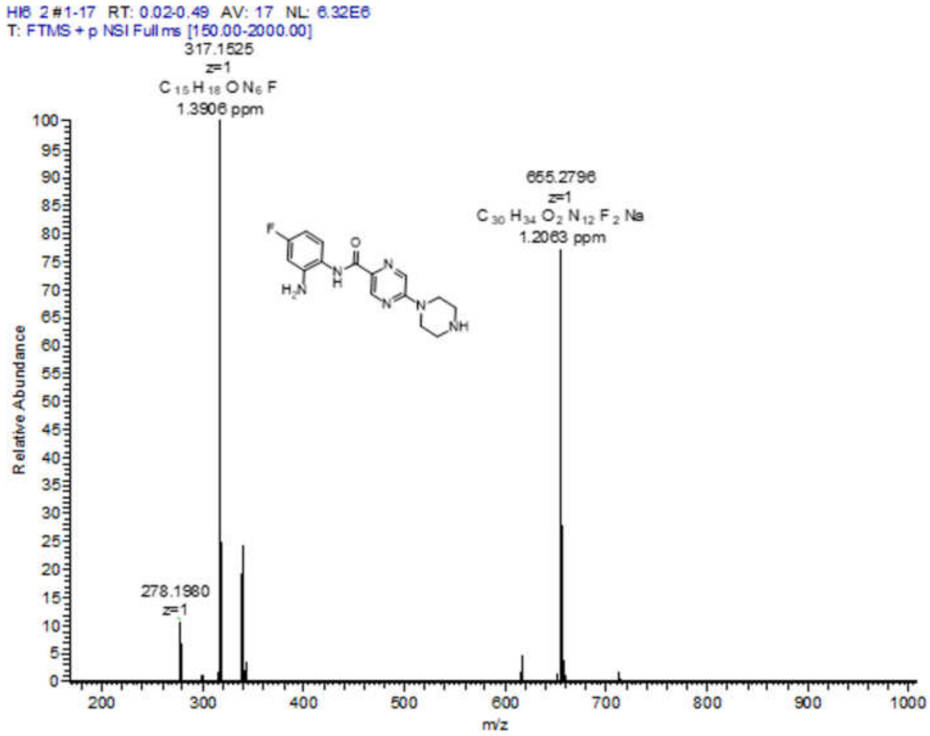

HRMS spectrum of compound **23b**

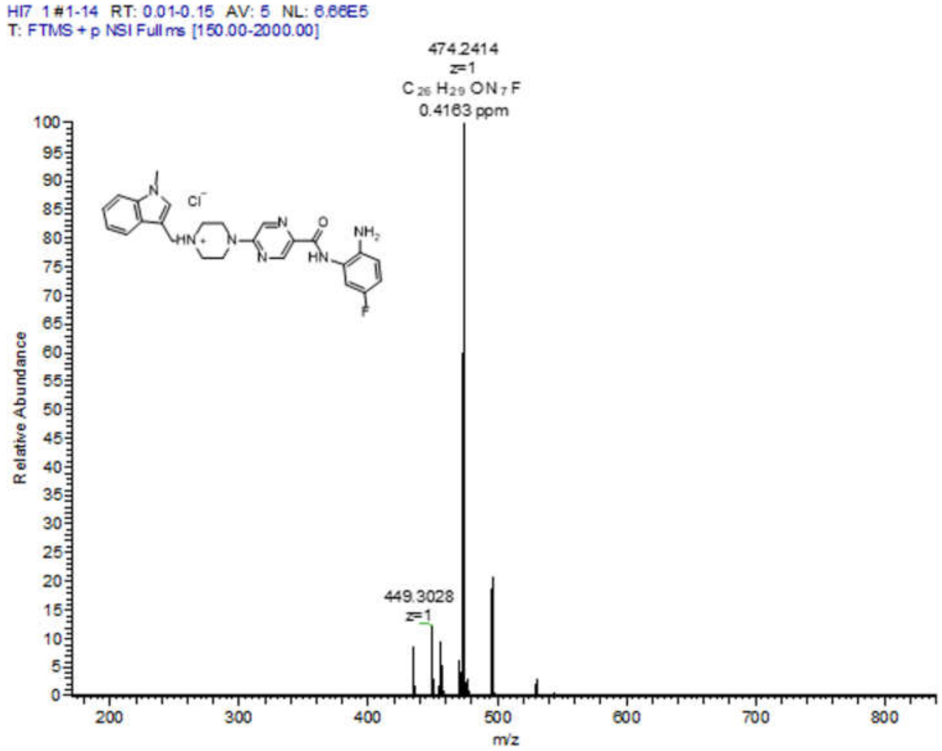

HRMS spectrum of compound **23c**

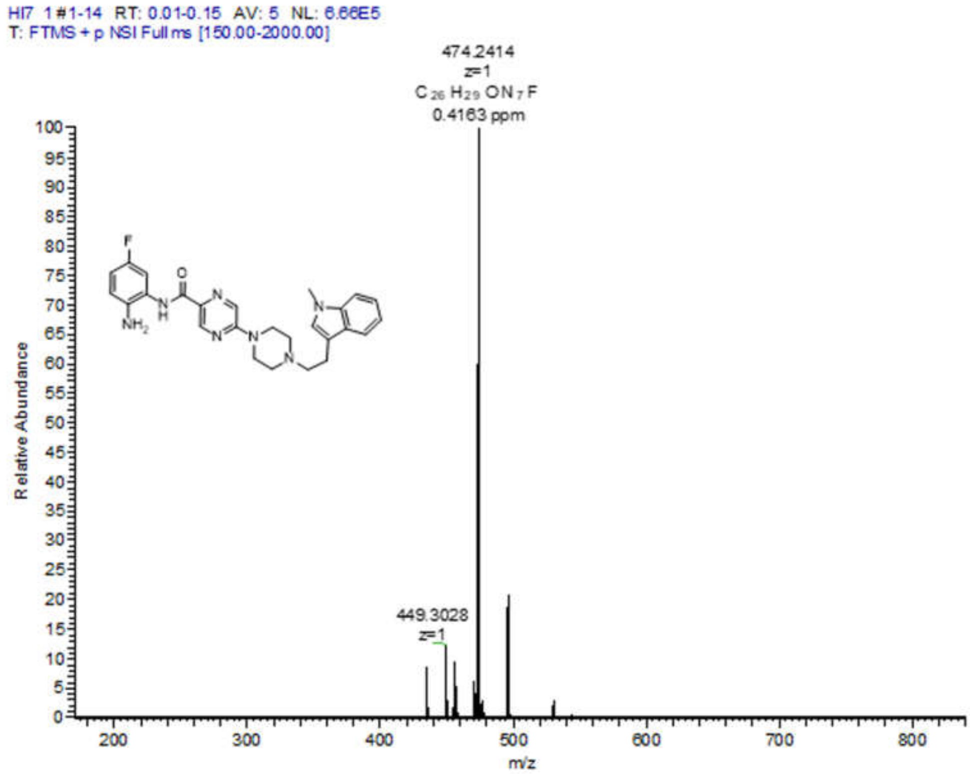

HRMS spectrum of compound **25a**

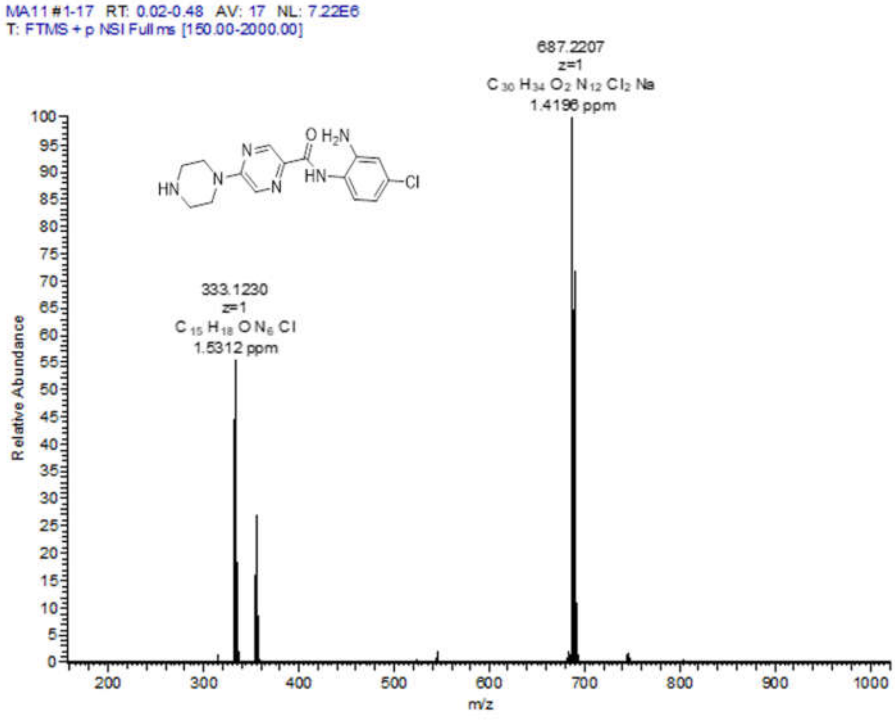

HRMS spectrum of compound **25b**

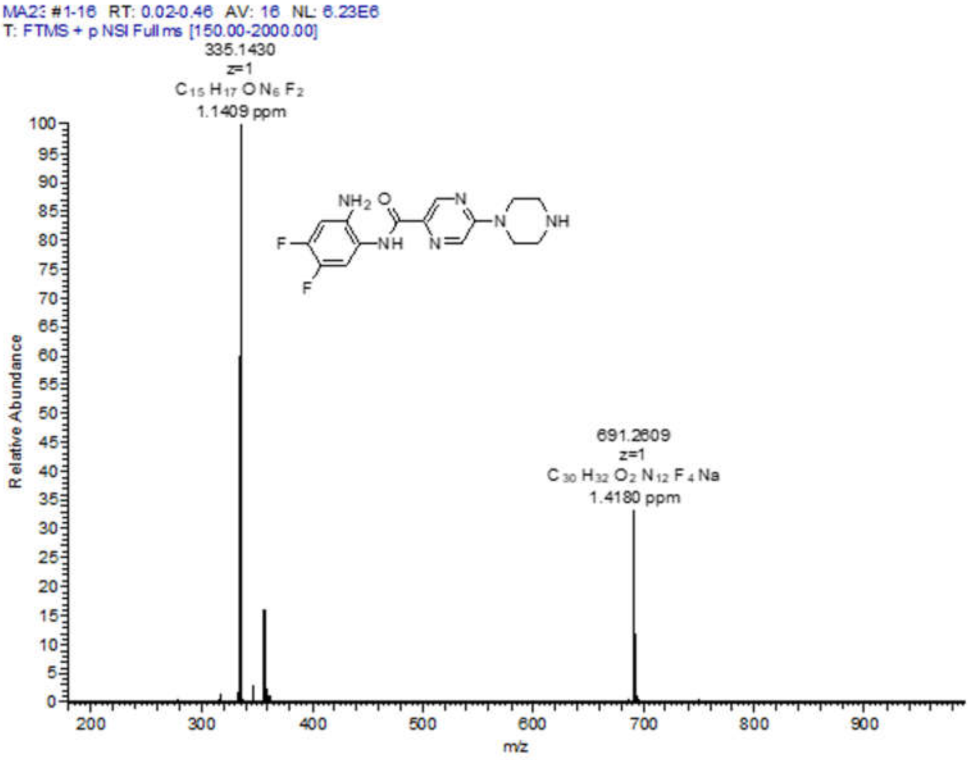

HRMS spectrum of compound **27a**

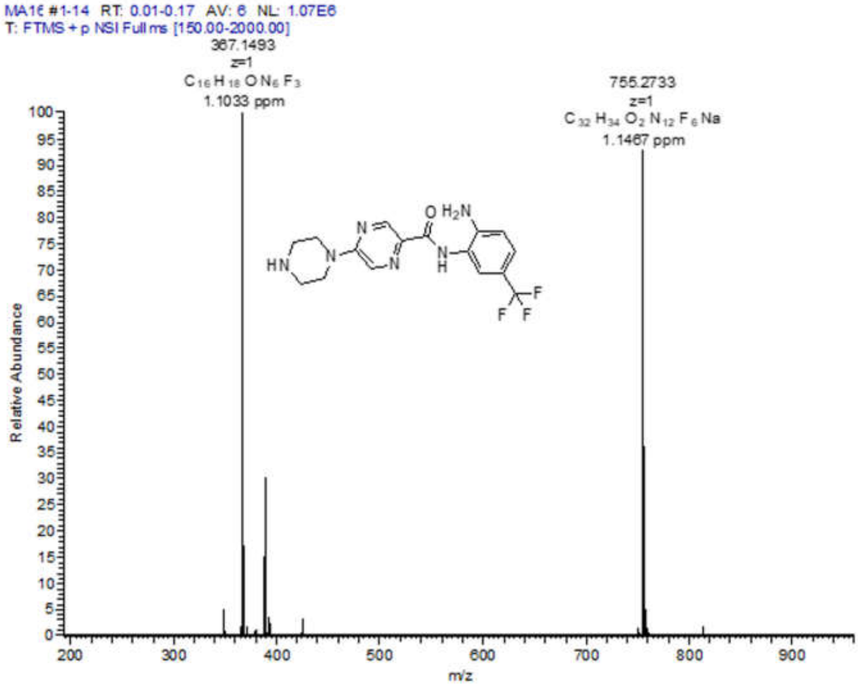

HRMS spectrum of compound **27b**

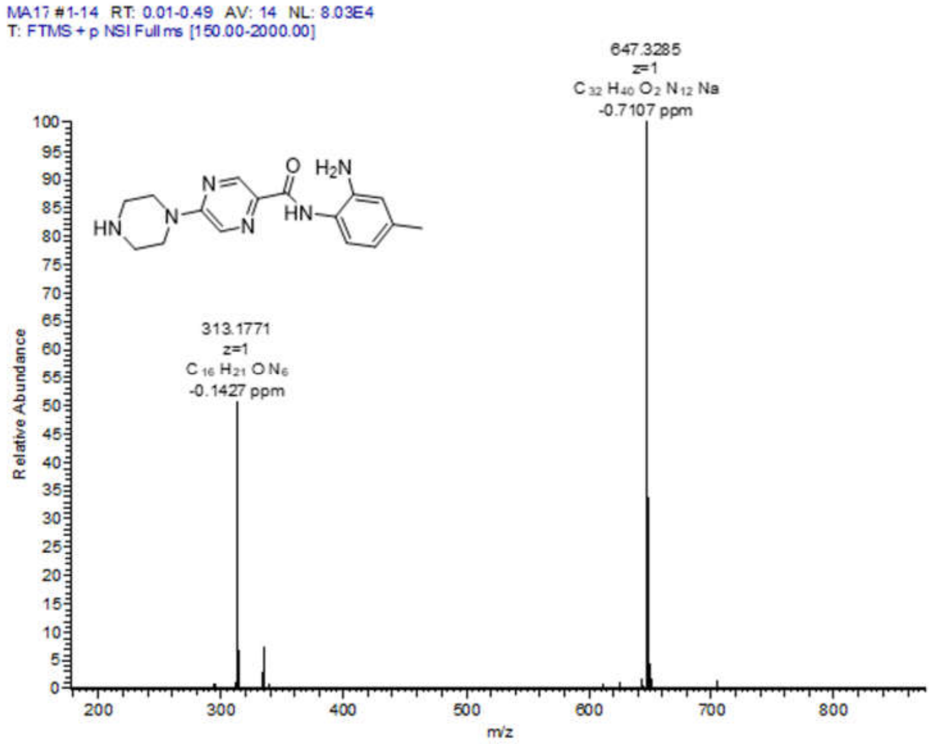

HRMS spectrum of compound **27c**

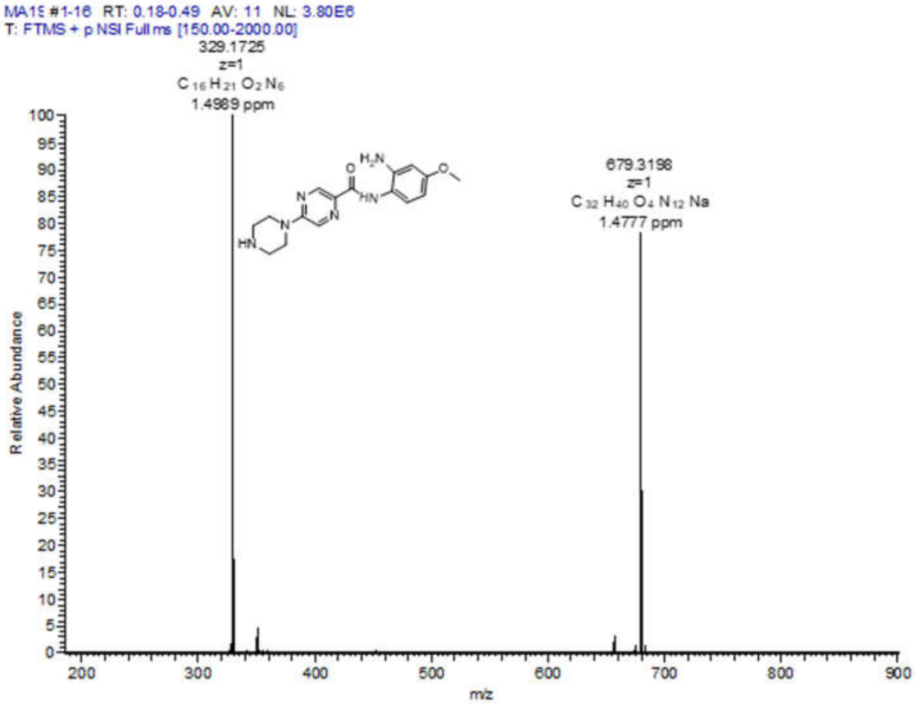

HRMS spectrum of compound **29a**

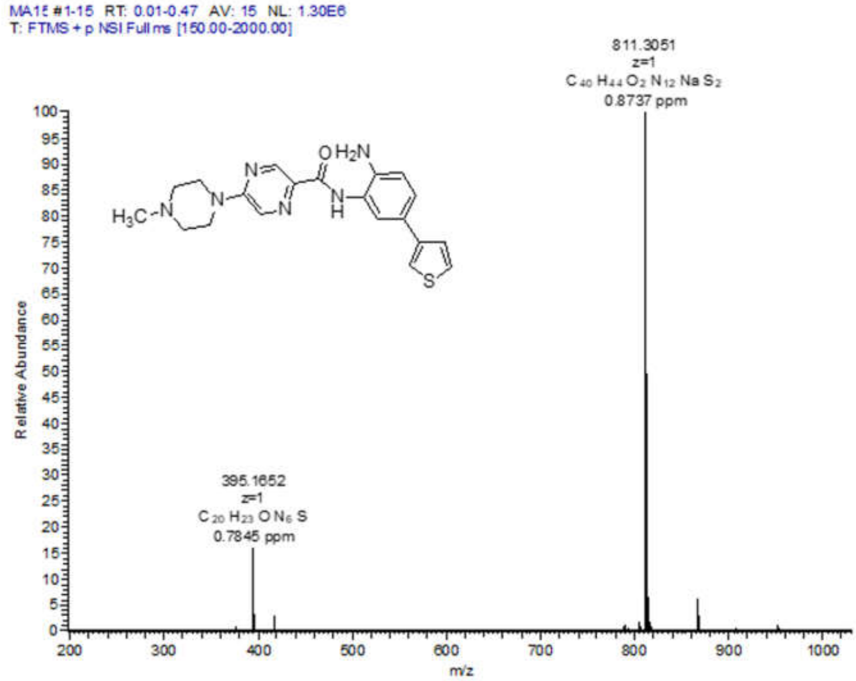

HRMS spectrum of compound **29c**

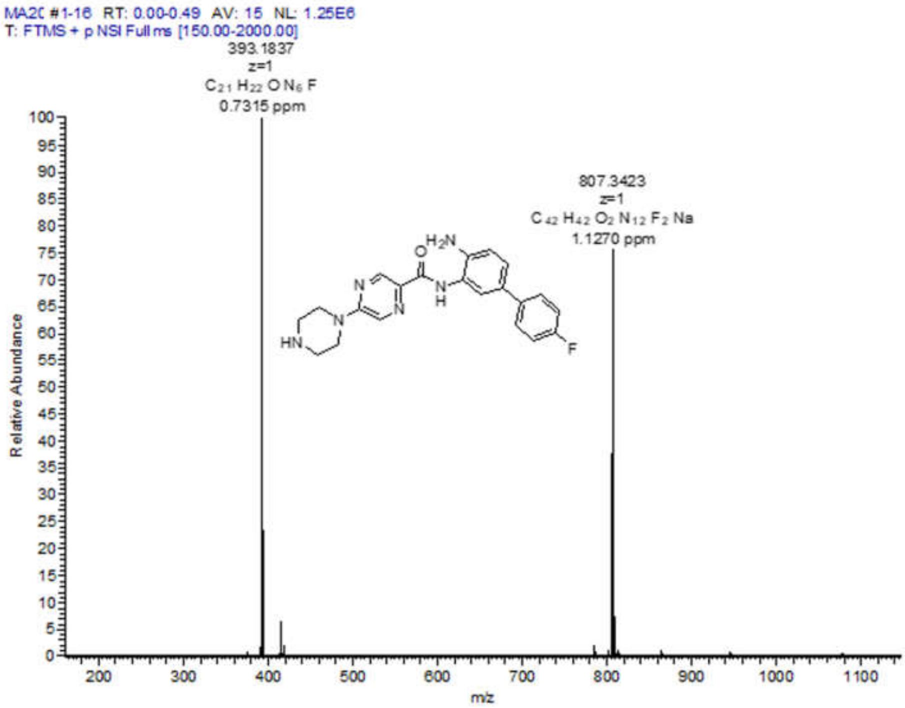

HRMS spectrum of compound **29d**

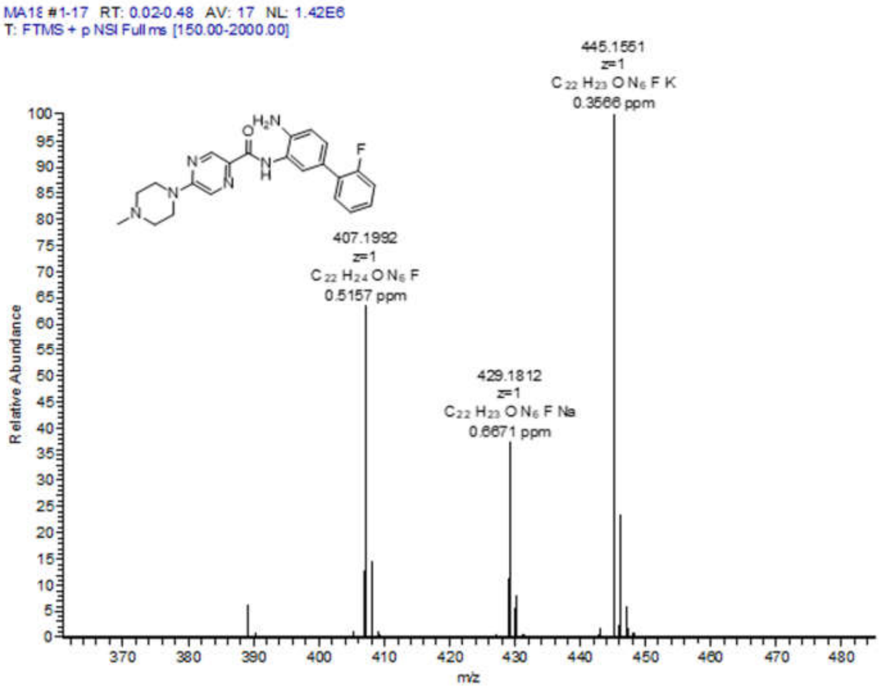

HPLC Spectra of final compounds

HPLC chromatogram of compound **19a**

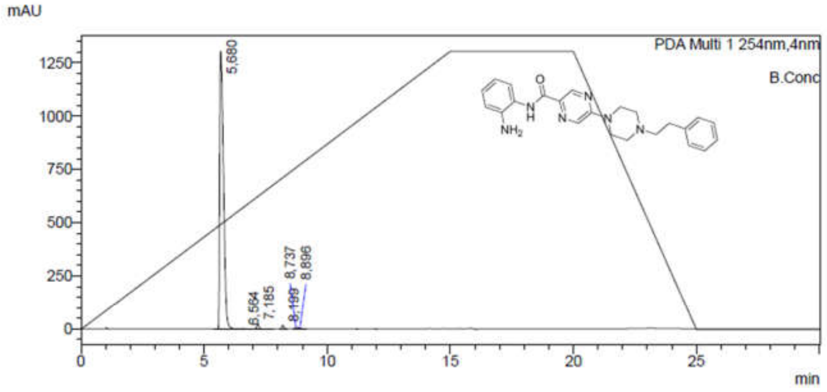

PDA Ch1 254nm

Peak#	Ret. Time	Area	Height	Area%
1	5,680	14020216	1303072	97,581
2	6,564	22049	2598	0,153
3	7,185	146661	24357	1,021
4	8,199	100938	16769	0,703
5	8,737	47073	7946	0,328
6	8,896	30808	3600	0,214
Total		14367745	1358341	100,000

HPLC chromatogram of compound **19b**

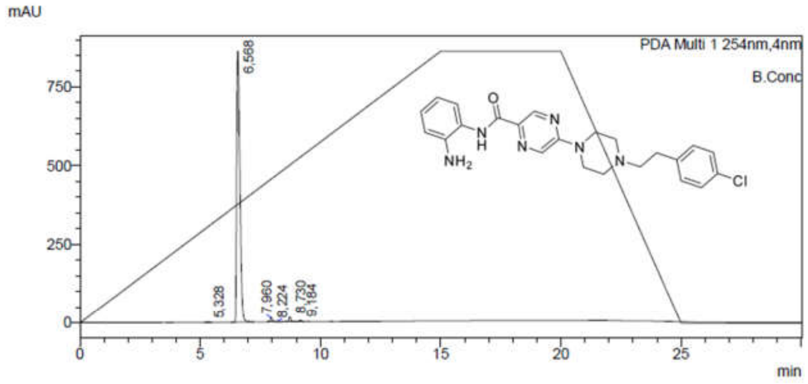

PDA Ch1 254nm

Peak#	Ret. Time	Area	Height	Area%
1	5,328	11941	1860	0,145
2	6,568	8008030	832627	96,981
3	7,960	88470	14084	1,071
4	8,224	17387	2611	0,211
5	8,730	103086	15409	1,248
6	9,184	28418	4529	0,344
Total		8257332	871120	100,000

HPLC chromatogram of compound **19c**

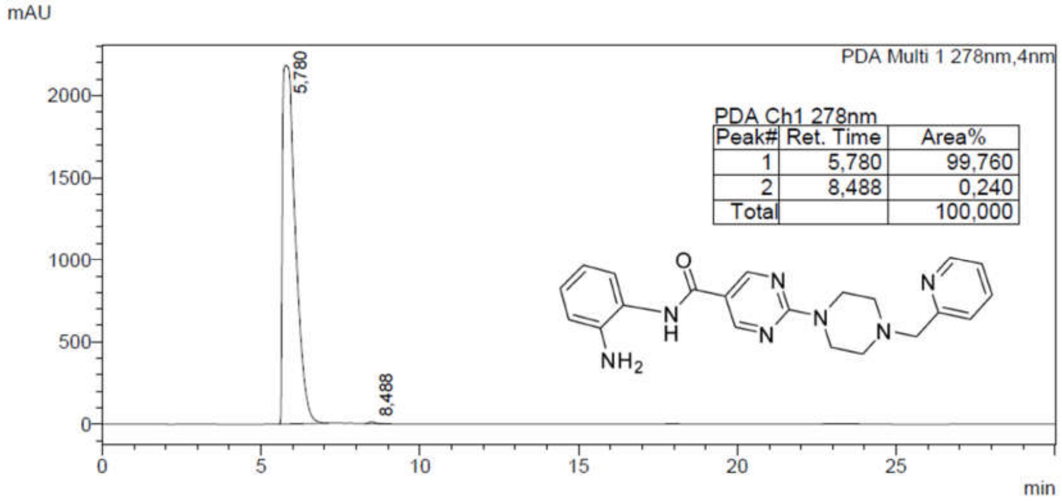

PDA Ch1 278nm

Peak#	Ret. Time	Area%
1	5,780	99,760
2	8,488	0,240
Total		100,000

HPLC chromatogram of compound **19d**

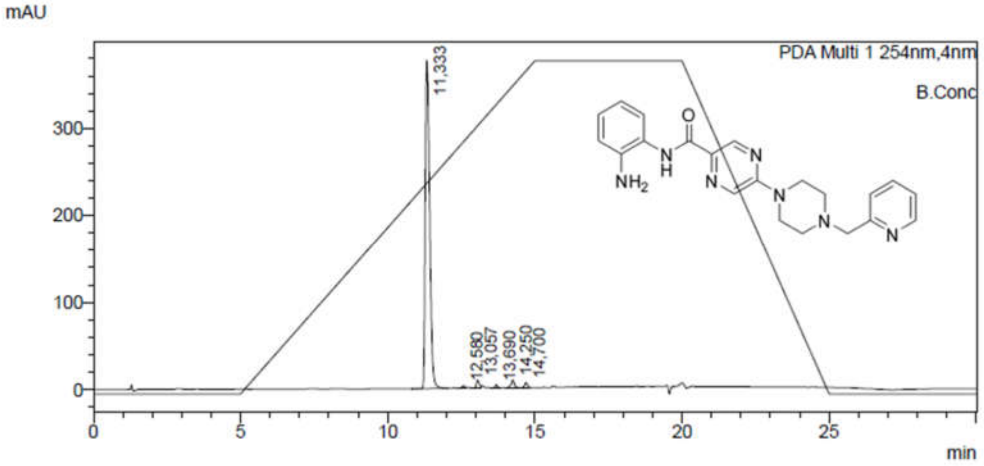

PDA Ch1 254nm

Peak#	Ret. Time	Area%
1	11,333	94,560
2	12,580	0,495
3	13,057	1,536
4	13,690	0,507
5	14,250	1,859
6	14,700	1,042
Total		100,000

HPLC chromatogram of compound **19e**

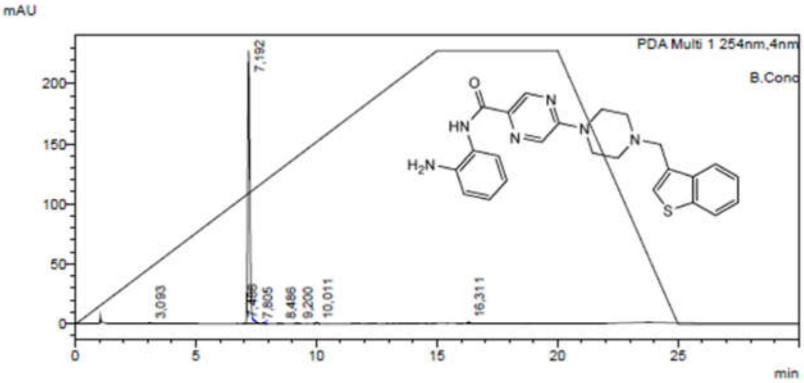

PDA Ch1 254nm

Peak#	Ret. Time	Area	Height	Area%
1	3,093	5265	790	0,363
2	7,192	1412248	227399	97,459
3	7,488	1845	602	0,127
4	7,805	3560	659	0,246
5	8,486	5535	962	0,382
6	9,200	5392	917	0,372
7	10,011	8778	1499	0,606
8	16,311	6454	1589	0,445
Total		1449076	234416	100,000

HPLC chromatogram of compound **19f**

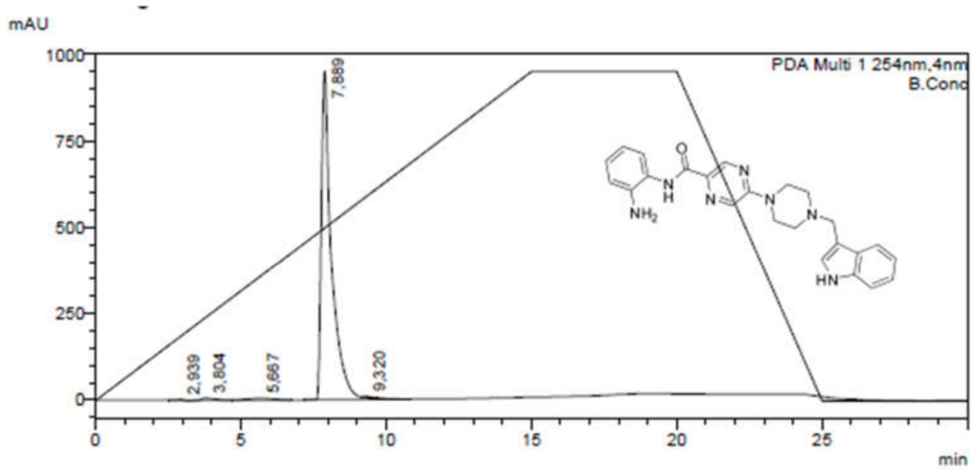

<Peak Table>

PDA Ch1 254nm

Peak#	Ret. Time	Area%	Area	Height
1	2,939	0,159	37795	2486
2	3,804	0,907	215671	6630
3	5,667	1,177	279920	5229
4	7,889	97,580	23205386	949557
5	9,320	0,177	42009	2865
Total		100,000	23780781	966767

HPLC chromatogram of compound **19g**

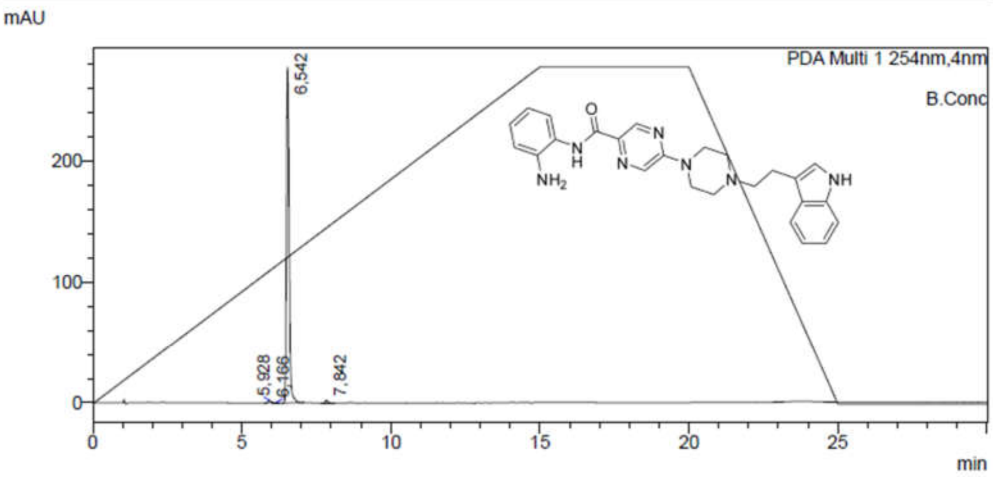

PDA Ch1 254nm

Peak#	Ret. Time	Area	Height	Area%
1	5,928	11302	1871	0,642
2	6,166	2777	526	0,158
3	6,542	1730349	263529	98,333
4	7,842	15249	2227	0,867
Total		1759677	268154	100,000

HPLC Chromatogram of compound **19h**

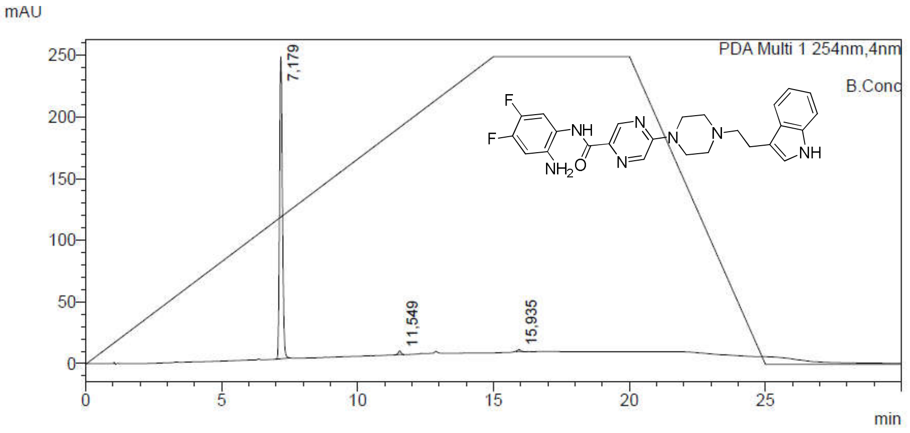

PDA Ch1 254nm

Peak#	Ret. Time	Area	Height	Area%
1	7,179	1800773	245095	98,294
2	11,549	20926	2983	1,142
3	15,935	10338	1526	0,564
Total		1832036	249604	100,000

HPLC Chromatogram of compound **19i**

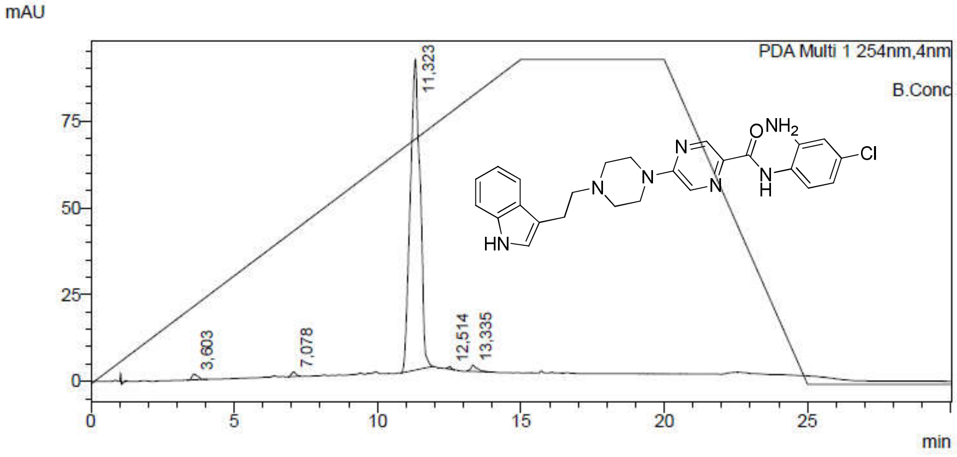

PDA Ch1 254nm

Peak#	Ret. Time	Area	Height	Area%
1	3,603	23128	1645	1,049
2	7,078	13908	1323	0,631
3	11,323	2135129	89492	96,871
4	12,514	5128	741	0,233
5	13,335	26807	1738	1,216
Total		2204100	94939	100,000

HPLC Chromatogram of compound **19j**

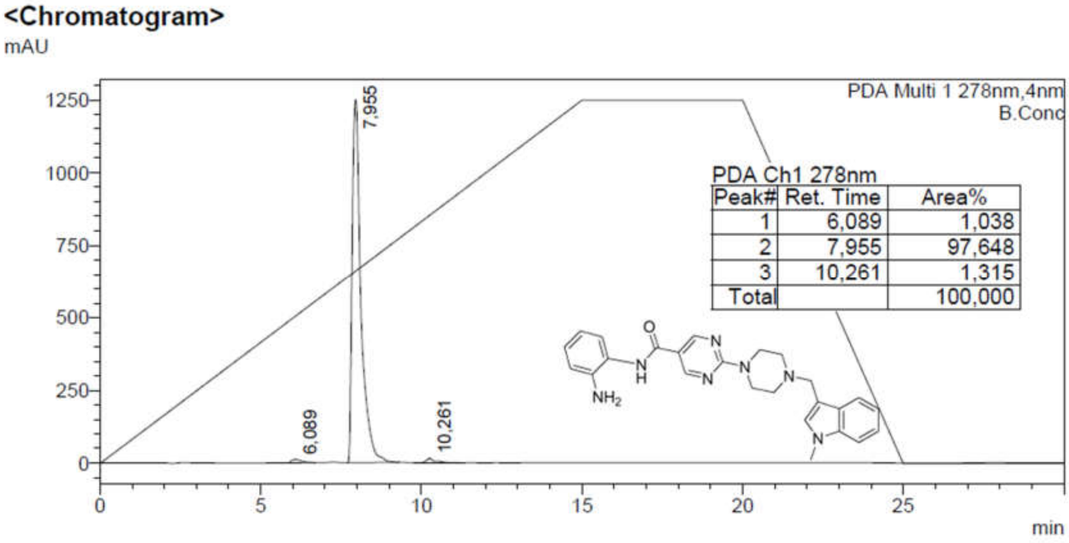

PDA Ch1 278nm

Peak#	Ret. Time	Area%
1	6,089	1,038
2	7,955	97,648
3	10,261	1,315
Total		100,000

HPLC Chromatogram of compound **19k**

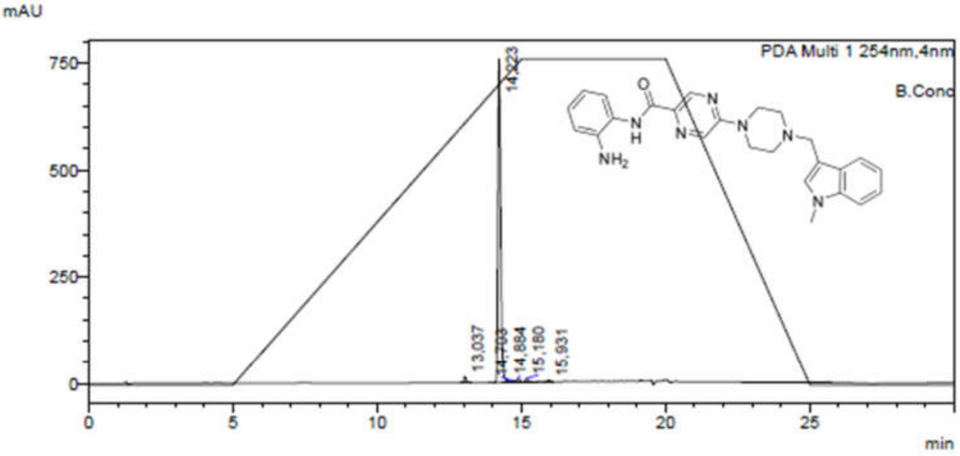

PDA Ch1 254nm

Peak#	Ret. Time	Area	Height	Area%
1	13,037	86724	14861	1,654
2	14,223	5004310	756877	95,438
3	14,703	14632	2859	0,279
4	14,884	59104	7638	1,127
5	15,180	54692	8790	1,043
6	15,931	24074	3784	0,459
Total		5243534	794810	100,000

HPLC Chromatogram of compound **19l**

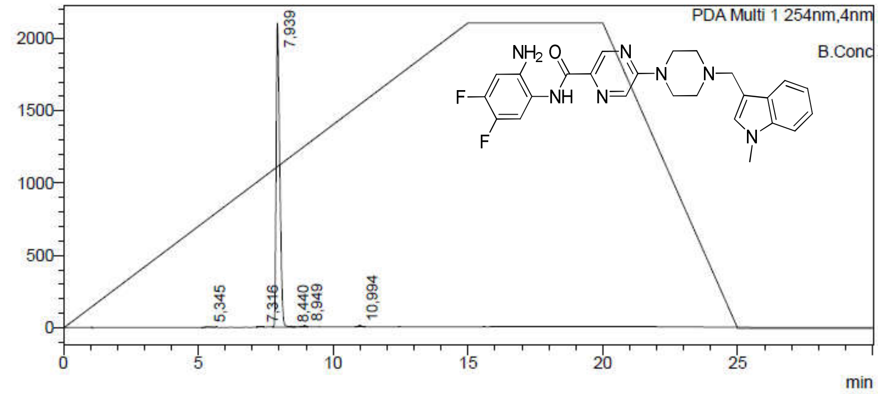

PDA Ch1 254nm

Peak#	Ret. Time	Area	Height	Area%
1	5,345	54077	3816	0,274
2	7,316	20295	3097	0,103
3	7,939	19486034	2105300	98,757
4	8,440	17360	3267	0,088
5	8,949	62751	10531	0,318
6	10,994	90739	12981	0,460
Total		19731255	2138992	100,000

HPLC chromatogram of compound **19m**

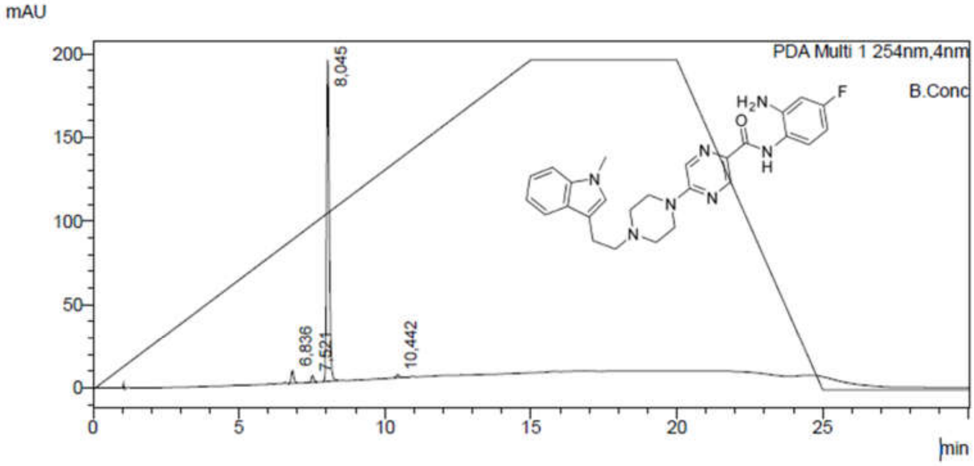

PDA Ch1 254nm

Peak#	Ret. Time	Area	Height	Area%
1	6,836	45658	7504	3,453
2	7,521	25036	4027	1,894
3	8,045	1239460	192607	93,744
4	10,442	12019	1872	0,909
Total		1322172	206011	100,000

HPLC chromatogram of compound **19n**

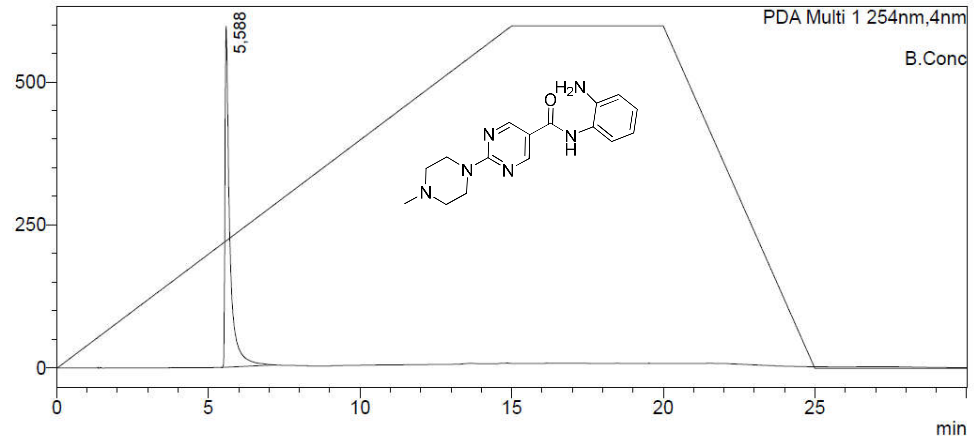

Ch1 254nm

k#	Ret. Time	Area	Height	Area%
1	5,588	6637169	596831	100,000
tal		6637169	596831	100,000

HPLC chromatogram of compound **19o**

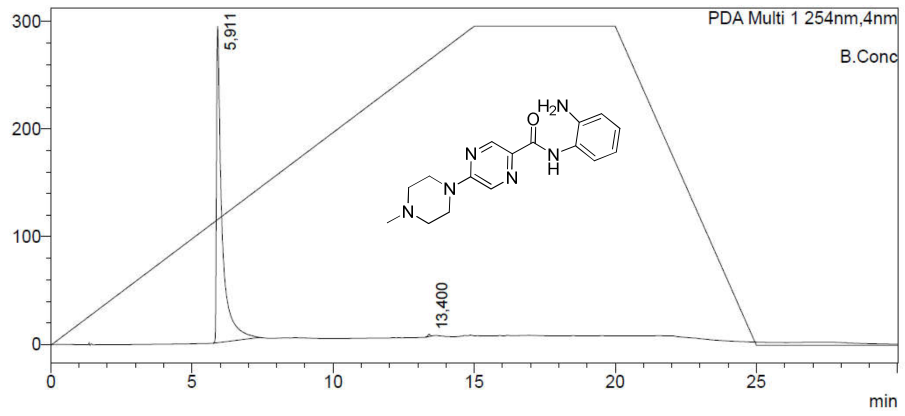

PDA Ch1 254nm

Peak#	Ret. Time	Area	Height	Area%
1	5,911	4013076	293914	99,727
2	13,400	10989	2191	0,273
Total		4024065	296105	100,000

HPLC Chromatogram of compound **21a**

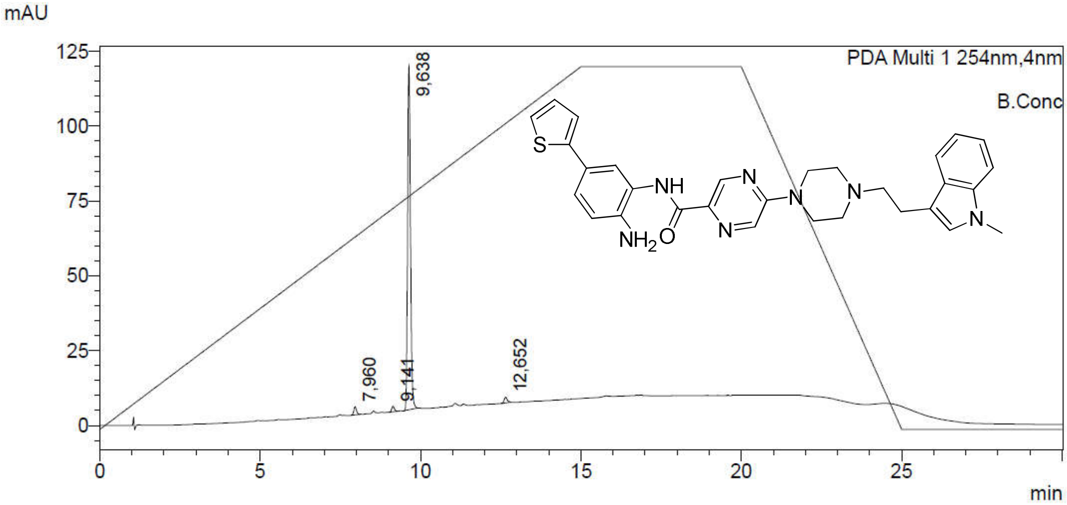

PDA Ch1 254nm

Peak#	Ret. Time	Area	Height	Area%
1	7,960	15944	2721	2,117
2	9,141	10787	1886	1,432
3	9,638	714116	114626	94,803
4	12,652	12420	1873	1,649
Total		753266	121106	100,000

HPLC chromatogram of compound **21b**

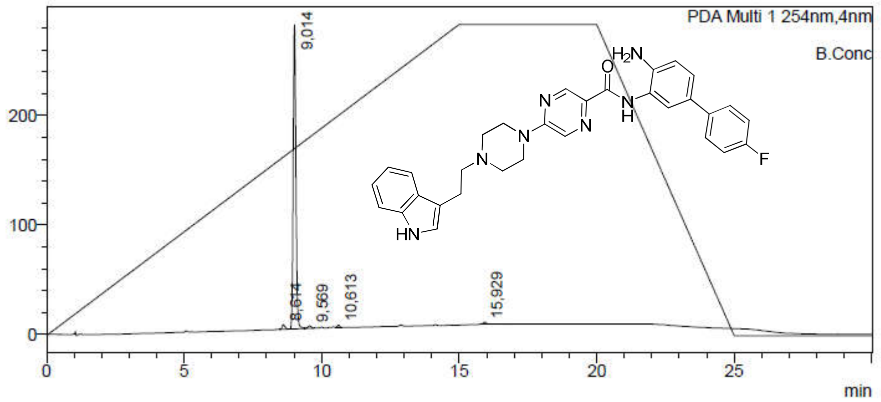

PDA Ch1 254nm

Peak#	Ret. Time	Area	Height	Area%
1	8,614	30915	4160	1,567
2	9,014	1899453	278205	96,253
3	9,569	20799	2190	1,054
4	10,613	12313	2149	0,624
5	15,929	9912	1448	0,502
Total		1973392	288152	100,000

HPLC chromatogram of compound **21c**

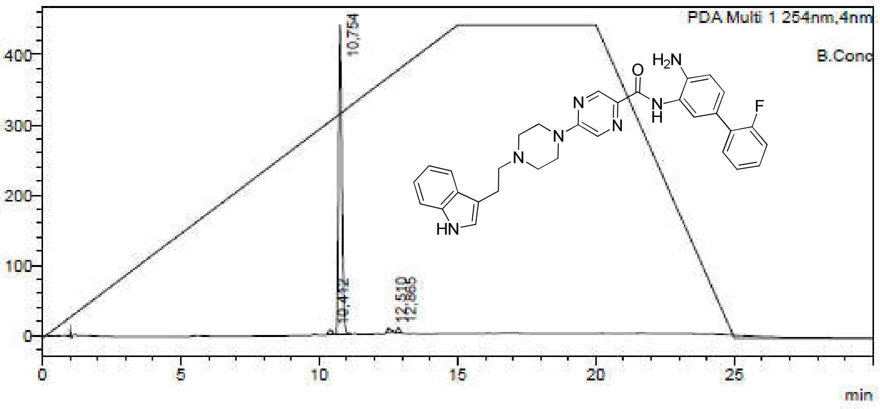

PDA Ch1 254nm

Peak#	Ret. Time	Area	Height	Area%
1	10,412	45816	5558	1,190
2	10,754	3708618	440502	96,357
3	12,510	45173	5696	1,174
4	12,865	49234	7085	1,279
Total		3848840	458842	100,000

HPLC chromatogram of compound **23a**

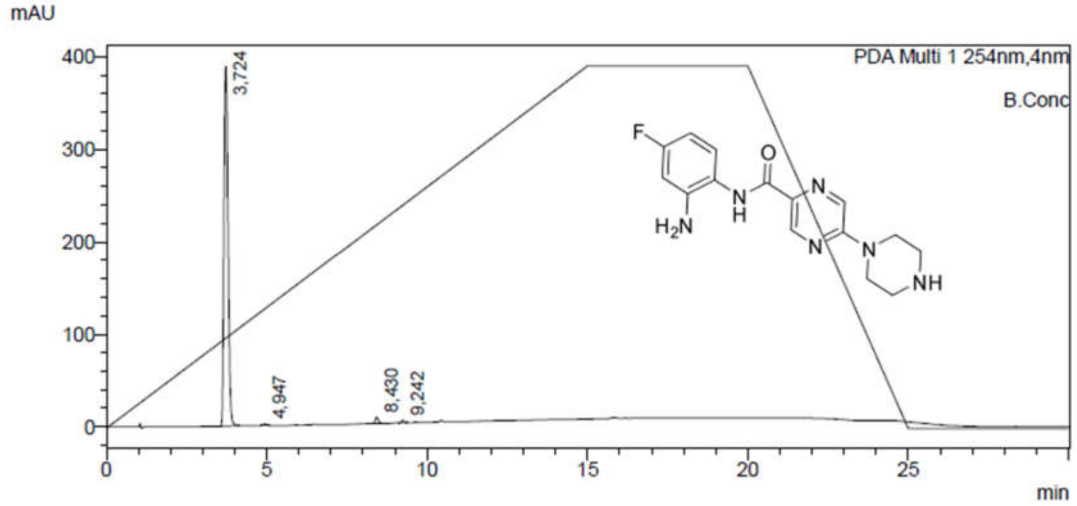

PDA Ch1 254nm

Peak#	Ret. Time	Area	Height	Area%
1	3,724	3418434	389040	97,915
2	4,947	11714	1753	0,336
3	8,430	45436	6808	1,301
4	9,242	15631	2520	0,448
Total		3491215	400121	100,000

HPLC chromatogram of compound **23b**

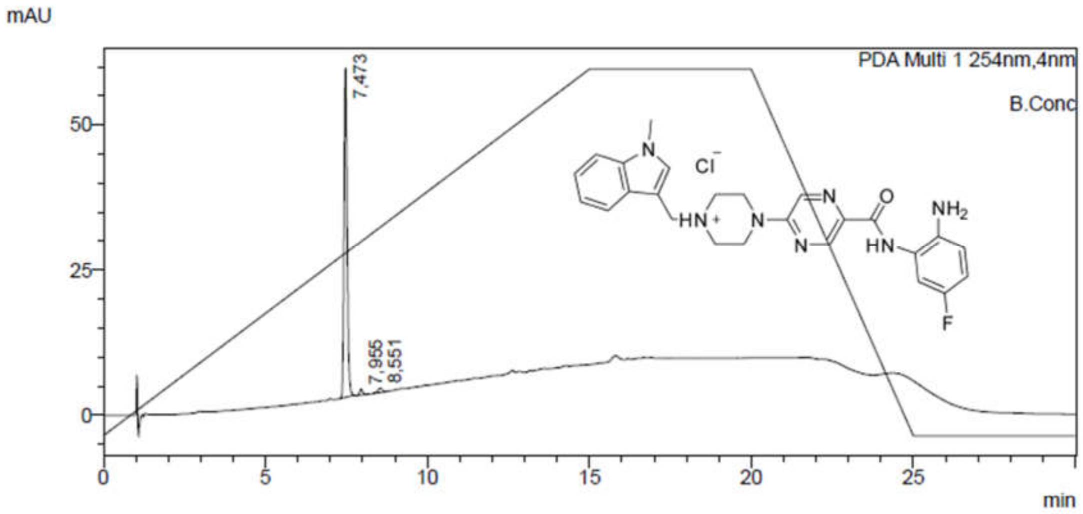

PDA Ch1 254nm

Peak#	Ret. Time	Area	Height	Area%
1	7,473	371403	56483	96,678
2	7,955	6247	1014	1,626
3	8,551	6515	736	1,696
Total		384165	58233	100,000

HPLC chromatogram of compound **23c**

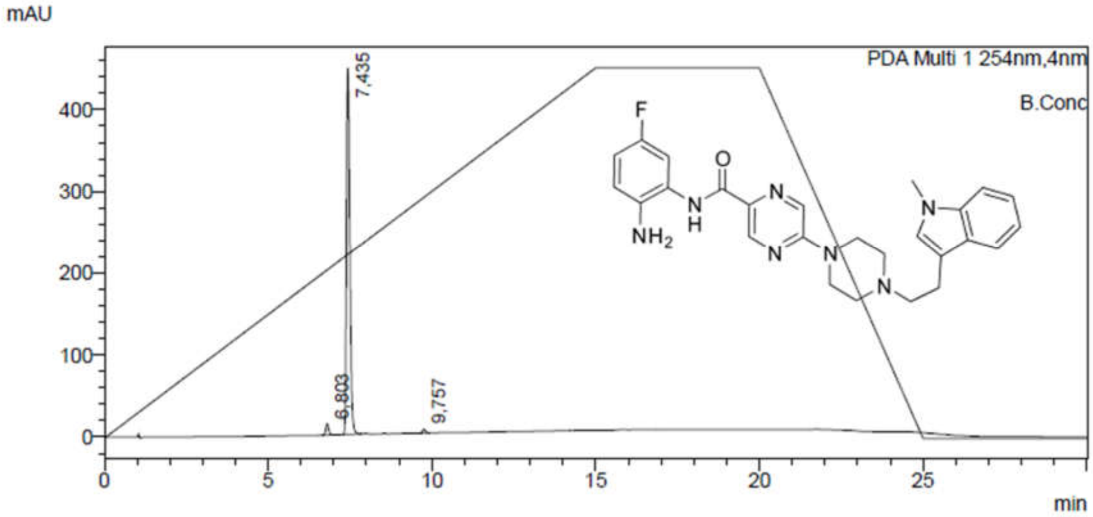

PDA Ch1 254nm

Peak#	Ret. Time	Area	Height	Area%
1	6,803	84466	14011	2,542
2	7,435	3208583	447729	96,573
3	9,757	29403	4717	0,885
Total		3322452	466457	100,000

HPLC Chromatogram of compound **25a**

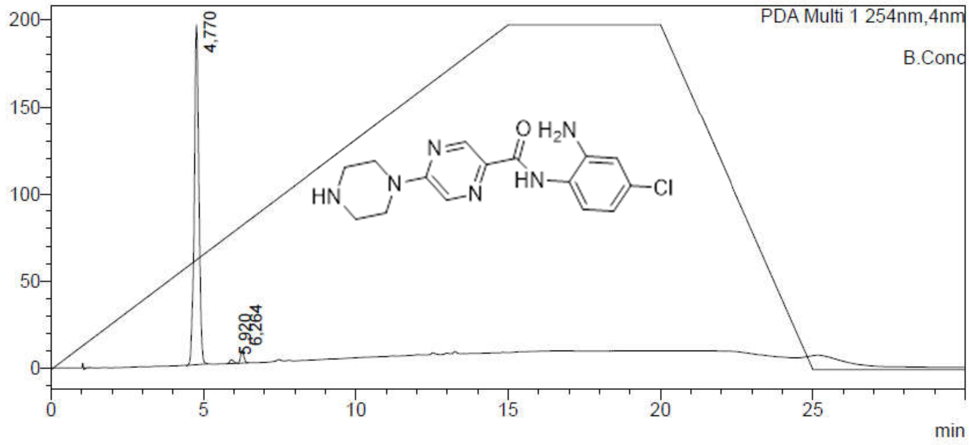

PDA Ch1 254nm

Peak#	Ret. Time	Area	Height	Area%
1	4,770	2050691	195407	96,548
2	5,920	19458	2166	0,916
3	6,264	53874	7107	2,536
Total		2124022	204680	100,000

HPLC Chromatogram of compound **25b**

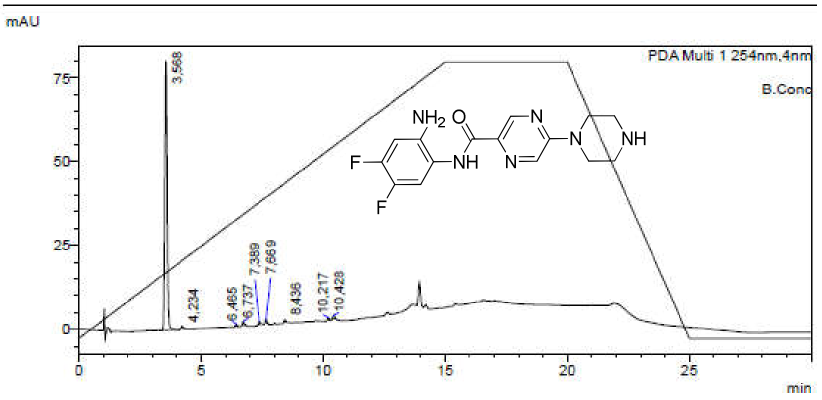

PDA Ch1 254nm

Peak#	Ret. Time	Area	Height	Area%
1	3,568	526433	79933	95,570
2	4,234	1347	429	0,245
3	6,465	2320	662	0,421
4	6,737	5038	1163	0,915
5	7,389	3607	862	0,655
6	7,669	2399	801	0,436
7	8,436	4048	866	0,735
8	10,217	1249	431	0,227
9	10,428	4394	901	0,798
Total		550836	86048	100,000

HPLC Chromatogram of compound **27a**

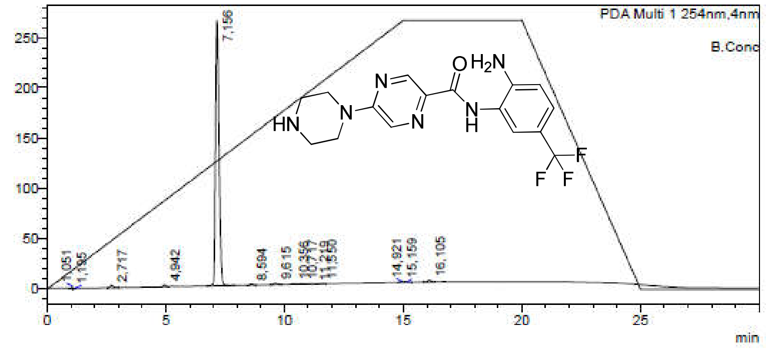

PDA Ch1 254nm

Peak#	Ret. Time	Area	Height	Area%
1	1,051	8158	2991	0,296
2	1,195	3172	461	0,115
3	2,717	26868	2363	0,975
4	4,942	15246	1867	0,553
5	7,156	2637602	264401	95,700
6	8,594	8329	923	0,302
7	9,615	16795	1498	0,609
8	10,356	4742	608	0,172
9	10,717	4486	518	0,163
10	11,219	1004	90	0,036
11	11,550	2111	225	0,077
12	14,921	8519	1103	0,309
13	15,159	2401	322	0,087
14	16,105	16684	1865	0,605
Total		2756118	279235	100,000

HPLC Chromatogram of compound **27b**

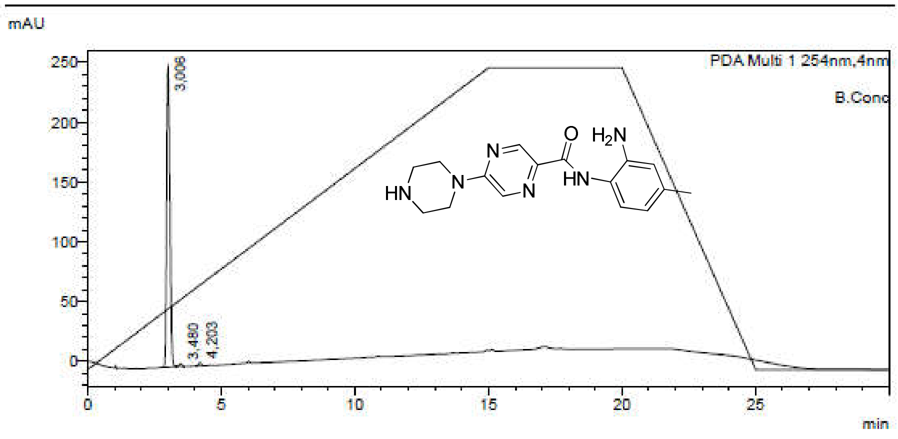

PDA Ch1 254nm

Peak#	Ret. Time	Area	Height	Area%
1	3,006	2237498	250098	98,212
2	3,480	21037	2505	0,923
3	4,203	19693	2753	0,864
Total		2278229	255356	100,000

HPLC chromatogram of compound **27c**

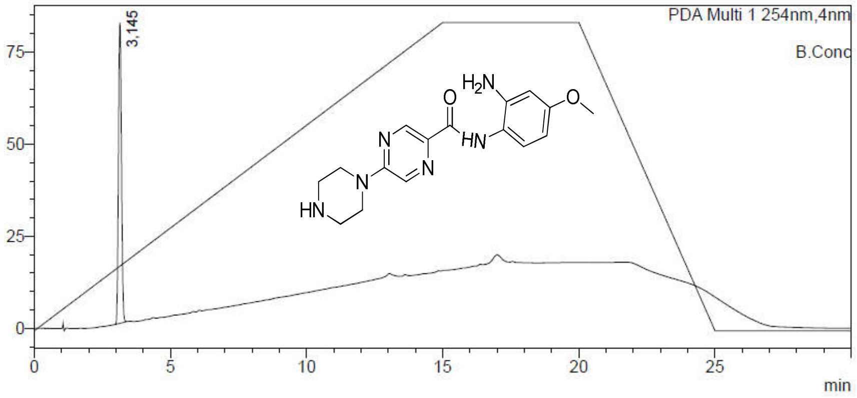

PDA Ch1 254nm

Peak#	Ret. Time	Area	Height	Area%
1	3,145	628233	81604	100,000
Total		628233	81604	100,000

HPLC Chromatogram of compound **29a**

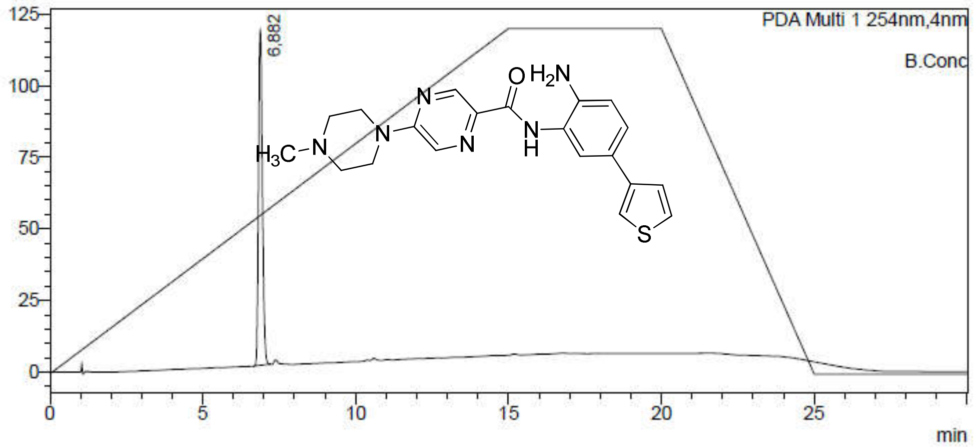

PDA Ch1 254nm

Peak#	Ret. Time	Area	Height	Area%
1	6,882	1036291	117555	100,000
Total		1036291	117555	100,000

HPLC Chromatogram of compound **29b**

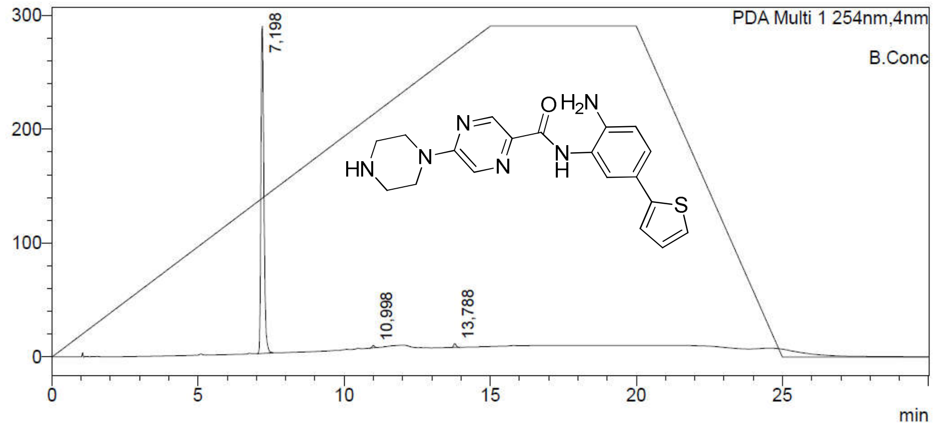

PDA Ch1 254nm

Peak#	Ret. Time	Area	Height	Area%
1	7,198	2006192	287302	98,468
2	10,998	12141	1992	0,596
3	13,788	19073	3388	0,936
Total		2037406	292681	100,000

HPLC chromatogram of compound **29c**

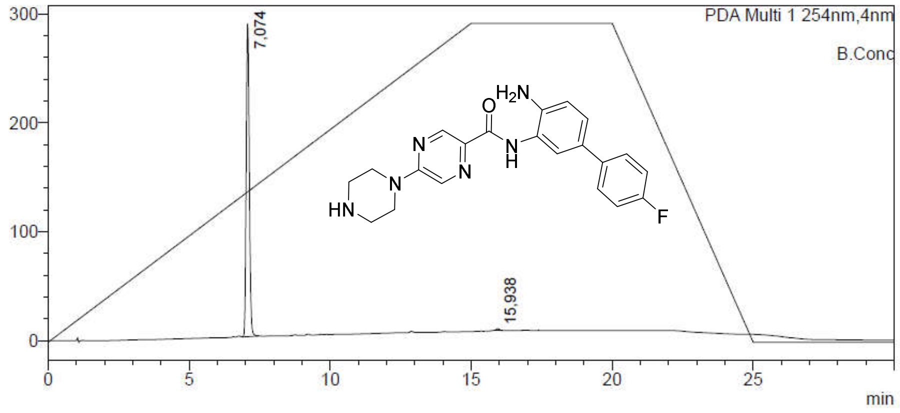

PDA Ch1 254nm

Peak#	Ret. Time	Area	Height	Area%
1	7,074	2200345	287622	99,547
2	15,938	10007	1459	0,453
Total		2210352	289081	100,000

HPLC chromatogram of compound **29d**

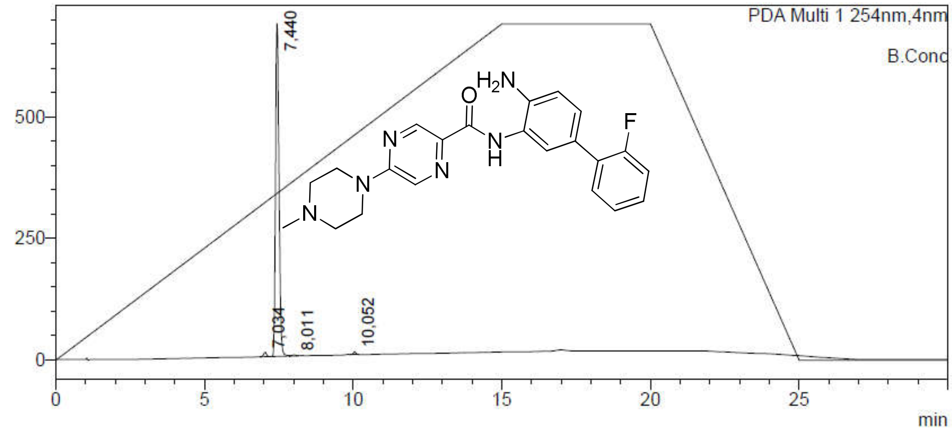

PDA Ch1 254nm

Peak#	Ret. Time	Area	Height	Area%
1	7,034	63299	8959	1,111
2	7,440	5576218	685071	97,864
3	8,011	20223	2347	0,355
4	10,052	38173	5937	0,670
Total		5697913	702314	100,000

7. IC_{50} plots of compounds 19f and 21a against different HDACs isoforms

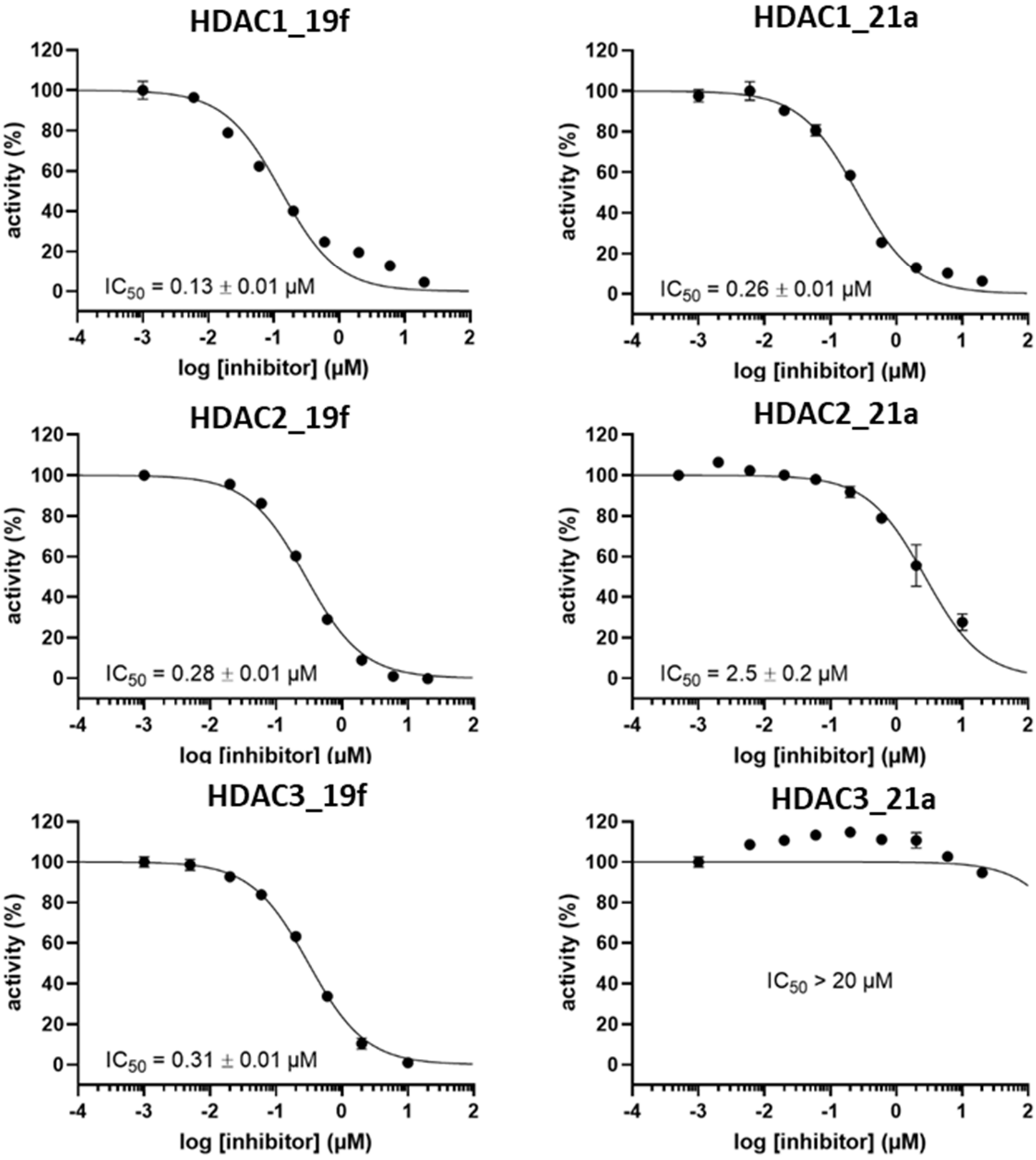

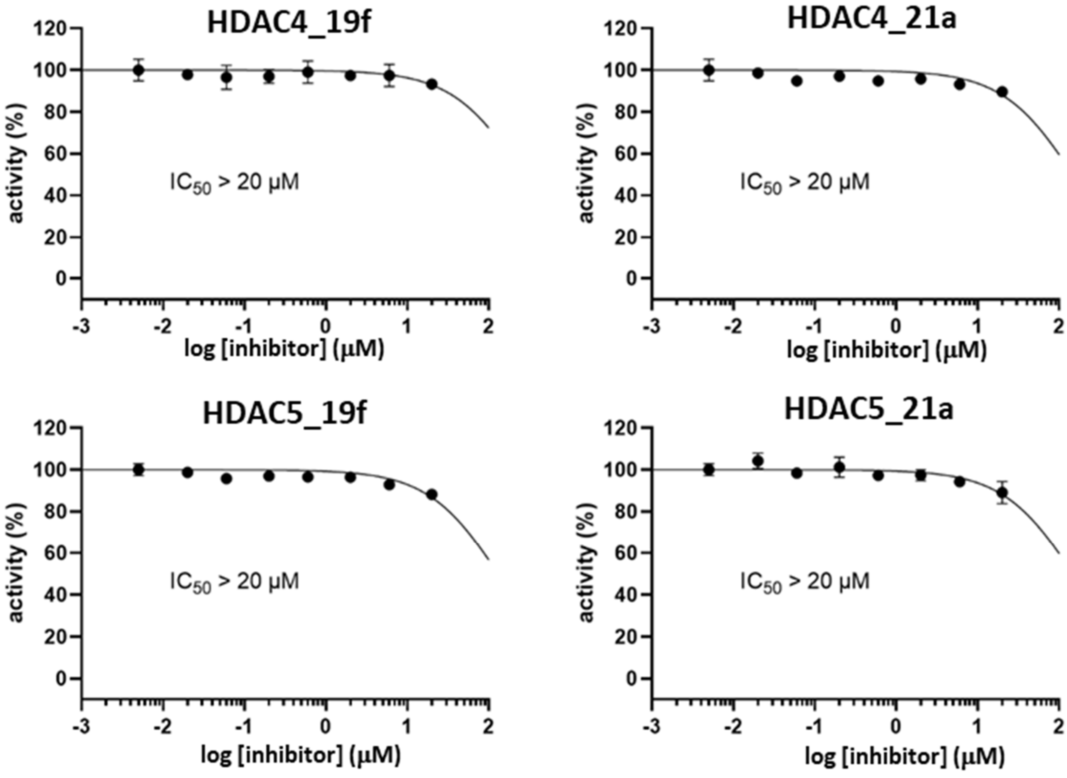
HDAC4_19f
HDAC4_21a
HDAC5_19f
HDAC5_21a
activity (%)
log [inhibitor] (µM)
IC50 > 20 µM
120
100
80
60
40
20
0
-3
-2
-1
0
1
2

HDAC6_19f

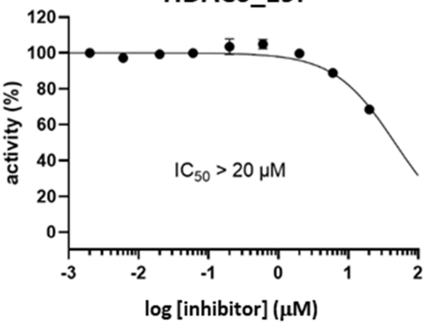

HDAC6_21a

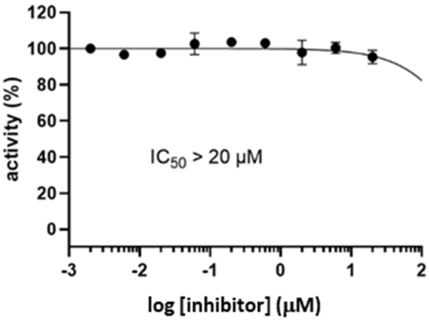

HDAC7_19f

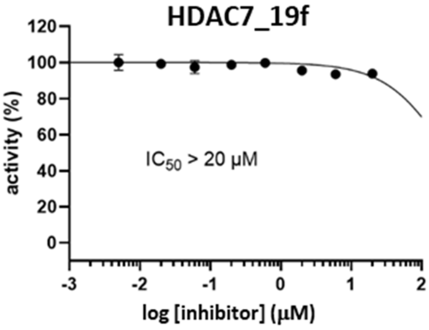

HDAC7_21a

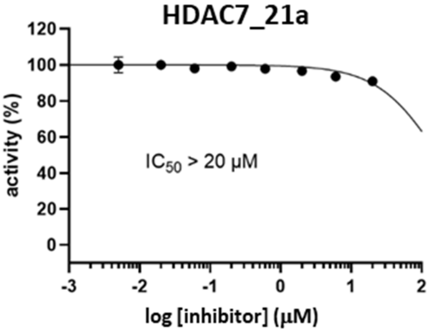

HDAC8_19f

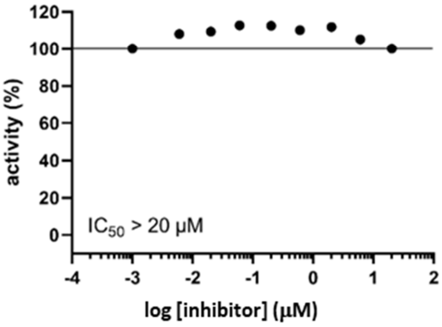

HDAC8_21a

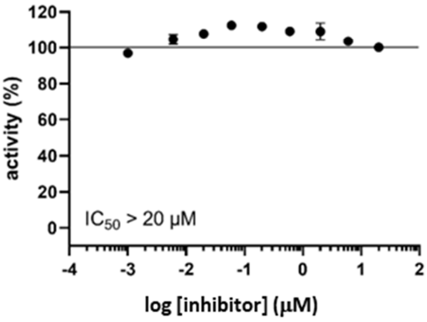

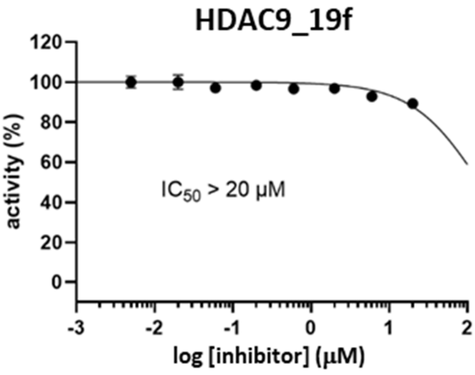
HDAC9_19f
activity (%)
log [inhibitor] (µM)
IC50 > 20 µM

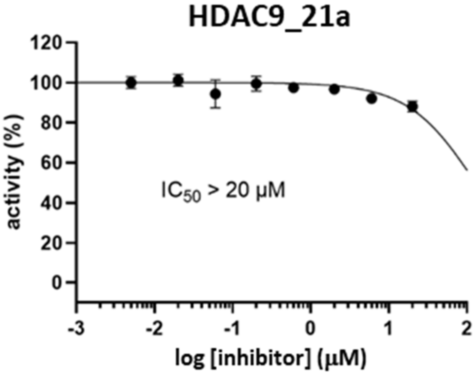
HDAC9_21a
activity (%)
log [inhibitor] (µM)
IC50 > 20 µM

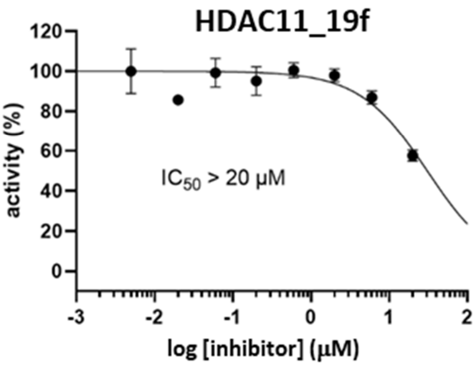
HDAC11_19f
activity (%)
log [inhibitor] (µM)
IC50 > 20 µM

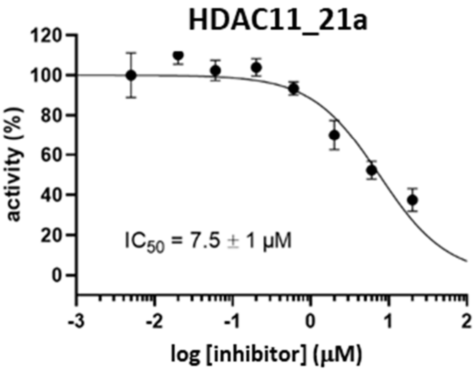
HDAC11_21a
activity (%)
log [inhibitor] (µM)
IC50 = 7.5 ± 1 µM

8. IC_{50} plots of reference inhibitors against HDAC1, -2, and -3

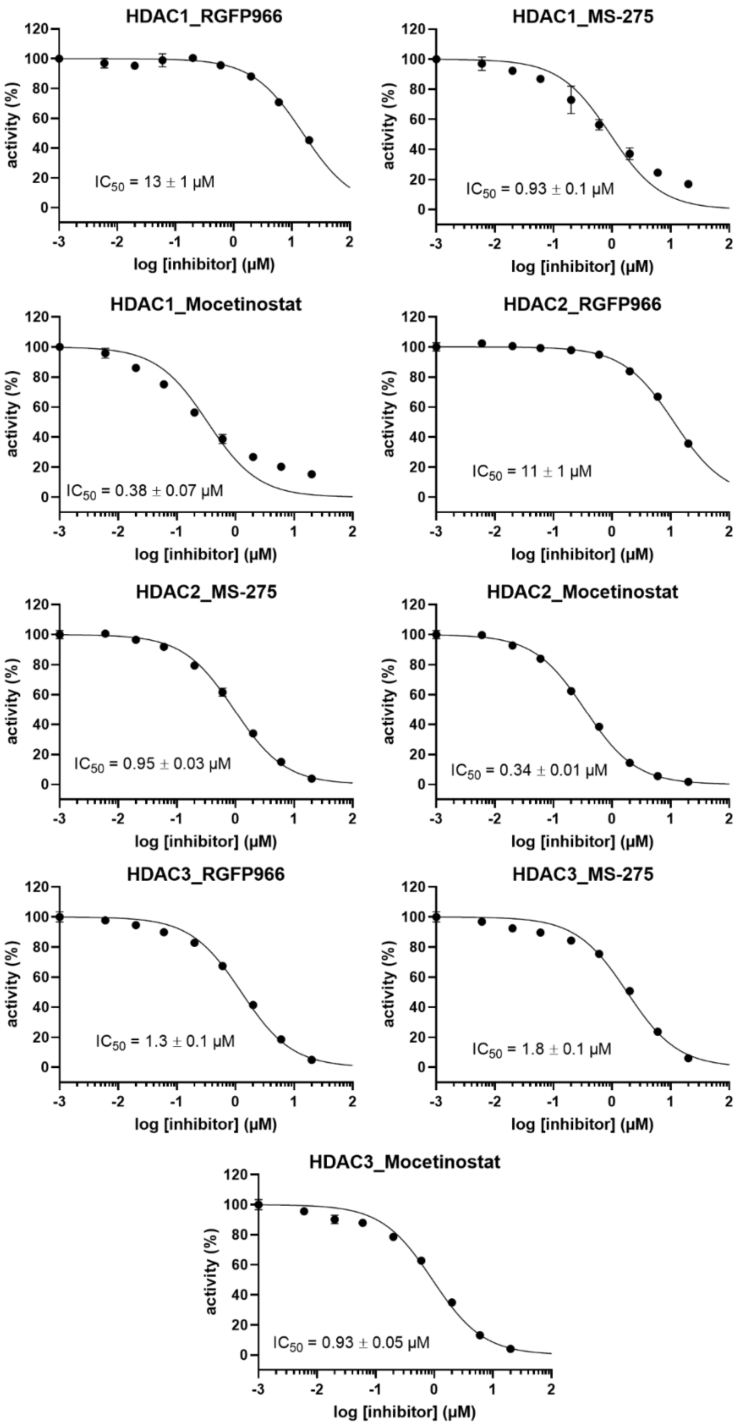

#

9. IC50 plots of tested inhibitors against HDAC1, -2, and -3

HDAC1

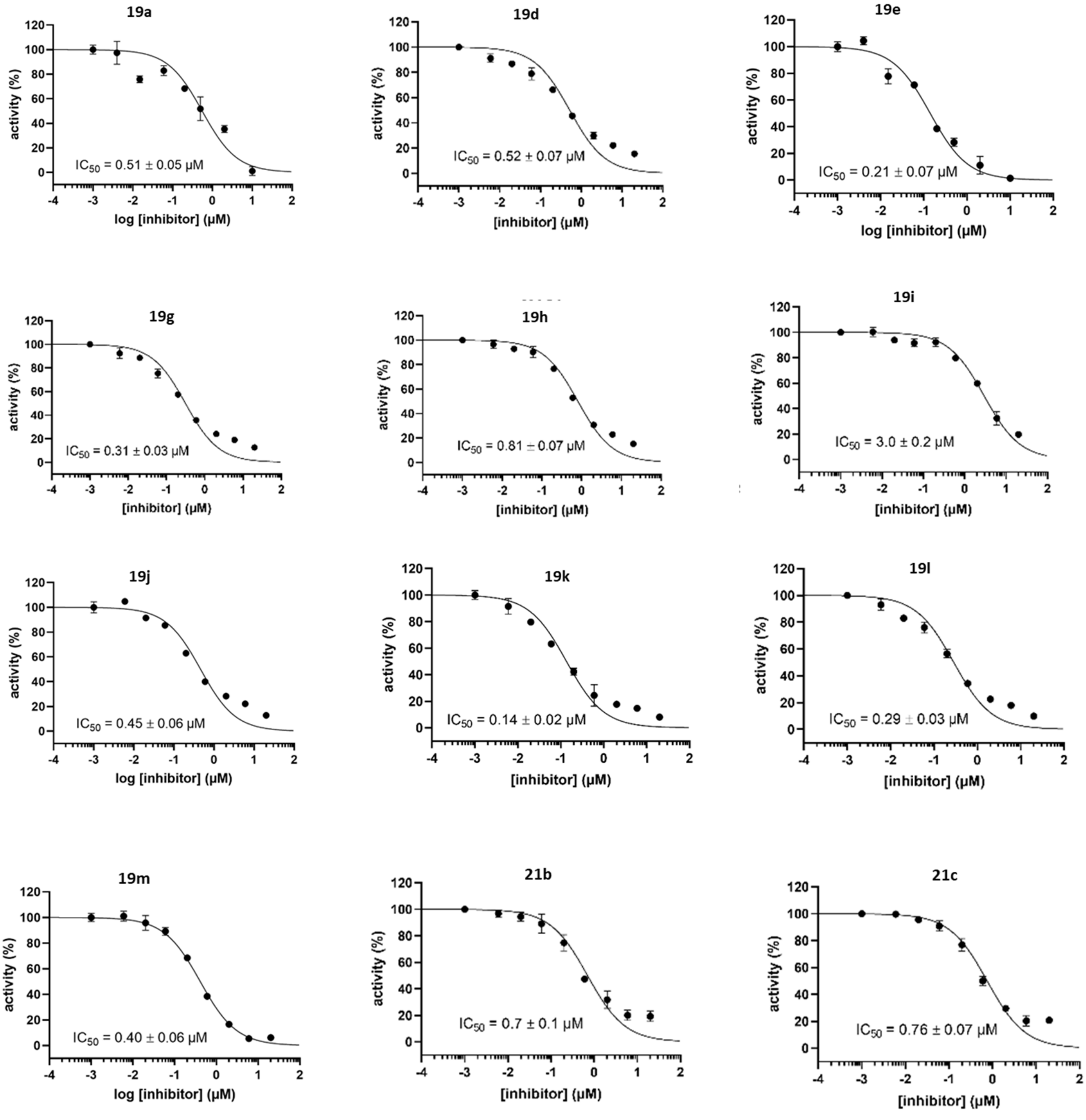

23a

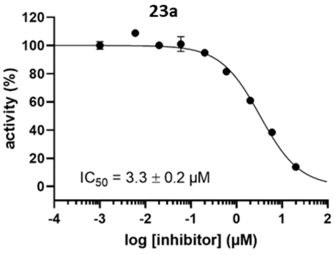

23b

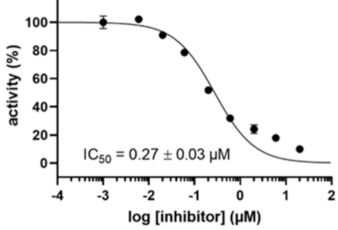

23c

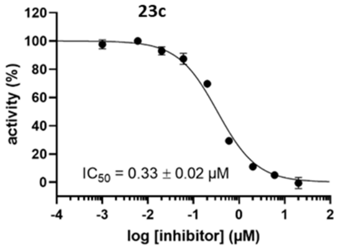

25b

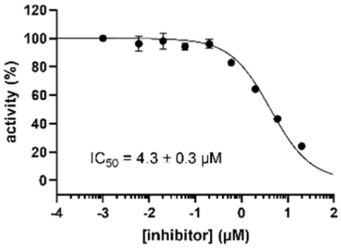

27c

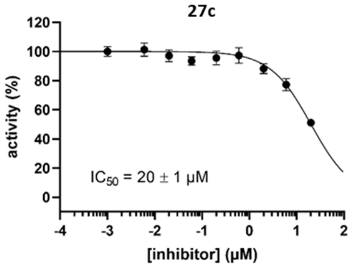

29a

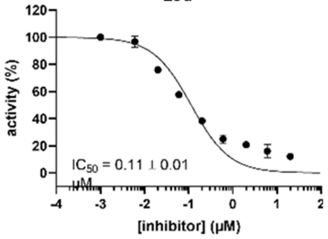

29b

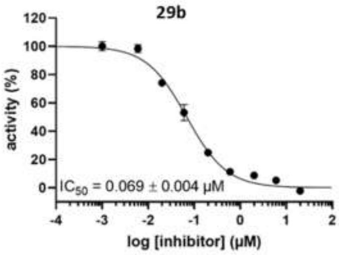

29c

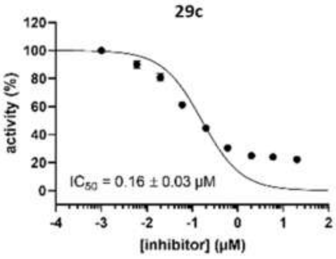

29d

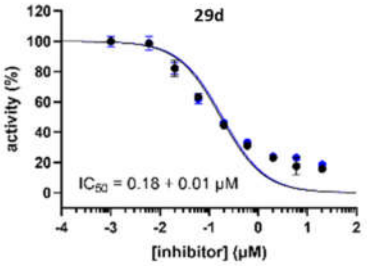

HDAC2

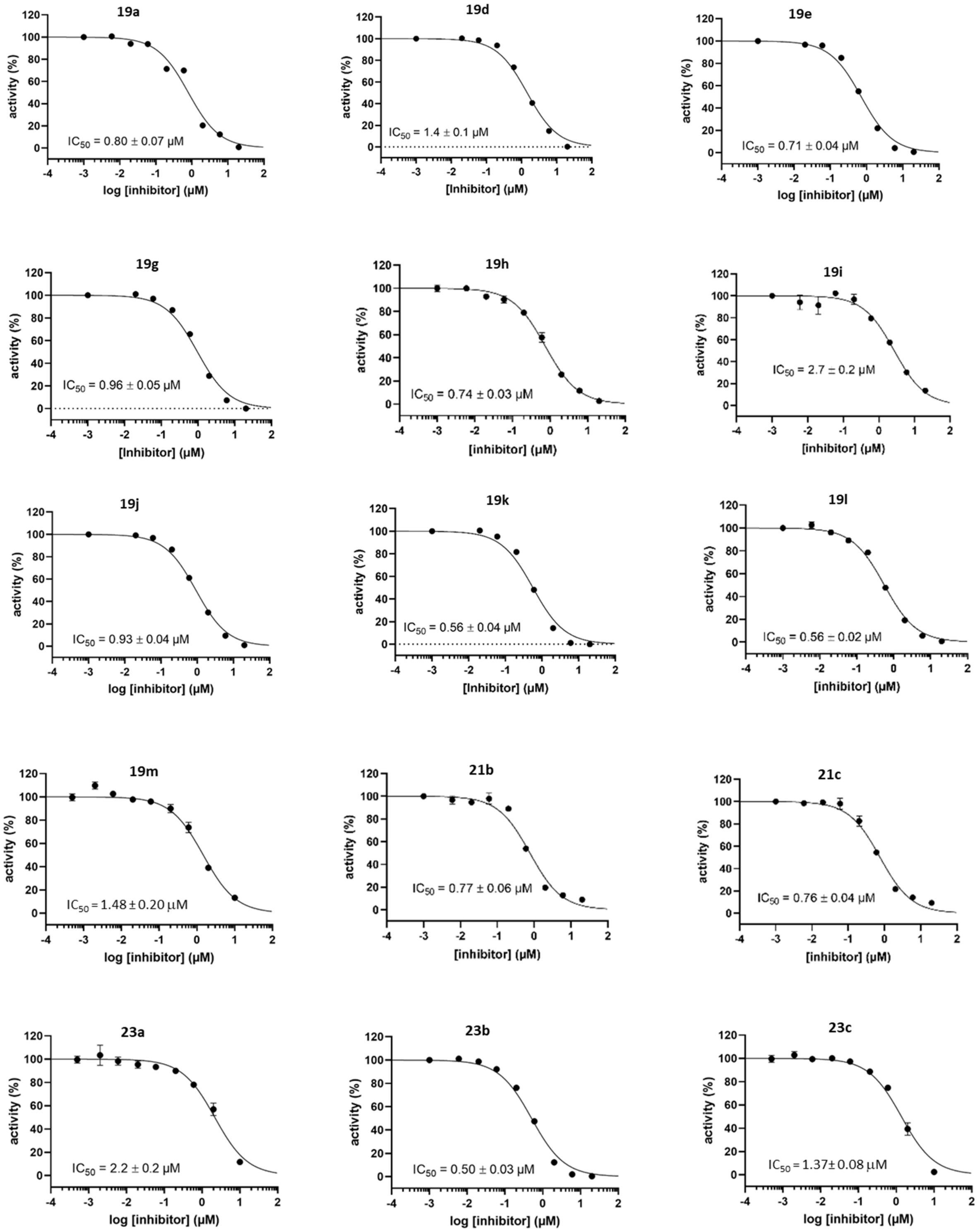
19a
activity (%)
IC_{50} = 0.80 ± 0.07 µM
log [inhibitor] (µM)
19d
activity (%)
IC_{50} = 1.4 ± 0.1 µM
[Inhibitor] (µM)
19e
activity (%)
IC_{50} = 0.71 ± 0.04 µM
log [inhibitor] (µM)
19g
activity (%)
IC_{50} = 0.96 ± 0.05 µM
[Inhibitor] (µM)
19h
activity (%)
IC_{50} = 0.74 ± 0.03 µM
[inhibitor] (µM)
19i
activity (%)
IC_{50} = 2.7 ± 0.2 µM
[inhibitor] (µM)
19j
activity (%)
IC_{50} = 0.93 ± 0.04 µM
log [inhibitor] (µM)
19k
activity (%)
IC_{50} = 0.56 ± 0.04 µM
[Inhibitor] (µM)
19l
activity (%)
IC_{50} = 0.56 ± 0.02 µM
[inhibitor] (µM)
19m
activity (%)
IC_{50} = 1.48 ± 0.20 µM
log [inhibitor] (µM)
21b
activity (%)
IC_{50} = 0.77 ± 0.06 µM
[inhibitor] (µM)
21c
activity (%)
IC_{50} = 0.76 ± 0.04 µM
[inhibitor] (µM)
23a
activity (%)
IC_{50} = 2.2 ± 0.2 µM
log [inhibitor] (µM)
23b
activity (%)
IC_{50} = 0.50 ± 0.03 µM
log [inhibitor] (µM)
23c
activity (%)
IC_{50} = 1.37± 0.08 µM
log [inhibitor] (µM)

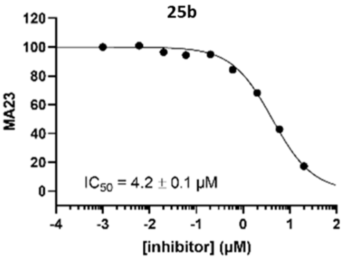
25b
MA23
[inhibitor] (μM)
IC_{50} = 4.2 ± 0.1 μM

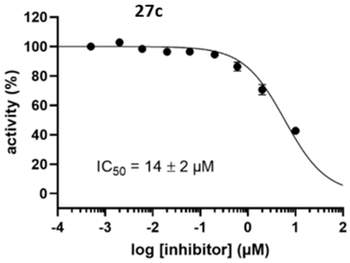
27c
activity (%)
log [inhibitor] (μM)
IC_{50} = 14 ± 2 μM

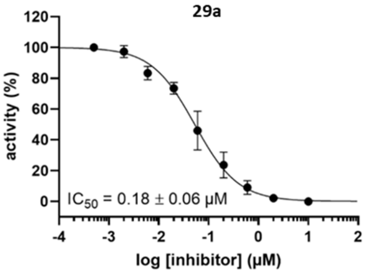
29a
activity (%)
log [inhibitor] (μM)
IC_{50} = 0.18 ± 0.06 μM

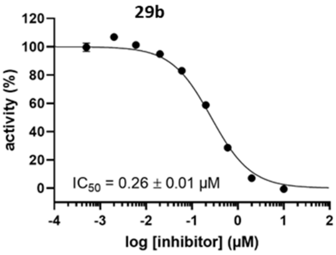
29b
activity (%)
log [inhibitor] (μM)
IC_{50} = 0.26 ± 0.01 μM

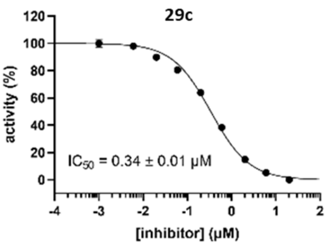
29c
activity (%)
[inhibitor] (μM)
IC_{50} = 0.34 ± 0.01 μM

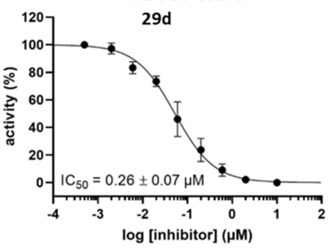
29d
activity (%)
log [inhibitor] (μM)
IC_{50} = 0.26 ± 0.07 μM

HDAC3

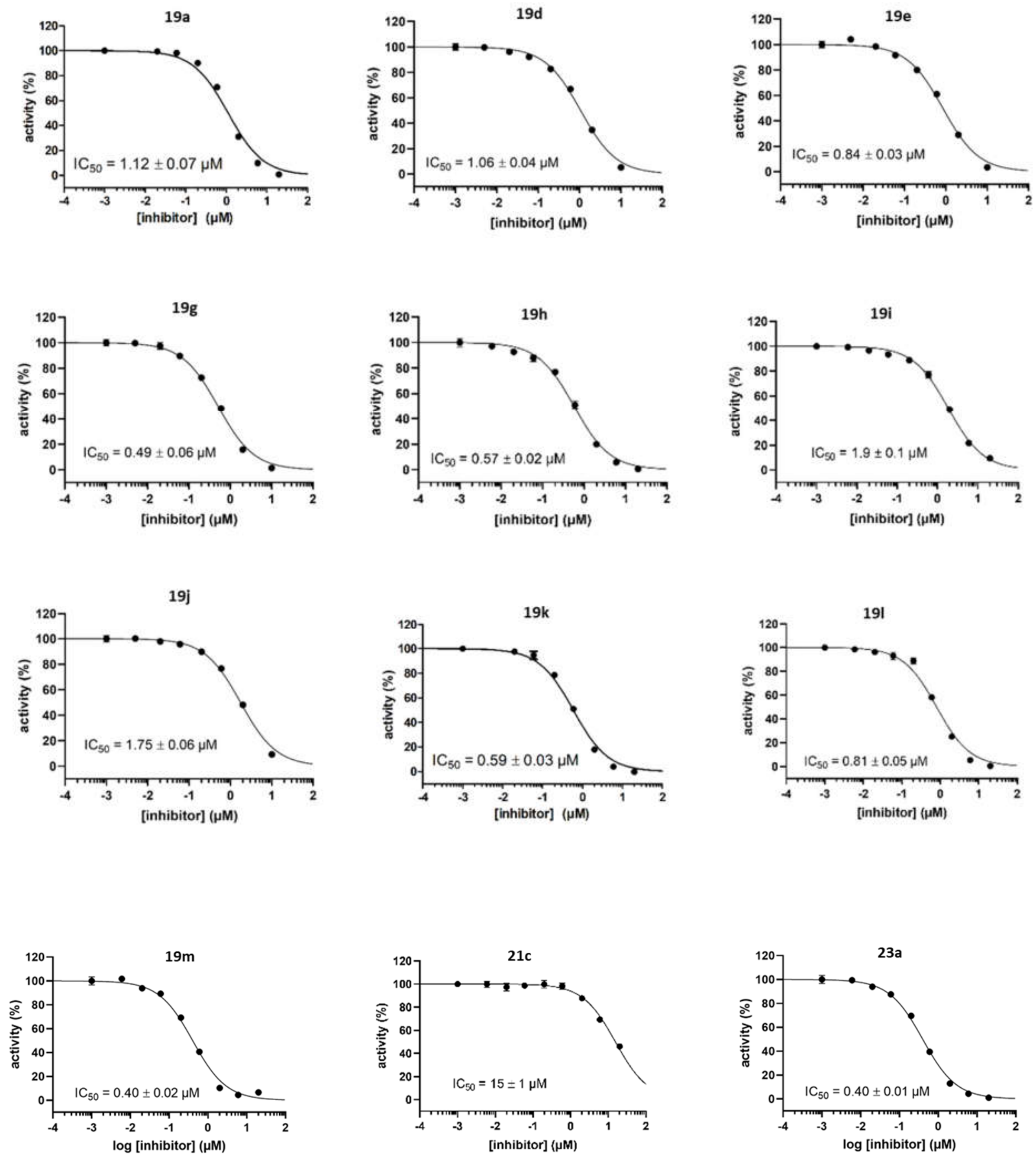
19a
IC50 = 1.12 ± 0.07 µM
19d
IC50 = 1.06 ± 0.04 µM
19e
IC50 = 0.84 ± 0.03 µM
19g
IC50 = 0.49 ± 0.06 µM
19h
IC50 = 0.57 ± 0.02 µM
19i
IC50 = 1.9 ± 0.1 µM
19j
IC50 = 1.75 ± 0.06 µM
19k
IC50 = 0.59 ± 0.03 µM
19l
IC50 = 0.81 ± 0.05 µM
19m
IC50 = 0.40 ± 0.02 µM
21c
IC50 = 15 ± 1 µM
23a
IC50 = 0.40 ± 0.01 µM
activity (%)
[inhibitor] (µM)
log [inhibitor] (µM)

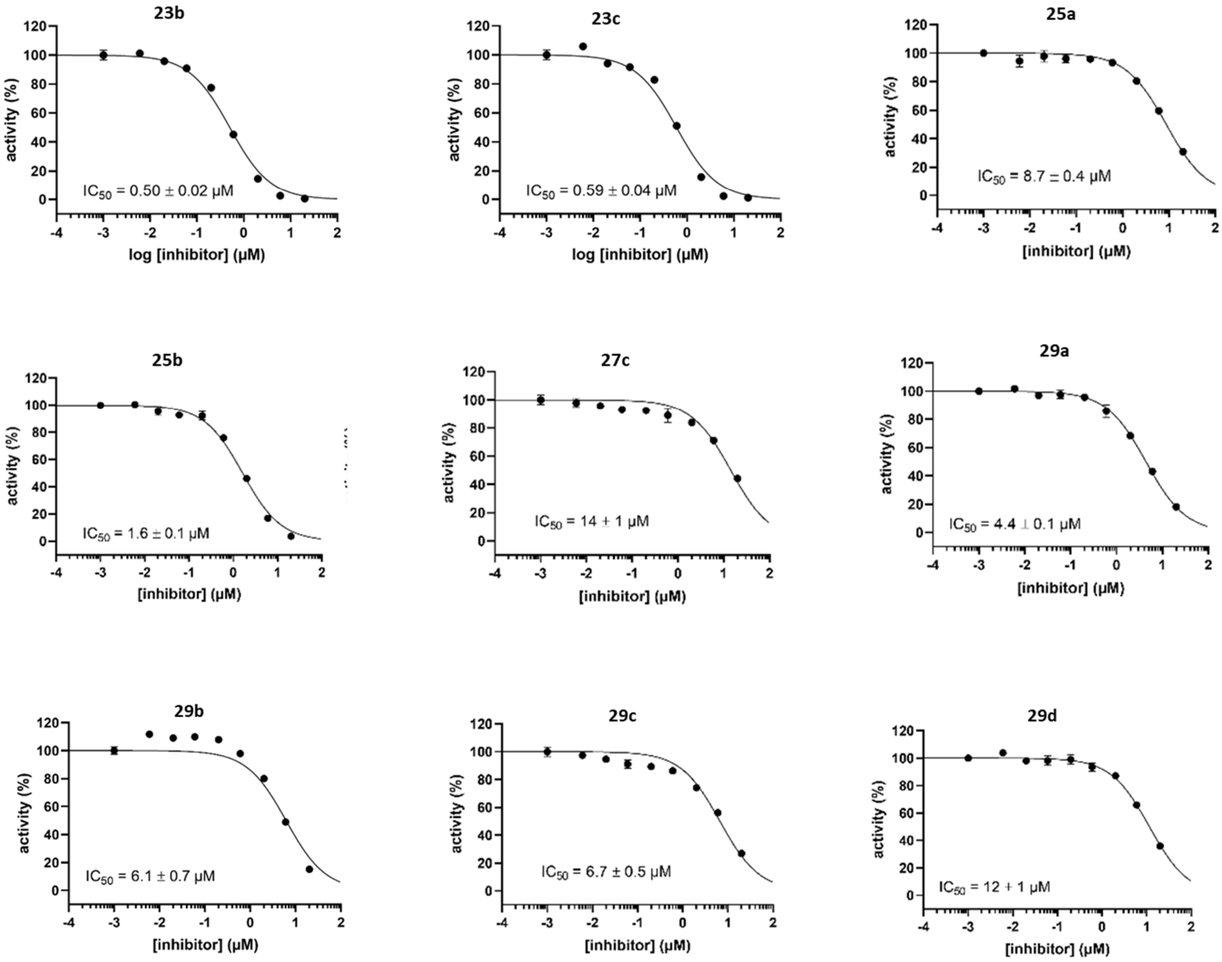
23b
IC50 = 0.50 ± 0.02 μM
23c
IC50 = 0.59 ± 0.04 μM
25a
IC50 = 8.7 ± 0.4 μM
25b
IC50 = 1.6 ± 0.1 μM
27c
IC50 = 14 ± 1 μM
29a
IC50 = 4.4 ± 0.1 μM
29b
IC50 = 6.1 ± 0.7 μM
29c
IC50 = 6.7 ± 0.5 μM
29d
IC50 = 12 ± 1 μM
activity (%)
log [inhibitor] (μM)
[inhibitor] (μM)

10. Molecular docking studies

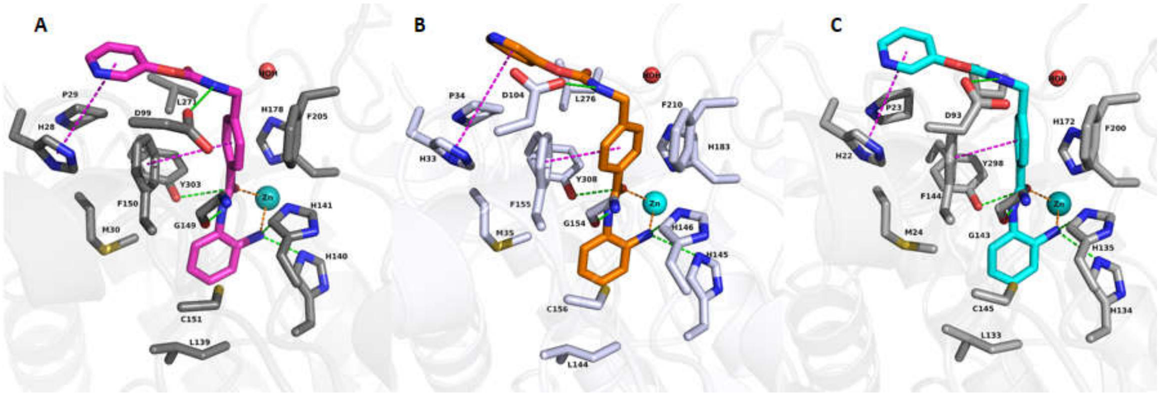

Fig. S1. Docking poses of Entinostat (A, magenta colored sticks) in HDAC1 (PDB ID 4BKX), (B, orange colored sticks) in HDAC2 (PDB ID 4LY1), (C, cyan colored sticks) in HDAC3 (PDB ID 4A69). Hydrogen bonds (green dashed lines), metal coordination (orange dashed lines), ionic interactions (cyan dashed lines) and aromatic interactions (magenta dashed lines) between inhibitors and the protein are shown. Relevant residues are shown in stick representation with dark grey carbon atoms in HDAC1, white carbon atoms in HDAC2, and light grey carbon atoms in HDAC3. The zinc ion is shown as cyan colored sphere. The conserved water molecule is shown as red sphere. The zinc-carbonyl oxygen and zinc-amino distance, respectively, are: 2.37 Å and 2.61 Å for Figure A, 2.45 Å and 2.31 Å for Figure B, 2.44 Å and 2.47 Å for Figure C.

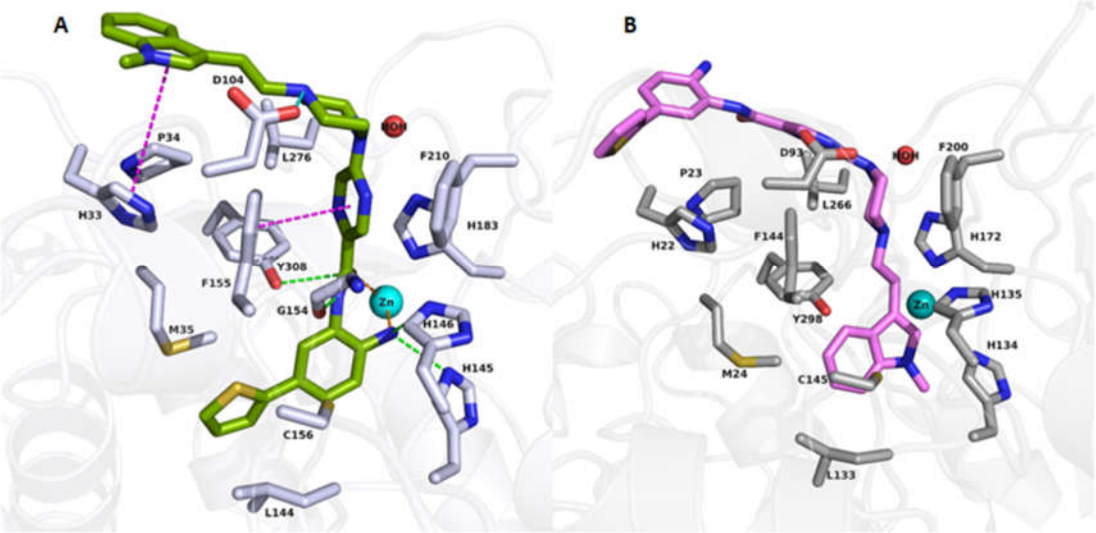

Fig. S2. Docking pose of **21a** (A, green colored sticks) in HDAC2 (PDB ID 4LY1), **21a** (B, pink colored sticks) in HDAC3 (PDB ID 4A69). Hydrogen bonds (green dashed lines), metal coordination (orange dashed lines), ionic interactions (cyan dashed lines) and aromatic interactions (magenta dashed lines) between inhibitors and the protein are shown. Relevant residues are shown in stick representation with white carbon atoms white carbon atoms in HDAC2 and gray carbon in HDAC3. The zinc ion is shown as cyan colored sphere. The conserved water molecule is shown as red sphere. The zinc-carbonyl oxygen and zinc-amino distance, respectively, are: 2.22 Å and 2.29 Å for Figure A.

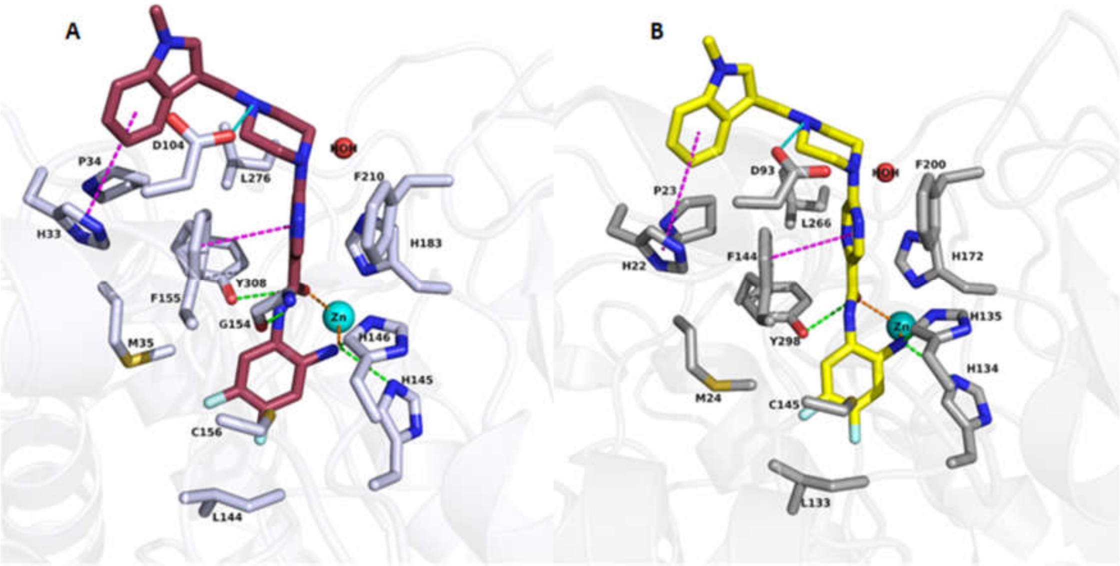

Fig. S3. Docking pose of **19l** (A, brown colored sticks) in HDAC2 (PDB ID 4LY1), **19l** (B, yellow colored sticks), in HDAC3 (PDB ID 4A69). Hydrogen bonds (green dashed lines), metal coordination (orange dashed lines), ionic interactions (cyan dashed lines) and aromatic interactions (magenta dashed lines) between inhibitors and the protein are shown. Relevant residues are shown in stick representation with white carbon atoms in HDAC2 and gray carbon in HDAC3. The zinc ion is shown as cyan colored sphere. The conserved water molecule is shown as red sphere. The zinc-carbonyl oxygen and zinc-amino distance, respectively, are: 2.23 Å and 2.37 Å for Figure A and 2.35 Å and 2.44 Å for Figure B.

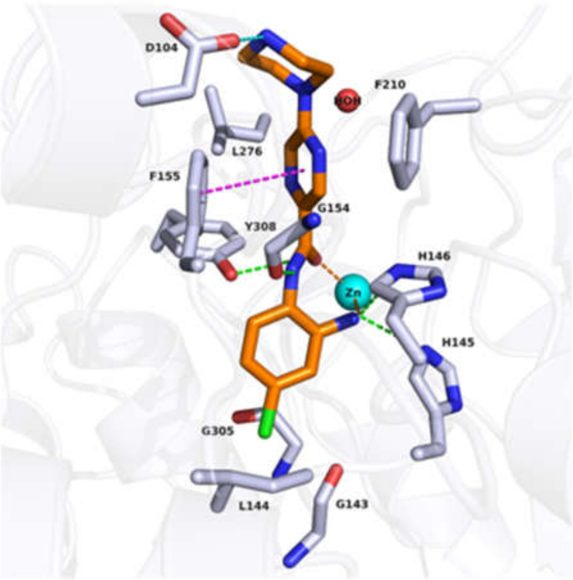

Fig. S4. Docking pose of **25a** (orange colored sticks) in HDAC2 (PDB ID 4LY1). Hydrogen bonds (green dashed lines), metal coordination (orange dashed lines), ionic interactions (cyan dashed lines) and aromatic interactions (magenta dashed lines) between inhibitors and the protein are shown. Relevant residues are shown in stick representation with white carbon atoms. The zinc ion is shown as cyan colored sphere. The conserved water molecule is shown as red sphere. The zinc-carbonyl oxygen and zinc-amino distance, respectively, are: 2.08 Å and 2.21 Å.

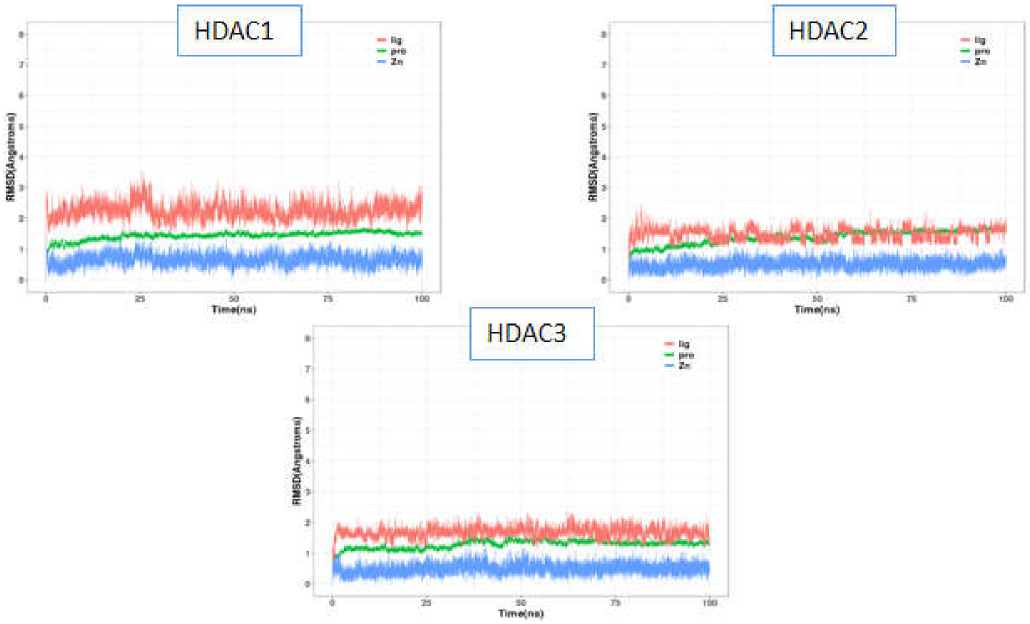

Fig. S5. Root-mean-square-deviation (RMSD) analysis of 100ns MD simulation of **23a** in complex with HDAC1/2 and 3 (PDB IDs: 4BKX, 4LY1, 4A69, respectively). Deviation of ligand is represented in red color, of protein in green color and of the zinc ion in blue color.

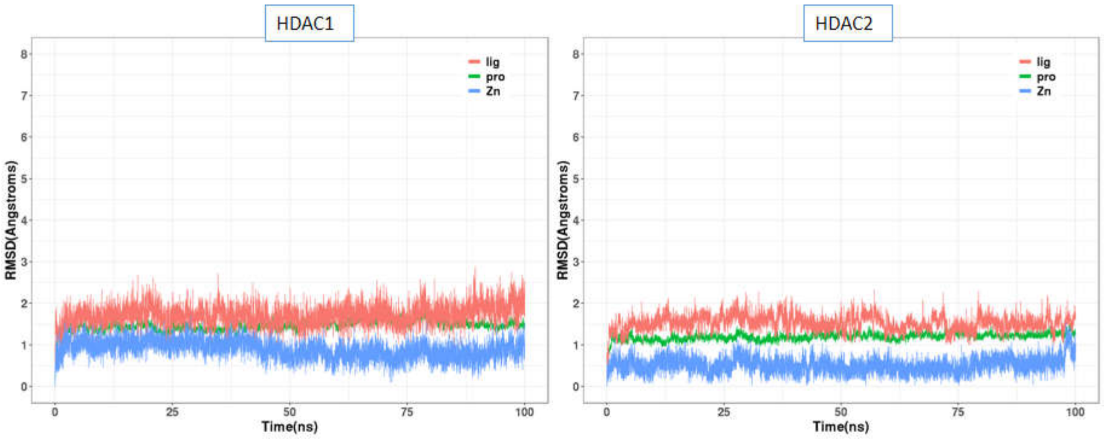

Fig. S6. Root-mean-square-deviation (RMSD) analysis of 100ns MD simulation of **29b** in complex with HDAC1/2 (PDB IDs: 4BKX, 4LY1 respectively). Deviation of ligand is represented in red color, of protein in green color and of the zinc ion in blue color.

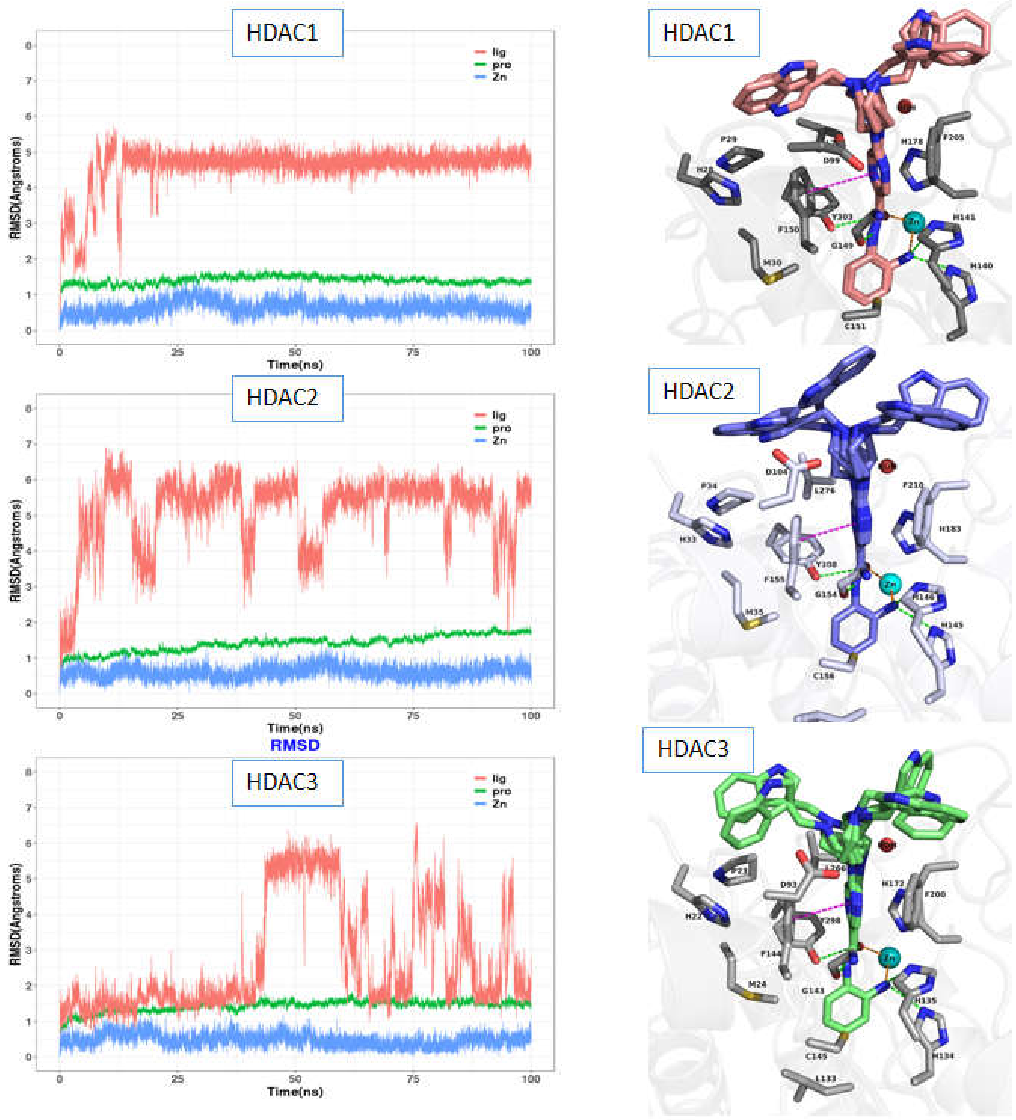

Fig. S7. On the left side of the picture, root-mean-square-deviation (RMSD) analysis of 100ns MD simulation of **19f** in complex with HDAC1/2 and 3 (PDB IDs:4BKX, 4LY1, 4A69, respectively) were shown. Deviation of ligand is represented in red color, of protein in green color and of the zinc ion in blue color. On the right side of the picture, the clusters analysis of **19f** in HDAC1/2 and 3 were shown. Relevant residues are shown in stick representation with dark

grey, white and light grey carbon atoms were shown, respectively. Hydrogen bonds (green dashed lines), metal coordination (orange dashed lines), ionic interactions (cyan dashed lines) and aromatic interactions (magenta dashed lines) between inhibitors and the protein are shown. The zinc ion is shown as cyan colored sphere. The conserved water molecule is shown as red sphere.

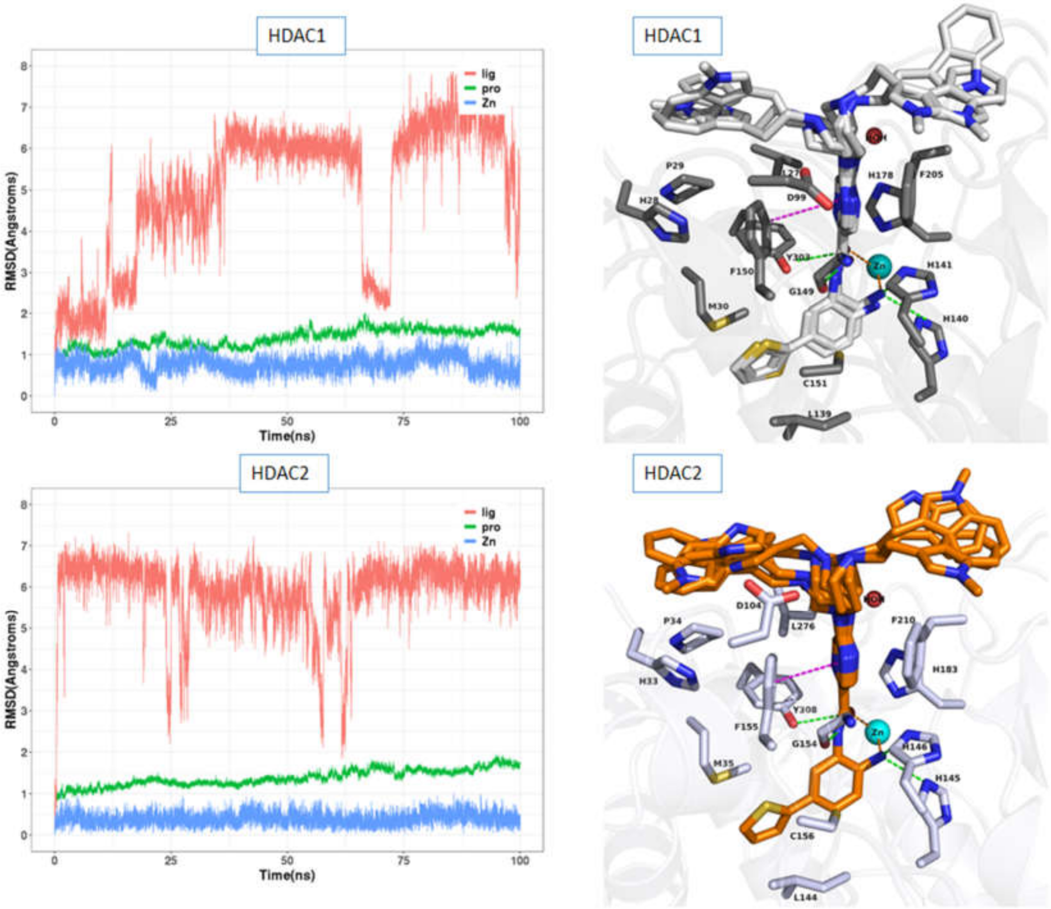

Fig. S8. On the left side of the picture, root-mean-square-deviation (RMSD) analysis of 100ns MD simulation of **21a** in complex with HDAC1/2 and 3 (PDB IDs: 4BKX, 4LY1, 4A69, respectively) were shown. Deviation of ligand is represented in red color, of protein in green color and of the zinc ion in blue color. On the right side of the picture, the clusters analysis of **21a** in HDAC1/2 were shown. Relevant residues are shown in stick representation with dark grey,

white carbon atoms were shown, respectively. Hydrogen bonds (green dashed lines), metal coordination (orange dashed lines), ionic interactions (cyan dashed lines) and aromatic interactions (magenta dashed lines) between inhibitors and the protein are shown. The zinc ion is shown as cyan colored sphere. The conserved water molecule is shown as red sphere.

11. References

1. Ram, S., & Ehrenkaufer, R. E. (1984). A general procedure for mild and rapid reduction of aliphatic and aromatic nitro compounds using ammonium formate as a catalytic hydrogen transfer agent. *Tetrahedron letters*. **1984**, *25*(32), 3415-3418.

2. Mo, C.; Zhang, Z.; Guise, C. P.; Li, X.; Luo, J.; Tu, Z.; Xu, Y.; Patterson, A. V.; Smaill, J. B.; Ren, X., 2-Aminopyrimidine derivatives as new selective fibroblast growth factor receptor 4 (FGFR4) inhibitors. *ACS Med. Chem. Lett.* **2017,** *8* (5), 543-548.

3. Hsieh, H.-Y.; Chuang, H.-C.; Shen, F.-H.; Detroja, K.; Hsin, L.-W.; Chen, C.-S., Targeting breast cancer stem cells by novel HDAC3-selective inhibitors. *Eur. J. Med. Chem.* **2017,** *140*, 42-51.

4. Dichiara, M.; Amata, B.; Turnaturi, R.; Marrazzo, A.; Amata, E., Tuning Properties for Blood–Brain Barrier Permeation: A Statistics-Based Analysis. *ACS chemical neuroscience* **2019,** *11* (1), 34-44.

5. Wagner, F. F.; Weïwer, M.; Steinbacher, S.; Schomburg, A.; Reinemer, P.; Gale, J. P.; Campbell, A. J.; Fisher, S. L.; Zhao, W.-N.; Reis, S. A., Kinetic and structural insights into the binding of histone deacetylase 1 and 2 (HDAC1, 2) inhibitors. *Bioorg. Med. Chem.* **2016,** *24* (18), 4008-4015.
